# Abstracts 13th YES Meeting

**DOI:** 10.1016/j.pbj.0000000000000032

**Published:** 2018-11-01

**Internal Medicine**

**PS005 Frequency of malignant disease in patients with positive stress test**

Aleksandar Milojevic^1^

^1^*Department of Cardiology, Clinical Centre of Serbia*

E-mail address: milojevic94a@gmail.com (Aleksandar Milojevic’)

**Introduction:** Coronary disease and malignancy are two leading causes of death in the modern world. Although considered as two separate entities these two diseases have some similarities and share some common ethiopathogenic mechanisms and have similar risk factors.

**Aim:** With this study we want to evaluate the frequency of malignant diseases on patients who appeared positive on exercise stress test.

**Methods:** We analyzed 172 patients who underwent exercise stress testing standard Bruce protocol. Test was considered positive if there was ST segment depression ≥1 mm of minimal duration of 80ms after the J point on ECG during ergometry or if there were new abnormalities or preexisting abnormalities worsening of left ventricular wall motion in two neighboring segments of the wall detected by echocardiography after the exercise test. Data regarding cardiac events were gathered by telephone interview from patients after 9 years median tracking period.

**Results:** Patients were split into two groups: the first consisted of patients with positive stress test (24; 16%), the second of those with negative stress test (126; 84%). Median tracking period was 92 months (iqr = 24 months). During the tracking period there were 10 (6,7%) newly diagnosed with a malignant disease. In those patients with newly diagnosed malignancy, there was higher frequency of positive stress test (4; 40% vs 20; 14,3% p = 0,032), they were significantly older (74 ± 6 vs 65 ± 11 p = 0,022) and also had slower heart rate recovery (29,3 ± 14,4 vs 42,4 ± 16,1 p = 0,012).

**Conclusion:** Conclusion based on small number of patients showed that patients with positive stress test as marker of coronary disease had larger incidence of malignant disease, what favors similar etiopathogenesis, most likely inflammation, of these two diseases.

**PS007 The role of exercise-induced ST-segment elevation in the aVR lead during the stress echo testing**

Filip Markovic’^1^, Sara Ivaniš^1^

^1^*Faculty of Medicine, University of Belgrade*

*E-mail address: flp.mark@gmail.com (Filip Markovic*’*)*

**Introduction:** Significance of exercised-induced ST-segment elevation (STE) in prediction of LM is still equivocal, especially in the patients undergoing stress echo exercise testing.

**Aim:** With this study we wanted to evaluate the role of exercise-induced STE in lead aVR in predicton of LM/ostial LAD stenosis and to assess its prognostic value for the future cardiac events.

**Methods:** We analyzed 203 consecutive patients who underwent stress echocardiography testing from January 2011. to December 2014. at Clinical Center of Serbia. We analyzed baseline clinical characteristics (classical coronary artery disease risk factors, age, gender), hemodynamic response to exercise (metabolic equivalents, blood pressure drop, heart rate recovery, target sub maximal frequency), rest and stress electrocardiograms as well as baseline/and peak exercise echocardiography images in all patients. We calculated Duke treadmill score and changes in wall motion score index (delta WMSI). Also we followed all 203pts for cardiac events (death, coronary artery bypass surgery, percutaneous coronary intervention, myocardial infarction).

**Results:** The LM/ostial LAD were found in 18/203pts, and in 12/38pts with aVR lead STE. Patients with significant LM/LM equivalents disease were older (66 ± 9yrs. vs. 61 ± 9yrs, p = 0.02), had worse deltaWMSI (0.35 ± 0.16 vs. 0.25 ± 0.16, p = 0.026) and lower Duke score (-5 ± 5 vs. 0 ± 5, p < 0.001). Logistic regression analysis selected age, Duke score and presence of aVR STE (Sn 71, Sp 86%) to be the most significant predictors (p < 0.05) of LM disease during the stress echo testing. Kaplan-Meier curve showed no statistical difference in cardiac events between patients w/wo exercise-induced aVR STE.

**Conclusion:** Exercise-induced aVR lead STE has good specificity and sensitivity for the detection of significant LM disease. Older age, lower Duke score and higher delta WMSI as well may point to significant LM stenosis. Patients with aVR lead exercise-induced STE should be referred to early revascularization due to better prognosis.

**PS028 The prevalence of mutations in VP1 gene of BK polyomavirus in renal transplant recipients**

Teodora Karanovic’^1^, Nevena Kosanovic’^1^, Danijela Karalic’^1^

^1^*Faculty of Medicine, University of Belgrade E-mail address: teodora.karanovic@gmail.com (Teodora Karanovic*’*)*

**Introduction:** Immunosuppressive therapy is a very important factor for reactivation of BK polyomavirus (BKPyV) in renal transplant recipients (RTR). Reactivation of BKPyV in immunocompromised patients can cause life-threatening diseases such as polyomavirus-associated nephropathy. Mutations in the VP1 gene and its BC loop may lead to the selection of more aggressive variants of BKPyV.

**Aim:** The aims of this study were to determine the prevalence of BKPyV DNA in blood and urine samples, distribution of BKPyV subtypes, and identification of nucleotide substitutions in VP1 gene and amino acids in VP1 of BKPyV in RTR.

**Methods:** The study included blood and urine samples of 26 RTR. Semi-nested PCR was used for amplification of 290-nt fragment within the VP1 gene and all positive PCR products were directly sequenced. Sequence analysis was performed by using appropriate bioinformatics tools.

**Results:** The frequency of BK viremia in RTR was 30.8% and viruria was 38.5%. Two subtypes of BK virus - I and IV, were detected among RTR in both blood and urine samples. The predominant BKV subtype was I. The non-synonymous nucleotide substitutions were detected in BC loop and b-sheets within the VP1 gene of BK virus. Statistically significant difference was found in the distribution of mutations within the BC loop and b-sheet among the BK virus isolates from the blood (p = 0.012). The number of non-synonymous nucleotide substitutions was higher in isolates found in blood than in urine samples, but without statistical significance (p = 0.231).

**Conclusion:** Frequent mutations in the BC loop of the VP1 region can cause a change in tropism and the selection of more aggressive variants of BKPyV. Further studies are needed in order to select the RTR with a higher risk for development of polyomavirus-associated diseases.

**PS031 Diastolic dysfunction as a predictor of cardiovascular events: a systematic review and meta-analysis**

Margarida Araújo^1^, Ricardo Ladeiras-Lopes^1,2,3^, Francisco Sampaio^2,3^, Adelino Leite-Moreira^1,3^, Ricardo Fontes-Carvalho^1,2,3^

^1^*Department of Surgery and Physiology, Faculty of Medicine of the University of Porto, Porto, Portugal,*^2^*Cardiology Department, Gaia/Espinho Hospital Centre, Vila Nova de Gaia, Portugal,*^3^*Cardiovascular Research Centre, Faculty of Medicine, University of Porto, Porto, Portugal E-mail address: anamargaridaaraujo@hotmail.com (Ana Margarida Ribeiro de Araújo)*

**Introduction:** Diastolic dysfunction is highly prevalent and a key pathophysiological contributor to several cardiovascular diseases, especially heart failure with preserved ejection fraction. In addition, some evidence suggests diastolic dysfunction is a risk factor for major adverse cardiovascular events.

**Aim:** This study sought to systematically review the evidence and quantify the association between diastolic dysfunction and risk of cardiovascular events and death.

**Methods:** MEDLINE was systematically searched (until October 2017). We included cohort studies that assessed diastolic function in adults from the community, providing a definition of diastolic dysfunction and regarding the development of any cardiovascular event or mortality. For the quantitative analysis, relative risk estimates referring to the comparison of individuals with versus without diastolic dysfunction were combined using a random effects model.

**Results:** Nineteen studies were identified for inclusion in the review, assessing a total of 63,802 participants. The classification system to diagnose diastolic dysfunction was quite different between studies. The median prevalence of diastolic dysfunction in studies including individuals with and without diastolic dysfunction was 35.1% (range 5.3%-65.2%). Comparing diastolic dysfunction with normal diastolic function, the summary relative risk estimate for cardiovascular event or mortality was 3.53 (95% CI: 2.75–4.53; I2 = 85.5%; 9 studies).

**Conclusion:** Despite the heterogeneous definitions found in the literature, the diagnosis of diastolic dysfunction is associated with a 3.53 times increased risk of cardiovascular events or death. This finding highlights the importance of developing easily applicable and consensual criteria for diagnosis, fostering research on effective treatment strategies when diastolic dysfunction is identified in the subclinical stage.

**PS033 Clinical and prognosis implications of other prothrombotic factors in Antiphospholipid Syndrome patients: a single-center cohort analysis**

Mariana Ribas Laranjeira^1^, Gilberto Rosa^2^, Ester Ferreira^2^

^1^*Faculty of Medicine of the University of Porto (FMUP),*^2^*Autoimmune Diseases Group, Department of Internal Medicine, São João Hospital Center, Porto, Portugal E-mail address: mariribaslaranjeira@gmail.com (Mariana Ribas Laranjeira)*

**Introduction:** The development of thrombosis is multifactorial in Antiphospholipid Syndrome (APS), with other inherited and acquired risk factors influencing the thrombotic profile. While inherited thrombophilia are still rare among APS patients, some works suggest a higher prevalence of C and S Proteins deficiencies and factor V Leiden mutation in these patients compared to the general population. Nevertheless, the exact frequency and clinical implications of the presence of these prothrombotic factors in APS are still poorly characterized.

**Aim:** The aim of our study was to evaluate the presence of other prothrombotic factors and their clinical and prognostic implications, and also to verify the role of such risk factors in thrombosis recurrence. In addition we assessed for the presence of non-criteria clinical manifestations.

**Methods:** All patients followed in an Autoimmune Diseases consultation with a diagnosis of APS fulfilling the Sidney revised criteria were included. Data regarding inherited thrombophilia was collected - Activated protein C resistance (APCR); Leiden V Factor mutation; C and S proteins deficiencies; Prothrombin G20210A mutation; and Antithrombin III deficiency.

**Results:** A total of 75 patients were analyzed, with 65.3% corresponding to primary APS. The mean age of the study sample was 40 ± 9.4 years and the mean duration of disease was of 6.57 years ± 4.78 years. Seventeen (22.7%) patients exhibited an inherited thrombophilia: 9 (16.1%) S protein deficiency, 5 (10.2%) APCR, 5 (8.5%) antithrombin III deficiency, 4 (7.1%) C protein deficiency, 2 (4.5%) prothrombin G20210A mutation and 1 (2.0%) Leiden V factor mutation. The presence of inherited thrombophilia did not show statistically significant association with clinical manifestations or recurrence of events.

**Conclusion:** Although with a significant prevalence in the studied sample, the presence of inherited thrombophilia did not display a significant clinical implication. However, positive findings might have been undermined due to the reduced sample size originating an underpowered study.

**PS037 The impact of changes of capillaroscopic findings on to the process of systemic sclerosis**

Nevena Koponja^1^

^1^*Institute of Rheumatology in Belgrade E-mail address: nevena.koponja@gmail.com (Nevena Koponja)*

**Introduction:** Systemic sclerosis(SSc) is a connective tissue disease where microangiopathy results in diffuse fibrosis of the skin and internal organs. Microvascular changes can be dynamic and can easily be assessed by capillaroscopy.

**Aim:** To show what is the average time of transition from one to another phase of scleroderma damage of capillaries and what is the significance of these changes in the SSc process.

**Methods:** In the period January 2012 – December 2017 year, capillaroscopy was performed in 400 patients with SSc at Institute of Rheumatology in Belgrade. The capillaroscopic findings(CF) are divided into four types: non-specific changes, early, active and late “scleroderma types” of capillaroscopic damage. The study included 53 patients who had changes in CF. Retrospective analysis included demographic, clinical and laboratory data of the examinees.

**Results:** In 53/400(13.25%) patients there was a change in CF. At the beginning of the follow-up, 11(21%) patients had non-specific changes, 25(47%) patients had early CF and 17(32%) patients had active CF. The total average transition time from one type to another type of CF was 27.68 ± 35.11 months. Improvement of CF was found in 15(28%) patients and deterioration in 38(72%) patients. In comparison to the initial forms of the CF, patients with non-specific CF had limited form of SSc(91%), diffuse hand swelling(54%), arthralgia/arthritis(45%) and involvement of lungs(48%); patients with early CF had limited form of SSc(64%), sclerodactyly(48%) and involvement of lungs(48%) and patients with active CF had diffuse form of SSc(53%), sclerodactyly(47%), digital ulcerations(35%) and involvement of lungs(65%). These differences in the frequency of involvement of certain organs were not statistically significant (p > 0.05).

**Conclusion:** Capillaroscopy should be performed for every 6 months in patients with SSc to identify rapid progression of the CF (<2 years) to an active and late scleroderma types which are associated with severe damage of internal organs.

**PS049 Analysing the complications of thrombotic therapy in ischemic stroke**

Varahabhatla Vamsi^1^, Tekwani Vinisha^1^, Vedula Ushakiranmayi^2^, Marina Sikorska^3^

^1^*General Medicine Faculty, Zaporozhye State Medical University, Ukraine,*^2^*Konaseema Institute of Medical Sciences, Amalapuram, India,*^3^*Department of Neurology, Zaporozhye State Medical University, Ukraine E-mail address: vamsivarahabhatla@gmail.com (Varahabhatla Sai Krishna Raghu Vamsi)*

**Introduction:** Thrombolytic therapy (TLT) using a recombinant tissue plasminogen activator (rt-PA) is undoubtedly an effective method of treating ischemic stroke by achieving reperfusion of the ischemic region of brain and improving the functional state of patients, but with certain complications.

**Aim:** The aim of our work was to analyze the cases of complications in TLT and to identify the factors that cause their development.

**Methods:** A retrospective analysis of 108 case histories of patients with ischemic stroke, who received TLT in 3–4.5 hours from the onset of the disease, was conducted. The dynamics of the neurological deficit (ND) was evaluated using the NIHSS scale, CT examination was performed before TLT and ultrasonic dopplerography (USDG) next day.

**Results:** Out of 108 case histories, complications after TLT were noted in 26 cases (24.07%). One of the most serious complications was hemorrhagic transformation (HT); 8 patients (30.8%); of them in 2 cases (7.7%) were asymptomatic haemorrhages, 2 cases (7.7%) hematoma up to 30 mm3, and in 3 cases (11.5%) hematoma up to 40 mm3, in 1 case (3.8%) there is a large hematoma with a breakthrough of blood into the ventricles, resulting in a fatal outcome. Frequent complications include reocclusion and rethrombosis. In 11 cases (42.36%) on USDG, reocclusion was noted. In 5 patients (19.2%), rethrombosis was observed, in 6(23.07%) out of 26 patients, at the time of discharge, there was a slight regression of ND by 1–3 points; in 8 (30.8%) there were increased ND, and 3(11.5%) cases had lethal outcome. In 1 case (3.8%), an angioedema was noted after 40 minutes of TLT, after management on next day, there was a regression of the ND by 3 points.

**Conclusion:** Complications can be reduced by mandatory USDG before treatment initiation, termination of TLT during complexity of situation and careful screening of eligible candidates.

**PS050 The significance of myocardial perfusion scintigraphy in planning of resynchronization therapy in patients with ischemic heart disease**

Nevena Kosanovic’^1^, Teodora Karanovic’^1^, Marijana Juga^1^, Dragana Šobic’-Šaranovic’^1^

^1^*Faculty of Medicine, University of Belgrade E-mail address: nevenakosanovic@gmail.com (Nevena Kosanovic*’*)*

**Introduction:** Rising prevalence of heart failure increased the importance of non-invasive diagnostic methods. By becoming the therapy of choice, resynchronization therapy increased it even more. Criteria for patient selection for this therapy, among others, include the myocardial viability.

**Aim:** The aims of this study were to examine the significance of the difference in the values of the heart ejection fraction determined by echocardiography and myocardial perfusion scintigraphy (MPS) and to calculate sensitivity and specificity of echocardiography in the assessment of myocardial viability, compared to MPS.

**Methods:** Forty-three patients underwent echocardiography and MPS. Afterwards CRT-P, CRT-D or ICD was implanted. A viability test was performed using MPS with technetium-99m MIBI. Scoring system, where segments with score 0–3 were viable, and segments with score 4 non-viable, was used to estimate binding of technetium-99m MIBI to the heart muscle. Standard 2D echocardiography was performed, where akinesia was non-viable myocardium. Using Paired t test we compared ejection fraction values obtained by echocardiography and MPS. Accuracy of echocardiography was estimated by calculating sensitivity and specificity of echocardiography in detecting myocardial viability compared with MPS as a „gold standard”.

**Results:** The mean value of ejection fractions on the MPS was 26.3 ± 7.9%, and at the echocardiography 25.9 ± 7.2%. There was no statistically significant difference in the ejection fractions determined by these methods. Echocardiography was not sensitive enough to detect viable myocardium in the basal segment of the septum, apical and medial segment of the lateral wall. Specificity of the echocardiography was not satisfactory for the assessment of viability of the septum, apical and medial segment of the inferior and the apical segment of the lateral wall.

**Conclusion:** Because of the inadequate sensitivity and specificity, echocardiography should not be used to evaluate myocardial viability. MPS is a better method for resynchronization therapy planning.

**PS054 Effective renal plasma flow in starting stages of chronic kidney desease in patients with arterial hypertension**

Nikola Glogonjac^1^

^1^*Faculty of Medicine, University of Novi Sad E-mail address: nglogonjac@gmail.com (Nikola Glogonjac)*

**Introduction:** Chronic kidney disease (CKD) is most commonly caused by arterial hypertension, which has major role not just in causing, but also in the progression through the stages of CKD.

**Aim:** To establish 1) values of effective renal plasma flow (ERPF) in patients with arterial hypertension and CKD; 2) Direction of changes in ERPF relative to normally expected values of this parameter; 3) Relation of changes in ERPF and glomerular filtration rate (GFR) in respondents.

**Methods:** We have done the retrospective analysis of blood samples, 24-hour urine and measurements of radionuclide clearances, clearance of hippuran and clearance DTPA in male patients in the first (I-CKD; n = 30),in the second (II-CKD; n = 30) and in the third stage(III-CKD; n = 30) of CKD. The control group (CG) was formed by 20 fitting age males, without known diseases.

**Results:** Normalised values of ERPF (453.1 ± 47,6 vs. 554.3 ± 19.6 ml/min/1.73m2, p = 0,00), as the deviation of ERPF in relation to normally expected values based on the age (-19.4 (-22.8 -16.0) vs. 9.6 (1.34–12.4) %, p = 0,00), are significantly smaller in respondents in I-CKD group relative to the control group. The statistical significance was not observed in values of GFR between the I-CKD group and the control group (110.4 ± 5.4 vs. 113.1 ± 9.4 ml/min/1.73m2, p > 0.05), while the deviation of GFR relative to normally expected values based on age was significantly higher (13.9 (3.8 – 23.9) vs. -5.6 (-10.3 – 15.4)%, p < 0.05) in I-CKD relative to control group.

**Conclusion:** In patients with arterial hypertension, ERPF values are reduced progressively in first three stages of CKD, unlike the values of GFR, which are elevated in the first, but reduced progressively in second and third stage of CKD.

**PS056 Assessment of macroprolactinemia using PEG metodology**

Dunja Bjelogrlic^1^, Teodora Drazic^1^

^1^*Faculty of Medicine, University of Novi Sad E-mail address: dunja.bjelogrlic@gmail.com (Dunja Bjelogrlic)*

**Introduction:** According to literature data, macroprolactinaemia is mainly cause of hyperprolactinaemia due to two different circulating large forms of prolactin. The relatively low frequency of the symptoms in macroprolactinaemia patients, created the need for a relatively simple, reliable method which can detect large, biologically inactive prolactin molecules such as polyethylene glycol precipitation method (PEG).

**Aim:** The aim of this study was to evaluate aforementioned methodology and its applicability in everyday laboratory practice.

**Methods:** This study included 82 patients who had come to the Center of Laboratory Medicine, Clinical center of Vojvodina in order to determine the serum prolactin levels. Obtained serum samples were poured into two aliquots. The first aliquot was frozen and stored at -20 °C and the other was immediately analyzed. Both kinds of aliquots were treated with the respective, aqueous solution or phosphate buffered saline of Merch 6000. All serum and supernatant samples were analyzed on the automated system Abbott Architect i2000sr. All the results were statistically processed by Data Analysis package.

**Results:** According to results, the lowest supernatant prolactin and calculated Recovery values were obtained from unfrozen serum samples using phosphate buffered saline. Statistical difference was not determined between Recovery values obtained from fresh and frozen serum samples treated with PEG dissolved in phosphate buffer (p = 0.893).

**Conclusion:** Based on the results, today is in use a reproducible method for laboratory determination of macroprolactinaemia based on PEG precipitation (dissolved in phosphate buffer) with equal reliability performance in the range of low, elevated and high prolactin levels.

**PS066 Prognostic role of endothelin-1 and von willebrand factor in pre-eclampsia development**

Vinisha Tekwani^1^, Burra Mithilesh Bharadwaj^1^, Gaidai Nataliya Victorovna^2^

^1^*Zaporozhye State Medical University,*^2^*Zaporozhye State Medical University Department of Obstetrics and Gynaecology E-mail address: vinishatekwani33@gmail.com (Vinisha Tekwani)*

**Introduction:** Preeclampsia affects 2 to 8% of all pregnancies and accounts for 10–15% of maternal deaths worldwide. Endothelial dysfunction is the background for the development of preeclampsia in pregnant women and is a debatable issue.

**Aim:** Our aim was to study the levels of Endothelin-1(ET-1) and Von willebrand factor(vWF) as indicators for determining preeclampsia and eclampsia during early stages of gestation in pregnant women.

**Methods:** We analyzed 75 pregnant women at 3rd maternity hospital, Zaporizhzhya. Dividing them into 2 groups, 1st group consisted 50 women with severe preeclampsia in anamnesis, 2nd group (control group) has 25 healthy women with physiological pregnancy. State of Endothelium and it's parameters like EDV (endothelium dependent vasodilation) of brachial artery, ET-1 and vWF were studied to diagnose the development of preeclampsia in presence of arterial hypertension. Levels of ET-1 and vWF were determined by radio-immune and immune-enzyme methods. The concentration of ET-1 and vWF were determined in picograms per milliliter (Pg/ml).

**Results:** During the analysis of pregnant women the following complications were in 1st group: preeclampsia-13 (26.1%), abortive threat-21(41.3%), preterm delivery-5(10.87%), SCRP (serum C- reactive protein)-3(6.5%), foetal distress-4(8.7%). Level of ET-1 in 1st group: 0.16 ± 0.02(18–22weeks of gestation), 0.27 ± 0.04(28–32weeks); In 2nd group: 0.04 ± 0.01(18–22 weeks), 0.06 ± 0.01(28–32 weeks). Concentration of vWF in 1st group:1.11 ± 0.06 (18–22 weeks), 1.41 ± 0.07(28–32 weeks); In 2nd group: 0.41 ± 0.04(18–22weeks), 0.83 ± 0.09(28–32weeks). Concentration of EDV(in %) of brachial artery in 1st group: 11.70 ± 1.15 (18–22weeks), 10.80 ± 1.14(28–32 weeks) and in 2nd group: 28.00 ± 1.63 (18–22weeks), 27.13 ± 1.54 (28–32weeks) respectively.

**Conclusion:** In anamnesis of pregnant women with preeclampsia during early gestation (18–22 weeks) there is an increase of ET-1 by 75%, vWF by 63% and reduction of EDV of brachial artery by 58% compared to healthy control group. Therefore, determining their plasma concentrations can help to detect Preeclampsia and by providing required medical care, complications can be reduced.

**PS072 Additional findings among patients diagnosed with pulmonary CT angiography due to suspected pulmonary embolism**

Katarzyna Ciuk^1^, Justyna Teęczar^1^, Maciej Polak^1^, Anna Gajdosz^1^, Jakub Wnuk^1^, Szymon Ciuk^1^

^1^*Jagiellonian University in Kraków E-mail address: kataryna962@gmail.com (Katarzyna Ciuk)*

**Introduction:** CT angiography (CTA) is a golden standard in diagnosing pulmonary embolism (PE). Although symptoms may be indicative, the result of the examination often excludes the suggested diagnosis. However, there are situations when other significant features are imaged accidentally.

**Aim:** The aim of the study was to evaluate the most common incidental findings discovered in CTA due to PE clinical suspicion.

**Methods:** There were 201 recorded cases (males 52.2%; mean age 66.3 ± 15.5, range 20–98 years) of pulmonary CTA performed due to suspected PE in archives of the Department of Radiology, University Hospital in Cracow between June and October 2017. CT scan range: from lung apices to adrenal glands. All cases were analysed in term of described lesions. Statistical significance was set at p < 0.05.

**Results:** Pulmonary embolism was confirmed in 14.9% of cases. These patients were significantly younger than these without PE (60.3 ± 15.2 vs. 67.4 ± 15.4 years, p = 0.020). Age group with the highest rate of confirmed PE was: 40 – 49 years (40.0%). The most common additional findings among these patients were spinal osteoarthritis (43.3%), lung tumour (20.0%), calcified atherosclerotic plaques in aorta and systemic arteries (16.7%), liver steatosis (13.3%). Liver steatosis (13.3% with PE vs. 4.1% without, p = 0.040, OR = 3.604) and lung tumour (20.0% vs. 7.6%, p = 0.032, OR = 3.038) coexisted significantly more often with PE. Abnormalities within lungs and pleura were detected in 72.1% of patients without PE: 34.5% pneumonia, 30.4% pleural effusion, 21.1% atelectasis. In 10.0% of patients neither embolism nor pulmonary lesions were found. In this group, the most common findings were spinal osteoarthritis (65.0%), calcified atherosclerotic plaques in coronary arteries (20.0%), pericardial effusion (15.0%), cardiomegaly (15.0%), hiatal hernia (15.0%).

**Conclusion:** Older patients are more prone to diseases which mimic symptoms of pulmonary embolism (pneumonia, pleural effusion, nerve compression due to spinal osteoarthritis). Pulmonary embolism is likely to coexist with lung tumour and liver steatosis.

**PS077 The impact of metformin therapy on the quality of metabolic control in children and adolescents suffering from diabetes**

Andjela Zovkic^1^, Vladana Stankovic^1^

^1^*Pediatric Internal Diseases Clinic, Faculty of Medicine, University of Nis, Nis, Serbia E-mail address: andjela.zovkic@gmail.com (Andjela Zovkic)*

**Introduction:** There is an increasing number of young people with signs of both main types of diabetes - they are obese with signs of insulin resistance, as well as signs of autoimmunity. Therefore, for each adolescent without diabetic ketoacidosis and rapid remission, especially those that are obese, metformin should be included due to its numerous metabolic and cardiovascular positive effects.

**Aim:** The aim was to show the role of metformin in the treatment of children and adolescents with diabetes at the Clinic for Children's Diseases in Nis.

**Methods:** By analyzing blood samples of 240 patients treated between 2015 and 2017, the values of HbA1c, BMI, total cholesterol, HDL, LDL and triglycerides were determined, as well as the age and duration of the disease in relation to C-peptide. Differences between the two groups were compared using the Mann Whitney test, and the comparison between the three groups was carried out using the Kruskal-Wallis test. A comparison of the frequency of certain parameters was performed using the Chi-Square test. Data analysis was performed using SPSS version 16.

**Results:** In patients treated with metformin, a statistically lower concentration of HbA1c (median 7.45% vs. 8.57%, p < 0.001) was found, while the concentration of C peptide was higher (0.48 ng/mL vs. 0.04 ng/mL, p < 0.001) compared to patients who were not treated with metformin. The group allocated metformin also showed statistically significant lower BMI values (18.96 vs. 20.10, p = 0.004), total cholesterol (4.18 mmol/L vs. 4.60 mmol/L, p = 0.004), HDL (1.41 mmol/L vs. 1.63 mmol/L, p = 0.019) and triglycerides (0.80 mmol/L vs. 0.94 mmol/L, p = 0.004).

**Conclusion:** Metformin has been shown to significantly improve the quality of metabolic control in children and adolescents suffering from diabetes.

**PS082 Total immunoglobulin E level in aspirin-induced asthma patients with subjected to aspirin desensitization- a one-year follow-up study**

Anna Urban'ska^1^, Adrianna Kot^1^, Ewa Zabiegło^1^, Wiktor Socha^1^, Kinga Pajdzik^1^, Katarzyna Ewa Tyrak^1^, Lucyna Mastalerz^1^

^1^*Jagiellonian University Medical College E-mail address: anna.uurbanska@gmail.com (Anna Patrycja Urban*'*ska)*

**Introduction:** Aspirin Exacerbated Respiratory Disease (AERD) refers to asthma, chronic rhinosinusitis with nasal polyposis and aspirin hypersensitivity. AERD subjects often have high levels of IgE in their blood. Aspirin desensitization is regarded as an effective and well-tolerated therapy for patients with AERD.

**Aim:** The aim of our study was to evaluate the influence of aspirin desensitization on total IgE concentration in blood in patients with AERD.

**Methods:** This is a prospective study of twenty-one AERD individuals subjected to 52 weeks of aspirin therapy. All participants were hospitalized two times over the period of one year. At baseline and during each follow-up visit blood was collected to evaluate total IgE concentration. Additionally, the participants were asked to complete the Visual Analogue Scale (VAS) for nasal symptoms and the Asthma Control Test (ACT) at each follow-up visit. The history of asthma exacerbations was also recorded. For statistical analysis, summary statistics and Student's t-test were used.

**Results:** 52 weeks of aspirin desensitization did not influence baseline total IgE concentration in blood (p = 0.37). The rate of protocol-defined exacerbations in the year of aspirin desensitization decreased significantly in reference of the year prior to AD (p = 0.001). The significant improvement was also observed in ACT score (p = 0.024) and VAS score (p = 0.008).

**Conclusion:** Aspirin desensitization do not cause a significant decline in total immunoglobulin E level in aspirin-sensitive individuals. Despite of this objective tools, patients report improvement of asthma control and nasal symptoms. The number of asthma exacerbations decrease. These results suggest that aspirin desensitization is effective.

**PS091 The use of laser doppler flowmetry in complex diagnostic of the risk of venous thromboembolism in pregnant women**

Daria Andreevna Nazarkina^1^, Igor Leonidovich Davydkin^1^, Olesya Evgenyevna Danilova^1^

^1^*Samara State Medical University E-mail address: d.a.nazarkina@mail.ru (Daria Nazarkina)*

**Introduction:** Venous thromboembolism is one of the leading causes of death in post-partum period. Frequency of thromboembolic complications varies from 0,6 to 5,0 per 1000 pregnant women.

Existing standarts in leading women during pregnancy do not provide examination of hemostatic system for all women. As a result both doctor and patient learn for the first time about tendency to hypercoagulation after the first episode of thrombosis.

**Aim:** The possibility of introduction of modern noninvasive technologies for early detection of hypercoaguation tendency and disturbance of microcirculation processes became our main goal.

**Methods:** We examined 130 pregnant women from different risk groups on base of Samara State Polyclinic N^o^15. All women included in the study were interviewed to gather anamnesis and underwent laser doppler flowmetry. Microcirculation test was performed with laser Doppler flowmetry using «LAKK-OP» According to the risk of VTE all women were divided into three groups.

**Results:** Results of the comparison of LDF examination with anamnesis and results of physical examination correlate with high VTE risk of pregnant women with average and high risk. Method of laser Doppler flowmetry can be used for early detection of hypercoagulation tendency and disturbance of microcirculation processes in pregnant women.

**Conclusion:** Microcirculation disturbance indicates dysfunction of the endothelium of pregnant women that leads to VTE, prematurity and death in post-partum period. Introduction of new noninvasive methods to examine hemostatic system will reduce the VTE risk.

**PS093 The relationship between anxiety disorders and metabolic syndrome, indicators of the biochemical atherosclerosis risk in male smokers**

Ieva Ramanauskaité^1^, Viktorija Andrejevaité^2^

^1^*Faculty of Medicine, Vilnius University, Vilnius, Lithuania,*^2^*Internal Diseases, Family Medicine and Oncology Clinic E-mail address: ieva.ramanauskaite7@gmail.com (Ieva Ramanauskaitë)*

**Introduction:** Cardiovascular disease (CVD) continues to be the leading cause of morbidity and mortality worldwide. Anxiety disorders enhance the development of CVD by both behavioural (unhealthy lifestyle habits) and biological pathways.

**Aim:** To assess the relationship between anxiety disorders and metabolic syndrome, biomarkers of lipid metabolism in male smokers.

**Methods:** Smoking (≥ 10 cigarettes per day, ≥ 10 years) males with anxiety disorders (research group) and smoking males without anxiety disorders (control group) participated in the research, in 2012–2014 they came to the Cardiologic prevention sub-division of Vilnius University hospital Santaros clinics for check up. The age of male smokers was 40–54 years. Statistical analysis was performed with, IBM SPSS “.

**Results:** The final data analysis included 257 participants. There were 63 (24.5%) smokers in the group with anxiety disorders and 194 (75.5%) smokers without anxiety disorders. Metabolic syndrome (MS) was diagnosed in 210 (81.7%) study subjects. The prevalence of MS components: abdominal obesity 85.2%, arterial hypertension 79.8%, hypertriglyceridemia 68.9%, hyperglycemia 58%, and low HDL-Ch 47.1%. The frequency of MS was similar in both study groups and was 82% (n = 159) and 81% (n = 51). Abdominal obesity rate was higher in participants without anxiety disorders compared to study group with anxiety disorders (88.1% vs 76.2%, p = 0.02). Hypertriglyceridemia was more common in smokers with anxiety disorders compared to smokers without anxiety disorders (79.4% vs 65.6%, p = 0.048). Dyslipidemia was diagnosed for 252 (98.1%) participants. The most common lipid disorder was increased total cholesterol level 228 (88.7%). The average total cholesterol, LDL-Ch, DTL-Ch concentrations were no statistically significantly different between study groups.

**Conclusion:** Anxiety disorders were associated with hypertriglyceridemia in male smokers. There was no relation between anxiety disorders and indicators of lipid and lipoprotein metabolism in male smokers: blood levels of total cholesterol, as well as high- and low-density lipoproteins.

**PS099 Electrocardiographic characteristics of active athletes - comparison of European and Seattle criteria**

Bojana Šaroškovic’^1^, Jelena Vidovic’^1^

^1^*Faculty of Medicine; University of Novi Sad; Serbia E-mail address: bojana.saroskovic@gmail.com (Bojana Šaroškovic*’*)*

**Introduction:** Continuous physical activity causes adaptive changes on all human organ systems, the most pronounced being the ones on the cardiovascular system. In order to identify pathological changes in the electrocardiographic record of active athletes, European (ESC) and modified Seattle criteria were set up to prevent sudden cardiac death.

**Aim:** Determine the prevalence of electrocardiographic abnormalities according to current ESC and Seattle criteria in active athletes. Examine the relationship between the ESC and Seattle criteria with an echocardiographic finding.

**Methods:** The study included 120 healthy subjects of both genders divided into 3 groups: Group I - 40 subjects who are actively engaged in athletics; Group II - 40 subjects who are actively engaged in basketball; Group III - control group - 40 subjects who are not engaged in sports. All subjects completed a questionnaire related to the extent and scope of physical activity, risk factors and genetic load. All subjects had a twelve-channel electrocardiography (ECG) record, as well as a standard echocardiographic examination.

**Results:** The study showed a statistically significant difference in the prevalence of ECG abnormalities that are not related to physical activity according to the ESC criteria between the test and control group and is 10 (25%) in the group engaged in athletics and 8 (20%) in the group engaged in basketball, comparing to control group. According to Seattle criteria, a statistically significant difference in the incidence of pathologic ECG changes between the tested groups and the control group was not observed. There is a statistically significant difference between the number of registered ECG changes according to European criteria and observed echocardiographic changes, while there is no statistically significant difference between ECG changes according to Seattle criteria and echocardiographic findings.

**Conclusion:** The results of the study have shown that the use of Seattle criteria in relation to European criteria in interpreting ECG records of active athletes significantly reduces the number of pathological findings. Pathological changes in the electrocardiographic record according to Seattle criteria best correlate with echocardiographic changes.

**PS107 Effectiveness of garlic (Allium Sativum) as antimicrobial agent against bacteria causing urinary tract infection**

Muhammad Zuhal Darwis^1^, Andi Muhammad Ariansyah^1^, Muhammad Nur Anshari Syakir^1^

^1^*Medical Faculty Universitas Muslim, Indonesia E-mail address: mzuhaldarwis@gmail.com (Muhammad Zuhal Darwis)*

**Introduction:** Urinary Tract Infection, is the presence of microorganisms in the urine that indicate the existence of pathogenic microbes in the urinary tract causing inflammation. Some of the bacteria that normally cause urinary tract infections are Staphylococcus aureus, Escherichia coli, Klebsiella pneumoniae, Pseudomonas aeruginosa, and Proteus sp. Generally found in women because of shorter urethra. The emergence of antibiotic resistance, leading to treatment failure in some cases. One strategy to overcome this is to use alternative therapies, one of them by using herbal plants, namely garlic (Allium Sativum) which has antimicrobial effects.[1]

**Aim:** To determine the effectiveness of garlic extract (Allium Sativum) against bacteria that cause urinary tract infections by measuring the diameter of the minimum inhibitory zone formed.

**Methods:** The study used true experimental post test with disc diffusion methods. Take garlic extract with 50% (5 gr garlic extract in 10 ml water as a solvent) and 80% (8 gr garlic extract in 10 ml solvent) concentration which then dripped on the medium for growth of bacterial culture causing urinary tract infection (Staphylococcus aureus, Escherichia coli, Klebsiella pneumoniae, Pseudomonas aeruginosa, and Proteus sp) then measured diameter of the minimum inhibit zone that formed and compared it with a positive control of 500 mg amoxicillin

**Results:** The inhibitory zone diameter of at least 50% concentration of garlic extract showed intermediate results in Pseudomonas aeruginosa bacteria with a diameter of 13.17 mm, and not sensitive to other bacteria. At 80% concentration showed sensitive results in Pseudomonas aeruginosa and Escherichia colibacteria with diameter 17.27 mm & 15.5 mm, the result almost equivalent compared with amoxicilin, with diameter 20.38 mm & 24.23 mm.

**Conclusion:** Garlic extract (Allium Sativum) with 80% concentration has antimicrobial effect against Pseudomonas aeruginosa and Escherichia coli bacteria

**References**

1. Kumar et al. 2009. Antibacterial activity of allicin from Allium sativum against antibiotic resistant uropathogens. The Internet Journal of Infectious Diseases. Vol 8, No. 1.

**PS108 Correlation between elevated blood glucose and the incidence of ulcers in type 2 diabetes mellitus patients in Dr. Wahidin Sudirohusodo Makassar Hospital in 2016**

Muhammad Nur Anshari Syakir^1^, Muhammad Zuhal Darwis^1^, Andi Muhammad Ariansyah^1^

^1^*Medical Faculty Universitas Muslim, Indonesia E-mail address: ansharisyakir@gmail.com (Muhammad Nur Anshari Syakir)*

**Introduction:** Diabetic ulcers are a form of chronic complications from Diabetes Mellitus (DM). In the United States, estimate the number of people with DM in the next 25 years will increase two folds.[1] Diabetic ulcers is an open wound on the skin surface that can be accompanied by local tissue death. The prevalence of diabetic ulcers in Indonesia is about 32%, 15% amputation rate, and 30% mortality. Some of the things are examined in perceiving a controlled DM, such as: Random and fasting blood glucose, and HbA1c. With the examination of blood glucose levels is expected to provide direction on what should be done for DM patients to avoid complications as well as diabetic ulcers that may occur.

**Aim:** To determine the relationship between elevated blood sugar and HbA1c on the incidence of ulcers in patients with type 2 Diabetes Mellitus in Dr. Wahidin Sudirohusodo Hospital, Makassar in 2016.

**Methods:** The type of research is analytical research using case control method by looking at secondary data in medical record. The samples of research are 66 people.

**Results:** Based on the results of the research showed that patients with high random blood glucose (≥200 mg/dl) and fasting blood glucose (≥126 mg/dl) have higher incidence of diabetic ulcer by 9.1% and 5.3%. Patients with high HbA1c (≥6.5%) also have higher incidence of diabetic ulcer by 25%.

**Conclusion:** The result of bivariate analysis showed that there was a significant correlation between high level blood glucose level and HbA1c on the occurrence of ulcers in patients with type 2 diabetes (p < 0.05).

**References**

1. Huang, E.S., Basu, A., O’Grady, M., Capreta, J.C. 2009. Projecting the Future Diabetes Population Size and Related Costs for the U.S. Diabetes Care, 32: 2225–9.

**PS109 The influence of giving skin extracts Arumanis mango (***Magnifera indica***L.) towards level of uric acid in white rat males (***Rattus norvegicus***)**

Andi Muhammad Ariansyah^1^, Muhammad Nur Anshari Syakir^1^, Muhammad Zuhal Darwis^1^

^1^*Medical Faculty Universitas Muslim, Indonesia E-mail address: Andimuhammadariansyahn@gmail.com (Andi Muh. Ariansyah)*

**Introduction:** Hyperuricemia can lead to several disease, like arthritis gout and nephropathy gout. There are several ways to maintaining normal level of uric acid in blood, for example: eating less food that contain high purine, consume drugs that lowering the level of uric acid, like Allupurinol, and there are also several fruit that has reducing uric acid level, which we can find it easily, consume it daily and less side effect, for example: Skin of mangoes, which contains Flavonoids and antioxidants mangiferin that can inhibit formation of uric acid.[1]

**Aim:** To determine the influence of giving skin extracts arumanis mango (*Magnifera indica* L.) towards level of uric acid in white rat males (*Rattus norvegicus*)

**Methods:** This research used the pre-post true experimental test design by using 20 rat males hyperuricemic induce by chicken liver juice and potassium oxonate and then divided to control and treatment group, each group consist of 10 rats. The control group were given a placebo sodium CMC 2 gr/dl and the treatment group were given the skin extract of Arumanis mango (*Mangifera indica* l.) 83 mg/dl with a total of 14 days of intervention.

**Results:** The results showed average levels of uric acid in blood of rats (*Rattus norvegicus*) on the 8th day of giving skin extract arumanis mango (*Mangifera indica* l.) is 9.46 mg/dl and on the 15th day is 1.89 mg/dl. In the end of experiment, there is a fairly significant difference in the levels of uric acid between control group is 3.5 mg/dl and group treatment is 1.8 mg/dl.

**Conclusion:** Based on the research we found there is influence of skin extract Arumanis mango (*Mangifera indica* l.) in lowering the levels of blood uric acid in rats (*Rattus norvegicus*).

**References**

1. Shah, K. A., Patel, M. B., Patel, R. J., & Parmar, P. K. (2010). Mangifera Indica (Mango). Pharmacognosy Reviews, 4(7), 42–49. http://doi.org/10.4103/0973-7847.65325

**PS110 Clinical and laboratory characteristics of the pyoderma gangrenosum patients: a retrospective research in Vilnius**

Algis Jaraminas^1^, Jurgina Useliene^2^, Milda Krivickaite^2^, Jurate Grigaitiene^2,3^

^1^*Faculty of Medicine, Vilnius University,*^2^*Center of Dermatovenereology, Vilnius University Hospital Santaros Clinics,*^3^*Vilnius University Clinic of Infectious and Chest Diseases E-mail address: aljaraminas@gmail.com (Algis Jaraminas)*

**Introduction:** Pyoderma gangrenosum (PG) is a rare neutrophilic dermatosis, characterized as a painful undermined ulceration of the skin with irregular violaceous border often affecting lower limbs. Due to its confusing clinical presentation, PG is often misdiagnosed and treated inappropriately.

**Aim:** To evaluate clinical and laboratory characteristics, concomitant diseases, delay in the diagnosis and applied treatment measures of the PG patients.

**Methods:** A retrospective study based on diagnostic criteria has been conducted between 2007 and 2017. Medical histories of 32 patients who met the diagnostic criteria were selected for descriptive data analysis.

**Results:** 16 (50%) patients were female. The mean age of the disease onset was 55,4 years, 65,4% of patients had elevated BMI. The average time to diagnosis was 14,7 months. In 43,8% of the cases, PG was misdiagnosed as other diseases. In 84,4% of the cases, the disease was presented as a painful ulceration, 31,3% experienced pathergy phenomenon, predominant localization was the lower limb (62,5%). A surgical procedure as aggravating factor was indicated in 15,6% of cases. The most prevalent clinical form was ulcerous PG (53,1%). Leukocytosis was present in 28,1% and neutrophilia in 37,5%. C-reactive protein (CRP) was elevated in 71%. Secondary bacterial colonization of lesions occurred in 59,4% of patients. At least one underlying systemic disease has been reported in 81,3%. PG-associated diseases were observed in 37.5%, with a predominance of ulcerative colitis and tumors. The most commonly used systemic treatments were steroids (90,6%), antibiotics (50%), immunomodulators (9,3%). The most often used topicals were antiseptics (87,5%) and corticosteroids (46,9%).

**Conclusion:** PG was more common in individuals over 50 years old. The disease is difficult to diagnose and often associated with underlying systemic diseases. Many of the patients were overweight or obese and had elevated CRP levels, secondary bacterial colonization. Neutrophilia occurred in about a third of the patients.

**PS111 The influence of arterial hypertension on the risk of various venous thrombosis types development**

Sara Dragovic^1^, Biljana Vuckovic^1^

^1^*Faculty of Medicine; University of Novi Sad; Serbia E-mail address: dr.agovicsara10@gmail.com (Dragovic Sara)*

**Introduction:** Thrombosis represents a lifelong process resulting in formation of blood cloth, that is the thrombus in blood vessels and/or hearth. It is characterized by heightened incidence and recurrence. From clinical perspective the classification with the respect to the sort of blood vessel thrombosis is of great importance. There is a provoked and unprovoked thrombosis due to the risk of recidive and further therapeutic possibilities.

**Aim:** Investigate the influence of the arterial hypertension on the risk of various thrombosis type development.

**Methods:** The research involved 200 participants, classified in two groups: a group of patients that previously had deep vein thrombosis or pulmonary thromboembolism and healthy control group. These two groups of participants were further classified, with the respect to the value of the arterial blood pressure to the participants with or without hypertension. The group of patients was also classified to the ones with provoked and primary (unprovoked) venous thrombosis. The analysis was adapted to “confounding” factors in order to avoid the bias within the obtained results and to assure the interpretation of the results is valid.

**Results:** The analysis of the presence of classic risk factors for venous and arterial thrombosis formation showed that they are much more frequent in patients than the healthy participants, as expected. With the respect to the sort of venous thrombosis, the more frequent one was unprovoked thrombosis, where there is a higher percentage of people with hypertension compared to the group of patients with provoked deep vein thrombosis. Within the group with unprovoked venous thrombosis, OR is 3.12 (CI 1.34–7.27), i.e. fully assimilated OR is 2.93 (CI 1.13–7.60) from which we draw conclusion that hypertension significantly increases the risk of spontaneous venous thrombosis formation, almost three times.

**Conclusion:** Arterial hypertension triples the risk of spontaneous venous thrombosis formation.

**PS113 The influence of dietary supplements consumption during pregnancy on the weight of newborns**

Jurgita Pilvelytë^1^, Samanta Plikaitytë^1^

^1^*Faculty of Medicine, Vilnius University, Lithuania E-mail address: jurgitap3@gmail.com (Jurgita Pilvelytë)*

**Introduction:** Low weight newborns have a higher risk of having respiratory problem, increased risk of infection and etc. So we made a study to find out if taking dietary supplements may affect the weight of newborns

**Aim:** Evaluate the influence of dietary supplements consumption during pregnancy on the weight of newborns

**Methods:** The study was conducted using an original anonymous questionnaire consisting of 35 questions to find out the physical data of the pregnant women and newborns and the main factors that affect the condition of pregnant women and newborns. A survey was conducted by interviewing women who gave birth in the Department of Obstetrics of Vilnius University Santaros Clinic. According to provided data, women were divided into 2 groups. One group included women who took dietary supplements during pregnancy (only those who consumed supplements like calcium, magnesium, zinc, vitamins A, B, C, omega fatty acids, complex vitamins). The other group included women who did not take dietary supplements. Newborns were divided into 6 subgroups according to their weight: 1st < 5 percentiles; 2nd 5–10 percentiles, 3rd 10–25 percentiles, 4th 25–75 percentiles, 5th 75–95 percentiles, 6th < 95 percentiles. The relation between the newborns weight and the use of supplements during pregnancy has been researched. Statistical analysis of data was performed using SPSS and MS Excel software. The results were statistically significant at p < 0.05

**Results:** 62% (116) women consumed dietary supplements, 38% (71 women) did not. In the first group the numbers of newborns in subgroups were: 1st 1, 2nd 3, 3rd 19, 4th 51, 5th 40, 6th 2. In the second group: 1st 4, 2nd 2, 3rd 14, 4th 33, 5th 16, 6th 2. The weight of newborns was not statistically different (p = 0.11) in both groups. Women's groups did not differ by other factors (nutrition, smoking, alcohol consumption, previous pregnancies and their outcomes). The smoking factor was not statistically significant (p = 0.76)

**Conclusion:** The consumption of dietary supplements has no influence on newborns weight

**PS114 The influence of calcium supplements consumption on lowering the development of gestational hypertensive conditions**

Samanta Plikaitytë^1^, Jurgita Pilvelytë^1^

^1^*Faculty of Medicine, Vilnius University, Lithuania E-mail address: samanta.plikaityte@gmail.com (Samanta Plikaitytë)*

**Introduction:** Hypertensive disorders complicate about 5–10% of pregnancies. Based on WHO data, it has been established that hypertension is one of the most important causes of maternal mortality in developed countries, and causes 16% pregnancy deaths

**Aim:** Compare whether women who took calcium during pregnancy have a lower risk of developing gestational hypertension than those who did not use calcium

**Methods:** The study was conducted by surveying women who gave a birth in the Midwifery Department of Vilnius University Santaros Clinics from November 2017 till April 2018. The study was conducted using an original anonymous questionnaire in which women had to answer questions about their pregnancy. Some of the questions were: if they had complications during pregnancy, name them, provide information about the use of supplements (calcium and others) during pregnancy, other factors (age, smoking, previous pregnancies, their outcomes, arterial blood pressure before pregnancy). Two groups of women were formed: in one group women had hypertensive disorders during pregnancy and in the other women did not have hypertensive disorders. The use of calcium supplements in these groups has been researched. Statistical analysis of data was performed using SPSS and MS Excel software, the results were taken as statistically significant at p < 0.05

**Results:** 185 women participated in the study, of which 149 did not have pregnancy induced hypertensive disorders and 36 who had hypertensive disorders during the pregnancy (2 women had preeclampsia, 3 had eclampsia, 1 had preeclampsia, eclampsia and pregnancy hypertension, 30 had pregnancy hypertension). Among pregnant women with hypertension, 11.1% of women (4 women) were taking calcium during pregnancy and 88.9% (32 women) were not taking calcium additives. Among non-hipertensive women - 61.7% of women (92 women) took calcium supplements and 38.3% (57 women) did not use calcium supplements during the pregnancy. The use of calcium supplements statistically significantly (p < 0.001) reduces the risk of developing gestational hypertension.

**Conclusion:** The consumption of calcium supplements may lower the possibility of gestational hypertensive conditions.

**PS120 Diagnostic of ahalases with magnetic resonance**

Bojana Potpara^1^, Tarik Plojovic’^1^

^1^*University of Belgrade E-mail address: potparabojana95@gmail.com (Bojana Potpara)*

**Introduction:** Achalasia is a chronic motor esophageal disorder characterized by the absence of peristalsis of the esophageal body and lower esophageal sphincter (LES) to relax during the swallowing act. Although, manometry is considered as a “gold standard”, radiological diagnosis is still crucial in the diagnostic algorithm of achalasia. The development and technological improvement of magnetic resonance (MRI) enables its application.

**Aim:** Presentation of MRI capabilities and its limitations, MRI examination techniques and testing corellation between functional MRI (fMRI) and manometry, in the assessment of the type of achalasia.

**Methods:** Based on the results of manometry, one of the three types of achalasia is identified. We observed the luminal width of the esophagus, thickness of the esophageal wall, peristaltic activity and its emptyng. The Kapa test has assessed the degree of agreement between MRI and manometry as a reference method.

**Results:** According to three types of achalasia we identified 14 (27.5%) patients with type 1, 31 (60.8%) with type 2 and 6 (11.8%) with type 3. Based on the mean values of esophageal wall thicknes, we found statistically significant difference measured at the axial plane at the left atrial level, between type 1 and 3 (p = 0.015), as well as type 2 and 3 (p = 0.021). The statistically significant difference due to wall thickness was found at the axial level measured 3 cm above the esophagogastric junction (EGJ) between type 1 and type 3 (p = 0.001) and type 2 and 3 (p = 0.009). The value of the coefficient of agreement between manometry and MRI was 0.672 (p < 0.001).

**Conclusion:** Based on agreement between manometry and MRI we conclude that MRI can be usefull diagnostic method in the assessment of LES function and differentiation achalasia types.

**PS121 Aspergillus flavus and Aspergillus ochraceus as producers of hemostaticaly active proteins**

Anastasa Orekhova^1^, Daria Bednenko^1^, Alexander Osmolovskiy^1^

^1^*Lomonosov Moscow State University E-mail address: stasya77@list.ru (Orekhova Anastasia)*

**Introduction:** New direction in the use of micromycetes enzymes is the development of diagnosticums for determining the key components of the hemostasis system, that is cheaper and more available than now used proteases isolated from the human bloodstream, recombinant proteins or native proteinases, derived from snake venom.

**Aim:** The aim of this work was selection of optimal concentrations range of extracellular protease-activator of micromycete Aspergillus ochraceus and A. flavus O-1. These proteases have protein C, factor X, t-PA, fibrin, collagen, elastin, plasmin and thrombin activator activity.

**Methods:** Cultivation of micromycetes were performed under submerged conditions. For isolation of the proteases from cultural fluid, isoelectrofocusing was performed by the Vesterberg method. During chromogenic detection, an enzymatic label catalyzes the conversion of a chromogenic substrate to produce a colored precipitate. Fraction with the maximum of the activator activity after chromogenic detection was collected.

**Results:** It was demonstrated the same results with specific activity proteinases of Aspergillus ochraceus and with Protac^®^ and RVV-X^®^. Similar experiments were conducted in the presence of factor X deficient plasma and protein C deficient plasma. It was found that concentration of protein C is identical with Protac^®^ (32,7% ± 5%) diagnostic system and specific activity proteinases of Aspergillus ochraceus (31,5% ± 5%). Uniform diagnostics interval was detected with RVV-X^®^ diagnostic system (36,6% ± 4%) and proteinases of Aspergillus ochraceus (37,4% ± 4%).

A. flavus O-1 protease has direct activity to plasmin and thrombin – 0.038 and 0.048 μmol pNA/ml/min respectively. Moreover, enzyme shows activator activity to t-PA, X factor and PC – 0.045, 0.029 and 0.033 μmol pNA/ml/min respectively. Also, activator to plasminogen activity was detected.

**Conclusion:** To sum up, proteases produced by Aspergillus ochraceus and A. flavus O-1 are very perspective for protein C, X factor, t-PA, plasmin and thrombin diagnostics. This method may be cheaper and easier than another methodics.

**PS127 A retrospective study of the clinical features of genital endometriosis and its treatment**

Bala Krishna Reddy Vadigala^1^, Raja Raghupathy Rao Cherukuri^1^, Myhaelo Ivanovich Pavliuchenko^2^

^1^*Zaporozhye State Medical University,*^2^*Department of Obstetrics and Gynecology, Zaporozhye State Medical University E-mail address: balakrish3320@gmail.com (Vadigala Balakrishna Reddy)*

**Introduction:** Genital endometriosis is a common benign disease of the female reproductive system. The medical and social significance of this problem is due to the fact that clinical manifestations are most often observed during the reproductive period, significantly reducing the quality of life and reproductive capabilities of women.

**Aim:** To study the clinical features of the course of genital endometriosis and the results of a combination of surgical and hormonal treatment.

**Methods:** Analysis of the clinical course and treatment in 95 patients with a diagnosis of genital endometriosis was performed. They were treated as inpatients at the gynecological department of the Zaporizhia Regional Clinical Hospital.

**Results:** The average age of the patients was 37.3 ± 9.75 years. The most frequent complaints of patients at diagnosis were – dysmenorrhea with pelvic pain during menstruation – 44 patients (46.31%), non-menstrual pelvic pain – 74 (77.89%), profuse menstruation (>80 ml/cycle) – 28 (29.47%), intermenstrual bleeding – 22 (23.15%), irregular menstrual cycles – 12(12.63%) and increased duration of menstruation (>8 days) – 5(6.75%). The total duration of the pain at the time of hospitalization was: < 7 days – 12 (16.21%), 8–14 days – 4 (5.4%), 15–30 days – 10 (13.5%), 1–6 months – 28 (37.85%), 7–12 months – 9 (12.25%) and 1–5 years – 11 (14.85%). According to the guidelines, surgery with a laparoscopic approach and hormonal therapy were prescribed to all the patients of reproductive age. While 67.28% patients were treated with 2 mg dienogest, 32.72% patients were treated with a combination of 0.03 mg ethinylestradiol and 2 mg dienogest. All the patients who received dienogest did not report any active complaints.

**Conclusion:** Although other symptoms are observed in patients with genital endometriosis, pain is the most common debilitating factor. The use of a combination of surgical and hormonal approaches is required to treat this ailment which can help improve the overall quality of life in these patients.

**PS128 The implications of antinuclear antibodies in systemic lupus erythematosus**

Ioana-Irina Rezus¸^1^, Celina Luca^1^, Andreea Ciolacu^1^, Albert George Nechifor^1^, Tudor S¸tefan Nicuriuc^1^, S¸tefana Luca^1^, Elena Rezus¸^1,2^

^1^*Grigore T. Popa University of Medicine and Pharmacy, Iasi, Romania,*^2^*Clinical Rehabilitation Hospital Iasi, Romania E-mail address: ioanairinarezus@yahoo.co.uk (Ioana-Irina Rezus)*

**Introduction:** Systemic lupus erythematosus (SLE) is an autoimmune disease of unknown etiology characterized by certain criteria and versatile laboratory characteristics being present in different combinations and expressions, varying from one patient to the other in evolution and prognosis.

**Aim:** The aim of this work is to diagnose the incidence of clinical manifestations and laboratory assessment that exhibit the severity of the Lupus disease and establish the existing relationships between them.

**Methods:** Our study includes 30 patients, 24 women and 6 men,between 20–60 years, hospitalized in the Clinic of Rheumatology, Medical Rehabilitation Hospital of Iasi in the period of October 2017 until April 2018. The patients have been diagnosed with SLE based on the criteria of the American College of Rheumatology. They were followed for the various aspects of clinical manifestations in SLE. The most significant observed were arthritis or arthralgia (84%), cutaneous manifestations (63%) and general symptoms such as asthenia, fever, Raynaud‘s phenomen, anorexia, weight loss (56%).

The immunological data we colected consisted of screening tests for the specific serological markers of SLE -different types of Antinuclear Antibodies (Anti-ds DNA Ab, Anti-Sm Ab) and also for some less specific markers (Anti-Ro(SSA) Ab, Anti-LA(SSB) Ab, Antiphospholipid Ab). In order to do this we used the immunofluorescence test and the enzyme-linked immunoassay (ELISA).

**Results:** Anti-ds DNA antibodies were detected in 67% of the patients, Anti-Sm antibodies in 29%, Anti-RO(SSA) and Anti-LA(SSB) antibodies in nearly 40% and Antiphospholipid antibodies in 47% of them.

**Conclusion:** Taking into account the clinical manifestations and the values of the named antibodies, we could associate the Anti-ds DNA antibodies with renal manifestations, the Anti-Sm antibodies with serositis and the Antiphospholipid antibodies with hemolytic anemia, the Anti-Ro(SSA) and Anti LA(SSB) antibodies being already connected with photosensitivity, and the late SLE onset, respectively in literature.

**PS132 Treatment of pericarditis in childhood**

Staša Krasic’^1^, Sergej Prijic’^1^, Saša Popovic’^1^, Vladislav Vukomanovic’^1^

^1^*School of Medicine, University of Belgrade, Belgrade, Serbia E-mail address: stasakrasic5@gmail.com (Stasa Krasic)*

**Introduction:** Recurrent pericarditis is the most important complication of an acute form of the disease. There is no unique treatment protocols for pediatrics patients with recurrent pericarditis.

**Aim:** The aim of the study was to analyze influence of acute pericarditis and first relapse treatment on the average number of relapses occurred.

**Methods:** Study was retrospective, and data were collected from medical records of patients diagnosed and treated from January 2011 to December 2017 at the tertiary referent heart center.

**Results:** Our study included 63 patients with average age 12.1 ± 3.4 years. Non steroid anti inflammatory drugs (NSAID) were used in 31/63 patients and 32/63 received combination of corticosteroides (CS) and NSAID. Recidives of pericarditis were registered in 12/63 patients, including 4/31 (12.9%) treated with NSAID and 8/32 (25%) treated with combination of CS and NSAID during acute disease. Total number of relapses in non steroid group was 0 (IQR: 0–0), and 0 (0–0.75) in CS group (p = 0.14). Additionally, in patients with recidivism total number of relapses was bigger in CS group (2, IQR: 1.25–5.75) than in NSAID group (1, IQR: 1–1) (p = 0.028). In 6/12 patients, first recidivism was treated with NSAID and colchicine, while treatment of the other 6/12 patients included combination with CS (CS + NSAID or CS + NSAID + colchicine). After first recidivism, there was trend toward for a higher number of relapses in CS group (1.5, IQR: 0–9.25) than in NSAID + colchicine group (0, IQR: 0–1) (p = 0.121). Also, a lower number of relapses was registered in colchicine treated patients (0.5, IQR: 0–2.25) than in group without colchicine (1, IQR: 0–1).

**Conclusion:** Non steroid anti inflammatory drugs and colhicine, as treatment for acute pericarditis and first recurrence of the disease, reduce the number of relapses. Therefore, combination with corticosteroids might not be recommended for treatment of the pericarditis in childhood.

**PS143 Blistering skin diseases: Santaros Clinics’ dermatovenereology centre 10 years experience**

Julija Mironova^1^, Gabrielë Adamonytë^1^, Jüratë Grigaitienë^1^, Jurgina Üselienë^1^

^1^*Faculty of Medicine, Vilnius University, Lithuania E-mail address: Gabriele.Adamonyte@gmail.com (Gabrielë Adamonytë)*

**Introduction:** Autoimmune blistering skin diseases are a heterogeneous group of disorders associated with autoantibodies that are directed against skin structural proteins. Knowledge of the clinical presentation of these disorders and of the relevant diagnostic procedures is important not just for dermatologists, but also for a large scope of other healthcare professionals.

**Aim:** To figure out the connection between blistering skin diseases clinical presentation and other chronic illnesses, laboratory tests results, patient age and gender.

**Methods:** A retrospective medical documentation analysis of the patients who were treated in Santaros Clinics’ Dermatovenereology Centre in 2007–2017. Statistical analysis was performed using SPSS.

**Results:** There were 135 patients hospitalized due to blistering skin disorders in 2007–2017: 36,6% men (n = 49), mean age 73,517,6 y., 63,4% women (n = 85), mean age 7615,1. 72,4% (n = 97) were from the city, whereas 27,6% (n = 37) were from the country. Mean age in the city (76,714,3 m.) was higher than in the country (71 19,4 m.). The most common disease in both city and country was bullous pemphigoid (respectively 64,9% and 43,2%). Mean hospitalization period was 10,987,9d., mean disease duration was 34,771,8 months. 67.9% of patients had blisters (n = 91), 76.1% - erosions (n = 102), 41% - erythema (n = 55), 14.9% - papules (n = 20), 24.6% - papules and vesicles (n = 33). 51.5% suffered itching (n = 69), 9% - dysphagia (n = 12), 3% - dysuria (n = 4), 3.7% experienced weight loss (n = 5). In bullous pemphigoid, CRP level elevated on average to 16,525.1 (p < 0.05), in pemphigus vulgaris - 8.316.2 (p < 0.05). Older patients experienced weight loss more frequently (p < 0.05). Histological examination was carried out for 93.3% of patients (n = 125), IF - 28.4%. (n = 38), cytological examination - 9%. (n = 12). For a systemic treatment the most common was Prednisolone - 71.6% (n = 96), for topical - Clobetazole cream - 21.6% (n = 29). 97,8% did not experience treatment effects (n = 131), 0.7% experienced hypertensive crisis (n = 1), 0.7% - thrombosis (n = 1), 0,7% - bleeding from GIT (n = 1).

**Conclusion:** We found that women tend to develop blistering skin diseases more often. CRP level elevation was observed in bullous pemphigoid and pemphigus vulgaris. Older age may be linked with weight loss. Generally, treatment side effects are rare.

**PS151 Impact of mild renal impairment and arterial stiffening to left ventricular concentric hypertrophy in moderate degenerative aortic stenosis**

Klaudiusz Bolt^1^, Dorota Długosz^1^, Wai Seng Sam^1^, Tomasz Nawara^1^, Bernadeta Chyrchel^1^, Olga Kruszelnicka^1^, Andrzej Surdacki^1^

^1^*Jagiellonian University Medical College E-mail address: klaudiusz.bolt@gmail.com (Klaudiusz Stefan Bolt)*

**Introduction:** Degenerative aortic stenosis (AS) is a progressive disease and left ventricular (LV) hypertrophy develops gradually already in moderate AS, an antecedent of severe AS. LV remodeling and symptomatic status in moderate AS may be affected by non-valvular factors, e.g. hypertension, CAD and renal function.

**Aim:** To assess effects of renal function on characteristics of real-world patients with moderate AS.

**Methods:** We reviewed hospital records of 150 subjects with moderate AS, out of whom 70 with pure AS in sinus rhythm, with EF > 40% and stable in-hospital creatinine entered the final analysis. The patients (age: 76 ± 9 years) were compared according to GFR [ml/min per 1.73 m2]: group A (GFR > 85), B (GFR = 60 − 85) and C (GFR = 15 − 59). We also calculated systemic arterial compliance (SAC) and valvulo-arterial impedance (Zva), an index of total LV afterload, from echocardiographic records and blood pressure.

**Results:** The 3 groups did not differ in aortic valve area, EF, LV mass, prevalence of symptoms or CAD. Compared to group A, group B subjects were older (p = 0.002), had lower SAC (p = 0.004) and higher pulse pressure (p = 0.004), relative LV wall thickness (RWT) (p = 0.04) and LV mass/volume ratio (p = 0.03). GFR correlated to SAC (r = 0.48, p = 0.002), pulse pressure (r =  − 0.38, p = 0.02), RWT (r =  − 0.46, p = 0.001) and Zva (r =  − 0.34, p = 0.04) only in groups A and B.

**Conclusion:** Mild renal impairment appears associated with LV concentricity in moderate AS irrespective of valve area. This relation can be mediated by concomitant arterial stiffening that increases LV afterload. Thus, prevention of early GFR decline may possibly delay development of LV diastolic dysfunction and symptoms in AS.

**PS152 Anatomical variants of pulmonary veins in patients with atrial fibrilation**

Albert George Nechifor^1^, Andreea Ciolacu^1^, Celina Luca^1^, Ioana Irina Rezus¸^1^, Grigore Tinică^1,2^, Cristina Luca^1,2^

^1^*Univeristy of Medicine and Pharmacy “Gr. T. Popa” Iasi,*^2^*Institute for Cardiovascular Diseases “Prof. Dr. George I. M. Georgescu” Iasi E-mail address: nechifor_aby@yahoo.com (Albert George Nechifor)*

**Introduction:** Multidetector computed tomography (MDCT) is often performed to assess cardiac structure and morphology. It is used in the planning of complex invasive procedures, such as pulmonary veins (PVs) isolation in patients with atrial fibrillation (AF). MDCT has an important role in identifying the pulmonary veins and branches, measuring their dimensions, and creating a structural map of the left atrium (LA).

**Aim:** LA and PVs morphology and dimensions are very important parameters in the ablation technique in order to avoid the complications that could appear during or after this procedure. The aim of the present study is to evaluate the prevalence of PVs anatomical variants in patients with AF compared to patients with sinus rhythm (SR).

**Methods:** We evaluated the venous drainage of LA in two groups of patients: a group of 50 patients with AF and a group of 50 patients with SR in order to find a possible relation between anatomical variants of PVs and AF, by using MDCT.

**Results:** For the right side, the presence of a separate ostium for the middle pulmonary vein was the most frequent variant in both groups of patients, with no significant difference between the groups. Accessory pulmonary veins were present mostly in the AF group, more frequently on the right side compared to the left one.

For the left side, a common left venous trunk was significantly higher present in patients with AF (32%) compared to patients in SR (12%).

**Conclusion:** A left venous pulmonary trunk and the accessory pulmonary veins are present more frequently in patients with AF and they could be anatomical factors associated with the development of AF. MDCT can accurately assess anatomical and morphological details as well as the potential individual features of the PVs and LA that are in relation with AF development.

**PS163 Contribution of vitamin D deficiency to arterial rigidity as assessed by Pulse Wave Velocity in angiographically proven Coronary Artery Disease**

Mahima Gupta^1^, Navin Mallya^1^, Poornima Manjrekar^2^, Padmanabha Kamath^3^, Harsha Ghanti^2^

^1^*Kasturba Medical College, Mangalore, India,*^2^*Department of Biochemistry, Kasturba Medical College, Mangalore, India,*^3^*Department of Cardiology, Kasturba Medical College, Mangalore, India, E-mail address: malu251097@gmail.com (Mahima Gupta)*

**Introduction:** Arterial stiffness is an important reflection of degeneration of the arterial wall. Emerging evidence suggests that arterial stiffness measured by Pulse Wave Velocity (PWV) is one of the earliest detectable signs of functional and structural changes in the arterial wall[1]. Moreover pressure wave travelling through the wall of an artery is influenced by the biological and mechanical properties of that blood vessel which is indirectly regulated by Vitamin D. Inflammatory changes, osteogenesis and bone-forming mechanisms, angiogenesis, andendothelial dysfunction are important issues associated with the pathogenesis of atherosclerosis, which are affected by vitamin D[2].Thus the role of Vitamin D as a predisposing factor to cardiovascular diseases is a subject of interest[3].

**Aim:** The aim of this study was to evaluate the Vitamin D and PWV in Coronary Artery Disease(CAD) patients and to correlate the findings with other cardiac parameters.

**Methods:** This case control study included 35 subjects with angiographic evidence of CAD and 27 apparently healthy individuals. All subjects underwent analysis for Vitamin D, PWV, heart rate, Cardiac output, Augmentation Pressure, Mean Arterial Pressure(MAP), Coronary Perfusion Pressure(CPP). Data was analysed using SPSS (Statistical Package for the Social Sciences) version 17. Pearsons Correlation was used for analysis and p value < 0.05 was considered significant.

**Results:** Patients with CAD had significantly lower Vitamin D levels compared to the control group (9.9 ± 6.9 vs. 37.3 ± 17 ng/ml, p = 0.00). PWV (9.2 ± 1.5 vs. 7.8 ± 1.7 m/s, p =  < 0.002) was significantly higher in CADs patients compared to healthy volunteers. Moderate correlation was found between PWV and MAP (r = 0.47, p = 0.004), CPP(r = 0.491, p = 0.003) and Augmentation Pressure (r = 0.441, p = 0.008).

**Conclusion:** Our study showed that CAD patients had higher PVW and lower Vitamin D levels. Thus arterial stiffness assessment can be considered a reliable tool to improve detection and risk stratification of patients with CAD.

**References**

1. Ranjith R, Binu T, George V, Madhu K, Devika P, Subair K et al. Aortic pulse wave velocity and its relationship with complexity of coronary artery disease based on SYNTAX score. Heart Asia. 2014;6(1):109-115.

2. Praveček M, Vuković-Arar Ž, Miškić B, Hadžibegović I. Vitamin D Deficiency in Acute Coronary Syndrome – Clinically Relevant or Incidental Finding?. Central European Journal of Public Health. 2017;61(3):185-190.

3. Effect of Vitamin D Supplementation on Arterial Stiffness and Central Blood Pressure Indexes: Demystifying the Evidence. Journal of the American Heart Association. 2017;6(10):e007466.

**PS173 The neutrophil-to-lymphocyte ratio – a predictor of severe coronary lesions in patients with stable coronary artery disease**

Balan Elena-Sabina^1^, Dobranici Mihaela^1^, Delcea Caterina^1^, Mihai Emilian^1^, Gheorghe-Andrei Dan^1^

^1^*Carol Davila’ University of Medicine and Pharmacy, Bucharest, România E-mail address: sabina.balan05@gmail.com (Balan Elena-Sabina)*

**Introduction:** The neutrophil-to-lymphocyte ratio (NLR) is a marker of inflammation that was recently correlated with severity and prognosis in acute coronary syndromes.

**Aim:** Our objective is to assess its correlation with the presence and severity of coronary artery lesions in patients with stable coronary artery disease (CAD).

**Methods:** We included consecutive patients with stable CAD admitted to our Cardiology department from September 2014 to May 2018 that underwent coronary angiography. Patients with an acute coronary syndrome during the last month and those with an active infection were excluded.

**Results:** Our sample consisted of 113 patients.50.44% were female. Mean age was 65.1 ± 8.34 years. Mean NLR was 2.66 ± 1.17.41.59% had significant coronary lesions, 12.39% had three-vessel coronary lesions and 26.54% had a SYNTAX score > 22.

NLR was higher in patients with significant coronary lesions compared to patients without significant lesions (2.96 ± 1.17 versus 2.48 ± 1.13, p = 0.02), in patients with three-vessel disease compared to those without (3.25 ± 1.46 versus 2.60 ± 1.11, p = 0.05) and in patients with a SYNTAX score higher than 22 compared to those with a lower score (3.13 ± 1.38 versus 2.58 ± 1.11, p = 0.07).

NLR was correlated neither to patients’ age nor to the patients’ gender.

In ROC curve analysis, NLR predicted the presence of significant coronary lesions with an AUC of 0.652 (95%CI 0.557 – 0.738, p = 0.003) with a cut-off of > 2.32 calculated with theYoudenindex associated criterion, 72.34% sensitivity and 61.19% specificity.

Patients with a NLR > 2.32 had an odds ratio of having significant coronary lesions of 3.87 (95%CI 1.73 – 8.65), p = 0.001.

**Conclusion:** NLR is an easily accessible, cost-efficient biomarker with a significant predictive value for the presence and severity of coronary lesions in stable CAD. We therefore propose the use of NLR as an additional severity marker in stable CAD.

**PS174 Health effects of a prolonged treatment of patients with the upper gastrointestinal tract diseases**

Barbara Habrat^1^, Magdalena Achtenberg^1^, Dorota Cibor^1^

^1^*Jagiellonian University Medical College E-mail address: 94habratbarbara@gmail.com (Barbara Anna Habrat)*

**Introduction:** Proton-pump inhibitors (PPIs) are a group of the most prescribed drugs in pharmacological treatment of upper gastrointestinal disorders. Therefore, safety of these drugs and their impact on patients’ health should be thoroughly examined.

**Aim:** The aim of our study was to evaluate the dosing, indication and type of inhibitor, as well as the incidence of adverse effects associated with chronic proton pump inhibitor (PPI). We also wanted to compare laboratory tests results and other clinical data of patients with a long-term PPIs use with results of patients not undergoing such therapy.

**Methods:** Study design was a case-control study evaluating patients with gastroesophageal disorders who were prescribed a PPIs for at least 1 year (PPI group) and patients who had never been prescribed a PPIs (control group) during 5 consecutive years.

**Results:** 687 adults were included: 344 patients in the PPI group (group 1) and 343 patients in the control group (group 2). The main type of inhibitor which was taken by over a half of the patients (51,7%) was pantoprazole. 63% of cases were patients with gastritis and duodenitis, functional dyspepsia or GERD. With regard to the test results, the decreased albumin serum level (<35 mg/ml) was reported in 20% of the PPI group compared to 33% in the control group (p = 0,01305). 8% of patients in group 1 had a reduced magnesium serum level (<0,66mmol/l) while significantly more (30%) in group 2 (p = 0,02918). There were no differences between groups if we take the calcium, vitamin B12 and vitamin D3 serum level into account. The median of ferritin concentration decreased with the increase in the dose of PPI used, but it was within the normal range.

**Conclusion:** Summing up, our research shows that there is no negative impact of a long-term PPIs therapy on results of laboratory tests and other clinical data.

**PS177 Analysis of waveform flow through carotid artery in younger and older respondents**

Aleksandra Ljubojevic’^1^, Zorica Nestorovic’^2^

^1^*Faculty of Medicine, University of Belgrade,*^2^*Institute of Biophysics in Medicine, Faculty of Medicine, University of Belgrade E-mail address: ljubojevicaleksandra@gmail.com (Aleksandra Ljubojevic*’*)*

**Introduction:** In organic systems such as cardiovascular there is the presence of short-term and long-term correlations that can give information about the condition of the cardiovascular system.

**Aim:** Nonlinear analysis examines whether there are differences in time correlations between younger and older respondents.

**Methods:** The carotid artery blood flow was measured by using the optical sensor in younger and older healthy respondents.

**Results:** The values of the skaling exponents α1 and α2 for each respondent were calculated by using DFA (Detrended Fluctuation Analysis) of the modified recorded blood flow signal. The ratio of α1 and α2 in young subjects is less than 1, and in older subjects it is greater than 1.

**Conclusion:** Comparison of the results showed that there are statistical differences in the values of the α1 and α2 exponents between the groups of respondents. This method can be used to monitor changes in carotid arteries during aging.

**PS182 Serum 25-hydroxyvitamin D levels and diabetic retinopathy in Type 1 diabetes patients – an association?**

Miguel Lopes^1^, Rita Laiginhas^2,3^, Carolina Madeira^4^, Margarida Barbosa^5^, Davide Carvalho^6,7^, Fernando Falcão-Reis^4,8^, Manuel Falcão^4,8^

^1^*Faculty of Medicine of Porto University,*^2^*Department of Ophthalmology, CHEDV,*^3^*PDICSS, Faculty of Medicine of Porto University,*^4^*Department of Ophthalmology, Centro Hospitalar de São João,*^5^*Department of Anesthesiology, Centro Hospitalar de São João,*^6^*Department of Medicine, Faculty of Medicine of Porto University,*^*7*^*Department of Endocrinology, Diabetes and Metabolism, Centro Hospitalar de São João,*^8^*Department of Surgery and Physiology, Faculty of Medicine of Porto University E-mail address: miguelopes96@hotmail.com (Miguel Carvalhais Valente Campos Lopes)*

**Introduction:** Diabetic retinopathy (DR) remains one of the leading causes of blindness worldwide. Besides its role in calcium homeostasis, Vitamin D has important immunomodulatory properties, playing a part in the regulation of angiogenesis. However, its role in autoimmune diseases, such as type 1 diabetes mellitus (DM1) and its microvascular complications, remains under discussion. Some experimental and clinical studies suggest a possible association between vitamin D deficiency and DR in type 2 diabetic patients. However, due to its lower prevalence, there are few studies on DM1 regarding this topic.

**Aim:** To assess the hypothesis that lower serum 25-hydroxyvitamin D levels are associated with prevalent DR in DM1 patients.

**Methods:** Retrospective review of a population of DM1 patients followed in a tertiary center from Portugal (Centro Hospitalar de São João). Patients were included if they had an available ophthalmological evaluation and a serum 25-hydroxyvitamin D level determination within the same year. DR status was classified based on fundoscopy findings in medical records. Binary logistic regression analysis was used for adjustments.

**Results:** We included 278 patients (51% male). The mean duration of DM1 was 20 ± 13 years, and 55% had DR. The mean level of 25-hydroxyvitamin D was not superior in patients without DR (mean level in DR patients = 20.4 ± 11ng/mL, mean level in non-DR patients = 22.6 ± 11ng/mL, p = 0.17). However, after adjusting for the duration of DM1, we found a significant association between 25-hydroxyvitamin D levels and prevalent DR (OR = 0.958, CI95% 0.92–0.99, p = 0.02). The interaction between the duration of disease and 25-hydroxyvitamin D serum levels was not significant (p = 0.78).

**Conclusion:** In our study, lower levels of 25-hydroxyvitamin D were associated with prevalent DR in DM1 patients, after adjusting for the duration of the disease. Future studies may elucidate the biological mechanisms between this association.

**PS186 Paracetamol poisoning - assessment of clinical symptoms and variations in the clinical profiles of patients**

Karol Mystek^1^, Monika Orłowska^2^, Iwona Popiołek^3^, Grzegorz Pore¸bski^4^

^1^*Department of Toxicology and Environmental Diseases, Jagiellonian University Medical College,*^2^*Jagiellonian University Medical College,*^3^*Department of Toxicology, University Hospital in Krakow,*^4^*Department of Allergology, Jagiellonian University E-mail address: karol.mystek@gmail.com (Karol Mystek)*

**Introduction:** Paracetamol is one of the most extensively used analgesic worldwide, being a component of hundreds over-the-counter and prescription medications which overdose, intentional or accidental, is relatively common.

**Aim:** We performed a retrospective study to analyze clinical symptoms of paracetamol poisoning and characterize variations in the clinical profiles.

**Methods:** In this retrospective study we analyzed data from the files and registers of Department of Toxicology of University Hospital in Kraków, between 2012 and 2018. A number of all 174 patients admitted to Hospital with diagnosis od paracetamol poisoning, based on potentially hepatotoxic paracetamol concentration in blood test performed upon admission, was included.

**Results:** The average dose of paracetamol taken by patients was 22530 mg. The manifestations reported included nausea, abdominal pain and impaired consciousness. The average values of the highest AST and ALT levels, during the period of hospitalization, were respectively 706 and 702, while the median of the highest INR levels amounted to 1,28. 44 patients reached hepatotoxic (>1000 IU) levels of ALT while only 18 of them developed hepatic lesions and 3 required liver transplantation. 75 patients used other substances together with paracetamol, 54 of them took other NSAIDs and 49 had positive alcohol testing. Among the coexisting diseases the most frequent were psychiatric disorders.

**Conclusion:** As the paracetamol poisoning is one of the most frequent among all types of drug intoxications, the knowledge of probable medical background, potential manifestations and prognosis is crucial in clinical practice. The performed study suggests that medical evaluation should be performed together with the mental health appraisal assessed by either psychiatrist or clinical psychologist.

**PS204 Dementia in heart failure – the risk factors and its impact on compliance**

Patrycja Furczyn'ska^1^, Konrad Ste¸pien’^1^, Karol Nowak^1^, Izabella Owsianka^1^, Aleksandra Włodarczyk^1^

^1^*Student's Scientific Group, Department of Coronary Artery Disease and Heart Failure, Jagiellonian University Medical College E-mail address: f.patrycja@wp.pl (Patrycja Furczyn*'*ska)*

**Introduction:** In the last few years studies pointing to an interdisciplinary relationship between heart failure (HF) and Alzheimer's disease were published. The authors unambiguously present HF as new and potentially modifiable risk factor of dementia. Because of recent identification and complexity of the problem, since now there has been no answers to a lot of questions concerning this topic.

**Aim:** The aim of the study was to assess etiological factors of dementia and its influence on compliance.

**Methods:** 490 patients hospitalized in the period of 2008–2017 because of exacerbation of chronic HF were enrolled. Among all alive, 142 (61,47%) agreed to take part in the study (94 – 66,20% men). The multivarious telephone assessment with the use of validated Bratzel-MMSE scale (dementia <  = 16 points) and MACCE questionnaire was performed. The occurrence of stroke was verified by the QVSFS questionnaire and information about smoking was collected. In order to assess compliance, Morisky questionnaire was used.

**Results:** In the study dementia (D) has been recognized among 30 people (21,13%). The D patients were older (78,53 ± 7,90 vs 75,26 ± 6,57 years), more often suffered from renal failure (46,67% vs 20,54%; p = 0,004) and peripheral arterial disease (PAD) (43,33% vs 16,07%; p = 0,001) than non-dementia (ND) patients. The QVSFS questionnaire showed that D patients were more often diagnosed with stroke (46,67% vs 20,54%; p = 0,004) and more often declared smoking (56,67% vs 35,71%; p = 0,038) which was connected with more pack-years (19,33 ± 20,42 vs 12,9 ± 21,50; p = 0,047). The D patients were also more often re-hospitalized because of exacerbation of HF (53,33% vs 28,57%; p = 0,017). Among both groups there were no further significant differences.

**Conclusion:** According to the results of our study, older age, renal failure, PAD, smoking and re-hospitalizations because of exacerbation of HF may be recognized as potential risk factors of dementia. Differences concerning compliance between both groups were not observed.

**PS209 Athlete's heart phenomenon in a young male population – cross-sectional study**

Sebastian Janiec^1^, Jeremiasz Kubisiowski^1^, Andrzej Nowak^1^, Dawid Bugara^1^

^1^*Students’ Scientific Group at Ist Department of Cardiology and Interventional Electrocardiology and Hypertension JUMC E-mail address: sjaniec@onet.eu (Sebastian Janiec)*

**Introduction:** In recent years, the number of young people regularly practicing sport has grown significantly in connection with their desire to achieve ideal physique. Prolonged excessive physical activity may be associated with the development of the athlete's heart phenomenon. Its long-term consequences are still controversial and debatable.

**Aim:** The aim of our study was to assess the prevalence of hemodynamic variability and disorders in the group of young, physically active men and to identify the factors determining them.

**Methods:** We invited 50 healthy men (age- 21–30 years), practicing sport at least twice a week to take part in our study. In all subjects: anthropometric measurements, bioelectrical impedance analysis (Bodystat analyzer), peripheral (Omron) and central blood pressure (BP) measurements were taken, with short-term heart rate variability (HRV) (Sphygmocor), ECG analysis and echocardiography performed. All the above mentioned examinations were repeated after one week.

**Results:** Basing on the two blood pressure measurements, 5(10%) participants were diagnosed with isolated systolic hypertension and 3(6%) with systolic-diastolic hypertension. Participants with established hypertension were characterized by higher pulse pressure (42.87 vs 33.29 mmHg, p = 0.006), lower LF/HF ratio (0.88 vs 1.57, p = 0.001) and were taller (185.37 vs 180.67 cm, p = 0.03) than normotensives.

In 6(12%) persons a slight bradycardia (<60 BPM) was reported. In the echocardiographic study, 4(8%) participants were characterized with significant left ventricular enlargement, and as many as 9(18%) persons with abnormal left atrial volume. Resting heart rate correlated negatively with the left ventricular end diastolic dimension (r = -0.33, p = 0.019) and the left atrium volume (r = -0.37, p = 0.007). The only factor determining the left ventricular mass was pulse pressure (r = 0.38, p = 0.007).

**Conclusion:** Hypertension is a common hemodynamic disorder observed in young athletes. Left atrial volume correlates negatively with resting heart rate, what may be related with a long-term risk of atrial fibrillation in athletes.

**PS211 The phenomenon of postprandial hypotension in young people – prevalence and determining factors**

Jeremiasz Kubisiowski^1^, Sebastian Janiec^1^, Dawid Bugara^1^, Agnieszka Ste¸pien’^1^, Anna Płotek^1^, Andrzej Nowak^1^

^1^*Students’ Scientific Group at Ist Department of Cardiology and Interventional Cardiology and Hypertension JUMC E-mail address: jeremiasz.kub@gmail.com (Jeremiasz Kubisiowski)*

**Introduction:** Decrease in blood pressure (BP) after a meal is a well-known hemodynamic phenomenon. In the elderly postprandial hypotension may enhance the risk of syncope and general mortality.

**Aim:** Our study aimed to assess the physiological changes in peripheral and central blood pressure (BP) values after ingesting a 75 g glucose solution occurring in the group of young adults and to identify factors determining them.

**Methods:** 52 subjects (men = 26) aged 20–25 participated in the study. In all of them: measurements of peripheral blood pressure (Omron), assessment of central blood pressure with the analysis of short-term heart rate variability (HRV) (Sphygmocor) and determination of the blood glucose level in the capillary blood (Contour Plus) were performed. The subjects were then given 300 ml of an aqueous solution of 75 g of glucose. All of the above mentioned tests were repeated after 60 and 120 min. from the glucose load.

**Results:** After glucose administration in the 60th minute of the test, we observed a significant decrease in systolic (D = -6.16 mmHg, p < 0.001), diastolic (D = -2.29 mmHg, p = 0.06), and central systolic BP (D = -5.37 mmHg, p < 0.001), as well as pulse pressure (D = -3.48 mmHg, p < 0.001). In multivariate analysis, reduction of systolic BP in 60th minute was determined by gender (b = -0.53, p < 0.001) and baseline systolic BP values (b = -0.65, p < 0.001). The factors determining the decrease in BP in the 60th minute of OGTT in men were: baseline BP (b = -0.44, p = 0.01), baseline glucose concentration (b = 0.39, p = 0.027) and baseline LF/HF ratio (b = 0.47, p = 0.01); while in female - only the baseline systolic BP value (b = -0.64, p = 0.004). Furthermore, baseline heart rate was the factor determining the decrease in systolic BP 120 min. after OGTT in women(b = 0.47, p = 0.022).

**Conclusion:** The glucose consumption resulted with a significant decrease in the systolic and diastolic BP values. Groups of women and men were characterized with different factors determining the decline in systolic BP.

**PS217 Anemia and Chron's disease**

Nedim Vanis^1^, Džan Horozic’^1^, Emir Sokolovic’^1^, Azra Husic’ Selimovic’^2^, Nenad Vanis^3^

^1^*Faculty of Medicine, University of Sarajevo,*^2^*Gastroenterology, Clinical Center University of Sarajevo,*^3^*Gastroenterology, Middle East Hospital, Bahrain E-mail address: nedim_08@yahoo.com (Nedim Vanis)*

**Introduction:** Crohn's disease is an inflammatory bowel disease (IBD) that causes transmural inflammation of the gastrointestinal tract. It is followed by abdominal pain, severe diarrhea, fatigue, weight loss, malnutrition, stool with or without gross bleeding and anemia. Although efficient therapeutic options have been developed for the treatment of CD associated anemia, treating anemia often has a low priority for gastroenterologist.

**Aim:** To evaluate prevalence of IBD-associated anemia in patients diagnosed for CD in the Department of Gastroenterology and Hepatology, Clinical Centre University of Sarajevo.

**Methods:** The study was conducted between March 2013 and March 2015 as a retrospective observational clinical trial. Total of 101 patients with histopathological and clinical verification of CD were included in the study. They were divided into four groups according to Crohn‘s Disease Activity Index (CDAI). Biochemical parameters were assessed: complete blood count (CBC), C-reactive protein (CRP), iron.

**Results:** In the present study patients (n = 29) with asymptomatic remission (CDAI < 149) had average hemoglobin serum level 14.3 g/dl while patients (n = 8) with severely active to fulminant disease (CDAI 451 to 1100) had average hemoglobin 7.3 g/dl (p < 0.001). In a group of patients (n = 43) with mildly to moderately active disease (CDAI 150 to 220) average hemoglobin was 11.8 g/dl, while in a group of patients (n = 21) with moderately to severely active disease (CDAI 221 to 450) was 10.7 (p < 0.05). Iron deficiency was diagnosed in 20.83% of the patients with CDAI < 220, compared with the patients with CDAI > 220 which was 82.75% (p < 0.001).

**Conclusion:** IBD-associated anemia is a unique example of the combination of chronic iron deficiency and anemia of chronic disease. Anemia, as a consequence of the Crohn's disease, should not be taken only as a diagnostic parameter, rather also as a complication of the disease, and therefor it deserves appropriate therapeutic approach.

**PS221 R2CHADS2 - a better predictor than CHADS2 and CHA2DS2-VASc for long-term all-cause mortality in heart failure patients**

Laurian Mihai Niculescu^1^, Caterina Delcea^1,2^, Ionut Codrin Gheorghe Hogea^1^, Adrian Buzea^1,2^, Mihaela Dobranici^1,2^, Ancuta Vijan^2^, Alexandra Andrus^2^, Mihaela Ailenei^2^, Alexandra Moise^2^, Simona Angheluta^2^, Gheorghe Andrei Dan^1,2^

^1^*Carol Davila’ University of Medicine and Pharmacy,*^2^*Colentina Clinical Hospital E-mail address: niculescu.laurian@gmail.com (Laurian-Mihai Niculescu)*

**Introduction:** The CHADS2, CHA2DS2-VASc and R2CHADS2 scores are well-known predictors of stroke in patients with atrial fibrillation. Due to the inclusion of major cardio-vascular comorbidities, they could potentially be useful in stratifying all-cause mortality in patients with heart failure (HF).

**Aim:** Our objective is to assess the predictive value of these scores for all-cause long-term HF mortality.

**Methods:** We retrospectively included HF patients admitted from January 2012 to December 2014. Patients with in-hospital mortality, acute coronary syndromes, pulmonary embolisms, neoplasms and systemic infections were excluded. Survival was assessed on June 1st 2018.

**Results:** The study group consisted of 726 patients with HF, 53.72% women, with a mean age of 71.57 ± 10.44 years. Long-term (5 years) mortality was 41.31%.

In the entire sample, in ROC curve analysis, R2CHADS2 score predicted mortality with an AUC of 0.640 (95%CI 0.598–0.683), p < 0.001, CHA2DS2VASc with an AUC of 0.547 (95%CI 0.504–0.590), p = 0.036, and CHADS2 with an AUC of 0.547 (95%CI 0.540–0.626), p < 0.001.

Best correlation of mortality percentages to score variability was obtained for the R2CHADS2 score. Mortality increased with each point of the score from 28% in patients with a score of 1 or 2, 30% in those with a score of 3, 50% in those with a score of 5, 57.89% in those with a score of 6 and 51.85% in those with a score of 7 or 8, p for trend < 0.001.

Regardless of the presence of atrial fibrillation, the three scores remained predictors of long-term all-cause mortality.

**Conclusion:** The CHADS2, CHA2DS2-VASc and R2CHADS2 scores are viable predictors of long-term all-cause mortality in heart failure patients. Of the three scores, R2CHADS2 has the best prognostic accuracy.

**PS223 Does left ventricular aneurysm influence survival in patients with heart failure and a history of myocardial infarction?**

Ionut Codrin Gheorghe Hogea^1^, Caterina Delcea^1,2^, Laurian Mihai Niculescu^1^, Adrian Buzea^1,2^, Anamaria Tociu^2^, Elena Stoichitoiu^2^, Antonia Lefter^2^, Raluca Popescu^1,2^, Gheorghe Andrei Dan^1,2^

^1^*Carol Davila’ University of Medicine and Pharmacy,*^2^*Colentina Clinical Hospital E-mail address: hcionut@yahoo.com (Ionut-Codrin-Gheorghe Hogea)*

**Introduction:** Left ventricular aneurysm (LVA) is a complication of myocardial infarction occurring in 8–15% of patients, with unclear long-term prognostic significance.

**Aim:** Our objective is to assess the impact of a LVA on long-term all-cause mortality of patients with heart failure due to ischemic coronary disease.

**Methods:** Patients with heart failure with prior myocardial infarction admitted to our clinic from January 2011 to December 2014 were retrospectively enrolled in this study. Acute coronary syndromes, neoplasms, pulmonary embolisms and systemic infections were exclusion criteria. Survival status was assessed in June 2018. Mean follow-up was 5.5 years.

**Results:** Our sample consisted of 203 patients, 62.6% male with a mean age of 70.72 ± 10.6 years. 55 had LVA. All-cause long-term mortality was 47% with no significant difference between patients with (48.15%) and without LVA (45.77%), p = 0.89.

LVA did not increase the long-term all-cause mortality risk (RR 1.05, 95%CI 0.75–1.46, p = 0.89).

In ROC curve analysis, age (AUC 0.627 (95%CI 0.549–0.705), p = 0.002), NT-proBNP (AUC 0.723 (95%CI 0.648–0.798), p < 0.01) and left ventricular ejection fraction (LVEF) (AUC 0.646 (95%CI 0.565–0.727), p = 0.001) were predictors of long-term all-cause mortality in all patients.

Patients with LVA were younger (66.87 ± 11.42 versus 72.06 ± 9.99 years, p = 0.001), but had higher median (interquartile range) NT-proBNP levels (3320 (1341; 9346) versus 1386 (557; 4158) pg/ml, p = 0.004) and lower median LVEF (27% (20; 35) versus 45% (35; 50), p < 0.001).

In ROC curve analysis, in patients with LVA the predictors for all-cause mortality were age (AUC 0.675 (95%CI 0.527–0.823), p = 0.026) and NT-proBNP (AUC 0.703 (95%CI 0.531–0.875), p = 0.035).

**Conclusion:** Although in patients with heart failure and prior MI the presence of a left ventricular aneurysm is associated to higher NT-proBNP levels and lower ejection fraction, this complication is not associated with higher long-term all-cause mortality.

Age, NT-proBNP levels and LVEF are independent predictors of survival in these patients.

**PS241 Concomitant anti-platelet therapy of atrial fibrillation and Ischemic Heart Disease**

Mashkur Abdulhamid Isa^1^, Svitlana Hrechko^1^

^1^*Bukovinian State Medical University E-mail address: mashforreal@yahoo.com (Mashkur Abdulhamid Isa)*

**Introduction:** Coronary heart disease (CHD), like atrial fibrillation (AF), increases the risk of thromboembolic events. However, the mechanism of thrombus formation in CHD is significantly different from that in the left chambers of the heart with AF. Dual antiplatelet treatment (DAPT) is a base of therapy of patients following elective percutaneous coronary interventions or acute coronary syndrome (ACS), such as ST elevation myocardial infarction, non-ST elevation myocardial infarction and unstable angina.

**Aim:** The use of different groups of drugs such as antiaggregants and/or anticoagulants is required for the prevention of thromboembolic complications; the effects of which we aim to study

**Methods:** 72 patients with ASA and AF were observed for 3 months. In all cases anticoagulants were prescribed: warfarin in 21 patients while rivaroxaban in 18 cases. DABT with acetylsalicylic acid was used in 33 cases: warfarin and aspirin in 16 patients while rivaroxaban and aspirin in 17 patients. The target levels of INR were monitored when warfarin was administered

**Results:** Rivaroxaban showed superiority in comparison to warfarin with regards to stroke and systemic embolism, especially among patients without concomitant use of antiplatelet therapy (p < 0.05) and a reduction in this effect in patients with concomitant antiplatelet therapy (p > 0,05).The rates of major bleeding increased in elderly patients on concomitant antiplatelet therapy.

**Conclusion:** Thus, rivaroxaban in patients with non-valvular etiology and coronary artery disease is similar in effectiveness to warfarin. It significantly surpasses the safety of warfarin and is less likely to cause large bleeding or intracranial hemorrhages.In patients who require dual or triple therapy, low-dose rivaroxaban might be a good alternative to warfarin due to good efficacy and low bleeding complications. Hence, rivaroxaban, along with other oral anticoagulants, can be prescribed for prolonged therapy in patients with AF and CHD, especially in patients with a high risk of bleeding

**PS245 Clinical characteristics of patients with premature cardiovascular disease. Retrospective study**

Gabriela Stanisława Kuczyn'ska^1^, Agnieszka Pura^1^, Marta Wilk^1^, Katarzyna Wąchała^1^, Larysa Bielecka^1^, Klaudia Bielecka^1^

^1^*Jagiellonian University Medical College, Cracow, Poland E-mail address: gabi_ja@o2.pl (Gabriela Stanisława Kuczyn*'*ska)*

**Introduction:** Cardiovascular diseases (CVDs) are the first cause of death globally in the group of patients from 15 to 49 years old. In this context the control of cardiovascular risk factors is especially important, as it is estimated that up to 80% of CVDs may be prevented by modifying patient's behaviour. Due to unf avourable long-term prognosis in younger population suffering from CVDs, even more effort should be put into their risk assessment and control.

**Aim:** We aimed to examine acute coronary syndrome (ACS) risk factors in the population with premature CVDs and identify the differences in their occurrence depending on the sex of the patients.

**Methods:** Medical records from consecutive patients from Department of Cardiac and Vascular Diseases in John Paul II Hospital in Cracow were analyzed. The inclusion criteria were diagnosed CVDs and age under 55 for women and 45 for men. To the study included 58 women and 42 men. We analyzed occurrence of 14 ACS risk factors. The patients were divided into groups based on sex.

**Results:** All patients had two or more of analyzed risk factors and were qualified to the group with very high risk of fatal cardiovascular disease over a ten-year period. 87% of examined had accompanying diseases, 86,42% had thickened intima-media complex and 85,57 had LDL higher 1,8 mmol/l. 76% had body mass index higher or equal to 25 kg/m2 and 71% had arterial hypertension. Statistically significant difference between groups occurred in alcohol abuse (21% men, 2% women, p < 0.005).

**Conclusion:** All of examined patients had two or more risk factors and should be under multidisciplinary medical care. The most common risk factors are modifiable.More research is needed to analyze the impact of CVD risk factors on prognosis and the long-term effects of reducing their number.

**Acknowledgements**

Assoc. Prof. Maria Olszowska PhD

**PS253 Early postprandial glucagon release prevents reactive hypoglycemia after gastric bypass**

Carolina B. Lobato^1^, Sofia S. Pereira^1^, Marta Guimarães^1,2^, Bollette Hartmann^3,4^, Linda Hilsted^5^, Mário Nora^1,2^, Jens J. Holst^3,4^, Mariana P. Monteiro^1^

^1^*Endocrine, Cardiovascular & Metabolic Research, Department of Anatomy, Unit for Multidisciplinary Research in Biomedicine (UMIB), Institute of Biomedical Sciences Abel Salazar (ICBAS), University of Porto, Porto, Portugal,*^2^*Department of General Surgery of Hospital São Sebastião, Centro Hospitalar de Entre o Douro e Vouga, Santa Maria da Feira, Portugal,*^3^*Department of Biomedical Sciences, Faculty of Health and Medical Sciences, University of Copenhagen, Copenhagen, Denmark,*^4^*Novo Nordisk Foundation Center for Basic Metabolic Research, Faculty of Health and Medical Sciences, University of Copenhagen, Copenhagen, Denmark,*^5^*Department of Clinical Biochemistry, Rigshospitalet, University of Copenhagen, Copenhagen, Denmark E-mail address: ccbritolobato@gmail.com (Carolina Coimbra de Brito Lobato)*

**Introduction:** The exponential increase in obesity prevalence [1] led to parallel rise in the number of bariatric surgery procedures performed worldwide, as the most effective means of reversing obesity-associated metabolic disorders [2] and achieving sustained weight loss [3]. Post-bariatric hypoglycemia is a rare but emerging clinical condition with poorly characterized etiology that lacks diagnostic criteria or established clinical management guidelines [4].

**Aim:** To characterize the post-prandial intestinal hormone dynamics after Roux-en-Y Gastric Bypass (RYGB) associated with post-bariatric post-prandial hypoglycemia (PPH).

**Methods:** Weight stable, non-diabetic patients (n = 19) from our single center cohort of post-bariatric patients (N = 2780), were invited to undertake a mixed-meal tolerance test (MMTT) with a standardized liquid meal after an overnight fast. Blood sampling was performed at baseline and at timed intervals for assessment of glucose, insulin, C-peptide, glucagon, glucose-dependent insulinotropic polypeptide (GIP), glucagon-like peptide-1 (GLP-1) and peptide YY (PYY) levels.

**Results:** During the MMTT, PPH occurred in a large proportion of post-RYGB patients (42%) characterized by a glucose nadir below 55 mg/dL (Hypo; n = 8) vs equal or above 55 mg/dL (NoHypo; n = 11). No differences in anthropometric (BMI-Hypo = 27.94 ± 2.63, BMI-NoHypo = 28.08 ± 3.36, p = 0.9190) or glycemic parameters (%HbA1c-Hypo = 5.19 ± 0.31, %HbA1c-NoHypo = 5.43 ± 0.28, p = 0.1098) between were found between the groups.

On MMTT, a lower glucagon excursion at 15’, preceding insulin and C-peptide 45’ peaks and glucose nadir, predicted PPH (ROC curve AUC = 0.8636; p = 0.0083). Insulin and C-peptide levels were also higher in Hypo patients (C-peptide T-AUC: p = 0.0447). No other differences in hormone profiles were found between the groups.

**Conclusion:** In hypoglycemia, glucagon is a well-known insulin antagonist [5]. Meal-triggered glucagon secretion preceding insulin excursion and glucose lowering during the MMTT, seems an unexpected key in preventing PPH. Thus, MMTT elicited hormone profile in post-RYGB patients provides relevant insights into endocrine mechanism underlying PPH. Ultimately, this could provide a starting point towards targeted interventions for prevention and treatment of PPH.

**References**

1. Collaborators, G.B.D.O., et al., Health Effects of Overweight and Obesity in 195 Countries over 25 Years. N Engl J Med, 2017. 377(1): p. 13-27.

2. Pories, W.J., et al., Who would have thought it? An operation proves to be the most effective therapy for adult-onset diabetes mellitus. Ann Surg, 1995. 222(3): p. 339–50; discussion 350–2.

3. Cummings, D.E., J. Overduin, and K.E. Foster-Schubert, Gastric bypass for obesity: mechanisms of weight loss and diabetes resolution. J Clin Endocrinol Metab, 2004. 89(6): p. 2608-15.

4. Eisenberg, D., et al., ASMBS Position Statement on Postprandial Hyperinsulinemic Hypoglycemia after Bariatric Surgery. Surg Obes Relat Dis, 2017. 13(3): p. 371-378.

5. Röder, P.V., et al., Pancreatic regulation of glucose homeostasis. Exp Mol Med, 2016. 48(3): p. e219-.

**Acknowledgements**

Unit for Multidisciplinary Research in Biomedicine is funded by FCT (UID/Multi/00215/2013).

**PS255 Association between intradialytic blood oxygen saturation and intradialytic hypotension**

Olivia Cyran^1^, Joanna Włostowska^1^, Barbara Downar^1^, Martyna Koz’ma^1^

^1^*Medical University of Lodz E-mail address: olivia.cyran@gmail.com (Olivia Anna Cyran)*

**Introduction:** Intra-dialysis hypotension significantly influences the prognosis of chronically dialyzed patients. Using the pulse oximeter to detect hypotension earlier could prevent it.

**Aim:** The aim of the study was to evaluate association between intradialytic blood oxygen saturation and intradialytic hypotension and the use of a pulse oximeter to predict intra-dialysis hypotension.

**Methods:** In this observational study, 15 chronic hemodialysis (HD) patients, with vascular access by arteriovenous fistula (AVF) or central venous catheter (CVC), were enrolled. Continuous non-invasive blood SO2 was monitored by a pulse oximeter; blood pressure (every 15 min) was monitored by ambulatory blood pressure monitoring. Predictive power of hypotension was expressed by the area under curve (AUC) sensitivity and specificity based on intradialytic variations in SO2.

**Results:** We analyzed the decrease in blood oxygen saturation and the change in blood oxygen saturation. Based on these observations and the observation of hypotensive episodes during dialysis, we developed the possibility of predicting intradialytic hypotension. The predictive value of hypotension by means of changes in saturation proved to be more predictive (AUC = 0.59). The hypotensive prognosis only with SO2 decreases proved to be less efficient (AUC = 0.48).

**Conclusion:** A pulse oximeter can help predict intra-dialysis hypotension.

Neurosciences

**PS009 Are not all cannabinoids are created equally? Results from a preliminary study on the adult depressive-like phenotype induced by chronic exposure to HU-210 during adolescence**

Miguel Ferreira^1,2^, Francisco M. Mouro^1,2^, Ana M. Sebastião^1,2^

^1^*Instituto de Farmacologia e Neurociências, Faculdade de Medicina, Universidade de Lisboa, Portugal,*^2^*Instituto de Medicina Molecular João Lobo Antunes, Faculdade de Medicina, Universidade de Lisboa, Portugal E-mail address: jorge.ferreira@medicina.ulisboa.pt (Jorge Miguel Farinha Ferreira)*

**Introduction:** Cannabis is the most widely consumed illegal drug amongst adolescents, with the lasting consequences of its use being highly relevant. Animal studies have shown that chronic exposure to cannabinoid receptor (CBR) agonists induce deficits in both the cognitive (e.g., memory) and affective (e.g., depressive-like phenotypes) domains, that persist into adulthood, after cannabinoid exposure has ceased [1–5], suggesting a lasting impact of adolescent exposure to CBR agonists.

**Aim:** To replicate the findings of an adult depressive-like phenotype resulting from chronic cannabinoid exposure during adolescence, using a drug (HU-210) yet untested for this purpose.

**Methods:** Adolescent female Wistar rats were administered daily intraperitoneal injections for 15 days in an escalating dosing schedule (PND35–39:25 mg/kg; PND42–46:50 mg/kg; PND49–53:100 mg/kg or equivalent vehicle). Behavioral testing occurred after a 26-day washout and consisted of the Elevated Plus Maze (EPM), Open Field (OFT), Social Interaction (SIT), Forced Swimming (FST), Sucrose Preference (SPT) and Marble Burying (MBT) tests.

**Results:** HU-210 decreased weight gain during the administration period, but this effect did not persist into adulthood. There were no differences between groups in either the EPM, OFT, SIT or MBT. In the FST, HU-210-exposed animals showed diminished climbing time, but no differences in either swimming or immobility times. During the SPT HU-210 animals consumed less food than controls, but no differences were found for either sucrose preference or consumption.

**Conclusion:** The results differ considerably from those previously reported [1–5] – with most of the expected deficits not being present. While these discrepancies might result from differences in experimental protocol, it is also possible that HU-210 might be qualitatively different from other CBR agonists. If the latter proves true, not only would it highlight questions as to the comparability of studies using different CBR agonists, but would also raise the question of why this compound differs from other CBR agonists in its effects.

**References**

1. Bambico, F. R., Nguyen, N.-T., Katz, N. & Gobbi, G. Chronic exposure to cannabinoids during adolescence but not during adulthood impairs emotional behaviour and monoaminergic neurotransmission. Neurobiol. Dis. 37, 641–655 (2010).

2. O'Shea, M., Singh, M. E., McGregor, I. S. & Mallet, P. E. Chronic cannabinoid exposure produces lasting memory impairment and increased anxiety in adolescent but not adult rats. J. Psychopharmacol. (Oxf.) 18, 502–508 (2004).

3. Realini, N. et al. Chronic URB597 treatment at adulthood reverted most depressive-like symptoms induced by adolescent exposure to THC in female rats. Neuropharmacology 60, 235–243 (2011).

4. Rubino, T. et al. Chronic D9-tetrahydrocannabinol during adolescence provokes sex-dependent changes in the emotional profile in adult rats: behavioral and biochemical correlates. Neuropsychopharmacology 33, 2760–2771 (2008).

5. Schneider, M., Schömig, E. & Leweke, F. M. Acute and chronic cannabinoid treatment differentially affects recognition memory and social behavior in pubertal and adult rats. Addict. Biol. 13, 345–357 (2008).

**PS015 Microglial motility in Alzheimer's disease and after Amyloid beta 42 immunotherapy**

Bethany George^1^, Diana Franco-Bocanegra^1^, James Nicoll^1^, Delphine Boche^1^

^1^*Clinical Neurosciences, Clinical and Experimental Sciences, Faculty of Medicine, University of Southampton, Southampton UK E-mail address: beg1g15@soton.ac.uk (Bethany Ella George)*

**Introduction:** Microglia, the immune cells of the brain, are ramified cells whom the function is dependent on cell motility. Evidence supports microglial impairment in Alzheimer's disease (AD), possibly linked to Amyloid beta (Abeta) protein accumulation, a hallmark of AD. In Abeta-42 immunised patients, Abeta removal is partly due to phagocytic microglia, supporting the association between microglia and Abeta clearance. We hypothesize that microglial motility is affected in AD and restored after Abeta-42 immunotherapy.

**Aim:** 1. To investigate microglial motile-protein Iba1 and Coronin-1A in post-mortem study.

2. To explore microglial morphology associated with motility.

**Methods:** Microglial motility was explored in the inferior parietal lobe of control (n = 32), AD (n = 44) and immunised (i)AD (n = 16) cases. Immunohistochemistry was performed for microglial motility-associated actin-binding protein Coronin-1A. Slides were scanned and 30 images in the grey and in the white matter were extracted for analysis with ImageJ to obtain protein load. Previous data for Iba1, Cofilin (proteins interacting with cytoskeleton F-actin) and P2RY12 (potassium channel-related protein) were used for further analysis. Microglial morphology, defined as ramified, reactive or amoeboid, was assessed using Iba1 staining.

**Results:** No significant difference in Coronin-1A load was observed between the 3 cohorts. However, in the AD group only, significant correlations were observed in the grey matter between Coronin-1A with Iba1 (P = 0.436, p = 0.003), and Cofilin (P = 0.530, p < 0.001) but not with P2RY12, using Spearman's Rho test. Assessment of microglial morphology showed increased Iba1 + microglia in iAD (p = 0.011), mainly as ramified microglia (p = 0.004), using Kruskal-Wallis test.

**Conclusion:** Our data support a role for microglial motility in AD pathogenesis, specifically involving proteins interacting with the cytoskeleton. Moreover, we have evidence to suggest that Abeta-42 immunotherapy might be beneficial towards microglia motility. Indeed, the iAD cohort with increased ramified microglia is promising, as these are considered healthy microglia able to survey the brain parenchyma.

**PS030 Cellular organisation of geniculate ganglion**

Jovana Vidanovic’^1^

^1^*Institute of Histology and Embryology, Aleksandar Đ. Kostic*’*, University of Belgrade E-mail address: moijovana@me.com (Jovana Vidanovic)*

**Introduction:** The geniculate ganglion is a collection of fibers and sensory neurons of the facial nerve, which receives fibers from motor, sensory and parasympathetic components of the facial nerve.

**Aim:** We tried to determine cellular structure of the geniculate ganglion, corelation between glia cells, neurons and structure of extracellular matrix, as well as the immunoreactivity of the ganglia cells.

**Methods:** The sample is made of 20 isolated geniculate ganglions.We used Leica Interactive Measurements software for morphometric analysis. Hystological analysis methodes included: Masson and Picro-Mallory staining, Gordon-Sweet staining for reticular and elastic fibers with orcein. Immunohistochemistry techniques included Dako LSAB + /HRP.

**Results:** The largest number of ganglions has triangular form, typical position and moderate cellularity. Ganglion cells are organized in the form of clusters. The use of pan-neuro markers of NSE, S-100 proteins and Sy showed that they are expressed in 85–90% of ganglion cells. IHH techniques showed a high percentage of SP and CGRP, as well as colocalization of both neuropeptides in the same ganglion cells. The expressions of VIP, NPY and somatostatin, were not observed by the application of these techniques, neither the activity of Ach-esterase, tyrosine hydroxylase and glutathione synthetase. Mast cells are present in the loose connective tissue of the geniculate ganglion.

**Conclusion:** Our study showed that SP was expressed in 70% of GG cells and CGRP in 62% of cells. It was shown that there was no statistically significant distribution in the CGRP-IR. The most numerous were medium-sized cells. IHH did not show the expression of VIP, NPY or somatostatin in ganglia cells, neither AchE, glutamine synthetase or tyrosine hydroxylase. This shows that cells do not synthesize Ach, GABA, nor dopamine and NA. Mastocytes were found in most of the examined geniculate ganglions.

**PS051 Prrxl1 and Casz1 – new insights into the development of the nociceptive system**

Miguel Pereira Macedo^1,2^, César Monteiro^1^, Filipe Monteiro^1,2^, Deolinda Lima^1,2^, Sandra Rebelo^1,2^

^1^*FMUP,*^2^*I3S E-mail address: miguel_macedo2@hotmail.com (Miguel Pereira da Cunha Coelho de Macedo)*

**Introduction:** Neural progenitors alter their phenotype over time generating different types of neurons and glial cells in specific chronological sequences. Regarding the somatosensitive system, the dorsal root ganglion (DRG) and the spinal cord dorsal horn present as fundamental stations of signal transmission and processing. The correct assembly of the neural network involved is central for the correct perception of thermal, mechanical and nociceptive stimuli. Prxxl1 is a master gene in the development of excitatory superficial dorsal horn neurons. Casz1 was demonstrated to be under positive regulation by Prxxl1 in the spinal cord but not DRG.

**Aim:** Characterization of the spatial and temporal expression of Prxxl 1 and Casz1 transcription factors in the mouse and human embryos and establishment of possible developmental equivalents.

**Methods:** Prrxl1-knockout and wild type mouse strains age E10.5 to E14.5 were bred and used as the animal model source of DRGs and spinal cord. Human embryos aged 8 to 14 weeks were obtained from the department of pathology in paraffin blocks and served as the human counterpart. Immunohistochemistry was used for sub-population identification with the use of double staining to assert potential co-localizations.

**Results:** In the DRG of both mouse and human, Casz1 is a pan-neuronal transcription factor broadly expressed from early embryonic to E14.4 and 14 weeks respectively, being co-expressed with various lineage markers including Prxxl1. In the spinal cord dorsal horn, Casz1's expression is transient and restricted to progenitors and a subpopulation of differentiating late born type B interneurons.

**Conclusion:** The data presented show that Prxxl1 and Casz1 are similarly expressed in both spatial and temporal terms in both mice and humans. This suggests that developmental events involving the genes under study are equivalent in both species thus supporting the use of the mice model to investigate the development of the somatosensory system.

**PS062 Evaluation subjects with suspected juvenile glaucoma**

Stanislava Seničic’^1^

^1^*School of Medicine;University of Belgrade, Belgrade, Serbia E-mail address: stasa0219@gmail.com (Stanislava Seničic*’*)*

**Introduction:** Juvenile open- angle glaucoma (JOAG) is relatively rare form of childhood glaucoma, typically presenting after four years of age and up to 35 years. Patients with JOAG present with high intraocular pressure (IOP), optic disc excavation, normal angles of gonioscopy and characteristic glaucomatous visual field loss (MD-mean deviation, PSD- pattern standard deviation).

**Aim:** To assess the occurrence of JOAG at our institution and to demonstrate the clinical characteristics of JOAG.

**Methods:** Children involved in this cross-sectional study examined between January 2017 and January 2018 at the Clinic for Eye Diseases, Clinical Center of Serbia in Belgrade as a tertiary institution in Serbia. The children were sent to the Clinic with suspected juvenile glaucoma. All subjects underwent a complete ophthalmic examination by a glaucoma specialist. Diagnostic observation also included a visual field test and scanning laser ophthalmoscopy-Heidelberg retinal tomography (HRT II).

**Results:** This study included 142 (142 eyes) children aged between 4–16 years (mean age of 11 ± 3 years), predominantly female (57%) and 14% of them had JOAG. The IOP values for both eyes ranged from 10 to 32 mmHg (19 ± 4) with positive correlation between higher IOP and the occurrence of glaucoma (r = 0.696, p < 0.001). Mean values of C/D ratio were 0.1 to 0.9 (0,5 ± 0,1) on right eye and 0.1 to 0.8 (0,5 ± 0,1) on left eye and children with JOAG had significantly higher mean values of C/D ratio (p = 0.001). 62% of all children had complained of symptoms of headache while 35% of children with JOAG had a headache (p = 0.38).

**Conclusion:** Most of the children who are referred to our institution with suspected juvenile glaucoma did not have confirmed diagnosis of glaucoma.They have glaucomatous-appearing optic disc without high IOP and with normal visual field and they not need medical treatment.

**PS076 The influence of hypnotic experiences on susceptibility to suggestions in the state of hypnosis**

Anna Dominika Kaczmarska^1^, Ewa Gus’tak^1^, Magdalena Uzar^1^, Jarosław Rachon’^1^

^1^*Department of Psychotherapy Jagiellonian University Medical College E-mail address: annadominikakaczmarska@gmail.com (Anna Dominika Kaczmarska)*

**Introduction:** Hypnosis is a state of consciousness involving focused attention, reduced peripheral awareness and enhanced capacity for response to suggestions.

**Aim:** The aim of the study was to describe potential individual differences in initial susceptibility to suggestions in the state of hypnosis, evaluate the influence of repetitive hypnotic experiences on the susceptibility and present the dynamics of change.

**Methods:** 30 hypnotic procedures, each lasting approximately 11 minutes, were performed by a licensed psychiatrist in the group of 6 participants (medical students: 5 female, 1 male), every one of them underwent 5 hypnosis. The procedures were based on the protocol designed and accepted by the Commission of Clinical Hypnosis of the Polish Psychiatric Association. The research data was acquired using the self-designed questionnaires: one filled out by the participants (subjective assessment), the second one by external raters (objective assessment). The suggestion effects were evaluated using a 1–4 point scale. The susceptibility index was calculated separately for each participant for every procedure. Statistical analysis of the data was accomplished.

**Results:** The initial susceptibility index differed significantly between participants ranging from 1 to 4. Notable changes in responds to particular suggestions were established (2,77 vs 3,07 in the subjective assessment; 2,47 vs 3,1 in objective). Generally the susceptibility changed significantly in the subjective assessment (33,3% 1; 8,3% 2; 25% 3; 33,3% 4 vs 16,7% 1; 8,3% 2; 20,8% 3; 54,2% 4; p = 0,037). However, there was slight difference in the objective assessment.

**Conclusion:** The research shows that susceptibility to suggestions in the state of hypnosis may be modified by different factors including repetitive experiences of the hypnotic relationship. Well-performed procedures may foster an increase in the subjective susceptibility and generate better clinical outcome, crucial for psychiatric and somatic patients treated with hypnosis. However, further research involving larger clinical groups is required.

**PS081 Characteristics of side effects of botulinum A toxin injections as a treatment for cervical dystonia in University Hospital in Kraków**

Paweł Somionka^1^, Radosław Kacorzyk^1^, Anna Iwan'ska^1^, Anna Urban'ska^1^, Katarzyna Graczyk^1^, Gabriela Rusin^1^

^1^*Jagiellonian University Medical College E-mail address: psomionka@gmail.com (Paweł Somionka)*

**Introduction:** Dystonia is the third most common neurological movement disorder after Parkinson's disease and essential tremor. Cervical dystonia (CD) is a typical form of focal dystonia and is characterized by spontaneous movement of head and neck caused by involuntary contractions of neck muscles, which leads to abnormal head posture. The treatment for CD consist of repeated intramuscular injections of botulinum A toxin (BTX). There are three commercially available forms of botulinum toxin: onabotulinumtoxinA (Botox), abobotulinumtoxinA (Dysport) incobotulinumtoxinA (Xeomin)[1].

**Aim:** The aim of our study was to estimate the frequency of side effects (SE) in treatment with BTX injections and its dependency on patients’ sex, age, total number of injections and type of toxin.

**Methods:** The observational retrospective study was performed on medical records of 400 patients with CD treated with BTX injections at the outpatient clinic of University Hospital in Kraków. Collected data was analysed using U Mann-Whitney test and chi-squared test.

**Results:** The study group consisted of 400 patients (70% women) with CD. The average age of patients was 56,2 ± 14,8 and the median of injections number was 13. SE were observed in 79 patients (20%). The most common SE were: dysphagia 25 of 79(31%) patients, head drop 20(25%), neck pain 11(14%), headache 8(10%) and allergic reaction 6(7,5%). 30 of 79(38%) patients had side effects after Botox, 39(49%) after Dysport and 10(13%) after Xeomin. There was statistically significant correlation between the frequency of SE and total number of injections (p < 0,01). However we did not find statistically significant correlation between frequency of SE and patients’ sex(p = 0,12) and age(p = 0,17).

**Conclusion:** Outcomes of study suggest that the most important factor affecting the frequency of side effects is total number of BTX injections. The most common SE was dysphagia. SE were most frequent in patients treated with Dysport. Considering long-term therapy, knowledge about SE is important for successful treatment.

**References**

1. “Treatment of focal dystonia with botulinum toxin A”-Sojer M, Wissel J, Müller J, Poewe W-Wien Klin Wochenschr. 2001;113 Suppl 4:6-10.

**Acknowledgements**

To our tutor of study-Małgorzata Dec-Ćwierk MD, PhD

**PS094 Mu-opioid receptor signalling remains altered in a pain facilitatory area of brain upon cessation of chronic opioid treatment**

Marília Sousa^1,2,3^, Ana Rita Costa^1,2,3^, Isaura Tavares^1,2,3^, Isabel Martins^1,2,3^

^1^*Departamento de Biomedicina, Unidade de Biologia Experimental, Faculdade de Medicina da Universidade do Porto, Portugal,*^2^*i3S, Instituto de Investigação e Inovação em Saúde, Universidade do Porto, Portugal,*^3^*IBMC - Instituto de Biologia Celular e Molecular, Universidade do Porto, Portugal E-mail address: mariliamag.sousa@hotmail.com (Marília Magalhães de Sousa)*

**Introduction:** Opioids are the most common analgesics used to treat moderate to severe pain. However, opioids also cause a paradoxical effect known as Opioid Induced Hyperalgesia (OIH). The exposure to opioids induces counter-adaptations at the μ-opioid receptor (MOR), which contribute to the development of OIH. It is not known whether these alterations remain after cessation of opioids.

**Aim:** Here we aim to study the alterations induced by opioid exposure and upon its cessation in a brain area involved in descending pain facilitation, the dorsal reticular nucleus (DRt), which is under opioidergic inhibition. We first determined, at the behavioural level, the timing of OIH occurrence and also the timing of complete reversal of OIH (Post-OIH). Then, at these timings, we evaluated the expression of MOR and the phosphorylated cAMP response element binding protein (pCREB), a transcription factor whose expression is up-regulated when MOR activation yields paradoxical excitatory cellular effects.

**Methods:** Male Wistar rats were implanted with osmotic mini-pumps for the continuous release of morphine (45 mg-1.ml-1.h-1) or saline. One week later, one group of animals (OIH) was euthanized while in a second group (Post-OIH), mini-pumps were removed and the animals were euthanized 2 weeks later. Nociceptive behaviour was assessed by von-Frey and Hotplate tests, which evaluate mechanical and thermal sensitivity, respectively. The expression of MOR and pCREB at the DRt was evaluated by immunohistochemistry.

**Results:** One week after morphine infusion, animals showed mechanical and thermal hypersensitivity and also an increase of MOR and pCREB expression at the DRt compared to saline. Two weeks after cessation of morphine, behavioural hypersensitivity was completely abolished, MOR expression was not altered while pCREB expression was increased compared to saline.

**Conclusion:** Our results suggest that intracellular cascades remain altered in a pain modulatory area after cessation of morphine, which might impact negatively on the effects of future pain treatments with opioid.

**Acknowledgements**

IASP Early Career Research Grant, NORTE 2020- Programa Operacional Regional do Norte e Fundo Social Europeu (Norte-08–5369-FSE-000026).

**PS130 Investigation of properties of catalase activity of immunoglobulin G in schizophrenia**

Polina Lemeshko^1^, Irina Mednova^2^, Evgeny Ermakov^3^, Arkady Semke^2^, Ludmila Smirnova^2^

^1^*Siberian State Medical University, General Medicine Department, Tomsk,*^2^*Mental Health Research Institute TNRMC,*^3^*Institute of Chemical Biology and Fundamental Medicine, Laboratory of Repair Enzymes, Novosibirsk; Novosibirsk State University, Department of Natural Sciences, Novosibirsk E-mail address: lemeshkopolina@bk.ru (Lemeshko Polina Dmitrievna)*

**Introduction:** Oxidative stress is known to be an important pathophysiological factor in schizophrenia. In our previous studies, it has been shown that IgG in patients with schizophrenia and healthy individuals have the ability to catalyze the decomposition of hydrogen peroxide, i.e. can exhibit catalase and oxidoreductase activities [1,2]. However, the mechanism of the catalase reaction catalyzed by antibodies remains unknown.

**Aim:** To study biochemical properties of catalase activity of IgG in patients with schizophrenia and healthy donors.

**Methods:** Serum of 20 patients with acute phases of schizophrenia from the Mental Health Research Institute Clinic and 30 healthy donors were investigated. IgG were obtained by affinity chromatography using protein-G-Sepharose column. The catalase activity was determined by the rate of hydrogen peroxide decomposition (Beer R.F.). Catalase inhibitor 3-amino-1,2,4-triazole (3-AT) was used for inhibition analysis. Statistical analysis was performed in Statistica 10.0 using the Mann-Whitney test.

**Results:** Using strict criteria such as the electrophoretic homogeneity of the isolated antibodies and the persistence of IgG activity after gel filtration under acidic conditions, we proved that the catalase activity is intrinsic property of IgG. IgG of patients with schizophrenia had 4.3-fold higher catalase activity (1.90 mM/mg protein/min) than that IgG of healthy people (0.44 mM/mg protein/min) (p < 0.05). Catalase-specific inhibitor 3-at reduced catalase activity of IgG in both patients and healthy donors. At a concentration of 50 M 3-AT, the catalase activity of IgG decreased by 50%. The activity is eliminated completely with the concentration of an inhibitor of 1 mM.

**Conclusion:** Inhibition of the catalase activity of IgG by 3-AT, suggests a similar catalytic mechanism with the canonical enzyme catalase. We assume that increase of catalase activity of IgG is the compensatory mechanism which probably reduces an oxidative stress level in patients with schizophrenia.

**References**

1. Ermakov EA, Smirnova LP, Bokhan NA, Semke AV, Ivanova SA, Buneva VN, Nevinsky GA. Catalase activity of IgG antibodies from the sera of healthy donors and patients with schizophrenia. PLoS One. 2017 Sep 25; 12(9):e0183867. doi: 10.1371/journal.pone.0183867.

2. Tolmacheva AS, Blinova EA, Ermakov EA, Buneva VN, Vasilenko NL, Nevinsky GA. IgG abzymes with peroxidase and oxidoreductase activities from the sera of healthy humans. Journal of Molecular Recognition 2015; 28(9):565–580. doi: 10.1002/jmr.2474.

**Acknowledgements**

This work was supported by Russian Science Foundation grant N18–15-00053.

**PS139 Behavioral and EEG aspects of H2S role in epileptic seizures experimentally induced by lindane**

Božo Kneževic’^1^, Nikola Šutulovic’^1^, Anida Ademovic’^1^, Aleksa Lekovic’^1^, Željko Grubač^1^

^1^*Laboratory of Neurophysiology; Faculty of Medicine; University of Belgrade E-mail address: bozo.knezevic@mensa.me (Kneževic*’ *Božo)*

**Introduction:** Hydrogen-sulphide (H2S) belongs to the family of gasotransmitters, with numerous newly discovered physiological and pathological roles. Its role in epileptogenesis is not fully known and can be assessed using GYY4137, slow-releasing H2S donor. Lindane causes generalized seizures that are manifested by pathognomonic ictal graphic elements in electroencephalogram (EEG) and appropriate behavioral changes.

**Aim:** The aim of this study was to examine whether a slow-releasing H2S donor (GYY4137) has a modulatory effect in a model of convulsions induced by lindane.

**Methods:** Wistar albino rats, to which the registration EEG electrodes were implanted, were treated with GYY4137 (75 mg/kg) 30 minutes before intraperitoneal administration of lindane (4 mg/kg). During the following 30 minutes from the administration of lindane, the EEG was registered and behavioral characteristics of convulsions were observed (incidence, latency, and intensity). The number and duration of the ictal periods were analyzed in the obtained records and descriptive scale with grades from 0 to 4 was used to estimate the intensity of the convulsions.

**Results:** Systemic administration of GYY4137 prior to lindane has led to a significant increase in the number of ictal periods, as well as the tendency of prolonging their duration. Incidence showed the tendency to increase and intensity of seizures was significantly increased (p < 0.05), while latency period was decreased, significantly (p < 0.01).

**Conclusion:** The results of this study obtained using GYY4137 as a slow-releasing H2S donor, have shown that H2S has a pro-epileptogenic role in lindane-induced epilepsy in rats.

**PS150 Preterm born effects on neonatal developmental milestones, anxiety fear response and learning and memory**

Leonardo Andrade^1^, Rúben Rocha^1,2^, Tânia Alves^1,3^, Pedro A. Pereira^1,2^, M. Dulce Madeira^1,2^, Armando Cardoso^1,2^

^1^*Department of Biomedicine – Unit of Anatomy, Faculty of Medicine, University of Porto, Alameda Prof. Hernâni Monteiro, 4200–319 Porto, Portugal,*^2^*Center for Health Technology and Services Research (CINTESIS), Faculty of Medicine, University of Porto, Rua Dr. Plácido da Costa,*^*3*^*Faculdade de Ciências da Universidade do Porto, Rua do Campo Alegre, s/n, 4169–007 Porto, Portugal E-mail address: leonardoandrade97@live.com.pt (Leonardo Araújo Andrade)*

**Introduction:** Preterm birth in humans is defined as delivery before 37 weeks of gestation and is a major public health issue. The neurologic impairment found in preterm humans reflect a global lesion of the brain as a result of combined acquired insults, altered developmental trajectories and disorganized reparative phenomena. Despite the knowledge about the pathophysiology of encephalopathy of prematurity, the information is insufficient to allow grounded delivery of specific target treatments.

**Aim:** Characterize the mifepristone (RU486) preterm non-inflammatory model by assessing the neurologic outcome in the neonatal period and anxiety, fear and cognition in adulthood, and compare its effects with those induced by the hypoxia-ischemia model of prematurity.

**Methods:** Pregnant Wistar rats were submitted to laparotomy on embryonic day (ED) 18 for occlusion of uterine arteries during 60 min (TSHI). In another group of Wistar rats, preterm parturition (ED21) was induced by RU486 administration on the previous day (ED20). Sham rats were also laparotomized, but received no treatment. Rat pups were then tested through neonatal developmental milestones. After weaning they were submitted to the following tests: Morris water maze, new object recognition, elevated plus-maze, open-field tests and fear conditioning.

**Results:** Rat pups from ED21 group did not reveal significant alterations in the neonatal developmental milestones and, after weaning, they did not significantly differ from controls in anxiety, fear and cognition. On the contrary, rat pups from TSHI group showed several significant changes in the neonatal developmental milestones, as well as in the anxiety, fear and cognition.

**Conclusion:** The present results suggest that the two-days preterm period was not enough to induce significant alterations in neonatal developmental milestones, and in anxiety, fear and cognition, as it happens when the insult occurs in uterus through hypoxia-ischemia.

**PS172 Evaluation of preterm born effects in the neurogenesis and neuropeptide Y in the hippocampus of young rats**

Tânia Alves^1,2,3^, Rúben Rocha^1,4^, Leonardo Andrade^1^, M. Dulce Madeira^1,4^, Armando Cardoso^1,4^

^1^*Department of Biomedicine – Unit of Anatomy, Faculty of Medicine, University of Porto, Alameda Prof. Hernâni Monteiro, 4200–319 Porto, Portugal,*^2^*Faculty of Sciences, University of Porto, Rua Campo Alegre 687, 4169–007 Porto, Portugal,*^3^*Institute of Biomedical Sciences Abel Salazar, University of Porto, Rua de Jorge Viterbo Ferreira 228, 4050-313 Porto,*^4^*Center for Health Technology and Services Research (CINTESIS), Faculty of Medicine, University of Porto, Rua Dr. Plácido da Costa, 4200–450 Porto, Portugal E-mail address: up201308105@fc.up.pt (Tânia Raquel Cunha Alves)*

**Introduction:** Preterm birth in humans is defined as delivery before 37 weeks of gestation and is a major public health issue. The neurologic impairment found in preterm humans reflect a global lesion of the brain as a result of combined acquired insults, altered developmental trajectories and disorganized reparative phenomena. Despite the knowledge about the pathophysiology of encephalopathy of prematurity, the information is insufficient to allow grounded delivery of specific target treatments.

**Aim:** Characterize the effects of mifepristone (RU486) preterm non-inflammatory model on the neurogenesis and expression of neuropeptide Y (NPY) in the hippocampus of young rats and compare these effects with those induced by the hypoxia-ischemia model of prematurity.

**Methods:** Pregnant Wistar rats were submitted to laparotomy on embryonic day (ED) 18 for occlusion of uterine arteries during 60 min (TSHI). In another group of Wistar rats, preterm parturition (ED21) was induced by RU486 administration on the previous day (ED20). Sham rats were also laparotomized, but received no treatment. One month after weaning, rats were perfused and processed for immunohistochemistry for doublecortin (DCX) and NPY. Areal densities were estimated by counting the number of cells within a given area of the hippocampus.

**Results:** In rats of the ED21 group, there were no significant alterations in the areal density of NPY neurons, but the areal density of DCX cell was significantly reduced. Conversely, in rats of the TSHI group there was a significant reduction in the density of NPY neurons, as well as in the density of DCX neurons in the hippocampus.

**Conclusion:** The present results suggest that albeit the two-days preterm period was not enough to induce significant alterations in the density of NPY neurons in the hippocampus, it was capable to induce alterations in the neurogenesis. Hypoxia-ischemia induced significant changes both in the NPY and neurogenesis.

**Acknowledgements**

This work is supported by ERDF through the operation POCI-01-0145-FEDER-007746 funded by the Programa Operacional Competitividade e Internacionalização – COMPETE2020 and by National Funds through FCT - Fundação para a Ciência e a Tecnologia within CINTESIS, R&D Unit (reference UID/IC/4255/2013).

**PS175 Indicators of oxidative stress in rat neural tissue after chronic exposure to upward and downward oriented static magnetic field**

Sanja Leštarevic’^1^

^1^*Faculty of Medicine, University of Belgrade E-mail address: sanjalestarevic@gmail.com (Sanja Leštarevic*’*)*

**Introduction:** Living organisms are constantly exposed to natural and artificial magnetic field, both static and dynamic. While the biological consequences of dynamic magnetic field are relatively well examined, the effects of static magnetic field (SMF) on biological systems are unclear. The effect of SMF, among other mechanisms, causes increase in reactive oxygen species (ROS) concentration, which are involved in etiopathogenesis of various neurological disorders.

**Aim:** The aim of this research was to examine the effects of chronic exposure to SMF of different orientations on the indicators of oxidative stress in the rat brain.

**Methods:** Animals were randomly divided into three groups (6 animals in each group). The first group was exposed to upward oriented SMF. The second group was exposed to downward oriented SMF, and the third group was the control group. The average magnetic field intensity was 1 mT. After seven weeks, the animals were sacrificed, and brain tissue was taken for further analysis. Concentration of malondialdehyde (MDA) as lipid peroxidation marker, hydrogen peroxide concentration and catalase activity in synaptosomes were measured.

**Results:** Statistically significant increase in MDA levels was found in the experimental group of rats exposed to upward oriented SMF (p < 0.001), as well as in the experimental group exposed to downward oriented SMF (p < 0.01). Significant decrease in catalase activity has been observed in group exposed to downward oriented SMF, both compared to control group (p < 0.05) and to group exposed to upward oriented SMF (p < 0.01). Exposure to SMF did not affect the concentration of hydrogen peroxide in synaptosomes of experimental groups compared to control.

**Conclusion:** Chronic exposure to SMF of moderate intensity led to an increase in MDA levels as a consequence of lipid peroxidation in rat brain. Downward oriented SMF led to decreased catalase activity.

**PS184 Design and development of a new mitochondriotropic antioxidant based on Kojic acid**

João Lourenço^1^, Catarina Oliveira^1^, Fernando Cagide^1^, Fernanda Borges^1^

^1^*CIQUP/Department of Chemistry and Biochemistry, Faculty of Sciences, University of Porto, Rua do Campo Alegre s/n, 4169-007, Porto, Portugal E-mail address: joaomlourenco7@gmail.com (João Manuel Rodrigues Lourenço)*

**Introduction:** Alzheimer's disease is a multi-factorial disease deeply associated with impaired cholinergic transmission and oxidative stress [1]. Data acquired so far have shown that various clinical pathologies including cancer, heart conditions or even neurodegenerative diseases (ND) are associated with mitochondrial dysfunction and oxidative stress [1–3]. In ND cases, this process has been related with the failure of the antioxidant protective system (e.g. enzymes like Superoxide Dismutase) and/or an increment in Reactive Oxygen Species production/accumulation, catalysed by transition metals, which can cause the destabilization of cellular membranes, damage of the blood-brain-barrier, disintegration of DNA and ultimately neuronal death [1–3].

**Aim:** Neuroprotective agents with an extended therapeutic window, namely those able to prevent and/or improve the oxidative stress process are urgently needed. Thus, the aim of this project has been focused on the design and development of an innovative antioxidant targeted to mitochondria using the kojic acid as a scaffold.

**Methods:** In order to achieve this goal, structural changes were performed in the natural-occurring compound by inserting an aliphatic carbon chain spacer linked to a triphenylphosphonium cation (TPP + ). After synthesis, purification and structural identification preliminary in vitro antioxidant non-cell assays and screening toward cholinesterase enzymes (AChE and BChE) has been performed.

**Results:** The mitochondriotropic antioxidant based on kojic acid was successfully obtained following a four-step synthetic strategy. The structural elucidation was performed by NMR and EM. Preliminary data have pointed out that the novel mitochondriotropic antioxidant is an AChE and BChE inhibitor.

**Conclusion:** The results obtained so far will be presented in this communication.

**References**

1. Oliveira C, Cagide F, Teixeira J, Amorim R, Sequeira L, Mesiti F, et al. Hydroxybenzoic acid derivatives as dual-target ligands: mitochondriotropic antioxidants and cholinesterase inhibitors. Front Chem. 2018;6:126.

2. Teixeira J, Oliveira C, Amorim R, Cagide F, Garrido J, Ribeiro J, et al. Development of hydroxybenzoic-based platforms as a solution to deliver dietary antioxidants to mitochondria. Sci Rep. 2017;7:6842.

3. Teixeira J, Cagide F, Benfeito S, Soares P, Garrido J, Baldeiras I, et al. Development of a mitochondriotropic antioxidant based on caffeic acid: proof of concept on cellular and mitochondrial oxidative stress models. J Med Chem. 2017;60:7084-7098.

**Acknowledgements**

This work was funded by FEDER funds through the Operational Programme Competitiveness Factors-COMPETE and national funds by FCT – Foundation for Science and Technology under research grants (QUI/UI0081/2013, NORTE-01-0145-FEDER-000028 and PTDC/DTP-FTO/2433/2014). C. Oliveira and F. Cagide grants are supported by NORTE-01-0145-FEDER-000028.

**PS202 Does flavonoids ameliorate withdrawal-induced effects in the hippocampal formation?**

Débora Inês Costa^1^, Catarina Cruz^2,3^, Armando Cardoso^2,3^, Maria Dulce Madeira^2,3^, Susana Maria Silva^2,3^

^1^*Faculty of Medicine of the University of Porto, 4200-319 Porto, Portugal,*^2^*Unit of Anatomy, Department of Biomedicine, Faculty of Medicine of the University of Porto, 4200-319 Porto, Portugal,*^3^*Center for Health Technology and Services Research (CINTESIS), 4200-450 Porto, Portugal E-mail address: debinesc@gmail.com (Débora Inês Vilas Boas Costa)*

**Introduction:** Flavonoids are polyphenolic compounds pointed as promising candidates in the prevention of neuroinflammation and its consequences [1]. Flavonoids are present in a variety of foods and beverages of plant origin and their ingestion has been associated with the reversal of brain lesions and age-related cognitive decrements [2].

**Aim:** This study aims to assess the potential of flavonoids in ameliorating effects of alcohol withdrawal on neuroinflammation in the hippocampal formation.

**Methods:** Eight-week old male Wistar rats were randomly assigned to three groups: (1) Withdrawn rats were given an aqueous ethanol solution (20%) for 6 months and then switched to tap water for a further 2 months. The ethanol introduction and removal was performed gradually over a 2-week period. (2) Withdrawn + flavonoids rats were treated as withdrawn rats but received, in addition, food pellets with embedded blackberry extract. (3) Control rats had free access to water and pellet food throughout the experiment. During the last month of treatment, rats were tested for anxiety in the open-field and elevated plus-maze and for learning and memory in the Morris water maze. Rats were sacrificed and brains were removed for molecular biology techniques. The expression levels of the pro-inflammatory COX-2, IL-1β, IL-6, TNFα and IL-15 were measured by qRT-PCR in isolated hippocampus.

**Results:** Our results show that the withdrawal-associated impairment of spatial learning and memory was not reversed by flavonoids. Moreover, the decreased anxiety induced by withdrawal was not reversed by flavonoids. In withdrawn + flavonoids rats, IL-15 and IL-1b levels increased significantly by 45% and 165%, respectively, and IL-6 levels decreased significantly, by 42%, relative to those of control and withdrawn rats. TNFa and COX-2 genes did not change between groups.

**Conclusion:** Our results indicate that flavonoids may actually modulate neuroinflammation in hippocampus, namely through changes in IL-1b, IL-6 and IL-15 expression.

**References**

1. Spencer JP, Vafeiadou K, Williams RJ, Vauzour D. Neuroinflammation: modulation by flavonoids and mechanisms of action. Mol Aspects Med. 2012; 33: 83-97.

2. Willis LM, Shukitt-Hale B, Joseph JA. Recent advances in berry supplementation and age-related cognitive decline. Curr Opin Clin Nutr Metab Care. 2009; 12: 91-94.

**Acknowledgements**

Authors wish to thank Professor Rita Negrão for helpful assistance with the molecular biology techniques.

**PS216 Unravelling the mechanisms behind microglia control of purinergic-mediated astroglial proliferation**

Rui Silva^1^, Clara Quintas^1^, Jorge Gonçalves^1^, Glória Queiroz^1^

^1^*Laboratory of Pharmacology, Department of Drug Sciences, Faculty of Pharmacy, University of Porto, Portugal E-mail address: rui.silva.19.07@gmail.com (Rui Filipe Almeida da Silva)*

**Introduction:** Astrocytes and microglia are the main cells coordinating the inflammatory response in the brain. During inflammation, dying or temporarily damaged cells release ATP that, acting on P2 receptors, induce astroglial proliferation. Astrocyte proliferation is important to form a glial scar preventing widespread inflammation and neurodegeneration[1]. Microglia have been shown to prevent astrocyte proliferation elicited by ATP, assuming pivotal roles in the coordination of astroglial responses[2]. Microglia ectonucleotidases metabolise ATP into adenosine, which activates A2 receptors. Microglia also release interleukins and prostaglandins that impair P2 receptors activation in astrocytes[2,3]. Therefore, a number of messengers produced by microglia may contribute to modulate astroglial proliferation induced by ATP.

**Aim:** The present study aims to identify the messengers produced by microglia that prevent ATP-induced astroglial proliferation.

**Methods:** Primary glial cultures were prepared from cortical hemispheres of newborn rats (P0-P2): co-cultures of astrocytes containing 10–15% of microglia, and cultures of astrocytes containing < 1% of microglia. The effect of drugs on astrocyte proliferation was evaluated by methyl-[3H]-thymidine incorporation. Results were analysed by unpaired student's t-test or one-way ANOVA followed by Dunnett's test.

**Results:** In astrocyte cultures, ATP-gamma-S (100 uM) increased astroglial proliferation to 185 ± 5% (n = 3; P < 0.05), an effect attenuated in co-cultures to 125 ± 2% (n = 3). In co-cultures, the effect of ATP-gamma-S was not increased by the selective A2A receptor antagonist, SCH 58261 (30 nM) nor by the selective A2B receptor antagonist, MRS 1706 (10 nM), however, it was restored by indomethacin (10 uM; 171 ± 16%; n = 4, P < 0.05). Indomethacin (10 uM) did not change the ATP-gamma-S effect in astrocyte cultures.

**Conclusion:** The results show that microglia impairment of ATP-gamma-S-mediated astroglial proliferation is independent of tonic activation of A2 receptors by adenosine but may involve eicosanoids released by microglia. This microglia-astrocyte communication may be relevant to fine tune the inflammatory response, allowing immune cells infiltration in the damaged area, before glial scar formation.

**References**

1. Burda and Sofroniew, 2014, Neuron 81:229-248.

2. Quintas et al., 2018, Front. Pharmacol. 9:418.

3. Paniagua-Herranz et al., 2017, Front. Pharmacol. 8:937.

**Acknowledgements**

This study was supported by Faculty of Pharmacy, University of Porto.

**PS237 The evaluation of the usefulness of measuring hypocretin-1 among patients with suspected narcolepsy in diagnostic uncertainty**

Agata Gabryelska^1,2^, Bartosz Szmyd^1^

^1^*Department of Sleep Medicine and Metabolic Disorders, Medical University of Lodz, Poland,*^2^*Department of Sleep Medicine, University of Edinburgh, UK E-mail address: bartosz.szmyd@stud.umed.lodz.pl (Bartosz Mirosław Szmyd)*

**Introduction:** Excessive daytime sleepiness (EDS) is a common sleep-related complaint. Continuous EDS is a diagnostic necessity for a narcolepsy - the disorder characterized by decreased ability to control sleep-wake cycles. One of the pathophysiologies associated with narcolepsy is a loss of hypocretin-1 (orexin) secreting neurones in the hypothalamus. This specific kind is called narcolepsy type 1 (NT1). The diagnosis criteria of NT1 are: EDS for at least 3 months and cerebrospinal fluid (CSF) hypocretin-1 concentration lower than 110 picograms/mL.

**Aim:** The aim of the research was to evaluate the usefulness of measuring hypocretin-1 in patients with suspected narcolepsy in diagnostic uncertainty.

**Methods:** The study included 27 patients of Scottish sleep clinic (Department of Sleep Medicine, University of Edinburgh) presenting excessive daytime sleepiness (EDS) to determine their uncertain narcoleptic status. All of them were examined in: extensive interview, polysomnography followed by multiple sleep latency test (MSLT), two weeks of actigraphic and sleep log recordings. Additionally, HLA-typing and a lumbar puncture to measure CSF hypocretin-1 levels were carried out.

**Results:** 18 patients had decreased hypocretin-1 level in CSF, which is 67% of whole group. Final diagnoses among these patients were: NT1, NT2 and idiopathic hypersomnia. Performed analysis of predictive potential of orexin measurement in NT1 diagnosis revealed: positive predictive value (PPV) equals 72%, specificity: 64%. Both, negative predictive value (NPV) and sensitivity, are 100%.Moreover, decreased orexin level was observed among patients with depression episode(s) in past medical history, who are not affected by narcolepsy (2 from 5).

**Conclusion:** Measuring CSF hypocretin-1 is a useful parameter in NT1 detection (100% NPV and sensitivity). Unfortunately, due to lower PPV and specificity it should not be used to confirm NT1 among patients with complex clinical course, especially history of depression.

**PS256 Magnetic resonance imaging findings in pediatric migraine and tension-type headache patients - experience from the University Children's Hospital in Belgrade, Serbia**

Michel Marjanovic^1^

^1^*University of Belgrade, Faculty of Medicine, University Children's Hospital Belgrade E-mail address: miche939@gmail.com (Michel Marjanovic)*

**Introduction:** Headache is a commonly present among the pediatric population. Magnetic resonance imaging (MRI) is utilized for detection of significant and treatable lesions that can be life threatening, requiring further treatment.

**Aim:** To determine the value of neuroimaging in children and adolescents with migraine and tension-type headache and estimate the frequency and severity of intracranial abnormalities.

**Methods:** The retrospective cross sectional study included 69 pediatric patients; 27 (39,1%) male and 42 (60,9%) female, with definite diagnosis: migraine or tension-type headache by ICHD- III classification [1] in the period: January 2015 – December 2017. A database of demographic data and MRI findings was analyzed, classifying patients according to need for further management.

**Results:** Migraine was present in 44 (63,8%) patients with average age of onset 12.2 ± 3.4 years and for Tension-type in 25 (36,2%) patients, aged 11.6 ± 3.8 years. No significant relation presented between headache diagnosis and demographic parameters. Minor developmental anomalies of Hippocampus, unilateral ventriculomegaly, borderline Chiari malformation and cysts of: Epiphysis, Arachnoid, Choroid plexus were MRI findings in 22 (31,9%) participants (p = 0,149) and insignificant statistically to further management; hence incidental benign. Venous angioma, Chiari I/II malformation, changes in sinuses or mastoids and cortical gliosis were seen in 23 (33,3%) subjects (p = 0,861); similarly statistically insignificant; therefore classified as incidental pathological. True pathological findings: Sinusitis, Mastoiditis, Hypophysis microadenoma, Sphenoid bone cysts and periventricular focal lesions, were present in 20 (29%) patients and significant for further management (p < 0,001). By logistic regression, patients with true and incidental pathological findings were 492,5 (p < 0,001) and 16,2 (p = 0,018) times more likely to need further management, respectively.

**Conclusion:** From 69 pediatric patients with Migraine and Tension-type headache with performed MRI; changes were found in 48 (69,6%) participants. Further management was required in 26 (54,2%) of patients with MRI abnormalities.

**References**

1. Headache Classification Committee of the International Headache Society (IHS) The International Classification of Headache Disorders, 3rd edition. Cephalalgia. 2018 Jan;38(1):1-211. doi: 10.1177/0333102417738202. PubMed PMID: 29368949.

**Acknowledgements**

Mentor: Doc. Dr. Dragana Bogicevic, Statistical aid: Mr. Marko Savic

Oncology & Molecular Biology

**PS023 Analysis of polymorphism -174G/C in the promotor region of the Interleukin 6 gene in cervical cancer**

Katarina Ivanovic’^1^, Stefan Ivanovic’^1^

^1^*Faculty of Medicine, University of Belgrade E-mail address: ivanoovic93@gmail.com (Stefan Ivanovic*’*)*

**Introduction:** Cervical cancer is one of the most common causes of mortality in women. It takes the sixth place in more developed countries and the second place in less developed ones. The occurrence of cervical cancer is multifactorial. Risk factors such as early initiation of sexual practice, large number of sexual partners, low socio-economic conditions, the use of oral contraceptives, lifestyle, genetic factors play an important role. Within genetic and immunological factors, one more closely examined is IL6, a cytokine which has an important role in development of inflammation and tumorigenesis. Polymorphism in the promoter region of IL6 -174G/C affects the intensity of the transcription and the expression of the gene.

**Aim:** The aim of study was to analyze the frequencies of genotypes and alleles at locus IL6 -174G/C in patients with cervical cancer, and to compare data with the control group, in order to determine whether one of the variants predisposes for the occurrence of disease.

**Methods:** The study included 80 women, of whom 30 were affected with invasive cervical cancer, while 50 women had a normal gynecological exam. Testing methodology included DNA isolation, allele-specific PCR for amplification of the polymorphic region (-174 G/C) and analysis of PCR products by gel electrophoresis.

**Results:** In the healthy group GG genotype dominated (43%), while in experimental group CG genotype was the most common (43.33%). There isn’t significant difference between the frequencies of genotypes. In experimental group the more frequent was allele C, while in control group the allele G (in both cases frequency is 0.52). Without Yates correction there is significant differences in alleles frequencies (p = 0.045304), while with Yates correction it isn’t (p = 0.066).

**Conclusion:** The polymorphism in the promoter region -174 G/C IL6 gene wasn’t associated with development cervical cancer in our patients, but further extensive studies are needed.

**PS071 Tumor budding in tumour tissue among operatively treated patients with lung adenocarcinoma**

Milena Vasilijevic’^1^

^1^*Faculty of Medicine Novi Sad E-mail address: vasilijevic93@gmail.com (Milena Vasilijevic*’*)*

**Introduction:** Lung adenocarcinoma, which represents 30%- 40% of all lung carcinoma, is one of the most significant research subjects in pulmonary pathology domain.

**Aim:** Determine the correlation between the presence and density of tumor budding and tumor size, nodal status and stage of disease within operatively treated patients with lung adenocarcinoma.

**Methods:** The study included 31 patient, operatively treated for lung adenocarcinoma during 2016. Microscopic analysis of routine histological slides was performed to establish the presence and density of tumor buds by Ueno method. These results were compared to tumor size, nodal status and stage of disease.

**Results:** In regard to gender, group of patients with tumor budding included 13 men (72,2%) and 5 women (27,8%). There were 7 men (53,8%) and 6 women (46,2%) in the group of patients without present tumor budding. There was no statistical significance found between males (66 ± 5,41) and females (65 ± 4,89) in the group with tumor budding, nor in the group without tumor budding (males 61 ± 5,99; females 60 ± 9,84), age considering. Statistically significant result in tumor size between groups of patients with (4,04 ± 1,98) and without (4,02 ± 2,18) tumor budding was not found. Group of patients with present tumor budding and in nodal status N1 pointed to statistical significance (p < 0,01). There was statistically significant presence of tumor budding in stage II and III of disease (p < 0,01).

**Conclusion:** Statistical significance in gender between groups of patients with and without tumor budding was not found. There was no statistically significant result in tumor size between groups of patients with and without tumor budding. Statistical significance was found between patients with present tumor budding and nodal status N1. Tumor budding was frequently present in acinar, solid and papillary type of lung adenocarcinoma.

**PS084 Accuracy and reliability of preoperative diagnosis in determining histopathologic tumor type and grade in patients with endometrial carcinoma**

Ivor Kolarski^1^

^1^*Faculty of Medicine University of Novi Sad E-mail address: ivor.kolarski993@gmail.com (Ivor Kolarski)*

**Introduction:** Endometrial carcinoma is the most common gynecological malignancy in the Western world. The most frequent sign of endometrial cancer is uterine bleeding, which occurs in more than 90% of cases, especially in postmenopausal women. Endometrial sampling is usually performed by dilatation and curettage. Preoperative histopathological finding is an important cornerstone in classifying patients in low- or high-risk groups for cancer spread, so its accuracy is significant in determining the extent of surgical treatment.

**Aim:** The primary aim of the study is to determine the agreement rate on tumor grade and histologic type between preoperative endometrial sampling and final diagnosis after operative treatment. The secondary aim is to determine the level of clinically relevant postoperative tumor upgrading and downgrading.

**Methods:** Retrospective analysis of 196 women with endometrial carcinoma from the period October 2012 – October 2017, that underwent oncologic evaluation and surgical treatment at Oncology Institute of Vojvodina, of whom 146 patients met all including criteria and were statistically analyzed. In this group of 146 patients, tumor grade and histologic type between preoperative and postoperative diagnosis were compared.

**Results:** Results showed moderate agreement between preoperative and postoperative histologic type with the highest agreement in the group of preoperative endometrioid adenocarcinoma. Low agreement on tumor grade between endometrial sampling and final diagnosis was found. The lowest agreement was in the group of preoperative grade 1 tumors, of whom more than 40% were reclassified as higher grade on final diagnosis.

**Conclusion:** Although is a cornerstone in determining the extent of surgical treatment, preoperative histopathological finding can be unreliable and insufficient in classifying patients in high- or low-risk groups. Tumor markers or immunohistochemical biomarkers might help to preoperatively differentiate between high- and low-risk tumors, as well as preoperative imaging techniques.

**PS096 Immune microenvironment-related gene expression in ovarian tumours**

Milda Ranc̆elytë^1^, Evelina Bojarska^1^, Agata Mlynska^2^

^1^*Faculty of Medicine, Vilnius University, Lithuania,*^2^*National Cancer Institute, Vilnius, Lithuania E-mail address: milda.rancelyte@gmail.com (Milda Ranc̆elytë)*

**Introduction:** Ovarian cancer tends to recur and become resistant to platinum-based chemotherapy. More detailed exploration of cancer immunology can be beneficial in detecting potential immunotherapy targets.

**Aim:** To determine the correlation between tumour microenvironment-related gene expression in ovarian tumours and overall survival (OS) or progression-free survival (PFS); to compare gene expression between the groups of chemotherapy-sensitive and chemotherapy-resistant patients.

**Methods:** 35 patients diagnosed with stage III-IV ovarian cancer and treated with adjuvant platinum-based chemotherapy were enrolled. OS and PFS were estimated. Patients were divided into groups based on their chemosensitivity and tumour progression. Quantitative polymerase chain reaction was used to assess the expression of genes associated with immune response and tumour infiltration.

**Results:** At the time of diagnosis the average age of the patients was 60.1 ± 11.2 years. Tumour grades were distributed as follows: G1–13.6%, G2–4.5%, G3–81.8% among chemosensitive patients; G2–15.4%, G3–84.6% among chemoresistant patients. Correlation was found between OS and expression of CXCL9 (r = 0.459, p < 0.05), COL5A1 (r = 0.503, p < 0.05) and GZMB (r = -0.348, p < 0.05) genes. Correlation was found between PFS and expression of CTLA4 (r = -0.436, p < 0.05), COL5A1 (r = 0.353, p < 0.05), GZMB (r = -0.378, p < 0.05) genes. Chemoresistant tumours had greater expression of IFN-g (1.9 times, p < 0.05) and TAP-1 (1.9 times, p < 0.05) genes in comparison to chemosensitive tumours. Chemosensitive tumours had greater expression of CXCL9 (3.2 times, p < 0.05), POSTN2 (3.8 times, p < 0.05), TAP-2 (1.6 times, p < 0.05) genes than chemoresistant patients. Non-progressing tumours had greater expression of CXCL9 (3.4 times, p < 0.05), CTLA4 (2.9 times, p < 0.05), VEGF-A (1.4 times, p < 0.05) genes than progressing tumours.

**Conclusion:** Chemosensitive and chemoresistant patients have variable expression of genes, associated with immune response and tumour infiltration. Greater expression of CXCL9 and COL5A1 in ovarian cancer tissue is associated with better survival rates, whereas greater expression of GZMB and CTLA4 is associated with worse survival rates.

**PS112 In vitro evaluation of nanostructured functionalized platforms for the sustained delivery of cisplatin**

Paulo Pereira^1^, Daniel Gaspar^1^, Claúdia Silva^1^, Fátima Martel^1,2^, Bárbara Joana Martins Leite Ferreira^3,4^, Teresa Margarida Santos^3,4^, Ana Luísa Daniel-da-Silva^3,4^

^1^*Unit of Biochemistry, Department of Biomedicine, Faculty of Medicine, University of Porto, Portugal,*^2^*I3S, University of Porto, Portugal,*^3^*Department of Chemistry, University of Aveiro, 3810–193 Aveiro, Portugal,*^4^*CICECO, University of Aveiro, Portugal E-mail address: paulopereira-07–10@hotmail.com (Paulo Alexandre Mesquita Pereira)*

**Introduction:** Cisplatin has an extraordinary anticancer activity against a variety of solid tumors. However, its clinical efficacy is contrasted by its toxicity profile.

**Aim:** To evaluate the cytotoxic and antiproliferative effect of core–shell functionalized nanoparticles prepared by our group.

**Methods:** Cytotoxicity (MTT assay), necrosis (LDH assay) and proliferation (3H-thymidine incorporation assay) will be evaluated in pancreatic cancer (PANC-1 and AsPc-1) and non-cancer (H6C7) cell lines.

**Results:** Cisplatin showed a concentration-dependent cytotoxic effect on both cancer and non-cancer cell lines. The cytotoxic effect of cisplatin-loaded nanoparticles (CisNano) towards cancer cell lines was less pronounced than that of cisplatin but, contrary to cisplatin, CisNano was less cytotoxic toward non-cancer cells.Cisplatin and CisNano showed a concentration-dependent necrotic effect in the three cell lines, with CisNano being more potent in relation to the cancer cell lines than cisplatin. Moreover, CisNano induced a much stronger necrotic effect in the two cancer cell lines than in the non-cancer cell line. In contrast, the necrotic effect of cisplatin was similar in a cancer (Panc-1) and a non-cancer cell line. Finally, cisplatin presented a more marked antiproliferative effect than CisNano in relation to all cell lines. However, the antiproliferative effect of cisplatin was similar in a cancer (AspC-1) and the non-cancer cell line (H6C7). In contrast, CisNano presented a much more marked antiproliferative effect in the two cancer cell lines than in the non-cancer.

**Conclusion:** CisNano, in comparison with cisplatin, is more potent in inducing necrosis but less potent in reducing cell viability and proliferation. However, in contrast to cisplatin, CisNano possesses a much more discrete effect in non-cancer pancreas cells than in cancer cells. So, these conjugates possess a higher selectivity toward cancer cells and may be promising carriers for intravesical delivery of cisplatin for pancreatic cancer therapy.

**PS125 Vitamin D modulates immune checkpoint molecule expression in head and neck squamous cell carcinoma patients**

Helge A. Krebs-Fleischmann^1^, Florian Bochen^1^, Bernhard Schick^1^, Maximilian Linxweiler^1^

^1^*Department of Otorhinolaryngology, Head and Neck Surgery; Saarland University Medical Center, Homburg, Germany E-mail address: hkfawj@googlemail.com (Helge Anand Krebs-Fleischmann)*

**Introduction:** Vitamin D deficiency is often observed in human cancer patients, particularily among patients with Head And Neck Squamous Cell Carcinoma (HNSCC). According to recent work by Bochen et al. (2018) [1], cancer patients with low 25-OH-vitamin D serum levels often had a negative HPV status, were afflicted with a higher rate of lymphogenic metastasis and displayed altered intra- and peritumoral immune cell infiltrate levels, thus indicating poor prognosis.

**Aim:** Considering the evidence gathered in recent years that vitamin D has a modulatory effect on the patients’ antitumor immune response, we examined to what extent vitamin D influences the immunological interplay between tumor and host immune cells on a molecular level in head and neck cancer patients by focusing on a subset of immunecheckpoints.

**Methods:** Tumor tissue samples from 115 HNSCC patients, of which 52 had adequate serum levels of vitamin D (> 10 ng/ml) while 63 exhibited a vitamin D deficiency (< 10 ng/ml), were subjected to conventional immunohistochemistry in order to analyze the tumor expression of the immunoregulative proteins MHCI, B7-H4, B7-H3, PD-L1 and CD80. Additionally, MHC1 and PD-L1 expression was studied on intra- and peritumoral leukocytes.

**Results:** The evaluation of the immunohistochemical stainings with an immunoreactive score demonstrated that an adequate vitamin D status was associated with a higher tumor expression of CD80, B7-H3 and MHC1. In contrast, vitamin D deficiency correlated with an increased B7-H4 tumor expression. Intra- and peritumoral leukocytes expressed more CD80 among patients with higher vitamin D levels. No considerable influence was detectable on the expression of PD-L1 on both tumor and leukocytes.

**Conclusion:** In conclusion, these observations concord to suggest that vitamin D stimulates the immunological antitumor activity in head and neck cancer patients by improving the ratio of activating and inhibitory immunecheckpoint receptors in the tumormicroenvironment, favoring tumor specific T-cell immune responses and hindering immune evasion mechanisms.

**References**

1. Bochen F, Balensiefer B, Körner S, Bittenbring JT, Neumann F, Koch A, Bumm K, Marx A, Wemmert S, Papaspyrou G, Zuschlag D, Kühn JP, Al Kadah B, Schick B and Linxweiler M. Vitamin D deficiency in head and neck cancer patients – prevalence, prognostic value and impact on immune function. OncoImmunology. 2018; in press; doi:10.1080/2162402X.2018.1476817

**PS126 Expression of HIF-1α in colon mucosa 10 and 20 cm away from malignant tumor**

Valentina Balint^1^, Aleksa Jovanovic’^1^, Sanja Despotovic’^2^

^1^*Faculty of Medicine University of Belgrade, Belgrade, Serbia,*^2^*Institute of Histology and Embryology “Aleksandar Đ. Kostic*”*’, Faculty of Medicine University of Belgrade, Belgrade, Serbia E-mail address: tina.balint@gmail.com (Valentina Balint)*

**Introduction:** During malignant progression, existing blood vessels are unable to supply growing tumor with higher oxygen and nutrients demands, which leads to the development of a hypoxic microenvironment. Hypoxia-inducible factor-1a (HIF-1a) is a transcription factor which regulates expression of many genes responsible for cell adaptation to hypoxia. Expression of HIF-1a is increased in colon carcinoma and transitional mucosa.

**Aim:** The aim of this paper was to determine whether there are some changes in HIF-1a expression in colon mucosa 10 cm and 20 cm away from colon adenocarcinoma.

**Methods:** Samples of colon mucosa (n = 5) 10 cm and 20 cm away from the tumor were obtained by biopsy while examining patients with malignant tumors of this organ and control samples (n = 5) were obtained during colonoscopy of healthy patients at the “Zvezdara” Hospital in Belgrade. The samples were immunohistochemically stained using HIF-1a antibodies. The Icy software determined the presence of HIF-1a in the superficial epithelium, the percentage of HIF-1a-positive crypts in the mucosa, and the percentage of HIF-1a-positive cells in every crypt.

**Results:** In comparison to superficial epithelium of colon mucosa of healthy examinees, there is increased expression of HIF-1α (%) 10 cm and 20 cm away from tumor. Also, there is significantly increased expression of HIF-1α at 10 cm compared to superficial epithelium 20 cm away from tumor.

The percentage of HIF-1a positive crypts is significantly greater 10 cm and 20 cm away from tumor in comparison to mucosa of healthy examinees. The percentage of HIF-1a positive cells within positive crypts is significantly greater 10 cm and 20 cm away from tumor compared to healthy examinees.

**Conclusion:** There is increased expression of HIF-1a in seemingly healthy colon mucosa 10 cm and 20 cm away from tumor compared to controls. This might be explained by the “field carcinogenesis” theory but further investigations are required.

**PS142 Investigation of association between ACE gene insertion-deletion (I/D) polymorphism and atherosclerosis in hemodialysis patients**

Veljko Banduka^1^, Nela Maksimovic^1^

^1^*Institute of human genetics, Faculty of Medicine University of Belgrade E-mail address: veljkobanduka@gmail.com (Veljko Banduka)*

**Introduction:** The angiotensin-converting enzyme (ACE) plays an important role in vascular wall homeostasis, blood pressure regulation and is considered relevant in the pathogenesis of cardiovascular diseases. In the patient population with chronic kidney disease, cardiovascular diseases are one of the leading cause of mortality. In hemodialysis patients, atherosclerotic lesions in blood vessels may be different from those in non-uremic subjects. Higher ACE levels and activity in blood and tissue, which are under direct genetic control, pose a known risk factor for aforementioned atherosclerotic lesions.

**Aim:** The aim of this study was to investigate the association of insertion/deletion (I/D) polymorphism in the ACE gene with intima-media thickness of both carotid arteries and presence of atherosclerotic plaques, as markers of cardiovascular diseases in hemodialysis patients.

**Methods:** Our study included 54 hemodialysis patients, mean age 54.4 years. Serum total cholesterol and triglycerides were measured. The expression of atherosclerosis was estimated by measuring intima-media thickness of both carotid arteries and based on presence of atherosclerotic plaques. The analysis of I/D polymorphism was performed by PCR method.

**Results:** There was no statistically significant difference in genotype frequencies (p = 0.494) between patients without atherosclerosis plaques or with minor stenosis, and patients with medium or severe stenosis. Also, there was no statistically significant difference in intima-media thickness depending on the ACE genotype on left (p = 0.880), or right carotid artery (p = 0.304).

**Conclusion:** Our results show that there is no statistically significant association of insertion/deletion (I/D) polymorphism of ACE gene with atherosclerosis development in the population of hemodialysis patients.

**Acknowledgements**

Participant at the 59th Congress of Students of Biomedical Science of Serbia with international participation - reward for the best research-paper on session

**PS160 Cytotoxicity of novel pyridine-fused chlorins as promising PDT agents against different cancer cell lines**

João Simões^1^, Mafalda Laranjo^2,3^, Nelson Pereira^1^, Teresa Pinho e Melo^1^, Maria Filomena Botelho^2,3^, Gonçalo Brites^2,3^, Bruno Nascimento^1^, Marta Piñeiro^1^

^1^*CQC and Department of Chemistry, University of Coimbra, Coimbra, Portugal,*^2^*Biophysics Institute; Institute for Clinical and Biomedical Research (iCBR), area of Environment Genetics and Oncobiology (CIMAGO); Faculty of Medicine, University of Coimbra, Coimbra, Portugal,*^3^*CNC.IBILI, University of Coimbra, Portugal E-mail address: salgueirosimoes@hotmail.com (João Carlos Salgueiro Simões)*

**Introduction:** The high selectivity of photosensitizers to solid tumors and the generation of cytotoxic reactive oxygen species (ROS) near the target minimize the side effects usually observed with common systemic drugs, giving photodynamic therapy (PDT) several advantages over the classic anticancer therapies.

**Aim:** The aim of this work is to disclose the synthetic details, structural characterization and cytotoxicity evaluation of new and very promising PDT agents against oesophagus cancer cell line (OE19) and melanoma cell line (A375).

**Methods:** Novel 4,5,6,7-Tetrahydropyrazolo[1,5-a]pyridine–fused chlorins were synthesized as were the corresponding PEGylated derivatives. The colorimetric MTT (3-(4,5-dimethylthiazol-2-yl)-2,5-diphenyltetrazolium bromide) test was used to evaluate the effect of these compounds on the metabolic activity. For this study, previously cultured OE19 and A375 cells were seeded in 48-well plates and, after 24 h, incubated with increasing concentrations of these compounds (50 nM – 10uM). After 24 h, the cells were irradiated with a proper light source (lcut-off < 560 nm) until a total of 10 J. MTT assay was performed 24 h later. Dose response curves were plotted and IC50 (half-maximal inhibitory concentration) values for each compound were determined.

**Results:** Although the di-ester chlorin showed IC50 values between 5 M and 10 M against OE19 and A375, their corresponding diol and PEGylated derivatives showed higher cytotoxicity with IC50 values up to only 250 nM against the OE19 cancer cell line and around 125 nM and 1 M, respectively, against A375. All of the studied compounds revealed dose-dependent anti-proliferative effects, and derivatives with increased hydrophilicity either obtained by the reduction of di-ester to diol derivative or by the addition of PEG moieties, showed a much higher activity against both the OE19 and A375 cancer cell line.

**Conclusion:** Therefore, we can conclude that we were able to develop new PDT agents that not only show enhanced chemical and structure stability, but also increased hydrophilicity and consequent higher cytotoxicity.

**Acknowledgements**

This work was funded by Fundação para a Ciência e a Tecnologia (FCT): POCI-01-0145-FEDER-PTDC/QEQ-MED/0262/2014 (COMPETE 2020); POCI-01-0145-FEDER-007630 and POCI-01-0145-FEDER-007440.

**PS169 Increased diagnostic accuracy in colorectal carcinoma using tumor budding aided by cytokeratin immunohistochemistry**

Navin Mallya^1^, Mahima Gupta^1^, Jyothi Kini^2^, Hema Kini^2^, Saraswathy Sreeram^2^

^1^*Kasturba Medical College, Mangalore, India,*^2^*Department of Pathology, Kasturba Medical College, Mangalore, India E-mail address: navinmallya@gmail.com (Navin Mallya)*

**Introduction:** Colorectal carcinoma is one of the leading causes of cancer-related death worldwide [1]. Prognosis and treatment decisions are established on the extent of the disease, as per TNM staging system [2]. However, outcome can vary within stage groups. The presence of tumor buds in colorectal carcinoma has a significant value related to local recurrence of disease, lymphovascular invasion, metastasis, and poor disease-specific survival. Tumor budding is defined as the presence of de-differentiated single cells or small clusters of upto 5 cells at the invasive front of the colorectal cancer[3].

**Aim:** The objective of this study is to describe the morphology of tumor budding and to determine the utility of pancytokeratin immunohistochemistry in detection of tumor budding in patients who undergo resection for colorectal carcinoma.

**Methods:** A retrospective cross-sectional study of all patients who underwent resection for colorectal cancer from January 2017 to May 2018 was done. The study was conducted at KMC Mangalore, India.Two pathologists independently evaluated tumor budding on routine Hematoxylin and Eosin stained formalin fixed, paraffin embedded sections as well as cytokeratin immunostained sections.

**Results:** A total of 50 colectomies were evaluated. The age range of the patients varied from 36 to 73 years with a male preponderance. Majority of the cases (46%) were moderately differentiated and most (41%) were TNM stage II. The analysis revealed a significant association between tumor budding and grade and stage of the tumor. The interobserver agreement was good between the two pathologists and improved with cytokeratin stained evaluation.

**Conclusion:** Colorectal carcinoma patients within the same disease stages tend to show heterogeneous outcomes. Since tumor budding can be quantitated especially with the aid of cytokeratin immunohistochemistry, its assessment has the capacity to enhance diagnostic accuracy and scoring tumor buds should become a component of the standard pathological assessment of colorectal carcinomas.

**References**

1. Koelzer V, Zlobec I, Lugli A. Tumor budding in colorectal cancer—ready for diagnostic practice?. 2018.

2. T-cell infiltrates and tumor budding: promising prognostic factors in the tumor microenvironment of colorectal cancer. Colorectal Cancer. 2013;2(3):173-176.

3. Dawson H, Lugli A. Molecular and Pathogenetic Aspects of Tumor Budding in Colorectal Cancer. Frontiers in Medicine. 2015;2.

**PS185 Alvespimycin is a promising drug in resistant Chronic Myeloid Leukemia**

Diogo Santos^1^, Raquel Alves^1,2^, Ana Cristina Gonçalves^1,2^, Joana Jorge^1,2^, Henrique Girão^3^, Steve Catarino^3^, Joana Barbosa de Melo^2,4^, Ana Bela Sarmento-Ribeiro^1,2,5,6^

^1^*Laboratory of Oncobiology and Hematology and University of Hematology,*^2^*Coimbra Institute for Clinical and Biomedical Research (iCBR) - Group of Environment Genetics and Oncobiology (CIMAGO),*^3^*Coimbra Institute for Clinical and Biomedical Research (iCBR),*^4^*Cytogenetics and Genomics Laboratory, Faculty of Medicine University of Coimbra,*^5^*Clinical Hematology Department, Centro Hospitalar e Universitário de Coimbra (CHUC),*^6^*Center for Neuroscience and Cell Biology (CNC), University of Coimbra, Coimbra, Portugal E-mail address: diogo.fmuc.santos@gmail.com (Diogo Pires Dos Santos)*

**Introduction:** HSP90 belongs to the heat shock protein family, a functional class of chaperone molecules that facilitates the maturation, stability, activity, and intracellular folding of more than 200 proteins. One of these proteins is BCR-ABL, the oncoprotein responsible for Chronic Myeloid Leukemia (CML). HSP90 inhibitors, by preventing nucleotide-dependent cycling interfere with the chaperone activity of HSP90, resulting in targeting of client proteins to proteasome degradation.

**Aim:** This work aims to study the effect of alvespimycin (17-DMAG), in monotherapy and in combination with imatinib, in chronic myeloid leukemia cell lines (sensitive and resistant to imatinib).

**Methods:** This study was performed in three CML cells lines: K562 cells, sensitive to Imatinib, and K562-RC and K562-RD cells resistant to Imatinib. Cells were incubated with 17-DMAG in monotherapy and in combination with 100 nM of Imatinib. Dose-response curves were determined by resazurin assay. Cell death was performed by microscopy (May- Grunwald Giemsa staining) and flow cytometry (FC; Annexin V/Propidium Iodide (PI) double staining). Caspase levels, mitochondrial membrane potential and cell cycle distribution were evaluated by Apostat, JC-1, and PI/RNase assay respectively, by FC. Expression levels of HSP family were analyzed by western blot.

**Results:** Our results showed that 17-DMAG induce a reduced metabolic activity in a time- and dose-dependent manner, alone and in combination with Imatinib, with an IC50 of approximately 50 nM for K562 and K562-RD cells and lower than 50 nM for the K562-RC cell line, after 48 hours of treatment. This compound induces cell death predominantly by apoptosis with mitochondria involvement. Furthermore, 17-DMAG induces cell cycle arrests in K562 in G0/G1 phase. K562 cells have slightly higher HSP90 expression comparing with imatinib resistant cells.

**Conclusion:** Our results suggest that alvespimycin could be used as a new potential approach in the treatment of CML, even in case of Imatinib resistance.

**PS187 Tetrahydrocannabinol (THC) exposure alters leptin and leptin receptor expression in placenta**

Débora Sofia Gonçalves^1^, Bruno Fonseca^1,2^, Luís Midão^1,2^, Marta Almada^1,2^, João Maia^1,2^, Jorge Braga^3^, Daniela Gonçalves^3^, Natércia Teixeira^1,2^, Georgina Correia da Silva^1,2^

^1^*Laboratório de Bioquímica, Faculdade de Farmácia Universidade do Porto, Porto, Portugal,*^2^*UCIBIO-REQUIMTE, Porto, Portugal,*^3^*Departamento da Mulher e da Medicina Reprodutiva, Serviço de Obstetrícia, Centro Materno-Infantil do Norte-Centro Hospitalar do Porto, Porto, Portugal E-mail address: up201305358@ff.up.pt (Débora Sofia Alves Gonçalves)*

**Introduction:** Tetrahydrocannabinol (THC) is the psycoactive compound of Cannabis sativa, the most common drug used by pregnant women. THC can cross the placental barrier due to its lipophilicity, which can cause the clinically well described pregnancy-related complications such as low birth weight, prematurity, intrauterine growth retardation and perinatal death in pregnant women consumers. THC may therefore affect placental development. Leptin (LEP), which plays an essential role in reproduction, is increased in placentas from complicated pregnancies. However, the effects of THC exposure in placental leptin regulation are still unknown.

**Aim:** Thus, the aim of this work was to study the impact of THC in LEP and its receptor (LEPR) expression.

**Methods:** For that purpose, a representative model of cytotrophoblast, the BeWo cell line and term placental explants were exposed to 10, 20 and 40 μM of THC for 24 h. LEP and LEPR expression was analyzed by immunohistochemistry, whereas gene expression was evaluated by qRT-PCR.

**Results:** Immunohistochemistry revealed LEP and LEPR localized to the syncitiotrophoblast cells. In BeWo cells, we found a significant increase of leptin and leptin receptor mRNA levels at THC 40 μM. In placental explants, we did not observe the same pattern, as leptin levels were already increased at 10 μM.

**Conclusion:** In general, we demonstrate that THC, at high concentrations may lead to hormonal deregulation since it increases transcription of the genes encoding leptin and leptin receptor, which may impair placental development related to cannabis consumption.

**Acknowledgements**

Work financed by FEDER through COMPETE and FCT through PTDC/DTP-FTO/5651/2014-POCI-01-0145-FEDER-016562; FCT/MEC and FEDER, under PT2020 (UID/01/0145/FEDER/007728) and CCDR-N/NORTE2020/Portugal 2020 (norte-01-0145-FEDER-000024.

**PS188 Female reproductive aging: exploring the effects of antioxidant treatment**

Filipa Timóteo Ferreira^1,2,3^, Natalina Alexandra Rocha^3^, Sara Mendes^1,2,3^, Adriana Raquel Rodrigues^1,2,3^, ^4^Liliana Matos^1,2,3, 4,5^, Henrique Almeida^1,2,3,6^, Elisabete Silva^1,2,3^

^1^*Ageing and Stress group - Instituto de Biologia Celular e Molecular,*^2^*Instituto de Investigação e Inovação em Saúde,*^3^*Departamento de Biomedicina - Faculdade de Medicina da Universidade do Porto,*^4^*Faculdade de Ciências da Nutrição e Alimentação da Universidade do Porto,*^5^*Faculdade de Medicina Dentária da Universidade do Porto,*^6^*Serviço de Ginecologia e Obstetrícia - Hospital CUF do Porto E-mail address: filipatferreira1996@gmail.com (Filipa Timóteo Ferreira)*

**Introduction:** Aging has been linked with an intracellular imbalance between free radicals and antioxidant mechanisms that results in a phenomenon known as oxidative stress (OS). In the ovaries, OS is believed to be related to increased inflammation, fibrosis and tissue dysfunction.

**Aim:** This study aimed at evaluating whether these features are age-related and if specific antioxidant treatment with a NADPH-oxidase (NOX) inhibitor (apocynin) could ameliorate them.

**Methods:** Mice aged 11–15 weeks (young) or 39–41 weeks (aged) were employed. Aged mice were treated with apocynin (5 mM) in the drinking water during 8 weeks. Ovaries were collected and processed for histological and molecular studies. Haematoxylin & Eosin, Sudan black and picrosirius red (PSR) staining were used for histological examination and evaluation of ovarian fibrosis. NOX1 expression was assessed by immunohistochemistry. mRNA expression of collagen types, inflammation markers, matrix metalloproteinases (MMPs) and MMP tissue inhibitor (TIMP) 2 was determined by qPCR.

**Results:** Considering the features usually observed in the ovarian cortex and medulla, the structure of both mice groups was similar. However, a significant age-related decrement in the number of primordial and primary follicles was noticed. In addition, aged ovaries exhibited more cysts and lipofuscin deposition in stromal cells, together with enhanced fibrosis. Ovarian NOX1 expression was evidenced in oocytes and corpus luteum. Treatment of aged females with apocynin neither affect ovarian structure nor follicle number. However, it significantly reduced PSR staining. Aging significantly increased mRNA expression levels of collagen types (Col1a1 and Col5a1), inflammation markers (CCL5, TGF-b and IL-1b), MMP9 and TIMP2. Interestingly, apocynin normalized their expression levels. Col3a1, TNF-a, MMP2 and MMP12 were not affected by aging.

**Conclusion:** These findings indicate that OS and fibrosis are associated with increased inflammation and collagen deposition, likely contributing to age-related decline in female fertility. Apocynin treatment resulted in significant beneficial effects in inflammation and collagen deposition.

**PS194 PSMA- and PSCA-specific chimeric antigen receptors (CARs) for prostate cancer therapy**

Darya Matvienko^1,2^

^1^*Novosibirsk State University,*^2^*Institut of Molecular and Cellular Biology of the Siberian Branch of the Russian Academy of Sciences E-mail address: dacikmatvienko@gmail.com (Darya Matvienko)*

**Introduction:** T- and NK-cells armed with chimeric antigen receptors (CARs), are novel and promising instruments for cancer treatment. This therapy has been shown to be remarkably efficient for the treatment of several types of B-cell malignancies, however, its efficiency in solid cancers, such as prostate cancer (PC), still remains low.

**Aim:** Our work is aimed at comparing the in vitro performance of PSMA- and PSCA-specific CARs of various designs. Specifically, we explore the cytotoxicity and mediated by CARs having distinct organization of the hinge region.

**Methods:** We produced six NK-cell lines expressing PSCA- or PSMA-CARs. The CARs either had IgG1- or CD8a-derived hinge regions or were hingeless. Surface expression of CARs was verified by flow cytometry. Analysis of the cytotoxic activity was carried out by the incubation of effector cells (CAR NK-cells) with target cells (PC cells) followed by FACS analysis.

**Results:** All six cell lines obtained expressed CARs at comparable levels. For PSMA-specific CARs, the hinge region structure was crucial for cytotoxic activity in vitro: the highest level was observed for the hingeless version of the CAR. For PSCA-specific CARs, all hinge regions resulted in same level of CAR-driven cytotoxicity.

**Conclusion:** We have successfully generated six CAR-expressing NK-cell lines with PSMA or PSCA specificity. Our in vitro study indicates that hingeless design is the best option for PSMA specific CARcompared to conventional IgG1- and CD8a- based hinge regions. We attribute this to the bulky nature of PSMA target protein. PSCA-specific CAR did not display any hinge preferences. We believe that our work will be instrumental to advancing the therapeutic modalities for PC.

This study was supported by the grant #16-14-10237 (Russian Science Foundation).

**PS198 Insights of Hippo signaling pathway in chick lung branching**

Violina B Barbosa^1^, Jorge Correia-Pinto^1^, Rute S Moura^1^

^1^*Instituto de Ciências da Vida e da Saúde (ICVS); ICVS/3B's Laboratório Associado, Escola de Medicina, Universidade do Minho, Campus de Gualtar, 4710-057 Braga, Portugal E-mail address: violina.barbosa@gmail.com (Violina Baranauskaite Barbosa)*

**Introduction:** Hippo signaling pathway and its effector YAP has been recognized as important growth regulator during embryonic development. The inhibition of this pathway in the liver causes overgrowth, whereas in pancreas – hypoplasia, therefore suggesting phenotypic differences depending on the organ system.

**Aim:** Hippo signaling in lung organogenesis remains uncertain, this project aims to elucidate its role during pulmonary branching and, for the first time, in the avian animal model.

**Methods:** All experiments were conducted in embryonic chick lungs during early branching stages (b1, b2, b3: one, two or three secondary bronchi/bronchus, respectively). The spatial distribution of Hippo signaling members were characterized by in situ hybridization. Moreover, in vitro lung explant culture was performed in lung culture medium for 48 hours, and protein levels of phosphorylated-YAP (cytoplasmic)/YAP (nuclear) were assessed by Western blot at two time-points (0 and 48 hours). Additionally, lung explants were cultured in YAP-TEAD inhibitor verteporfin (5 or 7.5 μM) and vehicle control (DMSO) supplemented media and analyzed morphometrically (D2/D0 ratio).

**Results:** In situ hybridization revealed that all Hippo signaling members are mainly expressed in mesenchymal compartment, throughout early stages of chick lung branching, except for mst1 and lats2. Western blot analysis showed similar expression levels of both YAP and phosphorylated-YAP in the three stages studied. After 48 hours in culture, YAP and phosphorylated-YAP protein levels were slightly decreased when compared to 0 hours, nonetheless, the phosphorylated-YAP/YAP ratio was maintained. Lung explants treated with 7.5 μM verteporfin displayed a statistically significant overall reduction in lung size and branching when compared to controls.

**Conclusion:** This study demonstrates, for the first time, the presence of Hippo signaling in early stages of avian pulmonary branching. Gene/protein expression and pathway modulation studies indicate that Hippo is active and possibly involved in the regulation of lung growth which ultimately may contribute in designing novel therapies for various embryopathies.

**Acknowledgements**

This work has been funded by FEDER funds, through the Competitiveness Factors Operational Programme (COMPETE), and by National funds, through the Foundation for Science and Technology (FCT), under the scope of the Project POCI-01-0145-FEDER-007038; and by the Project NORTE-01-0145-FEDER-000013, supported by the Northern Portugal Regional Operational Programme (NORTE 2020), under the Portugal 2020 Partnership Agreement, through the European Regional Development Fund (FEDER).

**PS203 Synthesis of novel Ru (II) and Ru (III) salen and salan complexes and their cytotoxic effect in breast and colorectal cancer cell lines**

Patrícia Santos^1^, Dina Murtinho^1^, Salomé Pires^2,3,4^, Juliana Araújo^1^, Rosana Martins^5^, Margarida Abrantes^2,3,4^, Cristina Gonçalves^3,6^, Filomena Botelho^2,3,4^, Maria Elisa Serra^1^

^1^*Coimbra Chemistry Center and Department of Chemistry, University of Coimbra, Portugal,*^2^*Biophysics Institute, Faculty of Medicine, University of Coimbra, Portugal,*^3^*Institute for Clinical and Biomedical Research (iCBR) area of Environment Genetics and Oncobiology (CIMAGO), Faculty of Medicine, University of Coimbra, Portugal,*^4^*CNC.IBILI, University of Coimbra, Portugal,*^5^*Department of Biology, University of Aveiro, Portugal,*^6^*Oncobiology and Hematology Laboratory, Applied Molecular Biology and University Clinic of Hematology, Faculty of Medicine, University of Coimbra, Coimbra, Portugal E-mail address: tita_zona@live.com.pt (Patrícia dos Santos)*

**Introduction:** Breast (BC) and colorectal (CRC) cancer are responsible for a high mortality rate worldwide. The search for new anticancer drugs has increased in the last decades since chemotherapeutic drugs used nowadays show many adverse effects and cancer resistance. Previous studies have shown that metallic salen complexes exhibit antitumor activity. Ru complexes have revealed cytotoxic activity, proving greater selectivity for tumor cells. Their lower toxicity makes them a credible alternative to current chemotherapeutic drugs.

**Aim:** Synthesize four novel Ru(III) and Ru(II) chlorinated salen and salan complexes and test their cytotoxicity on BC and CRC cell lines.

**Methods:** Ru salen complexes were synthesized from camphoric acid derivatives and the respective salans were obtained by reduction of the salens. MCF-7 and HCC1806 BC cell lines and LS1034 and WiDr CRC cell lines, were exposed to the synthesized compounds. The effect on metabolic activity was evaluated by colorimetric MTT test. Cell viability and death, alterations on cell cycle and mitochondrial membrane potential (MMP) were assessed by flow cytometry, after exposure to the tetrachlorinated Ru(III) salen.

**Results:** All compounds induced a decrease in cell proliferation in a dose-dependent way. Relatively to the salen complexes, the tetrachlorinated Ru(III) complex presents greater cytotoxicity (IC50 < 4 μM) for all cell lines. Generally, salan complexes showed IC50 values lower than 12 □M in all cell lines, with better results in MCF-7 cells (IC50 < 5 μM). Tetrachlorinated Ru(III) salen induced a decrease of cell viability in all cell lines, with an increase of apoptosis/necrosis, associated to a decrease of MMP. This complex also induced cell cycle arrest on G0/G1 phase for MCF-7 cell line and S phase for CRC cells.

**Conclusion:** All compounds revealed dose-dependent anti-proliferative effects. Salan complexes have in general shown reduced metabolic activity. However, the tetrachlorinated Ru(III) salen complex was the most promising, exhibiting high anticancer activity in all cell lines.

**Acknowledgements**

Thanks are due to Coimbra Chemistry Centre (CQC), supported by the Portuguese Agency for Scientific Research, Fundação para a Ciência e a Tecnologia (FCT), through Project N007630 UID/ QUI/00313/2013, co-funded by COMPETE2020-UE and UC-NMR facility for NMR data (www.nmrccc.uc.pt). The authors would also like to thank FCT and COMPETE-FEDER for the Strategic Project CNC.IBILI: UID/NEU/04539/2013.

**PS205 Evaluation of changes in selected bone marrow stem and progenitor cells in pediatric patients with acute lymphoblastic leukemia in the course of ALLIC 2009 treatment protocol**

Aleksandra Basaj^1^, Anna Kre¸towska^1^, Małgorzata Kowalska^1^

^1^*Department of Regenerative Medicine and Immune Regulation, Faculty of Medicine, Medical University of Bialystok, Poland E-mail address: aleksandra.izabela.basaj@gmail.com (Aleksandra Basaj)*

**Introduction:** Very small embryonic-like stem cells (VSELs) are non-hematopoietic pluripotential cells serving as a reserve for tissue repair. Endothelial progenitor cells (EPCs) are progenitor cells with surface markers of endothelial vascular cells playing a crucial role in neovascularization. Despite their significant role in terms of regeneration, they have not yet been comprehensively studied in terms of acute lymphoblastic leukemia (ALL) – characterized by uncontrolled proliferation of cancerous lymphoblasts.

**Aim:** We aim to evaluate changes in frequency of VSELs, HSCs and EPCs in the bone marrow prior to and during the treatment of ALL pediatric patients to investigate their possible role in the disease.

**Methods:** The study was carried out in 30 patients with ALL and 35 controls. The bone marrow samples were derived from ALL patients at the time of diagnosis, after 33-rd day of treatment, at the beginning of M protocol, and from control group during diagnostic procedures. The samples were stained with fluorochrome-labeled monoclonal antibodies in order to distinguish HSCs, VSELs and EPCs. The data acquisition was performed using FACSCalibur flow cytometer.

**Results:** We observed that ALL patients demonstrate higher levels of VSELs (p = 0,0201) and EPCs (p = 0.0530) in the bone marrow compared to control group. During the treatment, marked decrease of the VSELs (p = 0,06) and increase of EPCs (p = 0.0666) has been noted. We did not find changes in HSC levels, however, analysis showed significant changes in HSC and VSEL distribution among stem cells leading to downward tendency of VSEL/HSC ratio in the course of ALL treatment (p = 0,005).

**Conclusion:** High VSELs and EPCs levels at the time of diagnosis, as well as their decreasing (VSELs) and increasing (EPCs) tendencies in the course of treatment may suggest potential contribution of these cells to ALL pathogenesis. Nevertheless, further investigation is required for detailed evaluation of VSELs and EPCs role in ALL.

**PS225 Cu(II) complexes derived from imidazole show cytotoxic activity in breast and colorectal cancers**

Juliana Gonçalves Araújo^1^, Maria Elisa Silva Serra^1^, Ana Salomé Pires^2,3,4^, Patrícia Santos^1^, Rosana Martins^5^, Ana Margarida Abrantes^2,3,4^, Ana Cristina Gonçalves^3,6^, Maria Filomena Botelho^2,3,4^, Dina Murtinho^1^

^1^*Centro de Química, Department of Chemistry, Faculty of Sciences and Technology, University of Coimbra, Portugal,*^2^*Biophysics Institute, Faculty of Medicine, University of Coimbra, Portugal,*^3^*Institute for Clinical and Biomedical Research (iCBR) area of Environment Genetics and Oncobiology (CIMAGO), Faculty of Medicine, University of Coimbra, Portugal,*^4^*CNC.IBILI, University of Coimbra, Portugal,*^5^*Department of Biology, University of Aveiro, Aveiro, Portugal,*^6^*Oncobiology and Hematology Laboratory, Applied Molecular Biology and University Clinic of Hematology, Faculty of Medicine, University of Coimbra, Coimbra, Portugal E-mail address: ju.araujo.23@hotmail.com (Juliana Gonçalves Araújo)*

**Introduction:** Despite the existence of new therapeutic options, breast (BC) and colorectal (CC) cancers remain the leading causes of cancer death and the most commonly diagnosed worldwide. Studies have reported that imidazole derivates show anticancer, antimicrobial, antibacterial, antifungal and antioxidant activities. Furthermore, recently it has been found that the association between imidazole ligands and copper increases their DNA binding affinity giving potential anticancer activity. Therefore, we synthesized novel Cu(II) complexes using heterocyclic imidazole derivatives as ligands.

**Aim:** Evaluate the cytotoxic activity of these complexes in two BC and two CC cell lines.

**Methods:** Three nitroimidazole-derived ligands, one benzimidazole-derived ligand and the respective Cu(II) complexes were synthesized and tested on MCF-7, HCC1806, LS1034 and WiDr cell lines. To evaluate their cytotoxic activity on four cell lines, MTT colorimetric assay was used. Subsequently, cell viability and death were evaluated by flow cytometry using annexin V and propidium iodide.

**Results:** Some of these Cu(II) complexes exhibit anticancer activity. The two imidazole-derived complexes containing cyclohexylamine show the lowest IC50 values in the four cell lines. The benzimidazole-derived complex containing cyclohexylamine presents the highest anti-proliferative effect (IC50 < 6.5 μM). The best IC50 value (1.2 μM) with this complex was obtained in HCC1806, a chemoresistant BC cell line. Curiously, the complex containing piperidine presents a IC50 of 3.8 μM in HCC1806 cell line, while in the other BC cell line (MCF-7) no anticancer activity is observed. Preliminary studies indicate that benzimidazole-derived complex also induces a decrease of cell viability in all cell lines.

**Conclusion:** Cu(II) complexes derived from imidazole presented anticancer activity against all cell lines. The benzimidazole-derived complex containing cyclohexylamine revealed to be the most promising, especially in HCC1806, basaloid triple-negative breast cancer, known as therapy-resistant. It is also noteworthy that the complex containing piperidine presented specificity for the chemoresistant HCC1806 cell line.

**Acknowledgements**

Thanks are due to Coimbra Chemistry Centre (CQC), supported by the Portuguese Agency for Scientific Research, Fundação para a Ciência e a Tecnologia (FCT), through Project N007630 UID/QUI/00313/2013, co-funded by COMPETE2020-EU and UC-NMR facility for NMR data (www.nmrccc.uc.pt). The authors would also like to thank FCT and COMPETE-FEDER for the Strategic Project CNC.IBILI: UID/NEU/04539/2013.

**PS226 Search for microRNAs as chemotherapy response biomarkers in non small-cell lung cancer. Preliminary studies**

Marcin Kaszkowiak^1^, Justyna Kiszałkiewicz^2^, Bartosz Szmyd^1^, Magda Baran'ska^1^, Sylwia De¸bska-Szmich^3^, Piotr Potemski^3^, Ewa Brzezian'ska-Lasota^2^

^1^*Student's Scientific Society of Genetics and Molecular Medicine, Department of Biomedicine and Genetics, Medical University of Lodz,*^2^*Department of Biomedicine and Genetics, Medical University of Lodz, Pomorska 251, 92–213, Lodz, Poland,*^3^*Department of Chemotherapy, Chair of Oncology, Medical University of Lodz, Poland; Copernicus Memorial Hospital in Lodz E-mail address: mgkaszkowiak@gmail.com (Marcin Kaszkowiak)*

**Introduction:** Lung cancer is currently the second most frequently diagnosed type of cancer worldwide[1]. The most significant problem in its treatment is chemotherapy or chemo-radiotherapy resistance of cancer cells[2]. A new approach to the classification of patients for chemotherapy is based on the genetic profile of cancer, the expression of certain genes or miRNAs. Moreover many studies have demonstrated the potential role of miRNA in overcoming resistance[3–5].

**Aim:** We have been investigating an impact of selected microRNAs (miR-21, miR-155, miR-330) expression on response to chemotherapy. Statistical analysis will be performed to construct a microRNA-based chemotherapy response predictor that may be found useful in the treatment planning.

**Methods:** The study contained 13 patients with diagnosed NSCLC and 12 healthy volunteers. The biological material was blood serum samples obtained on admission and after each cycle of chemotherapy. MicroRNA expression levels (in serum and exosomes) were evaluated using qPCR method with global normalization technique.

**Results:** In serum, we found statistically significant differences in microRNA expression between before- and after- chemotherapy. miR-21 expression was elevated (1.40 vs 12.53 respectively; p = 0.01, Wilcoxon test) and miR-330 expression was decreased (9000.75 vs 216.88 respectively; p = 0.03, Wilcoxon test). In exosomes, miR-21 expression was higher in NSCLC samples vs. control group both before (2.53 vs 0.66 respectively; p < 0.01, UMW test) and after (1.99 vs 0.66 respectively; p < 0.01, UMW test) chemotherapy. Comparing serum and exosomes, higher miR-330 expression in serum before (9000.75 vs 91.86 respectively; p < 0.01; Wilcoxon test) and higher miR-21 expression in serum after (12.53 vs 1.99 respectively; p = 0.01, Wilcoxon test) chemotherapy was revealed.

**Conclusion:** Changes of miR-21 and miR-330 expressions caused by chemotherapy suggest their involvement in response to the treatment. However, in research for miRNA's biomarkers an attention should be paid to variances in miR-21 and miR-330 expressions between serum and exosome samples.

**References**

1. R. L. Siegel, K. D. Miller, and A. Jemal, “Cancer statistics, 2018,” CA. Cancer J. Clin., vol. 68, no. 1, pp. 7–30, 2018.

2. P. Baxevanos and G. Mountzios, “Novel chemotherapy regimens for advanced lung cancer: have we reached a plateau?,” Ann. Transl. Med. Vol 6, No 8 (April 2018) Ann. Transl. Med. (Focus “Breakthroughs Treat. Adv. Lung Cancer Mak. Prog. Through Innov., 2018.

3. C. Rolfo et al., “Impact of microRNAs in Resistance to Chemotherapy and Novel Targeted Agents in Non-Small Cell Lung Cancer,” Current Pharmaceutical Biotechnology, vol. 15, no. 5. pp. 475–485, 2014.

4. L. MacDonagh, S. G. Gray, S. P. Finn, S. Cuffe, K. J. O’Byrne, and M. P. Barr, “The emerging role of microRNAs in resistance to lung cancer treatments,” Cancer Treat. Rev., vol. 41, no. 2, pp. 160–169, 2015.

5. E. Giovannetti, A. Erozenci, J. Smit, R. Danesi, and G. J. Peters, “Molecular mechanisms underlying the role of microRNAs (miRNAs) in anticancer drug resistance and implications for clinical practice,” Crit. Rev. Oncol. Hematol., vol. 81, no. 2, pp. 103–122, 2012.

**PS229 Cytotoxicity assessment of GuttaFlow**^**®**^** Bioseal**

Inês Ferreira^1,2^, Anabela Paula^1,2,3,4^, Mafalda Laranjo^2,3,4^, Filomena Botelho^2,3,4^, Manuel Marques Ferreira^1,3,4^

^1^*Dentistry Area, Faculty of Medicine, University of Coimbra,*^2^*Biophysics Institute, Faculty of Medicine, University of Coimbra,*^3^*iCBR, Coimbra Institute for Clinical and Biomedical Research, Faculty of Medicine, University of Coimbra,*^4^*CNC.IBILI, University of Coimbra E-mail address: ines17ferreira@gmail.com (Inês Oliveira Ferreira)*

**Introduction:** Endodontic treatment aims to prevent the presence of microorganisms and promote tissue repair through three-dimensional filling and sealing of root canal system. To promote healing and restoration of dental function, dental materials must be both restorative and biologically neutral. The cytotoxicity of material influences cell viability and can cause death. In order to maintain periapical tissue health after dental tissue changes, bioceramic cements have been developed, such as GuttaFlow^®^ Bioseal.

**Aim:** To evaluate cytotoxic effects of GuttaFlow^®^ Bioseal cement in the MDPC-23 cell culture.

**Methods:** The MDPC-23 mouse odontoblast-like cell line was cultured and plated. Cell culture media were conditioned during 24 h at 37°C with GuttaFlow^®^ Bioseal cement pellets. Cell cultures were incubated with the conditioned media and serial dilutions during 24, 72 and 120 hours. In order to evaluate metabolic activity and cell viability, the MTT and SRB assays were performed. Production of reactive oxygen species was evaluated by fluorimetry using the dihydroetidium and DCFDA probes.

**Results:** The cells exposed to GuttaFlow^®^ Bioseal conditioned media seems to be concentration and time-dependent. A decrease in the metabolic activity of MDPC-23 cells was observed when cells were exposed to the higher concentrations (100% and 50%), being more evident for longer incubation periods. However, maintenance of metabolic activity is ensured for lower concentrations (25% to 6,25%). Viability was maintained after incubation with the conditioned media in the concentrations of 25% and 6,25%, but a decrease of 70% was observed after exposure to the higher concentration. An imbalance in the production of peroxides and superoxide anion was observed.

**Conclusion:** In vitro cytotoxicity studies are essential to evaluate the safety of endodontic cements. GuttaFlow^®^ Bioseal cement showed some cytotoxicity depending on time and concentration, which might be related the production of ROS. These results encourage further studies to understand the influence of endodontic cements in the oral microenvironment.

**PS232 In silico drug-repurposing for augmenting K-Ras inhibitor treatment in pancreatic cancer**

Stan’czak Marcin^1^, Małachowska Beata^1^

^1^*Department of Biostatistics and Translational Medicine, Medical University of Lodz, Lodz, Poland E-mail address: marcin.stanczak@o2.pl (Marcin Stan*’*czak)*

**Introduction:** KRAS mutations are known to be an important factor in the pathogenesis of pancreatic adenocarcinoma. Our research, performed in collaboration with the Dana-Farber Cancer Institute, on potential selective inhibitors of K-Ras protein showed initial efficacy, which inexplicably declined over time.

**Aim:** Identification of intracellular changes indicating K-Ras inhibitor resistance and identification of substances that could potentially reverse them to make K-Ras inhibitor treatment effective.

**Methods:** Proteomics analysis was performed with tandem-mass-tagged mass spectrometry on MIA PaCa-2 cells cultured in 5 mM K-Ras inhibitors resolution for 1 hour, 4 hours, 24 hours, 72 hours, 7 days and 2 months and on cells incubated in dimethyl sulfoxide as a control. For identification of changes in expression of biologically-important groups of genes we performed Gene Set Enrichment Analyses [1]. Connectivity Maps were used to identify chemicals potentially capable of inverting undesired changes [2].

**Results:** A total of 17 significantly enriched gene sets (nominal p < 0.05) showed two interesting patterns of changes over time. In the first one (12 sets) genes responsible for protein were down-regulated for first 7 days of drug admission (NES < -1.3), but after 2 months of treatment became up-regulated (NES > 1.3). The other group consisted of 3 gene sets up-regulated in every measurement during the first week (NES > 1.4) and down-regulated after 2 months (NES < -1.8). Proteins that they code are constituents of respiratory chain. Connectivity Map, concerning changes in expression between 7 days and 2 months, showed strong negative connectivity with daunorubicin (tau = -98) and mitoxantrone (tau = -98).

**Conclusion:** Prolonged treatment with new K-Ras inhibitor led to selection of resistant clones. We identified changes in proteins reflecting dysregulation of genes’ expression, which can be associated with this phenomenon. They could potentially be reversed with topoisomerase inhibitors: daunorubicin and mitoxantrone.

**References**

1. Subramanian A. et al: Gene set enrichment analysis: A knowledge-based approach for interpreting genome-wide expression profiles, PNAS, 2005

2. Subramanian A. et al: A Next Generation Connectivity Map: L1000 Platform and the First 1,000,000 Profiles, Cell, 2017

**Acknowledgements**

Special thanks to Chandhoke Amrita Singh, Santana Naiara Codina and Mancias Joseph from Department of Radiation Oncology, Dana-Farber Cancer Institute, Boston, MA, USA

**PS234 Uterine protein oxidative modifications as a contributor to defective placentation**

Sara Mendes^1,2,3^, Filipa Ferreira Timóteo^1,2,3^, Adriana Raquel Rodrigues^1,2,3^, Liliana Matos^1,2,3,4,8^, Joaquim Saraiva^1,2,5,6^, Henrique Almeida^1,2,3,7^, Luís Guedes Martins^1,2,5^, Elisabete Silva^1,2,3^

^1^*I3 s – Instituto de Investigação e Inovação em Saúde, Universidade do Porto, Portugal,*^2^*Ageing and Stress Group, IBMC – Instituto de Biologia Celular e Molecular, Portugal,*^3^*Unidade de Biologia Experimental, Departamento de Biomedicina, Faculdade de Medicina da Universidade do Porto, Portugal,*^4^*Faculdade de Ciências da Nutrição e Alimentação da Universidade do Porto, Portugal,*^5^*Centro Hospitalar do Porto EPE, Portugal,*^6^*Trofa Saúde Hospital, Trofa, Portugal,*^7^*Serviço de Ginecologia e Obstetrícia, Hospital CUF, Porto, Portugal,*^8^*Faculdade de Medicina Dentária da Universidade do Porto, Portugal E-mail address: saramendes313@gmail.com (Sara Rafaela Silva Mendes)*

**Introduction:** Emerging work indicates that an oxidative uterine microenvironment at the time of implantation may condition placentation by interfering with extravillous trophoblast migration and invasion capacity.

**Aim:** In the present work we identified age-related protein oxidative modifications at placental site and used an in vitro extravillous trophoblasts cellular model to study their involvement in placentation.

**Methods:** Uterine samples were collected at delivery by elective caesarean section, which was approved by the ethical committee of “Centro Materno-Infantil do Porto”. Protein carbonylation was detected by oxyblot and specific protein carbonylation was verified by immunoprecipitation. Albumin expression and localization was determined by PCR and immunohistochemistry. HTR-8SV neo cell line was used as an in vitro model of first trimester extravillous trophoblast. The in vitro effect of carbonylated albumin on cell viability, proliferation and adhesion was quantified with neutral red. Scratch assay and collagen-coated transwells were used to evaluate its role in motility and invasion, respectively.

**Results:** A moderate correlation between total protein carbonylation and maternal age was observed at the placental site. A highly carbonylated 66 kDa protein (identified as albumin) showed strong positive correlation with maternal age (r = 0.5272; P = 0.0434). Moreover, carbonylated/total albumin ratio correlated strongly and significantly with maternal age (r = -0.6909; P = 0.0021). Albumin mRNA expression was found at the placental site. Immunohistochemistry showed that it features preferentially arteries and veins endothelium and connective tissue cells between muscle fascicles. In vitro results showed that carbonylated albumin (100 – 25 μg/ml) had no effect on cell viability and proliferation. Contrarily, at the highest concentration tested, cell motility was significantly reduced (24%). Blocking extracellular matrix collagen with carbonylated albumin (100 μg/ml) interfered significantly with trophoblast adhesion and invasion capacity (16% and 18%).

**Conclusion:** Maternal ageing is accompanied by selective oxidative modifications of uterine albumin. In vitro studies demonstrate that it has a deleterious role in trophoblast interaction with its environment.

**PS243 Differential effects of doxorubicin and mitoxantrone in brain glutathione levels of different aged mice**

Ana Carvalho^1^, Ana Reis-Mendes^1^, Margarida Duarte-Araújo^2^, Rita Guedes^1^, Salomé Monteiro^3^, Félix Carvalho^1^, Maria Lourdes Bastos^1^, João Paulo Capela^4^, Vera Marisa Costa^1^

^1^*UCIBIO, REQUIMTE, Laboratory of Toxicology, Department of Biological Sciences, Faculty of Pharmacy, University of Porto, Porto, Portugal,*^2^*Department of Imuno-Physiology and Pharmacology, Institute of Biomedical Sciences Abel Salazar, University of Porto, Portugal,*^3^*LAQV, REQUIMTE, Faculty of Pharmacy of University of Porto, Portugal,*^4^*FP-ENAS (Unidade de Investigação UFP em Energia, Ambiente e Saúde), CEBIMED (Centro de Estudos em Biomedicina), Faculdade de Ciências da Saúde, Universidade Fernando Pessoa, Porto, Portugal E-mail address: arcdc97@gmail.com (Ana Rita Dias Carvalho)*

**Introduction:** The long-term cognitive deficits that follow chemotherapy are generally referred to as “chemobrain”. Anticancer drugs like doxorubicin (DOX) and mitoxantrone (MTX) are used to treat a variety of cancers and their long-term toxicity has been proved in several organs, but data regarding their effects on the brain is still scarce.

**Aim:** This work aimed to evaluate the toxicity towards the brain of clinically relevant doses of DOX and MTX in male CD-1 mice of three different ages: infants (4 weeks), adults (3 months) and old (18–20 months).

**Methods:** All animals received biweekly intraperitoneal administrations, for 3 weeks. Control mice were injected with saline solution, MTX-treated groups received a total cumulative dose of 6 mg/kg in all groups, DOX-treated infant and adult groups received a total cumulative dose of 18 mg/kg and old mice received a total cumulative DOX dose of 9 mg/kg. During the experimental period, food and water intake, body weight and animal welfare were assessed daily. Mice were euthanized one week (adults and old animals) or seventeen days (infants) after the last injection. To evaluate the brain's oxidative stress, total glutathione (GSHt), reduced glutathione (GSH) and oxidized glutathione (GSSG) levels were determined, as well as the GSH/GSSG ratio.

**Results:** In adult and infant mice, DOX (18 mg/kg) caused weight decrease after the last injection. As early as day 10, these DOX groups revealed lower food intake than their respective controls. Brain levels of GSHt, GSH and GSH/GSSG ratio were decreased in DOX adults, but DOX infant brains had no changes in these parameters. Moreover, MTX did not cause significant changes in the brain glutathione levels in any of the groups tested.

**Conclusion:** This data suggest that DOX causes a decrease in mice body weight and significantly impairs the redox status of the adult brain, and therefore DOX neurotoxicity requires further research.

**Acknowledgements**

ARM and VMC acknowledge Fundação da Ciência e Tecnologia (FCT) for their grants (SFRH/BD/129359/2017 and SFRH/BPD/110001/2015, respectively). This work was supported by FEDER funds through the Operational Programme for Competitiveness Factors – COMPETE and by national funds by the Fundação para a Ciência e Tecnologia (FCT) within the project “PTDC/DTP-FTO/1489/2014 – POCI-01-0145-FEDER-016537”.

**PS246 Evaluation of (NH4)14(NaP5W30O110)•31H2O induced hepatotoxicity and nephrotoxicity**

Janko Zekovic’^1^, Milica Zdravkovic’^1^, Marko Dinčic’^2^, Mirjana B. Čolovic’^3^, Mila C’etkovic’^4^, Tamara Kravic’ Stevovic’^4^, Ali S. Mougharbel^5^, Urlich Kortz^5^, Danijela Krstic’^6^

^1^*Faculty of Medicine, University of Belgrade, Belgrade, Serbia,*^2^*Institute of Pathological Physiology, Faculty of Medicine, University of Belgrade, Serbia,*^3^*Department of Physical Chemistry, “Vinc̆a*” *Institute of Nuclear Sciences, University of Belgrade, Belgrade, Serbia,*^4^*Institute of Histology and Embryology, Faculty of Medicine, University of Belgrade, Serbia,*^5^*Department of Life Sciences and Chemistry, Jacobs University, Bremen, Germany,*^*6*^*Institute of Medical Chemistry, Faculty of Medicine, University of Belgrade, Belgrade, Serbia E-mail address: zekovic.janko@gmail.com (Janko Zekovic*’*)*

**Introduction:** Polyoxometalates (POMs) are negatively charged clusters containing transition metals in high oxidation states surrounded by oxygen atoms. Recent studies have shown their antiviral, antibiotic, antitumor and antidiabetic effects. However, their potential therapy application requires more detailed toxicological studies.

**Aim:** Evaluation of hepatotoxic and nephrotoxic effects of polyoxotungstate (NH4)14(NaP5W30O110)•31H2O were evaluated by a pathohistological analysis of liver and renal tissue of rats previously treated by this compound. Our preliminary results had demonstrate hypoglicemiant effect of this POM in rats with streptozotocin-induced diabetes.

**Methods:** This research was performed on adult Wistar male rats (n = 14). Animals were divided into two groups, the control (n = 7) and experimental group (n = 7). During 14 days, the experimental group was receiving daily a single dose of POM (20 mg/kg) per os. Afterwards the animals were sacrificed, and a histological analysis of liver and renal tissue was carried out using light and transmission electron microscopy.

**Results:** Light microscopy showed that glomerules and tubules of the experimental group did not differ from the control, but focal necrosis between the hepatic lobules was found in the experimental group. In addition, transmission electron microscopy did not show any ultrastructural alterations in kidney tubules, except for mitochondria which appeared to be dark and oedematous. Furthermore, in liver tissue there were small fields of coagulation necrosis between the hepatocytes accompanied with extravasated erythrocytes.

**Conclusion:** The study showed that (NH4)14(NaP5 W30O110)·_31_H_2_O treatment induced mild, ultrastructural mitochondrial alterations of the rat kidney as well as perilobular discrete focal necrosis of the liver tissue, so further toxicological researches are required.

**PS252 Adrenergic control of human macrophage polarization and its putative impact on cancer progression**

Diogo Adão^1,2^, Maria José Oliveira^2,3^, Jorge Gonçalves^1^

^1^*Faculdade de Farmácia da Universidade do Porto,*^2^*Instituto de Investigação e Inovação em Saúde- Universidade do Porto,*^3^*Instituto de Engenharia Biomédica; Faculdade de Medicina da Universidade do Porto E-mail address: diogo95sa@gmail.com (Diogo da Silva Adão)*

**Introduction:** An association between the nervous and the immune system has been recently described. Some evidence suggest that, a continued exposure to stress can result in release of neuroendocrine messengers, namely the catecholamines noradrenaline and adrenaline, which may promote tumorigenesis and affect the efficacy of cancer treatments (Janson, W. et al 2014). Noradrenaline and adrenaline exert their effects through activation of a family of G-protein coupled receptors, named adrenoceptors (AR). The b2-AR subtype has been particullary pointed as an immunodulating and tumorigenic receptor, because of its expression in several immune cell types, such as macrophages (Scanzano, A. et al 2015; Lattin, J. et al 2007). Macrophages are one of the most abundant cells in the tumour microenvironment and tumour-associated macrophages (TAMs) coordinate different stages of tumorigenesis, such as immunosuppression (Mantovani, A. et al 2006; Pinto, Marta L. et al 2017). The type of effects on tumours depends on the macrophage phenotype. Macrophages may act as tumour supressors (M1-like) or promotors (M2-like), depending on the stimuli they are exposed to (Cardoso, Ana P. et al 2015). Therefore, an adrenoceptor-mediated manipulation of macrophage phenotype may constitute an appealing alternative on immunotherapy of cancer.

**Aim:** In this study, we aimed to investigate how activation of b-AR influences the phenotype of activated human macrophages, to evaluate the potential of an interference with the adrenergic mechanisms in the modulation of tumour microenvironment.

**Methods:** Monocytes were isolated from healthy blood donors, provided by Hospital São João. Monocytes were plated and differentiated into macrophages and further stimulated with LPS or IL-10 towards a pro-(M1-like) or anti-inflammatory (M2-like) phenotype, respectively. Subsequently, these cells were treated with 100 nM Isoprenaline, a b-AR agonist. Expression of typical macrophage lineage and polarization markers was determined by flow cytometry of unstimulated, LPS- and IL-10 stimulated macrophages in the presence or absence of isoprenaline. Pro- and anti-Inflammatory cytokine production profile was measured by ELISA in conditioned media from distinct macrophage populations. Evaluation of macrophage morphology, cytoskeleton organization and expression b-AR was done by immunocytochemistry.

**Results:** Flow cytometry results obtained so far show that isoprenaline, incubated for 72 h, decreased CD86 expression (M1 marker) and increase CD163 expression (M2 marker). In order to confirm these results, pro- and anti-inflammatory cytokines profile was measured by ELISA. Interestingly, the levels of pro-inflammatory cytokines, namely IL-6, IL12/23p40 and TNF-a decreased in the presence of isoprenaline. Immunocytochemistry experiments confirmed the expression of b2-AR in human macrophages in different phenotypes. Macrophage morphology and cytoskeleton organization was evaluated by F-actin and a-tubulin staining. In six conditions analysed, macrophages presented a heterogeneous morphology. However a decrease in macrophage size was observed in presence of isoprenaline.Ongoing experiments aim to better characterize macrophage morphology. Their phagocytic activity, the transduction pathways involved in the b2-AR-mediated phenotype alterations and evaluate whether the adrenergic stimuli change the invasion capacity of cancer cells, and also if it alters the ability of macrophages to stimulate cancer cell invasion.

**Conclusion:** Altogether our data demonstrate that b2-adrenergic stimulation of human macrophages prevented polarization towards a proinflammatory phenotype, which consolidates previous data obtained in rodent models.

**References**

1. Cardoso, Ana P. et al., 2015. Matrix metalloproteases as maestros for the dual role of LPS- and IL-10-stimulated macrophages in cancer cell behaviour. BMC Cancer. 15:456

2. Janson, W. et al., 2014. A Nervous Tumor Microenvironment: The Impact of Adrenergic Stress on Cancer Cells, Immunosuppression, and Immunotherapeutic Response. Cancer Immunology, Immunotherapy. 63(11): 1115-1128.

3. Lattin, Jane. et al., 2007. G-protein-coupled receptor expression, function, and signalling in macrophages. Journal of Leukocyte Biology. Vol.82

4. Mantovani, A. et al., 2006. Role of tumor-associated macrophages in tumor progression and invasion. Cancer and Metastasis Reviews, 25(3)315–322.

5. Pinto, Marta L. et al., 2017. Decellularized human colorectal cancer matrices polarize macrophages towards an anti-inflammatory phenotype promoting cancer cell invasion via CCL18. Biomaterials 124. 211-224.

6. Scanzano, Angela. et al., 2015. Adrenergic regulation of innate immunity: a review. Frontiers in pharmacology.171

**PS254 Metabolism as a therapeutic target in acute myeloid leukemic – glycolysis or oxidative phosphorylation?**

Beatriz Lapa^1^, Joana Jorge^1,2^, Raquel Alves^1,2,3^, Ana Salomé Pires^3,4^, Ana Margarida Abrantes^3,4^, Margarida Coucelo^3,5^, Maria Filomena Botelho^3,4^, Ana Cristina Gonçalves^1,2,3^, Ana Bela Sarmento-Ribeiro^1,2,3,5^

^1^*Laboratory of Oncobiology and Hematology (LOH), University of Clinic of Hematology and Applied Molecular Biology, Faculty of Medicine, University of Coimbra, Coimbra, Portugal,*^2^*Coimbra Institute for Clinical and Biomedical Research (iCBR) - Group of Environment Genetics and Oncobiology (CIMAGO), Coimbra, Portugal,*^3^*Center for Neuroscience and Cell Biology (CNC.IBILI), University of Coimbra, Coimbra, Portugal,*^4^*Biophysics Unit, Faculty of Medicine, University of Coimbra, Coimbra, Portugal,*^5^*Clinical Hematology Service, University Hospital of Coimbra, Coimbra, Portugal E-mail address: bialapa_325@hotmail.com (Beatriz Santos Lapa)*

**Introduction:** Cancer cells undergo changes in their metabolic programs to maintain unregulated cellular proliferation and survival. These cells convert glucose to lactate regardless of oxygen availability, shifting from oxidative phosphorylation (OXPHOS) to glycolysis. Hence, this metabolic shift may be dependent on oncogenes expression and may constitute a new molecular target in acute myeloid leukemia (AML).

**Aim:** Our aim was to study the potential of glycolysis and OXPHOS metabolism as a therapeutic target in AML cell lines.

**Methods:** We used six AML cell lines (THP-1, KG-1, NB-4, HL-60, HEL and K-562). Cells were incubated in the presence or absence of 2-deoxy-d-glucose (2-DG; glycolysis inhibitor) or oligomycin (OXPHOS inhibitor). Metabolic activity was assessed by Resazurin Assay and glucose intake by 18F-FDG uptake. Sanger sequencing was used to evaluate IDH1/2 exon 4 mutations. PCR will also be used to analyze the expression of GLUTs (14 genes), hexokinase (4 genes) and HIF-1a.

**Results:** Preliminary results showed that 2-DG reduces metabolic activity in a time and dose dependent manner in all cell lines. Oligomycin reduces metabolic activity in time and cell line dependent manner. NB-4 cell line was the most affected by this treatment, suggesting the dependence on OXPHOS, whereas THP-1 appears to be the most resistant. 18F-FDG intake increased when cells were treated with oligomycin, showing a shift to glycolysis. As expected, 18F-FDG intake decreased when cells were treated with 2-DG. No mutations were found in IDH1/2 exon 4. Studies on gene expression are still ongoing.

**Conclusion:** All AML cell lines appear to be dependent on glycolysis, in particular THP-1 cells. However, NB-4 cells appear to be also OXPHOS dependent. Altogether these results suggest that cancer metabolism could be a therapeutic target in AML. However, the choosing between targeting glycolysis and/or OXPHOS may depend on genetic background.

Physiology & Immunology

**PS003 IL-17, IL-21 and IL-22 polymorphisms in rheumatoid arthritis: a systematic review and meta-analysis**

Inês Agonia^1^, Juliana Couras^1,2^, Anita Cunha^1^, Alda Andrade^1^, Juliana Macedo^1^, Bernardo Sousa-Pinto^3,4,5^

^1^*Faculty of Medicine of the University of Porto, Porto, Portugal,*^2^*Department of Physics, University of Aveiro, Aveiro, Portugal,*^3^*Basic and Clinical Immunology Unit, Department of Pathology, Faculty of Medicine, University of Porto, Porto, Portugal,*^4^*MEDCIDS—Department of Community Medicine, Information and Health Decision Sciences, Faculty of Medicine, University of Porto, Porto, Portugal,*^5^*CINTESIS—Center for Health Technology and Services Research, Porto, Portugal E-mail address: inesagoniaferreira@gmail.com (Maria Inês Agonia Ferreira)*

**Introduction:** Rheumatoid Arthritis (RA) is an inflammatory disease in whose pathogenesis participate several proinflammatory cytokines, including those produced by Th17 cells.

**Aim:** We performed a systematic review aiming to assess the roles of polymorphisms in Th17 cytokines - IL-17A, IL-17F, IL-21 and IL-22 - in the susceptibility and clinical characteristics of RA.

**Methods:** We searched in three electronic databases for observational studies assessing the association between susceptibility and clinical characteristics of RA and polymorphisms of the cytokines IL-17A, IL-17F, IL-21 and IL-22. From the selected studies, we extracted information on the studied polymorphisms, outcomes assessed, and frequencies and demographic characteristics of participants. We performed a random effects meta-analysis assessing the association between different genotypes of the IL-17A rs2275913 polymorphism and susceptibility to RA. Studies’ quality was assessed using Q-Genie tool.

**Results:** Thirteen studies were included in this systematic review. Five IL-17A polymorphisms were described to be associated with susceptibility to RA. In particular, for the rs2275913 polymorphism, our meta-analysis showed the AA genotype to be significantly associated with lower susceptibility to RA (OR = 0.76; 95%CI [0.61–0.93]; p = 0.008), while the opposite was observed for the GG genotype (OR = 1.18; 95% CI [1.02–1.36]; p = 0.03). Concerning Il-17F polymorphisms, rs763780 polymorphisms were described to be significantly associated with tender joints, creatinine levels and sedimentation rate. Finally, considering Il-21, five polymorphisms were explored, from which only rs6822844 and rs4505848 were described to have significant association with susceptibility to RA. No studies were found regarding IL-22 polymorphisms in RA.

**Conclusion:** In conclusion, our study showed that the IL-17A rs2275913 polymorphism is significantly associated with susceptibility to RA, while some IL-17 polymorphisms appear to be associated with different clinical characteristics of that disease. Nevertheless, regarding some polymorphisms, some contradictory evidence was found and, thus, further studies are needed to clarify the roles of Th17 cytokines’ polymorphisms in RA.

**Acknowledgements**

We thank Professor Luís Delgado (Basic and Clinical Immunology Unit, Department of Pathology, Faculty of Medicine, University of Porto) for his help and advice during the writing of this article.

**PS014 Analysis of the significance of the functional polymorphism Asp299Gly in Toll like receptor 4 gene in recurrent pregnancy losses**

Katarina Ivanovic’^1^, Stefan Ivanovic’^1^

^1^*Department of Human Genetics, School of Medicine, University of Belgrade E-mail address: ikatarina.1996@gmail.com (Katarina Ivanovic*’*)*

**Introduction:** Recurrent pregnancy loss (RPL) or habitual abortion involves three or more consecutive pregnancy losses, before 20 weeks, with the same partner. The incidence of recurrent pregnancy loss is 1 in 300 pregnancies, or 1% to 2% of women experience RPL. The occurrence of RPL is multifactorial. The most common causes are chromosomal abnormalities, uterine anatomic abnormalities, endocrine disorders, antiphospholipid antibody syndrome, thrombophilias, immunologic and environmental factors, infections.Within various immunological factors, one of the newest research subjects are polymorphisms in genes that control immune response. Such an example is polymorphism Asp299Gly in TLR4 receptor gene.

**Aim:** The aim of study is to examine the significance of polymorphism Asp299Gly in occurrence of RPL.

**Methods:** The study included 131 respondents, of whom 43 respondents didn’t have spontaneous abortions, while 88 subjects had three or more successive spontaneous abortions. Molecular-genetic analyzes included DNA isolation and genotyping of polymorphism Asp299Gly using RQ-PCR.

**Results:** The results indicate that AG genotype is predominant in experimental and control group, but there is difference among genotypes distribution (AG = 88.64%; AA = 10.23%; GG = 1.14% and AG = 60.47%; AA = 37.21%; GG = 2.33%). In control group, allele A is predominant (A = 0.67, G = 0.33), while in experimental, frequency of alleles is approximately the same, with allele A slightly more (A = 0.55, G = 0.45).

**Conclusion:** The study found that there is statistically significant difference in frequency of genotypes of polymorphism rs4986790, in subjects with RPL and control group (p < 0.001 ∗). Also, without Yates correction exists, while with Yates correction there isn’t statistically significant difference in frequency of alleles among subjects (p = 0.0464 ∗, p = 0.0632). Based on the results, it can be concluded that polymorphism rs4986790 is associated with occurrence of RPL, among our respondents.

**PS017 Relationship between insulin and insulin-like growth factor-1 in obesity**

Teodora Drazic^1^, Dunja Bjelogrlic^1^

^1^*Faculty of Medicine, University of Novi Sad E-mail address: drazicteodora@gmail.com (Teodora Drazic)*

**Introduction:** Insulin and insulin-like growth factor 1 (IGF-1) have the same origin, but their biological effects in homeostatic conditions are very different. Although insulin resistance, hyperinsulinemia and obesity are associated conditions, changes in the concentrations of IGF-1 could be dependable on multiple factors.

**Aim:** The aim of the study was to compare serum concentrations of insulin and IGF-1 between obese and lean subjects, and also to examine the possible associations between serum concentrations of insulin, IGF-1 and fatty mass of the subjects.

**Methods:** This study was conducted as a retrospective analysis of the medical data obtained from subjects who have been examined in the Center for laboratory medicine in Novi Sad. We used results of the biochemical findings and anthropometrical characteristics of the subjects.

**Results:** Obese subjects had (n = 50, median age-37.5; 30/50 female) a significantly higher concentrations of insulin, compared to the lean subjects (16.75 (11.9 – 24.5) vs. 6.05 (4.5 – 8.9) mIU/l, P < 0.0001). In obese group, serum concentrations of IGF-1 (94 (63 – 125) vs. 121.5 (106 – 146) mg/mL, P = 0.001) were significantly lower compared to lean group. In regression analysis, the fatty mass percentage was independently associated with the IGF-1 concentration (b = - 1.18; t = -2.98; P = 0.004).

**Conclusion:** In addition to significantly higher concentrations of insulin, obese subjects compared to normal weight subjects, have significantly lower serum concentrations of IGF-1. The concentrations of insulin and IGF-1 are negatively associated, and the fatty mass percentage is an independent predictor of the IGF-1 concentration, which points to a disorder of GH-IGF-1 axis in extreme obesity, which causes additional metabolic disorders in extreme obese people.

**PS018 Different low-level lead exposure profiles induce long-lasting physiological changes**

Liana Shvachiy^1^, Vera Geraldes^1,2^, Ângela Amaro-Leal^1^, Isabel Rocha^1,2^

^1^*Cardiovascular Autonomic Function Lab, Centro Cardiovascular da Universidade de Lisboa, Faculdade de Medicina, Universidade de Lisboa, Lisboa, Portugal, 2 Instituto de Fisiologia, Faculdade de Medicina, Universidade de Lisboa, Lisboa, Portugal E-mail address: shvachiy.liana@gmail.com (Liana Shvachiy)*

**Introduction:** Lead (Pb) is a toxic metal, and its widespread use has resulted in environmental contamination and significant public health problems. Exposures to lead (Pb) during developmental phases can alter the normal course of development, with lifelong health consequences. Permanent Pb exposure leads to behavioural changes, cognitive impairment, sympathoexcitation, tachycardia, hypertension and autonomic dysfunction. However, the effects of an intermittent lead exposure, increased in the past years, have not been studied.

**Aim:** To describe lead health effects along different profiles of lead exposure, including a new animal model of intermittent low-level lead exposure.

**Methods:** Foetuses were singly (PbS), intermittently (PbI) or permanently (PbP) exposed to water containing lead acetate (0.2% w/v) until adulthood (28 weeks of age). At 12, 20 and 28 weeks of age, blood pressure, electrocardiogram, heart rate and respiratory frequency were recorded, baroreflex gain and chemoreflex sensitivity calculated, plus determination of low frequencies (LF) and high frequencies (HF)for autonomic evaluation. An age- and sex-matched control group has been used. One-way ANOVA with Tukey's multiple comparison between means were used (significance p < 0.05) for statistical analysis.

**Results:** Our data showed a clear association between lead exposure, hypertension and cardio-respiratory reflexes impairment, without heart rate changes. At 28 weeks, PbI group, the new animal model of lead exposure, showed a less pronounced hypertension when compared to PbP group. Moreover, we showed that only a longer lead-free period is capable to reverse baroreflex impairment, without significant changes in chemoreflex function. Regarding the autonomic data, the overactivity of the sympathetic nervous system, evaluated by the LF band, is concomitant with baroreceptor reflex impairment and/or hypertension.

**Conclusion:** In summary, this study brings new insights on the environmental factors that influence nervous and cardiovascular systems during development, which can help creating public policy strategies to prevent and control the adverse effects of Pb toxicity.

**PS040 Characterization of the thyroid function of an animal model of heart failure with preserved ejection fraction and the impact of triiodothyronine supplementation on metabolic and cardiac function**

Catarina Vale^1^, João Sérgio Neves^1^, Madalena von Hafe^1^, Glória Conceição^1^, Dulce Fontoura^1^, Daniela Miranda-Silva^1^, Sara Leite^1^, Alexandre Gonçalves^1^, Soledad Bárez-López^2,3^, André P Lourenço^1^, Inês Falcão-Pires^1^, Adelino Leite-Moreira^1^

^1^*Departamento de Cirurgia e Fisiologia, Unidade de Investigação Cardiovascular, Faculdade de Medicina, Universidade do Porto, Porto, Portugal,*^2^*Department of Endocrine and Nervous System Pathophysiology, Instituto de Investigaciones Biomédicas Alberto Sols, Consejo Superior de Investigaciones Científicas (CSIC)-Universidad Autónoma de Madrid (UAM), Madrid, Spain,*^3^*Department of Endocrine, U-708, Center for Biomedical Research on Rare Diseases (Ciberer), Instituto de Salud Carlos III, Madrid, Spain E-mail address: catarinavale9@hotmail.com (Catarina Afonso Couto e Vale)*

**Introduction:** Heart failure with preserved ejection fraction (HFpEF) accounts for at least half the cases of heart failure and there is still no effective treatment for it. Thyroid hormones (TH) imbalance has been implicated in metabolic and cardiac dysfunction. However, the TH status in HFpEF and the effect of TH supplementation remain largely unknown.

**Aim:** The aim of this study is to characterize thyroid function and to evaluate the impact of triiodothyronine supplementation in an animal model of HFpEF.

**Methods:** Firstly, we compared the thyroid function of ZSF1 Obese rats (animal model of HFpEF with hypertension, dyslipidemia, obesity and diabetes) with that of ZSF1 Lean rats by measuring serum and tissue levels of T3 and T4 and serum levels of TSH. Then, we compared the metabolic and cardiac function of ZSF1Ob with that of ZSF1 Obese rats supplemented with T3 (ZSF1Ob + T3). Animals were weighted and submitted to insulin resistance and oral glucose tolerance testing, echocardiography, invasive hemodynamic evaluation and tissue collection. Single cardiomyocyte sarcomere shortening was monitored and cytosolic Ca2 + transients were recorded.

**Results:** Compared to ZSF1Ln, ZSF1Ob presented with a significant decrease of serum and left ventricle levels of TH. Visceral adipose tissue TH levels and TSH serum levels were not significantly different. ZSF1Ob + T3 showed significantly lower body, liver, perigonadal fat and perirenal fat weights and improved glucose metabolism and insulin sensitivity compared to ZSF1Ob. Echocardiographic and hemodynamic evaluation showed improved diastolic and systolic function in supplemented rats. This was further supported by an improved Ca2 + and sarcomere relaxation and cardiomyocyte contractility. Lastly, treatment with T3 returned the resting sarcomere length to a more physiological range and improved contractile response to Ca2 + transients.

**Conclusion:** In conclusion, HFpEF presents with local and systemic hypothyroidism. Triiodothyronine supplementation improves cardiac and metabolic function of ZSF1Ob rats. Thyroid hormones may be an effective therapeutic target for HFpEF.

**PS074 Values of blood pressure, lung function and muscular strength in students with hypermobility**

Nikola Bakic’^1^, Aleksandra Rakovac^1^

^1^*Department of Physiology, Faculty of Medicine Novi Sad, University of Novi Sad E-mail address: bakicnikola44@gmail.com (Nikola Bakic*’*)*

**Introduction:** Elastin and collagen are the most important components of bones, tendons, skin, lung tissue and the arterial walls. Weak connective tissue is a pathological state with changed structure of collagen fibers, which leads to a number of symptoms.

**Aim:** The goal was to determine the prevalence of weak connective tissue in students aged 20.63 ± 0.77 from Faculty of Medicine University of Novi Sad, and to compare values of blood pressure, lung function and muscle strength between students with weak and normal connective tissue.

**Methods:** The study included 100 healthy physically non active students (50 female and 50 male) divided into two groups: students with weak and students with normal connective tissue. Diagnosis of hypermobility was confirmed using Brighton score and the Beighton criteria. Anthropometric parameters, blood pressure, lung function and muscle strength of upper and lower extremities were measured. Student‘s t-test was used to determine the differences between groups. Statistically significant difference was set at p ≤ 0.05.

**Results:** Significant differences were noted between tested groups in values of body height (p = 0.014), body weight (p = 0.021), systolic (p < 0.001) and diastolic (p = 0.004) blood pressure. Parameters of lung function were also significantly different between groups regarding to VC (p < 0.001), FVC (p = 0.05) and FEV1 (p = 0.25). When analyzing the values of dynamometric parameters between groups a significant difference was also observed.

**Conclusion:** High percentage (67%) of medical students of University of Novi Sad have weak connective tissue. Students with weak connective tissue have significantly lower values of blood pressure, lung function and dynamometric parameters in comparison to students with normal connective tissue.

**PS075 Innate immune response against plasmodium liver stage parasites is enhanced by targeted nutritional supplementation**

Daniela Henriques Brás^1^

^1^*Instituto de Medicina Molecular; Instituto de Higiene e Medicina Tropical E-mail address: nihenriques@gmail.com (Daniela Cristina de Henriques Brás)*

**Introduction:** During the asymptomatic liver stage of malaria infection, Plasmodium parasites scavenge host nutrients to support their multiplication[1]. One of these nutrients is arginine (Arg), whose metabolism is crucial for the parasite's intra-hepatic development[1].

Arg is the only amino acid-based supplementation evaluated in the context of malaria. However, although Arg supplementation can enhance nitric oxide (NO) production and improve survival in animal models of Plasmodium infection, results obtained in the clinic are unclear[2–4].

**Aim:** Preliminary results from the host laboratory have shown that supplementation of C57BI/6J mice with RKV, which combines Arg (R) with Lysine (K) and Valine (V), two amino acids described as inhibitors of the arginase enzymes[5], leads to a striking decrease of hepatic infection by the rodent malaria parasite P. berghei. Thus, our aim was to elucidate the mechanism of hepatic parasite elimination upon RKV supplementation.

**Methods:** Plasmodium liver infection was characterized employing real-time PCR and immunofluorescence microscopy. The impact of RKV supplementation on the immune system was ascertained employing knockout mice and depleting specific immune cell populations.

**Results:** The decrease in Plasmodium liver infection upon RKV supplementation results mostly from a reduction in the number of infected hepatocytes, supporting a role of the host's immune system on parasite elimination. Parasite elimination does not rely on NO production nor on a boost of the Type-I IFN response, previously reported as crucial to control liver stage infection[6,7]. Natural Killer (NK) cells were identified as the effector cells involved in RKV-dependent parasite elimination. Signaling via Myd88, seems to also be essential for this elimination process, although the cells in which this signaling occurs remain unidentified.

**Conclusion:** We found that NK cells, commonly used in immunotherapies against cancer and viral infections[8], can also be stimulated to act against Plasmodium liver stage infection, making these immune cells an appealing target for new antimalarial strategies.

**References**

1. Meireles P, Mendes AM, Aroeira RI, et al. Uptake and metabolism of arginine impact Plasmodium development in the liver. 2017;(September 2016):1–12. doi:10.1038/s41598–017-04424-y.

2. Martins YC, Zanini GM, Frangos JA, Carvalho LJM. Efficacy of different nitric oxide-based strategies in preventing experimental cerebral malaria by Plasmodium berghei ANKA. PLoS One. 2012;7(2). doi:10.1371/journal.pone.0032048.

3. Zhu X, Pan Y, Li Y, Cui L, Cao Y. Supplement of L-Arg improves protective immunity during early-stage Plasmodium yoelii 17XL infection. Parasite Immunol. 2012;34(8-9):412-420. doi:10.1111/j.1365-3024.2012.01374.x.

4. Appleton J. Arginine: Clinical potential of a semi-essential amino acid. Altern Med Rev. 2002;7(6):512-522.

5. Downs AH and CE. The inhibition of arginase by amino acids. J Biol Chem. 1945;(157):427-446.

6. Miller JL, Sack BK, Baldwin M, Vaughan AM, Kappe SHI. Interferon-Mediated Innate Immune Responses against Malaria Parasite Liver Stages. Cell Rep. 2014;7(2):436-447. doi:10.1016/j.celrep.2014.03.018.

7. Cabello CM, Bair WB, Lamore SD, et al. Host-cell sensors for Plasmodium activate innate immunity against liver-stage infection. Nat Med. 2010;46(2):220-231. doi: 10.1038/nm.3424.

8. Poli A, Michel T, Patil N, Zimmer J. Revisiting the Functional Impact of NK Cells. Trends Immunol. 2018;xx:1-13. doi:10.1016/j.it.2018.01.011.

**Acknowledgements**

Agradeço a toda a minha equipa MPrudêncio Lab do IMM, e em especial à minha orientadora Dra. Patrícia Meireles e ao meu co-orientador Dr. Miguel Prudêncio.

**PS078 Effects of silica-rich water intake on the systemic and peritoneal inflammation in rats exposed to chronic low level (900** **MHz) microwave radiation**

Vladana Stankovic^1^, Andjela Zovkic^1^

^1^*Institute for Pathophysiology, Faculty of Medicine, University of Nis, Nis, Serbia E-mail address: vladanas.14@gmail.com (Vladana Stankovic’)*

**Introduction:** Microwave (MW) exposures from mobile phones are a possible cause of immunocompetent cells number and activity alterations. Additionally, water soluble forms of silica or silicon dioxide (SiO2) found in the drinking water showed nonspecific immunostimulating effects and increased phagocytic activity in animals. Peritoneal macrophages (PMs), as a part of intestinal barrier, might have a role in autoimmune and proinflammatory disorders under the influence of MW radiation and silica-rich water.

**Aim:** The study evaluated the effect of silica-rich water intake on systemic inflammation and functional characteristics of peritoneal macrophages (PMs) in rats chronically exposed to low level of microwave radiation.

**Methods:** Wistar Albino rats were exposed to 900 MHz MW from mobile phone, during 3 months. The four-treatment arms model involved rats that received standard water (SW; 6 mg/L SiO2) and experimental groups with silica-rich water (EW; 19 mg/L SiO2). PMs were harvested by peritoneal lavage and divided in unstimulated and lipopolysaccharide stimulated subgroups. The serum and culture medium were subjected to ELISA analyses of different cytokines. The phagocytic ability was assessed by measuring Neutral Red uptake.

**Results:** The MW exposed rats with silica-rich water intake (MW + EW) had significantly lower serum TNF-α and IL-2 levels, but higher IL-10 levels, than the MW + SW rats, while soluble ICAM-1 and VCAM-1 were not changed. Phagocytic activity of LPS-stimulated PMs was preserved in MW + EW, unlike MW + SW group. The unstimulated MW exposed PMs from MW + EW group had lower TNF-α levels than MW + SW (p < 0.01), but all PMs produced significantly more IL-10 regardless of the water type. The LPS-stimulated MW exposed PMs released significantly less TNF-α than their matched sham controls, while MW + EW group produced more IL-10 than the MW + SW (p < 0.05).

**Conclusion:** Taken together, silica-rich water seems to prevent MW-induced systemic and peritoneal inflammation, reflecting its ability to shape monocyte plasticity, thereby altering the balance between their proinflammatory and anti-inflammatory properties.

**PS097 Exhaled breath condensate microRNAs as potential biomarkers to identify and endotype asthma in school-aged children**

João Cavaleiro Rufo^1,2,4,5^, Diana Silva^1,2^, Pedro Cunha^10^, Mariana Farraia^10,11^, Pedro Moreira^5,10^, Luís Delgado^1,2^, Miguel Soares^1,7,8,9^, André Moreira^1,2,5,10^

^1^*Faculdade de Medicina da Universidade do Porto, Porto, Portugal,*^2^*Centro Hospitalar São João, Porto, Portugal,*^3^*Faculdade de Ciências da Universidade do Porto,*^4^*Institute of Science and Innovation in Mechanical Engineering and Industrial Management (INEGI), Porto, Portugal,*^5^*EPIUnit - Instituto de Saúde Pública, Universidade do Porto, Porto, Portugal,*^6^*Instituto de Ciências Biomédicas Abel Salazar Universidade do Porto, Porto, Portugal,*^7^*Departamento de Biomedicina-Unidade de Biologia Experimental, Centro de Investigação Médica (CIM), Faculdade de Medicina da Universidade do Porto,*^8^*Pain Group, Instituto de Biologia Molecular e Celular (IBMC); Instituto de Investigação e Inovação em Saúde, Universidade do Porto,*^9^*Laboratório de Apoio à Investigação em Medicina Molecular (LAIMM), Faculdade de Medicina da Universidade do Porto, Porto, Portugal,*^1^^*0*^*Faculdade de Ciências da Nutrição e Alimentação da Universidade do Porto, Porto, Portugal,*^11^*Universidade de Aveiro E-mail address: francisca_castromendes@hotmail.com (Ana Francisca Soares de Castro Mendes)*

**Introduction:** MicroRNAs (miRNAs) are small non-coding RNAs that modulate almost all biological processes. As such, miRNA profiling in disease states constitutes a powerful tool of diagnostic and prognostic value. Detection and quantification of miRNAs in exhaled breath condensate (EBC) has been poorly explored.

**Aim:** We aimed to assess miRNAs in EBC as potential biomarkers to diagnose and endotype asthma in schoolchildren.

**Methods:** In a cross sectional, nested case control study, all the asthmatic children (n = 65) and a random sample of controls (n = 121), aged 7 to 12 years, attending 71 classrooms from 20 local schools were selected. Participants underwent skin-prick testing, spirometry with bronchodilation, had exhaled level of nitric oxide determined and EBC collected. Based on previous studies ten miRNAs were chosen and analyzed in EBC by reverse transcription-quantitative real-time PCR. Asthma was defined based on positive bronchodilation or medical diagnosis with reported symptoms in the past 12 months. Factor analysis and generalized linear models were applied to identify miRNA profiles and their associations with asthma and their features.

**Results:** Exhaled breath condensate miR-126-3p and miR-133a-3p were associated with asthma and with positive bronchodilation without symptoms, respectively. Levels of miR-126-3p and miR-146a-5p were associated with higher small airways response after bronchodilation while levels of miR-328-3p were associated with lower FEV1 response after bronchodilation. Associations were found between miR-126-3p and miR-328-3p with exhaled NO and between miR221-3p and breathing difficulties.

**Conclusion:** These results showed that miRNAs can be measured in EBC of schoolchildren, providing further support for the possibility of using microRNAs as biomarkers of asthma and associated lung function impairment. Further, miRNAs may assist asthma endotype establishment on the way to a more personalized treatment.

**PS102 MicroRNA-146a role in the pathophysiology of pulmonary arterial hypertension**

Luís Pimentel^1^, Pedro Mendes-Ferreira^1^, Diana Santos-Ribeiro^1^, Rui Adão^1^, Carolina Maia-Rocha^1^, Cláudia Monteiro-Pinto^1^, Steeve Provencher^2^, Sébastien Bonnet^2^, Adelino Leite-Moreira^1^, Carmen Brás-Silva^1,3^

^1^*Department of Surgery and Physiology, UnIC - Cardiovascular Research Centre, Faculty of Medicine, University of Porto, Portugal,*^2^*Pulmonary Hypertension Research Group, Institut Universitaire de Cardiologie et de Pneumologie de Québec, Laval University, Quebec City, Canada,*^3^*Faculty of Nutrition and Food Sciences, University of Porto, Portugal E-mail address: luispimentel.med@gmail.com (Luís Daniel Ribeiro Pimentel)*

**Introduction:** Pulmonary arterial hypertension (PAH) is characterized by excessive pulmonary vascular remodelling, resulting in elevated pulmonary vascular resistances and right ventricle (RV) overload and failure. PAH remains incurable, and new therapeutic approaches are required. MicroRNA (miR)-146a promotes vascular smooth muscle cell proliferation and vascular neointimal hyperplasia, both important hallmarks of PAH. Additionally, MiR-146a represses several signalling pathways which could play a major role in PAH.

**Aim:** To investigate MicroRNA-146a role in the pathophysiology of PAH and RV heart failure (HF).

**Methods:** Human RV samples were obtained from autopsies, heart transplantation or cardiac surgery, and categorized as normal RV (NRV), compensated RV hypertrophy (CRV) and decompensated RVHF (DRV). Lung tissue samples were collected from explanted lungs or during lung resection from healthy segments. Pulmonary artery smooth muscle cells (PASMC) were isolated from PAH patients and treated with miR-146a inhibitor. Wild-type (WT) and miR-146a knock-out (miR-146a-/-) mice were submitted to either 3 weeks of chronic hypoxia-induced PAH with weekly Sugen 5416 administration (SuHx) or pulmonary artery banding (PAB)-induced RV hypertrophy.

**Results:** MiR-146a expression was increased in the RV of patients with DRV when compared to NRV. This increase was inversely correlated with decreased RV function. Lung tissue from PAH patients showed an increase in miR-146a levels when compared to controls, and the same was observed in isolated PASMC. PAH-PASMC treated with miR-146a inhibitor showed decreased proliferation and increased apoptosis compared to non-treated cells. MiR-146a-/- mice developed significantly decreased RV hypertrophy in both SuHx and PAB models, and showed decreased RV dilation secondary to PAB.

**Conclusion:** MiR-146a expression is increased in both the RV and Lung of PAH patients, and its deletion in animal models results in decreased RV hypertrophy, suggesting that this miRNA plays an important role in the pathophysiology of PAH and might be a therapeutic target in this condition.

**PS106 Optimization of a synaptosomal model to study the effect of carotenoids in peroxynitrite - induced lipid peroxidation**

Pedro Nicola^1^, Marisa Freitas^1^, Sónia Rocha^1^, Félix Carvalho^2^, Eduarda Fernandes^1^, Daniela Ribeiro^1^

^1^*LAQV, REQUIMTE, Laboratory of Applied Chemistry, Department of Chemical Sciences, Faculty of Pharmacy, University of Porto, 4050-313 Porto, Portugal,*^*2*^*UCIBIO, REQUIMTE, Laboratory of Toxicology, Department of Biological Sciences, Faculty of Pharmacy, University of Porto, 4050-313 Porto, Portugal E-mail address: up201403029@fc.up.pt (Pedro Daniel Maia Nicola)*

**Introduction:** Carotenoids are lipophilic antioxidants, considered to be determinant contributors for a good health status, as they play an essential role in the maintenance of redox homeostasis in the lipid environment, and contribute for a decreased risk of developing various chronic degenerative diseases, such as cancer, inflammation, and others. However, antioxidant or pro-oxidant behaviour of carotenoids and respective metabolites, and their possible consequences to our health are not yet fully clarified, especially due to the lack of effective in vitro models. Lipids cover about 60% of the brain's dry weight, making brain tissue a good model basis for the evaluation of the antioxidant effect of lipophilic compounds, in conditions close to a real scenario environment.

**Aim:** This work aims to explore the antioxidant versus pro-oxidant effects of β-carotene and its metabolite, apocarotenal, in a rat synaptosomal model.

**Methods:** A micro-analysis assay, using synaptosomes, was developed to enable the high-throughput screening of the putative inhibitory effect of carotenoids, against peroxynitrite (ONOO-) - induced lipid peroxidation. Synaptosomes were isolated from rat brain as previously described (1) and the compounds to be tested were incorporated during the synaptosomes preparation procedure. After the optimization process, the optimal experimental conditions for the assay were found: synaptosomes (0.1 mg protein/mL) were incubated with ONOO- (10 mM), for 15 min, at 37°C. In sequence, lipid peroxidation was evaluated by the thiobarbituric acid reactive substances (TBARS) assay.

**Results:** The studied carotenoids, at the tested concentrations, demonstrated a slight inhibition of the lipid peroxidation.

**Conclusion:** The obtained results confirm the uncertainties about carotenoids’ great potential as antioxidants. The experimental model developed may constitute an useful research tool as the lipophilic compounds are incorporated during the brain homogenization step, avoiding solubility problems that often appear in other in vitro oxidative stress models.

**References**

1. Barbosa, D.; Capela, J.; Oliveira, J.; Silva, R.; Ferreira, L.; Siopa, F.; Branco, P.; Fernandes E.; Duarte, J.; Maria de Lourdes Bastos, M.; Carvalho, F, Br. J. Pharmacol. 2012, 165, 1017-1033.

**Acknowledgements**

This work received financial support from the European Union (FEDER funds POCI/01/0145/FEDER/007265) and National Funds (FCT/MEC, Fundação para a Ciência e Tecnologia and Ministério da Educação e Ciência) under the Partnership Agreement PT2020 UID/QUI/50006/2013, and “Programa Operacional Competitividade e Internacionalização” (COMPETE) (PTDC/QEQ-QAN/1742/2014 – POCI-01-0145-FEDER-016530), and under the framework of QREN (NORTE-01-0145-FEDER-000024).

**PS115 Thrombolytic activity of extracellular proteases from new strains of mycromycetes**

Elizaveta Rukavitsyna^1^, Elena Kornienko^1^, Aleksander Osmolovskiy^1^

^1^*Lomonosov Moscow State University E-mail address: hoppahm@gmail.com (Elizaveta Rukavitsyna)*

**Introduction:** Current medicine is looking for high effective thrombolytic enzymes. One of the promising sources of such enzymes are microorganisms and in particular micromycetes. Undoubted interest propose fibrinolytic proteases isolated from *Aspergillus flavipes, A. ochraceus, A. sydowii and Sarocladium strictum* [1,2].

**Aim:** Determination of effectiveness of proteases from *Aspergillus flavipes, Aspergillus ochraceus, Aspergillus sydowii* and *Sarocladium strictum* to hydrolyse trombi in vitro

**Methods:** The preparation of isolated enzymes of micromycetes was added to the freshly prepared thrombi, mixture was incubated at 37°C for 30–120 minutes. To obtain the thrombus a solution of 0.1% thrombin was added to human blood plasma. The lysis efficiency of proteases of micromycetes was expressed as a percentage, measuring the primary and final thrombus mass [3].

**Results:** The efficiency of thrombus hydrolysis by proteases of micromycetes in vitro shows, that proteolytic enzymes of *Aspergillus flavipes* are able to lyse the thrombus for 70% in 120 min, of *A. ochraceus* for 94% and *A.sydowii* for 97% at the same time. Proteolytic enzymes of *Sarocladium strictum* can lyse thrombus for 60% in 90 min.

**Conclusion:** Thus, it can be concluded that the proteolytic enzymes secreted by the micromycetes *Aspergillus flavipes*, *A. ochraceus, A. sydowii* and *Sarocladium strictum* have a significant efficacy of thrombolytic action. The results obtained in the course of the experiment are comparable with the effectiveness of the pulmonary embolism, which was shown for streptokinase, which are a rabbit antibiotic obtained from streptococci [4].

**References**

1. T.S. Sharkova, A.V. Kurakov, A.A.Osmolovskiy, E.O.Matveeva, V.G.Kreyer, N.A.Baranova, N.S.Egorov, 2015, Screening of producers of proteinases with fibrinolytic and collagenolytic activities among micromycetes, Microbiology, Vol. 84, No. 3, pp. 359-364.

2. A.A.Osmolovskiy, E.D.Rukavitsyna, V.G.Kreier, N.A.Baranova, N.S.Egorov, 2017, Production of proteinases with fibrinolytic and fibrinogenolytic activity by a micromycete Aspergillus ochraceus, Microbiology, Vol.86, No.4, pp 512-516.

3. Kotb E., 2012. Fibronolytic bacterial enzymes with thrombolytic activity. Springer Briefs in Microbiology, 74 p.

4. Prasad S. et al. Development of an in vitro model to study clot lysis activity of thrombolytic drugs // Thrombosis Journal. 2006. V. 4. No. 1. P. 14.

**PS146 Acute endothelial and angiogenic response to restricted blood flow exercise with cooling in healthy volunteers – the pilot study**

Martyna Schönborn^1^, Agnieszka Trynkiewicz^1^, Małgorzata Ceben’ko^1^, Mikołaj Maga^1^

^1^*Department of Angiology, Jagiellonian University Medical College E-mail address: martyna.schonborn@gmail.com (Martyna Nadzieja Schönborn)*

**Introduction:** Peripheral artery occlusive disease (PAOD) is a serious health issue that affects 20% of Europeans above 55 years old. However, treatment methods of patients with PAOD, especially those with non-critical lower limbs ischemia are still a matter of debate. One of the most important non-invasive form of treatment – physical training – could be modified by adding vein occlusion and cooling, but its acute influence on endothelium function and angiogenesis has never been explored yet.

**Aim:** The aim of this study was to evaluate acute endothelial and angiogenic response to physical training with restricted blood flow and cooling among healthy volunteers.

**Methods:** 35 healthy volunteers (age 24,8 ± 2,6; 51,4% males, height 174,7 cm ± 9,4, weight 68,3 kg ± 13,6) completed a 21-minutes interval training on NuStep T5XR with vein occlusion performed by cooling liquid pressure cuffs (arm cuffs with 40 mmHg and leg cuffs with 65 mmHg) and cooling seat. Endothelium functions and angiogenic processes were monitored with laboratory parameters - vascular endothelial growth factor (VEGF), clusters of differentiation (CD31, CD34) and imaging examinations - stiffness index (SI), reflexion index (RI), reactive hyperaemia index (RHI) and augmentation index (AI). Blood samples were collected and imaging examinations were performed prior as well as 20–30 minutes after the exercise.

**Results:** All laboratory parameters were significantly higher after the training – CD34 (582 ± 85.1 vs 138.7 ± 35.7, p < 0,0001), CD31 (286.3 ± 96.2 vs 102.8 ± 28.3, p = 0,0012) and VEGF (126.1 ± 11.9 vs 28.6 ± 5.8, p < 0,0001). Moreover, physical training resulted in RI decline (69,52 vs 65,37, p = 0,013) and SI decline (7,60 vs 7,121 p = 0,024). RHI and AI were uninfluenced by exercise.

**Conclusion:** Our results suggest an important acute angiogenic response to restricted blood flow exercise with cooling, showing also an influence on RI and AI values. It is the pilot study and the results will be implemented into larger research project including patients with PAOD to confirm these findings.

**PS154 Mechanism of methotrexate's cytotoxicity, in vitro**

Mario Robert Balo^1^

^1^*Faculty of Medicine, University of Belgrade E-mail address: balo.mario15@gmail.com (Mario Balo)*

**Introduction:** Since mid-twentieth century, methotrexate (MTX) has been used as an antifolate agent in the treatment of various cancers, including acute lymphoblastic leukemia. Despite being used in clinical practice for such a long time, its underlying molecular mechanisms of action and cell death induction especially of cancer cells, still haven’t been fully understood and explained.

**Aim:** The aim of this study was to determine the mechanisms of methotrexate's cytotoxicity in vitro.

**Methods:** We used acute lymphoblastic leukemia (MOLT-4) and human lung fibroblast (MRC-5) cell lines. The viability of the cells treated with MTX was assessed by measuring the activity of intracellular acid phosphatase, while morphology of the cells was determined by light microscopy. The mechanisms of cell death were analyzed using flow cytometry of the cells stained with fluorescein isothiocyanate conjugated pan-caspase inhibitor and acridine orange for the detection of caspase activity and acidic vesicles cytoplasm content, respectively.

**Results:** MTX increased caspase activity in MOLT-4 cells after 24 h and decreased their viability after 48 h. But it did not cause the acidification of MOLT-4 cytoplasm. MTX decreased the viability of MRC-5 cells after 24 h, but after 72 h the viability was significantly greater than of leukemic cells under the same experimental conditions. The amount of intracellular acidic vesicles content in MRC-5 cells increased after 24 h and was followed by the increase in caspase activity later on (48 h).

**Conclusion:** Cytotoxic action of MTX was stronger on human leukemic cells compared to healthy human fibroblasts after 72 h treatment. MTX led to earlier and more intensive activation of caspases in leukemic cells than in healthy fibroblasts, while not increasing the amount of acidic vesicles in MOLT-4 cells. But, MTX led to intracellular acidification of healthy fibroblasts’ cytoplasm which suggests the activation of autophagy.

**PS170 The impact of cholesterol supplementation in experimental pulmonary arterial hypertension**

Liliana Costa^1^, Lucas Lopes^2^, Sara Leite^2^, Rita Ferreira^1^, M. Rosário Domingues^1^, Adelino Leite-Moreira^2^, Rita Nogueira-Ferreira^2^, André P. Lourenço^2^

^1^*QOPNA, Department of Chemistry, University of Aveiro, Aveiro, Portugal,*^2^*Department of Surgery and Physiology, Faculty of Medicine, University of Porto, Porto, Portugal E-mail address: liliana.isabel.costa@ua.pt (Liliana Isabel Moreira Costa)*

**Introduction:** Cardiac cachexia (CC) is a severe complication of chronic heart failure (HF), characterized by poor prognosis [1]. The cholesterol paradox has emerged from reports of low serum levels of total cholesterol as an adverse prognostic marker in HF [2,3]. The endotoxin-lipoprotein hypothesis states that higher circulating levels of cholesterol and lipoproteins can attenuate the CC-related immune activation [4–6].

**Aim:** Our aim was to test the endotoxin-lipoprotein hypothesis in vivo, by the analysis of the effects of dietary supplementation with cholesterol in monocrotaline (MCT)-induced pulmonary arterial hypertension (PAH), HF and CC animal model.

**Methods:** Adult male Wistar Han rats were injected with MCT (60 mg/kg) or an equal volume of vehicle. Five days after, MCT-injected rats were randomly allocated to consume a normal diet or a cholesterol supplemented diet (cholesterol 2% and cholic acid 0.25%). Between the 25th and 30th day, animals underwent echocardiographic and hemodynamic evaluation. We assessed body weight (BW) evolution, cardiomyocyte cross-sectional area and pulmonary arterioles wall-thickness. Plasma concentration of total cholesterol, high density lipoprotein-cholesterol (HDL-C), triglycerides, tumour necrosis factor-alpha (TNF-a) and endotoxin LPS was also determined.

**Results:** PAH was successfully established by the increase of mean pulmonary arterial pressure, accordingly with a higher pulmonary arterioles wall-thickness. MCT administration induced right ventricle hypertrophy and dysfunction, accompanied with significant BW loss, related with CC. Cholesterol supplemented diet induced an increase of plasma total cholesterol, HDL-C and non-HDL cholesterol concentration, liver weight and left ventricle cardiomyocyte cross-sectional area. It also showed a trend towards decreased TNF-a and endotoxin LPS plasma levels.

**Conclusion:** Our results showed that MCT administration effectively resulted in PAH, RV dysfunction and CC. Results suggest that higher lipoprotein content may have an anti-inflammatory role in CC, although further investigation is needed in order to clarify these effects.

**References**

1. T. Martins, R. Vitorino, D. Moreira-Gonçalves, F. Amado, J. Alberto and R. Ferreira, Recent insights on the molecular mechanisms and therapeutic approaches for cardiac cachexia, Clin Biochem, 2014, 47(1–2), 8–15.

2. P. Velavan, P.H. Loh, A. Clark and J.G.F. Cleland, The cholesterol paradox in heart failure, Congest Hear Fail, 2007, 13(6), 336–341.

3. H. Fröhlich, N. Raman, T. Täger, D. Schellberg, K.M. Goode, S. Kazmi, M. Grundtvig, T. Hole, J.G.F. Cleland, H.A. Katus, S. Agewall, A.L. Clark, D. Atar and L. Frankenstein, Statins attenuate but do not eliminate the reverse epidemiology of total serum cholesterol in patients with non-ischemic chronic heart failure, Int J Cardiol, 2017, 238, 97–104.

4. M. Rauchhaus, A.J.S. Coats and S.D. Anker, The endotoxin-lipoprotein hypothesis, Lancet, 2000, 356(9233), 930–933.

5. S. von Haehling, J.C. Schefold, J. Springer and S.D. Anker, The cholesterol paradox revisited: heart failure, systemic inflammation, and beyond, Hear Fail Clin, 2008, 4(2), 141–151.

6. A.P. Lourenço, F. Vasques-Nóvoa, D. Fontoura, C. Brás-Silva, R. Roncon-Alburquerque and A.F. Leite-Moreira, A Western-type diet attenuates pulmonary hypertension with heart failure and cardiac cachexia in rats, J Nutr, 2011, 141(11), 1954–1960.

**Acknowledgements**

This work was supported by Portuguese Foundation for Science and Technology (FCT), European Union, QREN, FEDER and COMPETE for funding the QOPNA (UID/QUI/00062/2013) and Unidade de Investigação Cardiovascular (UID/IC/00051/2013) research units and the research projects PTDC/DTP-PIC/4104/2014, NETDIAMOND (POCI-01-0145-FEDER-016385) and DOCnet (NORTE-01-0145-FEDER-000003). S.L. is supported by individual fellowship grant (SFRH/BD/110404/2015).

**PS178 Phenotypic and functional characterization of decidual natural killer cells at term pregnancy**

Ricardo de M. Vieira^1,2^, Ângela C. Crespo^2,3^, Jack L. Strominger^2^, Tamara Tilburgs^2^

^1^*Faculty of Medicine, University of Coimbra, 3004-504 Coimbra, Portugal,*^2^*Department of Stem Cell and Regenerative Biology, Harvard University, Cambridge, MA 02138,*^3^*Program in Cellular and Molecular Medicine, Boston Children's Hospital, Boston, MA 02115 E-mail address: mvieira.ricardo@gmail.com (Ricardo de Mendonça Vieira)*

**Introduction:** The mechanisms underlying the immune tolerance of the pregnant woman towards the foreign fetus during pregnancy are still an upfront challenging topic for the biomedical community. Natural Killer cells from decidua (dNK), as the main cell population in the uterine microenvironment, are known to be crucial for trophoblast implantation and protection against infections that may impact pregnancy success, but their function during the third trimester of pregnancy is largely unknown.

**Aim:** For a better understanding of the immune balance throughout the course of a pregnancy, a phenotypic and functional characterization of dNK cells at term pregnancy decidua basalis and decidua parietalis tissues is in this work assessed.

**Methods:** Discarded human placenta and maternal decidua tissue samples were obtained from women after a healthy term pregnancy delivery (gestational age > 37 weeks) or undergoing voluntary medical pregnancy termination (gestational age 6–12 weeks) at a local reproductive health clinic.

**Results:** The results obtained are innovative: dNK from decidua basalis and decidua parietalis show differences in their cell surface NK receptor profile, granules content and ability to degranulate in response to PMA/Ionomycin. Exploratory studies to determine the impact of KIR expression in relation to degranulation capacity of dNK cells was also studied and it is demonstrated that KIR2DL1 + /S1 + dNK cells have an increased levels of cytolytic granules.

**Conclusion:** Future studies should expand functional analysis on term placenta dNK to increase scientific knowledge relevant to clinical practice, namely the mechanism that induce labour. This will, not only contribute for an overall picture of the nine months pregnancy but also help in predicting and preventing complications, such as viral and bacterial infections, during pregnancy.

**Acknowledgements**

I express my gratitude to Dr. Tamara Tilburgs, for guiding me through experimental protocols, to contribute to my critical thinking and for encouraging me to be more independent and pragmatic researcher. I truly appreciated the inputs of Professor Manuel Santos Rosa together with Dr. Tamara on the results discussions throughout the entire process. Dr. Ângela Crespo couldn’t have been more welcoming and she is, to me, a true reference as a scientist. Thank you all for leading me to become a better scientist and to learn to conduct rigorous experiments.

**PS192 Effects of polyoxotungstates on liver and kidney function**

Aleksa Jovanovic’^1^, Valentina Balint^1^, Marko Dinčic’^2^, Mirjana Čolovic’^3^, Ali S. Mougharbel^4^, Ulrich Kortz^4^, Danijela Krstic’^5^

^1^*Faculty of Medicine, University of Belgrade, Belgrade, Serbia,*^2^*Institute of Pathological Physiology, Faculty of Medicine, University of Belgrade, Belgrade, Serbia,*^3^*Department of Physical Chemistry, “Vinc*’*a” Institute of Nuclear Sciences, University of Belgrade, Belgrade, Serbia,*^4^*Department of Life Sciences and Chemistry, Jacobs University, Bremen, Germany,*^5^*Institute of Medical Chemistry, Faculty of Medicine, University of Belgrade, Belgrade, Serbia E-mail address: jaleksa159@gmail.com (Aleksa Jovanovic*’*)*

**Introduction:** Polyoxometalates are clusters of oxygen and transitional metals, exhibiting antimicrobial, antitumor, antiacetylcholinesterase and antihyperglycaemic effects. Despite their promising medical application, the proved toxicity is a major limit for their broader usage.

**Aim:** The aim of this paper was to examine effects of two polyoxotungstates: (NH4)14[NaP5W30O110] 31H2O (NaP5W30) and (NH4)14[AgP5W30O110] 31H2O (AgP5W30) on liver and kidney function in exposed Wistar rats by monitoring relevant biochemical parameters.

**Methods:** Experiments were performed on adult male Wistar rats. The animals in the first (control) group (n = 7) were receiving saline (0.9% NaCl) per os. The second (n = 7) and third group (n = 7) animals were receiving orally 20 mg/kg/day of NaP5W30 and AgP5W30, respectively, for 14 days. Blood samples were taken by cardiac puncture. The activities of alanine aminotransferase (ALT), aspartate aminotransferase (AST) and the concentrations of total proteins, albumins, urea and creatinine were then determined in isolated serum.

**Results:** Statistically significant decrease of serum total proteins and albumins concentrations in the animals treated with both polyoxotungstates compared to the control group was obtained, while serum ALT and AST activities were not significantly different among the groups. Statistically significant increase of the renal toxicity biomarkers, serum urea and creatinine concentrations, in the both treated groups related to the control group was observed.

**Conclusion:** The absence of a significant difference among groups, considering ALT and AST activities, suggests that the investigated polyoxotungstates in the applied dose do not induce acute hepatotoxicity. However, the found significant decrease of total proteins and albumins concentrations in the exposed animals implies that NaP5W30 and AgP5W30 alter synthetic liver function. Additionally, the statistically significant increase of serum urea and creatinine concentrations in the treated animals suggests impaired kidney function.

**PS195 Changes in gene expression and adenosine A2B receptor activity in the failing right ventricle secondary to pulmonary arterial hypertension**

Eduardo Martins-Dias^1^, Mafalda Bessa-Gonçalves^1^, Bruno Bragança^1^, Anna Esteve-Codina^2^, Adriana Vinhas^1^, Mariana Certal^1^, Fátima Ferreirinha^1^, Maria Adelina Costa^1^, Estela Bastos^3^, Ivo Glynne Gut^2^, Paulo Correia-de-Sá^1^, Ana Patrícia Fontes-Sousa^1^

^1^*Laboratório de Farmacologia e Neurobiologia, Center for Drug Discovery and Innovative Medicines (MedInUP), Instituto de Ciências Biomédicas Abel Salazar (ICBAS) – Universidade do Porto (UP), Porto, Portugal,*^2^*Centro Nacional de Análisis Genómico (CNAG) – Centre for Genomic Regulation (CRG), Barcelona Institute of Science and Technology (BIST), Barcelona, Spain,*^3^*Department of Genetics and Biotechnology, School of Life and Environmental Sciences (ECVA), Center of the Research and Technology of Agro-Environmental and Biological Sciences (CITAB), University of Trás-os-Montes and Alto Douro (UTAD), Vila Real, Portugal E-mail address: eduardo.dias.1994@gmail.com (Eduardo Ferreira Martins Dias)*

**Introduction:** Pulmonary arterial hypertension (PAH) is a progressive, potentially fatal disease characterized by an adverse pulmonary vascular remodeling that consequently overloads the heart. Adenosine is an important regulatory molecule that has been implicated in right ventricle (RV) remodeling and fibrosis during PAH development, though its role remains controversial and poorly understood.

**Aim:** This study aimed at evaluating the in vitro activity of adenosine A2A and A2B receptors in cultured cardiac fibroblasts (CFs), as well as the right ventricular gene expression pattern of PAH rats.

**Methods:** Cell proliferation and type I collagen production were measured by MTT and Sirius Red assays, respectively, in cultured CFs isolated from the RV of male Wistar rats with PAH induced by monocrotaline (60 mg/kg, SC; MCT group). RV samples were collected for RNA sequencing in order to perform a differential gene expression analysis in PAH and healthy rats.

**Results:** Pretreatment with NECA (10 mM), a non-hydrolysable adenosine analogue, significantly augmented proliferation of cultured CFs from PAH rats compared to control littermates. The adenosine A2A receptor antagonist, SCH442416 (100 nM), failed to modify NECA-induced CFs growth, while the A2B antagonist, PSB603 (100 nM), attenuated (P < 0.05) the proliferative effect of NECA (10 mM). Regarding the differential gene expression analysis, data show several genes (FDR < 0.05) are upregulated in RV samples of PAH rats, in particular those encoding proteins involved in cell cycle progression, DNA replication, extracellular matrix-receptor interactions, but also in the TGF-b and PI3K-AKT signaling pathways.

**Conclusion:** Results point towards adenosine A2B receptor as a putative therapeutic target to attenuate RV remodeling and fibrosis in PAH, but also suggests the involvement of other P1 receptors (e.g. A1 and A3) in excessive CFs proliferation and extracellular matrix deposition. In addition, the identification of gene expression changes underlying RV remodeling during PAH pathogenesis may be extremely useful to future research based on gene-targeted therapies.

**Acknowledgements**

Work supported by FCT (UID/BIM/4308/2016)

**PS199 Impact of CTLA-4 inhibition on anti-cancer activity of lymphocytes against MDA-MB-231 breast cancer cell line model**

Anna Kretowska^1^, Aleksandra Basaj^1^, Malgorzata Kowalska^1^

^1^*Department of Regenerative Medicine and Immune Regulation, Medical University of Bialystok, Poland E-mail address: annammkretowska@gmail.com (Anna Kretowska)*

**Introduction:** CTLA-4 is an immune checkpoint molecule pivotal in regulating the intensity of immune response. Decreased immune system activation through CTLA-4 was shown when interacting with adequate ligands highly expressed in cancers. Breast cancer is the most commonly diagnosed cancer among women. Therefore efforts aimed at increasing anti-cancer activity through immune checkpoint molecule blockage in breast cancer patients are of great importance in novel therapeutics development.

**Aim:** In this study, we aimed to determine whether in vitro inhibition of CTLA-4 could improve anti-cancer activity of lymphocytes in reference to MDA-MB-231 breast cancer cell line model.

**Methods:** The study was performed on MDA-MB-231 breast cancer cell line. Cancer cells were subjected to 24-hour incubation with activated PBMC from healthy blood donors, in presence or absence of CTLA-4 blocking antibodies. Staining with CFSE was used to establish cell proliferation, propidium iodide (PI) to determine cell cycle status. LDH-based assay was conducted to evaluate anti-cancer cytotoxic effect. FACSCalibur flow cytometer was used for acquisition of proliferation and cell cycle data, and LEDETECT96 microplate reader for colorimetric measurement of LDH level in supernatants.

**Results:** The acquired data analysis revealed that inhibition of CTLA-4 on PBMC resulted in significant decrease (p = 0.0391) of MDA-MB-231 breast cancer cells proliferation. Additionally, cell cycle arrest in G1/S-phase transition was observed. Assessment of LDH activity showed cytotoxic activity of PBMC alone on cancer cells (p = 0.0273) with significantly enhanced effects found in additional presence of CTLA-4 inhibition (p = 0.0039).

**Conclusion:** In conclusion, inhibition of CTLA-4 immune checkpoint molecule might have a beneficial impact on lymphocytes’ anti-cancer activity, and thus, reducing breast cancer cells progression and viability. Further experiments are required to comprehensively evaluate the effects of CTLA-4 blockage on breast cancer cells and to proceed toward development of novel therapeutic approaches.

**PS201 Sensor B-cell platform based on the HIV-specific broadly neutralizing antibody 10E8**

Darya Chernikova^1^, Andrey Gorchakov^1^, Konstantin Baranov^2^, Alexander Taranin^1^

^1^*Novosibirsk State University; Institute of Molecular and Cellular Biology SB RAS,*^2^*Institute of Molecular and Cellular Biology SB RAS E-mail address: d.chernikova@g.nsu.ru (Chernikova Darya)*

**Introduction:** Identification of antigens driving affinity maturation of HIV-specific antibodies towards broad neutralization has remained one of the most challenging endeavors in the HIV vaccine development field. In order to isolate such candidate immunogens and to analyze whether they may activate B-cells expressing BCRs based on immature/germline forms of bnAbs, a robust test system that faithfully recapitulates human B-cell biology is needed. Thus, developing human B-cell sensor lines with surface expression of immature/germline variants of bnAbs may represent one of the possible solutions.

**Aim:** The aim of our study is to create a cellular platform composed of B-cell sensor lines expressing antibodies of the 10E8 lineage at different maturation steps.

**Methods:** Coding sequences for 10E8 antibodies corresponding to germline, intermediate, and mature variants were extracted from the published datasets. Variable sequence of the 10E8 heavy chain was cloned in-frame with the constant region of human IgG1, self-cleaving P2A sequence and the light chain-encoding sequence. This cassette was placed into the pCDH vector (zeo resistant), therefore, lentiviral constructs encoding membrane-anchored forms of different variants of 10E8 were generated: germline, two early intermediate and mature. The constructs were co-transfected into HEK293T cell line with the packaging plasmids, the lentiviral particles obtained were used for transducing human lymphoma cell line BJAB.

**Results:** Four human B-cell lines expressing membrane-anchored forms of bnAb 10E8 at various maturation stages were established. Upon stimulation, the expected increase in intracellular Ca2 + levels was observed using Ca-flux assay. Thus, these cell lines became specifically activated upon addition of anti-human IgGs and several candidate immunogens, i.e. 10E8-based BCRs assembled on their surface were fully functional.

**Conclusion:** The cellular sensor platform developed in our study is instrumental to the identification and comparison of immunogens that drive maturation of the 10E8 antibody lineage and which may serve as the components of an effective vaccine against HIV-1.

**PS212 Moderate RA activity is associated with increased IL6, IL8, IL10 and IFNg serum concentration comparing to remission patients**

Ksenija Antic^1^

^1^*School of Medicine, Military Medical Academy E-mail address: ka.ksenija.antic@gmail.com (Ksenija Antic*’*)*

**Introduction:** Rheumatoid arthritis is chronic autoimmune disease of unknown cause. All processes in RA pathogenesis are directed by a network of cytokines produced by lymphocytes, macrophages, resident cells such as osteoclasts, chondrocytes and fibroblasts and other cells present in inflamed synovium.

**Aim:** Evaluation of the association between RA disease activity based on DAS-CRP score and cytokines levels measured in peripheral blood of RA patients.

**Methods:** Serum concentrations of cytokines (IL1b, IL2, IL4, IL5, IL6, IL8, IL10, IL12, TNFa, IFNg) were measured in samples of RA patients and control group by using commercial ELISA kits. Patients were divided in four groups according to disease activity determined by DAS-CRP (remission, low, moderate and high active disease). This study was approved by Ethics Committee of Military Medical Academy of Serbia.

**Results:** Concentrations of IL12 were higher in all patients groups than in the control group, but without significant difference. Concentrations of IL1b and IL2 were significantly elevated in all groups of patients compared to control group. The highest concentration of IL6, IL8, IL10 and IFNg was detected in a group of patients with moderate disease activity and those values were significantly higher compared to patients in remission and with the control group. Concentrations of IL4 and IL5 were almost equal in patents in remission and in the control group. Patients with low, moderate and high active disease had significantly elevated IL4 and IL5 levels compared to controls and patients in remission. Average TNFa concentration was the highest in patients with low active RA and that was significantly higher than in patients in remission and controls.

**Conclusion:** All analyzed cytokines, except IL12, could be new diagnostic markers of RA, indicators of disease activity or new therapeutic targets.

**PS219 Microenvironment influence on stem-like cells differentiation**

Filipa Teles^1,2^, Carlos Miguel Marto^1,2,3,4,5^, Mafalda Laranjo^2,4,5^, Anabela Paula^1,2,4,5^, Ana Cristina Gonçalves^4,5,6^, Maria Filomena Botelho^2,4,5^, Eunice Carrilho^1,2,4,5^

^1^*Dentistry Area, Faculty of Medicine, University of Coimbra, Portugal,*^2^*Biophysics Institute, Faculty of Medicine, University of Coimbra, Portugal,*^3^*Experimental Pathology Institute, Faculty of Medicine, University of Coimbra, Portugal,*^4^*Institute for Clinical and Biomedical Research (iCBR), area of Environment Genetics and Oncobiology (CIMAGO), Faculty of Medicine, University of Coimbra, Coimbra, Portugal,*^5^*CNC.IBILI, University of Coimbra, Portugal,*^6^*Laboratory of Oncobiology and Hematology (LOH), Faculty of Medicine, University of Coimbra, Portugal E-mail address: filipateles00@gmail.com (Ana Filipa de Sousa Teles)*

**Introduction:** Regenerative dentistry research has advanced significantly, much because of the identification of dental stem cells. Various sources of stem cells are known, both of oral and systemic origin. However, harvesting these cells poses some limitations, therefore, new sources of stem cells are needed. Dedifferentiation of adult oral cells is a methodology our group has been studying. Influence of microenvironment in cell differentiation is known, thus the interaction between stem-cells and gingival fibroblasts is of great interest regarding future regenerative procedures.

**Aim:** This work aims to evaluate the interference of gingival fibroblast conditioned media in stem-like cells viability and differentiation.

**Methods:** A primary culture of mouse gingival fibroblasts was obtained by explant methodology and cultured under adherent conditions. After plating, a 4-day dedifferentiation protocol was performed to obtain stem-like cells. These cells were incubated for 48 hours with media conditioned by several numbers of gingival fibroblasts. Protein content and DNA amount were evaluated by SRB and crystal violet assays, respectively. Differentiated cells morphology was evaluated by crystal violet and May-Grunwald Giemsa staining.

**Results:** Gingival conditioned media did not alter significantly the protein and DNA contents of stem-like cells. All groups show morphological changes compatible with stem-like phenotype and phenotypic differences were observed between groups.

**Conclusion:** Treatment with gingival fibroblasts conditioned media ensured cell viability. Morphological alterations encourage evaluation of lineage differentiation, which will be performed using Alizarin red s, Oil Red and Alcian Blue colorations.

**PS224 Stimulation of MAC-inhibitory protein (CD59) but no complement decay-accelerating factor (CD55) induces release of neutrophil extracellular trap (NETs)**

Justyna Holka^1^, Paulina Tarnowska^1^, Weronika Bystrzycka^2,3^, Aneta Manda-Handzlik^2,3^, Małgorzata Wachowska^3^, Olga Ciepiela^3^

^1^*Student's Scientific Group at Department of Laboratory Diagnostics and Clinical Immunology of Developmental Age, Medical University of Warsaw, Poland,*^2^*Postgraduate School of Molecular Medicine, Medical University of Warsaw, Poland,*^3^*Department of Laboratory Diagnostics and Clinical Immunology of Developmental Age, Medical University of Warsaw, Poland E-mail address: justyna.holka@gmail.com (Justyna Anna Holka)*

**Introduction:** MAC-inhibitory protein, also known as CD59 is a membrane inhibitor of reactive lysis found on cell surface of leukocytes and erythrocytes. When complement activation leads to deposition of C5b678 on host cells, CD59 can prevent C9 from polymerizing and forming the complement membrane attack complex. CD55 recognizes C4b and C3b fragments that are created during activation of C4 (classical or lectin pathway) or C3 (alternative pathway) thus indirectly blocks the formation of the membrane attack complex. CD59 and CD55 attach to host cells via a glycophosphatidylinositol (GPI) anchor. A mutation of PIG-A gene, which leads to deficiency of GPI, is found in patient with paroxysmal nocturnal hemoglobinuria.

**Aim:** The aim of the study was to establish, if release of neutrophil extracellular traps might be dependent on CD59 or CD55 activation and possible mechanisms of their action.

**Methods:** Neutrophils were isolated from the blood of healthy donors. Cells were obtained by density gradient centrifugation and subsequent polyvinyl alcohol sedimentation of erythrocytes. Subsequently, neutrophils were incubated with human antiCD59 or antiCD55 monoclonal antibody and 100 nM PMA was added to stimulate NETs release. In the second part of the experiment neutrophils were incubated with human antiCD59 and also with NETs inhibitors: DPI, ABAH and NAC. The process of NETs release was assessed 3 h post stimulation by fluorescent microscopy and fluorometry.

**Results:** Anti-CD59 antibody induced NETs release in a concentration-dependent manner, with the highest release at 0.25 – 1.25 μg/ml (unstimulated neutrophils vs. PMA∗∗∗ p≤0.001 and unstimulated neutrophils vs. CD59∗ p≤0.05). DPI, ABAH and NAC inhibited NETs release under influence of anti-CD59 (CD59 vs. CD59 + DPI p∗∗∗∗∗0.05, CD59 vs. CD59 + ABAH∗∗ p≤0.01, CD59 vs. CD59 + NAC∗ p≤0.05). Fluorescent microscopy confirmed results of quantification method. None of studied anti-CD55 monoclonal antibody concentration lead to release of NETs.

**Conclusion:** Neutrophil extracellular traps release is dependent on activity of GPI-anchored proteins which regulates complement activation pathway. Stimulation of CD59 antigen leads to NETs release on reactive oxygen species/myeloperoxidase activity dependent pathway.

**PS251 Expression of GLUT1 and GLUT4 in right ventricle in pulmonary arterial hypertension – effects of chronic treatment with NRG-1**

Ana Rita Pinto^1^, Carolina Maia-Rocha^1^, Rui Adão^1^, Pedro Mendes-Ferreira^1^, Adelino Leite-Moreira^1^, Carmen Brás-Silva^1,2^

^1^*Department of Surgery and Physiology, Faculty of Medicine, University of Porto, Portugal,*^2^*Faculty of Nutrition and Food Sciences, University of Porto, Porto, Portugal E-mail address: anaritapinto4@gmail.com (Ana Rita Barbosa Pinto)*

**Introduction:** Glucose is transported into the heart by facilitative glucose transporters (GLUTs). The two major isoforms of GLUTS in the myocardium are GLUT1 and 4. Neuregulin-1 (NRG-1), a protein that has been shown to play beneficial effects on Pulmonary Arterial Hypertension (PAH) and right ventricle (RV) hypertrophy1, has also been associated with the regulation of glucose metabolism.2

**Aim:** In this study we aimed to investigate the GLUT1 and 4 expressions in RV and the effect of chronic treatment with NRG1 on these transporters, in an animal model of PAH.

**Methods:** Wistar rats randomly received 60 mg/kg of monocrotaline (MCT) or vehicle. After 14 days, they were randomly treated with rhNRG-1 (40 mg/kg/day) or vehicle. The study resulted in 4 groups: control (CTRL); CTRL + rhNRG-1; MCT and MCT + rhNRG-1. Between the 21st and 24th hemodynamic studies and sample collection were performed.

**Results:** The decrease in ejection fraction (EF) correlates with increased GLUT1 expression (p = 0.0005) and decrease in GLUT4 expression (p = 0.0167). We observed that increased GLUT1 expression correlates with increased hypoxia-inducible factor 1-alpha (HIF1a) expression (p < 0.0001). The decrease in GLUT4 expression was shown to correlate with increased brain natriuretic peptide (BNP) (p = 0.0032) and endothelin 1 (ET1) (p = 0.0006) expression. We observed an increase of GLUT1 in the MCT group (4.13 ± 0.49AU) compared to the CTRL group (1.00 ± 0.19AU), in the MCT + rhNRG-1 group these values were completely reverted (1.66 ± 0.31AU). GLUT4 increased in all groups of animals treated with rhNRG1 (CTRL + rhNRG-1 = 1.41 ± 0.09AU and MCT + NRG1 = 1.48 ± 0.18AU vs. CTRL = 1.00 ± 0.16AU and MCT = 0.75 ± 0.02AU).

**Conclusion:** We observed that the expression of GLUT1 is associated with the development of the disease whereas GLUT4 is affected by chronic treatment with rhNRG1. The expression of GLUTs correlate with parameters of cardiac function and disease and chronic treatment with rhNRG-1 attenuates the changes. We can conclude that therapeutic effects of rhNRG-1 in PAH might be due, in part, to the regulation of GLUTs.

**References**

1. Mendes-Ferreira P (2016) Neuregulin-1 improves right ventricular function and attenuates experimental pulmonary arterial hypertension. Cardiovasc Res.1; 109(1):44-54.

2. Cantó C, et al. (2004). Neuregulin signaling on glucose transport in muscle cells. Journal of Biological Chemistry. 279: 260-268.

**PS269 Chronic spontaneous urticaria - potencial etiological factors**

Zorana Kovacevic^1^

^1^*Faculty of Medicine,*^1^*University of Belgrade E-mail address: zoca005@gmail.com (Zorana Kovacevic)*

**Introduction:** Chronic spontaneous urticaria (CSU) is characterized by spontaneus appearance of wheals, angioedema or both, lasting more than 6 weeks, with no eliciting factor involved. From the patients with CSU, it is considered that 45% have “autoimmune urticaria” (AU) which occurs in patients with autoimmune diseases. Other causes can be infections - especially Helicobacter pylori, but also infections of respiratory and urinary tract. Cause may also be parasite infestation –Toxoplasma gondii, Toxocara canis, Stronyiloides stercoralis.

**Aim:** To determine association of CSU with autoimmune diseases, infections, or other potential provoking and contributing factors to this disease, and to compare differences between patients with and without angioedema.

**Methods:** This retrospective study included 42 patients hospitalized at Clinic for Allergology and Immunology of the Clinical Center of Serbia, with a confirmed diagnosis of CU, in period from July 2017 to June 2018.

**Results:** Patients were 69.05% women and 30.95% men, average age 53 ± 15.9 years. Wheals and angioedema were present in 29 (69.04%) patients while others (13/42) had no angioedema. Sensibilisation to at least one allergen was confirmed in 47.6% of patients. Regarding infections, 18 (42.85%) patients had at least one at the same time with CSU, 9 of them had urinary and 7 had respiratory infection. Autoimmune diseases were present in 28.6% of patients, most common was autoimmune thyroiditis. Autoimmune antibodies were present in 54.8% of patients (the most common were antinuclear antibodies – ANA). There was no statistically significant difference between patients with and without angioedema regarding presence of autoimmune diseases (p = 0.08) nore autoimune antibodies (p = 0.321).

**Conclusion:** This study has showen a high percentage association of CSU with potential provoking and contributing factors in its onset.

Public Health & Medical Informatics

**PS019 Prevalence of antibiotic self-medication among Port Said University students**

Khaled Mohamed Abdelwahab^1^, Ahmed Gamal Khoder^1^, Ahmed Mostafa^1^, Mohamed Elshaer^1^

^1^*Faculty of Medicine, Port Said University E-mail address: ahmedkhoder2017@yahoo.com (Ahmed gamal faried mohamed khoder)*

**Introduction:** An antibiotic is defined as a chemical substance that selectively inhibits growth of microorganisms and causes minimum damage to the host cells. They are normally only prescribed for more serious bacterial infections, for example, pneumonia. When prescribed, it is important to take the entire course of antibiotics which helps to prevent resistance developing to that antibiotic.

Antibiotics, which were hailed as the miracle drugs that cured most infected people before, do not work in many cases today. This is because bacteria are increasingly becoming resistant to antibiotics at an alarming rate and the resistance is spreading throughout the world among all species of bacteria. The main reason for this resistance development is the excessive use of the antibiotics.

The only way that is left is just to encourage individuals to follow rational use of drugs and to raise awareness among patients and society by conducting different seminars emphasizing on how to avoid misuse of antibiotics.

**Aim:** This study is aiming to Increase awareness towards antibiotic self-medication among Port Said university students.

**Methods:** A cross-sectional, descriptive study design was selected to measure the prevalence of self-medication antibiotics and know reasons leading to self-medication antibiotics among Port Said university students. (n = 414)

**Results:** Of all 414 participants we found that (80.2%) had taken antibiotics within 2017 and (61.8%) of them had taken antibiotics without consultation(75.1%)of students take antibiotics due to presence at home,(10.9%) of them to save cost. According to our question about time of stoppage antibiotics we found that (59.2%) stopped after symptoms disappeared.

**Conclusion:** Based on the results of our study, we concluded that we need to educate patients regarding antibiotic use and the consequences of misuse: what diseases actually require antibiotics, why full daily doses must be respected, and danger of keeping part of a course for future uncontrolled use.

**Acknowledgements**

- First and foremost, all praises and thanks to Allah, the Almighty, for his assistance and favor to accomplish this work.

- We would like to express our deepest appreciation to our supervisors,Dr. Mohamed Elshaer lecturer of pharmacology at Faculty of Medicine- Ain shams University and Dr. Ahmed Mustafa,Assistant Lecturer of pharmacology Faculty of Medicine – Port Said University

- Who guided us throughout our project and offered with constructive criticism that made our project what it is.

- We would like to express our sincere thanks towards Port-Said university students who devoted their time to help us in the implementation of this project.

- Nevertheless, we express our gratitude toward our colleagues for their kind co-operation and encouragement, which help us in completion of this study.

**PS029 Lifestyle and health status of future Portuguese physicians: a cross-sectional study**

Sara Leite^1,2^, Maria Mafalda Osório^1^, Joana Miguel^1^, Miguel Monteiro^1^, Marisa Lage^1^, Carlos Carvalho^1,3^

^1^*Institute of Biomedical Sciences Abel Salazar, University of Porto (ICBAS-UP), Porto, Portugal,*^2^*Department of Surgery and Physiology, Faculty of Medicine, University of Porto (FMUP), Porto, Portugal,*^3^*Northern Regional Health Administration - Public Health Department, Porto, Portugal E-mail address: leite.sara1@gmail.com (Sara Vanessa de Amorim Leite)*

**Introduction:** Non-communicable diseases, which have been strongly associated with unhealthy habits, are the main cause of death in developed world [1]. Studies have shown that a deterioration of physicians’ lifestyles and health during medical training can lead to delivering a deficient health promotion message for their patients [2,3].

**Aim:** The aim of this study was to assess lifestyle and health status of Portuguese medical students.

**Methods:** A cross-sectional survey was conducted in March 2018 via social networks in five Portuguese medical schools located in Porto, Lisbon, Coimbra and Braga, using a questionnaire self-administered to undergraduate students. Results were described in terms of proportions and differences according to year of training were analysed.

**Results:** The questionnaire was completed by 399 medical students. The majority (84%) had normal BMI, while 9% were overweight and 8% underweight. 78% believed they had a healthy diet. Throughout the medical school years, an increasing proportion of students choose taking homecooked meals instead of eating out (66% in the 6th year vs 41% in the 1st year).

Seventy one percent said they practiced some exercise. The highest proportion of sedentary students (52%) was observed in the first year of medical school when compared with the rest of the years (27%).

Among medical students who reported an unhealthy diet (22%) and students who did not exercise (29%), lack of time to cook or to exercise were identified as the main reasons (53% and 62%, respectively).

**Conclusion:** Contrary to other countries [2,4], this study revealed that more than half of Portuguese medical students choose a healthy diet and practice exercise. Nevertheless, there is still room for integration of health-promotion counselling that can be carried out during medical training in order to improve health outcomes for future doctors and their patients [5,6].

**References**

1. Global, regional, and national age-sex specific all-cause and cause-specific mortality for 240 causes of death, 1990–2013: a systematic analysis for the Global Burden of Disease Study 2013. Lancet (London, England). 2015;385(9963):117-71.

2. Majra J. Do our medical colleges inculcate health-promoting lifestyle among medical students: a pilot study from two medical colleges from southern India. International journal of preventive medicine. 2013;4(4):425–9.

3. Sajwani RA, Shoukat S, Raza R, Shiekh MM, Rashid Q, Siddique MS, et al. Knowledge and practice of healthy lifestyle and dietary habits in medical and non-medical students of Karachi, Pakistan. JPMA The Journal of the Pakistan Medical Association. 2009;59(9):650–5.

4. Carter AO, Elzubeir M, Abdulrazzaq YM, Revel AD, Townsend A. Health and lifestyle needs assessment of medical students in the United Arab Emirates. Medical teacher. 2003;25(5):492–6.

5. Henry RC, Ogle KS, Snellman LA. Preventive medicine: physician practices, beliefs, and perceived barriers for implementation. Family medicine. 1987;19(2):110–3.

6. Frank E, Rothenberg R, Lewis C, Belodoff BF. Correlates of physicians’ prevention-related practices. Findings from the Women Physicians’ Health Study. Archives of family medicine. 2000;9(4):359-67.

**PS034 Quality of postoperative analgesia: patients’ attitudes**

Maja Vlade Vilotijevic^1^, Milica Dejan Durkovic^1^

^1^*Institute of Pharmacology, Clinical Pharmacology and Toxicology, Faculty of Medicine University of Belgrade E-mail address: maja.vilotijevic95@gmail.com (Maja Vilotijevic)*

**Introduction:** The experience of childbirth is very important in womens life. Unfortunately only few studies have examined obstetric patients’ anesthesia preferences and satisfaction. One of the obligations of obstetricians and anesthesiogists is eliminating postoperative pain. Appropriate pain management allows better physical and psychological recovery.

**Aim:** Assessment of the patients’ preferences and satisfaction with the anesthesia and postcesarean analgesia.

**Methods:** This is a cross-sectional study for which specially created questionnaire was distributed to 62 consecutive women in order to determine patients preferences, anticipatory fears and postoperative anesthesia outcomes. Questionnaire comprises questions about demographic characteristics, previous experience with general and regional anesthesia, questions about anticipatory fears and outcomes after the caesarean section, whose intensity is graphically evaluated on a visual analog scale of 0 to 100 mm (VAS 0–100).

**Results:** The results of the study show that the most patients were satisfied (68.9%), with no serious side effects. The greatest fear was throat pain (marked on VAS 0–100, mean 20 mm), followed by fear of side effects of anesthesia on baby, vomiting and shivering. The most common clinical side effect was throat pain (35 mmVAS 0–100), followed by shivering and vomiting. There were no significant statistical correlations between demographic characteristics, the previous birth experience with the frequency of anticipatory attitudes or post-operative experience. However, there was significant difference between the level of anticipated and experienced intensity of anesthesia adverse effects.

**Conclusion:** The choice of the anesthesia technique in which cesarean section should be performed should be made by the doctors and patients together, taking into account the preferences of the mother. Raising the awareness of doctors about anticipatory attitudes, as well as the most commonly experienced unwanted effects of anesthesia, would significantly contribute to finding the most optimal solution in treatment - effective pain relieving, without harmful consequences.

**PS041 Options to reduce avoidable hospitalizations and rehospitalizations for persons with schizophrenia spectrum disorders - Latvian health billing system's data analysis**

Alise Gorčakova^1^, Juris Ba-rzdin·š^1^, Artjoms Baharevs^2^

^1^*University of Latvia,*^2^*Riga Stradins University E-mail address: alise.gorcakova@gmail.com (Alise Gorčakova)*

**Introduction:** Schizophrenia spectrum disorders (SSD) are severe, chronic, debilitating and they affect 7/1000, or up to 23 million in whole world [1]. Factors influencing readmissions of patients with SSD has been studied in a whole world [2,3]. There is not much information about prescription dispensing and substance abuse (SA) association with readmission rate.

**Aim:** To find association between regular prescription dispensing, SA and readmission rate among persons with SSD.

**Methods:** Latvia's health billing system's and Cause of death database data, collecting data about patients, who were hospitalized in one of Latvia's psychiatric hospitals 2014.-2016, with diagnose code F20-F29 (ICD-10). Data was gathered using MS Excel 2013, statistics were analyzed using IMB Statistics SPSS 23.

**Results:** A retrospective study included 3804 patients, 1733 (45,6%) men and 2071 (54,4%) women, mean age 45 (SD = 14,35). All patients were in Psychiatric registry. 311 were in SA registry and 3493 were not. Results elucidated that average readmission rate was higher among persons who were in SA registry (4,40), comparing with persons whose were not (3,47) (p < 0,001). Mean all dispensed prescriptions during 24 months was higher among persons who were in SA registry (18,59), comparing with persons, who were not (18,28) without significant difference (p = 0,737). Mean dispensed prescription rate in a month was higher among persons, who were in SA registry (1,98), comparing with persons, who were not (1,68) (p < 0,05). There was significant medium correlation between readmission rate and dispensed prescription rate in month (R = 0,682, p < 0,001). Correlation between hospitalization rate and average all dispensed prescriptions during 24 months was negligible (R = 0,110, p < 0,001).

**Conclusion:** Patients with SSD who were in SA registry dispensed prescription more often. Patients who were in SA registry were rehospitalized more often. There is association between readmission rate and dispensed prescriptions in a month.

**References**

1. http://www.who.int/en/news-room/fact-sheets/detail/schizophrenia

2. Hung YY, Chan HY, Pan YJ. Risk factors for readmission in schozphrenia patients following involuntary admission. PloS One J. 2017; 12(10): e0186768

3. Thongkam J, Sukmak V, Mayusiri V. Predicting Schizophrenia ar Risk of Readmissions in the Sort- and Long-Term using Decision Tree Model. KKU Researeh Journal. 2016; 21(3): 91-103

**PS043 Oral health evaluation among patients with schizophrenia using General Oral Health Assessment Index (GOHAI)**

Irena Ognjanovic’^1^, Dragana Ignjatovic’ Ristic’^1,2^, Strahinja Milenkovic’^3^

^1^*Faculty of Medical Sciences; University of Kragujevac,*^2^*Department of Psychiatry; Clinical Center Kragujevac,*^3^*Faculty of Engineering; University of Kragujeva E-mail address: irena.ognjanovic@gmail.com (Irena Ognjanovic*’*)*

**Introduction:** Studies have reported that the oral health status of people suffering from schizophrenia was poor compared with healthy population. They pay less attention to the presence of conditions such as dental caries and periodontal disease. If left untreated, these conditions can lead to partial or total tooth loss, thus compromising nutrition and general physical health.

**Aim:** The aim of this study was to evaluate the psychometric characteristic of the Serbian version of the GOHAI (General Oral Health Assesment Index) in the representative sample of patients with schizophrenia, to evaluate oral health, and to determine strategi

**Methods:** This study was performed among 40 patients with schizophrenia. The average age was 44.37 years (SD = 11.53). Patients had completed two-part questionnaire: first was consisted of socio-demographic questions, questions related to general health, oral health, nutrition and bad habits. Second part was GOHAI, composed of 12 items divided in three dimensions that address physical function, pain and discomfort, and psychosocial aspects.

**Results:** More than 40% of respondents brush their teeth not once a day. Patients who have been treated due their mental disease more than 10 years significantly more rarely brush their teeth, but have the highest GOHAI score (p < 0.05). Patients who smoke more than 20 cigarettes a day have significantly higher scale score (p < 0.05). Approximately 60% of respondents almost never visit their dentist. People with higher education level seem to visit their dentist more often, brush their teeth more often and express satisfaction with their oral health. There is a signifficant correlation between material status of patients and brushing-teeth frequency.

**Conclusion:** This study showed correlation between the duration of mental illness, bad habits, brushing-teeth frequency, frequency of dental visits, education level, economic status and oral health satisfaction.

**PS048 Traveler's diarrhea – What is the incidence in a post-travel context?**

Ana Teresa Vieira da Costa Tavares^1^, Margarida Ferreira Sousa^1^, Ana Luísa Vilaça Pinheiro^1^, Ana Filipa Martins Gonçalves^1^, Ana Leonor Rei da Cruz Escaleira^1^, Ana Manuela Moreira Azevedo^1^, Beatriz Bravo Peixoto^1^, Dinis Bento Loyens^1^, João Pedro Almeida Cor

^1^*Instituto de Ciências Biomédicas Abel Salazar - Universidade do Porto E-mail address: teresa25tavares@gmail.com (Ana Teresa Vieira da Costa Tavares)*

**Introduction:** Traveler's diarrhea is an infectious disease with a significant incidence, even though it seems to be decreasing. Several risk factors for traveler's diarrhea have been identified, namely the destination, place of stay and where people eat. [1,2]

**Aim:** Characterizing the Portuguese traveling population that has been to a traveler's consult in CHP (Centro Hospitalar do Porto) and identifying traveler's diarrhea incidence and risk factors.

**Methods:** This is a prospective cohort study. It was conducted from November 21st 2017 to April 10th 2018. The study population consisted of adults that went to a traveler's consult in CHP and to a tropical or subtropical destination. During the consult, the subjects signed an informed consent form and provided their e-mail, phone number, destination and travel dates. After their travel, they were asked to fill an online questionnaire. The results were analyzed using SPSS and Data Science.

**Results:** The incidence of traveler's diarrhea was 21,4%. Most people stayed in hotels and were traveling for holidays or work. The travels lasted between 3 and 86 days. The advice given on the consult was followed by 97,25%. In most cases, the diarrhea lasted between 1 and 2 days. Through Data Science, we found an association between traveling for pleasure and following the consult's advice (weight = 100), between following the advice about water and food simultaneously and not acquiring traveler's diarrhea (weight = 26), and between eating in restaurants and not having diarrhea (weight = 59).

**Conclusion:** Our incidence of diarrhea and its duration is similar to that found in other studies. The association between following advice on food and water reinforces the idea that these are the most important vectors in disease transmission. In future studies, it would be important to have a bigger sample to focus on the risk factors and on the associations found with Data Science but not with statistics.

**References**

1. Steffen, Robert MD. Epidemiology of travelers’diarrhea, Journal of Travel Medicine, Volume 24, Issue suppl_1, 1 April 2017, Pages S2–S5

2. Steffen, Robert MD. Epidemiology of travelers’diarrhea, Journal of Travel Medicine, Volume 41, Issue Supplement_8, 1 December 2005, Pages S536–S540

**Acknowledgements**

We would like to thank Dr^a^ Maria João Gonçalves, Drª Sandra Xará, Doutor José Sousa and Dr^a^ Celina Gonçalves

**PS064 The assesment of physical activity among Moroccan medical students**

Oumaima Outani^1^, Abdelkader Jalil El Hangouche^2^, Taoufiq Dakka^2^

^1^*Faculty of Medicine and Pharmacy of Rabat, University Med V Rabat, Morocco,*^2^*Laboratory of Physiology, Faculty of Medicine and Pharmacy of Rabat, University Med V Rabat, Morocco E-mail address: outani.oumaima@gmail.com (Oumaima Outani)*

**Introduction:** According to the World Health Organization (WHO) physical inactivity is a global public health problem and approximately 3.2 million deaths each year are attributable to insufficient physical activity, [1] however this problem was poorly investigated among Moroccan university students.

**Aim:** The aim of our study is to assess the level of physical activity among Moroccan medical students

**Methods:** A cross-sectional study with 150 Moroccan healthy medical students was conducted in Moroccan Faculty of Medicine. The mean age of the sample was 21.3. Percentage of women was 61%.

The International Physical Activity Questionnaire was used to measure the physical activity (IPAQ) [2];

To avoid any unusual interference the study was performed far from examination period.

**Results:** The IPAQ levels were low (<600 MET-min/Week) in 15.9% of the students, moderate (a minimum of at least 600 MET-min/week) in 76.8% of the students and in 7.3% the IPAQ levels were high (a minimum of at least 3000 MET-min/ week). Higher levels of physical activity were related to regular practice and male students.

**Conclusion:** We concluded that 96.3% of our sample didn’t reach a high level of physical activity according to IPAQ, while it is the high level that is associated with greater health benefits, and the low level is related to greater risk factors of heart diseases, diabetes, and psychological diseases.

**References**

1. World Health Organisation: Global Strategy on Diet, Physical Activity and Health

2. Guidelines for Data Processing and Analysis of the International Physical Activity Questionnaire (IPAQ) - Research Gate

**PS067 How endoscopic submucosal dissection for gastric lesions is being implemented: results of an European inquiry**

Miguel Araújo Martins^1^, Pedro Pimentel Nunes^1,2,3^, Diogo Libânio^2^,Marta Borges Canha^1^, Mário Dinis Ribeiro^2,3^

^1^*Departamento de Fisiologia e Cirurgia Cardiotorácica, Faculdade de Medicina da Universidade do Porto,*^2^*Serviço de Gastroenterologia, Instituto de Oncologia do Porto,*^3^*CIDES/CINTESIS Faculdade de Medicina da Universidade do Porto E-mail address: miguel.pedro96@gmail.com (Miguel Pedro Araújo Martins)*

**Introduction:** Our previous study in 2010 focusing on how endoscopic submucosal dissection (ESD) was established in European countries suggested that this technique was performed at few centers, with most endoscopists performing a low number of procedures [1].

**Aim:** The aim of this study was to determine the widespread use of ESD in the treatment of gastrointestinal superficial lesions, 8 years later.

**Methods:** European endoscopists (n = 153) that published articles related with endoscopic mucosal resection and submucosal dissection between 2005 and 2017 were asked to complete an online survey from December 2017 to February 2018.

**Results:** A total of 33 European endoscopists completed the survey (22%), of which 42% reported that ESD was performed in their centre (93% in the stomach; 71% in the colorectum; 64% in the esophagus and 14% in the duodenum). In 2016, each endoscopist had treated a mean of 18 gastric, 17 esophageal and 44 colon and rectum lesions. In the stomach, lesions were located mainly in the antrum (40%), while colorectal lesions were located more commonly in the rectum (57,5%). Dual knife was the most used in incision and in circumferential/ mucosal dissection (40%), followed by Flush and Hybrid-knife (13% each). The majority of resected lesions were Paris classification IIa (> 80% in every location). En-bloc resection rates were: 95% in the stomach; 98,5% in the esophagus and 85% in the colorectum. Complete resection was achieved in 90%, 85%, and 80%, respectively. The most frequent post-resection histology in the upper gastrointestinal tract was intramucosal adenocarcinoma (stomach 40%; esophagus 42,5%); in colon and rectum the majority of lesions were adenomas (55%), and only 10% had superficial submucosal invasion. Major complications (perforation or major bleeding) occurred more often in colorectal ESD lesions (4%, vs. 1% of esophagogastric cases)

**Conclusion:** ESD seems to be performed by a great number of centres and endoscopist. Our results suggest that ESD is being successfully implemented in Western countries, achieving a good rate of efficacy and safety according to European guidelines, with the exception of colorectal ESD that appears to be used more often than necessary [2].

**References**

1. F. Ribeiro-Mourão, P. Pimentel-Nunes, M. Dinis-Ribeiro. Endoscopic submucosal dissec0on for gastric lesions: results of an European inquiry. Endoscopy 2010; 42: 814–819

2. P. Pimentel-Nunes. Endoscopic submucosal dissec0on: European Society of Gastrointes0nal Endoscopy (ESGE) Guidelines. Endoscopy 2015; 47: 829-854

**PS068 Colonization of extended-spectrum beta-lactamase-producing bacteria (ESBL) in hospitalized preterm infants**

Miloš Jovic’evic’^1^, Željana Brankov^1^

^1^*Institute of Microbiology and Immunology, School of Medicine, University of Belgrade, Belgrade, Serbia E-mail address: jovicevic.milos@gmail.com (Miloš Jovic*’*evic*’*)*

**Introduction:** Extended-spectrum b-lactamase (ESBL) producing enterobacteria are a significant cause of healthcare associated infections. In preterm infants, due to prolonged hospitalization and frequent use of antibiotics, the risk of colonization with multiresistant bacteria including ESBL producing bacteria is higher.

**Aim:** The aim of this study was to assess the carriage rate of ESBL-producing enterobacteria in hospitalized preterm neonates, and to determine antimicrobial susceptibility pattern of resistant isolates.

**Methods:** The study included 30 randomly selected premature infants hospitalized at the Institute for Neonatology, Belgrade, Serbia. Duration of hospitalization was 3 to 178 days. Patients’ rectal swabs were taken in the same day and they were inoculated onto Chrom ESBL agar (bioMerieux, France). The isolated colonies were identified with API 20E (bioMerieux, France). Confirmation of ESBL production and antimicrobial susceptibility testing was done following EUCAST 2018 recommendations.

**Results:** Colonization was observed in 23 patients: 7 had *E. coli*, 7 had *K. pneumonia*, 2 had *A. baumannii* and 5 had *E. coli* and *K. pneumoniae*. The overall frequency of ESBL producing bacteria was 60%: 100% of E. coli strains and 30% of K. pneumonia strains. ESBL positive isolates were uniformly resistant to III and IV generation cephalosporins and ampicillin and susceptible to various degree to amikacin (94%), amoxicillin clavulanic acid (94%) and sulfamethoxazole-trimethoprim (100%). Nine patients were colonized with carbapenem-resistant *K. pneumoniae* susceptible to amikacin. Both *A. baumannii* strains were resistant to all tested antibiotics except to ampicillin with sulbactam (CLSI breakpoints used).

**Conclusion:** Our results showed that in preterm neonates the incidence of gut colonization with resistant bacteria is alarmingly high. Especially worrisome is the finding of high rate of carbapenem-resistant *K. pneumoniae*, and multidrug resistant *A. baumannii*. These results imply the necessity of further more thorough analysis of colonization with resistant bacteria in this group of patients.

**PS080 The impact of flavoured mineral water drinks and sugar substitutes on exogenic erosion of teeth enamel**

Anna Lewandowska^1^

^1^*Faculty of Medicine and Dentistry, Medical University of Warsaw E-mail address: ja.anialewandowska@gmail.com (Anna Lewandowska)*

**Introduction:** An increase in the consumption of soft drinks based on mineral water has been observed in recent years. Various reports claim that such drinks constitute high risk for the development of dental erosion. Dental erosion is becoming a worldwide phenomenon, affecting from 31% to 74% children all over the world. Flavoured mineral waters are still considered as a “healthy” alternative to carbonated soft drinks. However, in the composition of flavoured mineral water there are certain ingredients such as acidity regulator or fruit acid which change its properties making a mineral water drink erosive.

**Aim:** The aim of this study was to determine the influence of flavoured mineral water drinks and sugar substitutes solutions on the enamel dissolution.

**Methods:** Flavoured water soft drinks (strawberry, cherry, raspberry, apple, still water) and sweeteners solutions (xylitol, erythritol, stevia, glucose – fructose) were tested. Hydroxyapatite powder was incubated with tested solutions to determine the following parameters: pH, titrable acidity the concentration of phosphorus.

**Results:** The incubation of hydroxyapatite with flavoured mineral water caused statistically significant washout of phosphorus comparing to citric acid.

The flavoured mineral waters differ by the titrable acidity parameter with the highest value for the strawberry (6,71 ± 0,05 ml) and the lowest for the apple(4,52 ± 0,10 ml) mineral water drink. The results of phosphorus release from the hydroxyapatite show that the apple mineral water causes the lowest apatite dissolution (16,9 ± 1,27 μg/1 mg of hydroxyapatite) and the strawberry mineral water causes the highest apatite dissolution (25,2 ± 3,34 μg/1 mg of hydroxyapatite).

**Conclusion:** Consumption of flavoured mineral waters constitutes a very high risk of enamel erosion. The amount of dissolved hydroxyapatite was dependent on the titrable acidity of the drink. There are no significant differences in erosive potential of non-cariogenic sweeteners (xylitol, stevia, erytritol) and commonly used glucose-fructose syrup.

**PS086 Estimation of the sociodemographic and psychopathologic factors in children and adolescents with conduct disorder**

Arpad Rajsli^1^

^1^*Faculty of Medicine Novi Sad, University of Novi Sad E-mail address: rajsli.arpad@gmail.com (Arpad Rajšli)*

**Introduction:** Conduct disorders are characterized by a repetitive and persistent pattern of dissocial, aggressive or defiant conduct, which can lead to major violation. It is more common in males. As one of the risk factors, below average intelligence quotient is connected with conduct disorder. These disorders belong to “externalising disorders”, so it manifests with externalising psychopathological symptoms.

**Aim:** The aim is to analyse sociodemographic and psychopathologic factors in children and adolescents with conduct disorder, inpatients of the Department of the Child and Adolescent Psychiatry in the Clinical Centre of Vojvodina.

**Methods:** The study was conducted by analysing archival data and using medical documentation. The data was drawn from medical documentation and clinical data of inpatients from 1.9.2012. until 30.9.2017. The clinical sample is made of 95 patients (55 male and 40 female patients) aged from 10 to 19.

**Results:** After using statistical analysis is found that 57.9% are male, 44.2% are aged between 14 and 16, and with mother lives 35.8%. Even if statistically significant difference exists from the test result, the number of the patients with average intelligence quotient is higher than the below average's. There is found statistically significant difference between existing and the beforehand defined layout of relative frequencies of psychopathologic symptoms.

**Conclusion:** The sample is mostly male, in middle adolescence and most of them live with their mother. The biggest part of the patients belongs to the average category of intelligence quotient. There are more patients than expected with both, internalising and externalising, psychopathologic symptoms.

**PS087 Comparison of cardiovascular risk factors, prevalence of stable angina and acute coronary syndrome depending on patients’ age**

Izabela Z.ak^1^, Katarzyna Jasin'ska^1^, Anna Kominko^1^, Łukasz Wiewiórka, MD^1^, Maciej Nizio^1^

^1^*Jagiellonian University Medical College,*^*2*^*Students’ Scientific Group at Interventional Cardiology, Department in John Paul II Hospital in Cracow, Institute of Cardiology, Jagiellonian University Medical College,Head: Prof. Krzysztof Żmudka MD PhD E-mail address: iz.zak.mail@gmail.com (Izabela Z*.*ak)*

**Introduction:** Coronary artery disease is a paramount problem of the aging population. Depending on presentation and risk factors the treatment may differ markedly.

**Aim:** The aim of the study was to assess differences in coronary artery disease presentation depending on patients’ age (under/over 75 yo) and to compare cardiovascular risk factors in those ranges.

**Methods:** 1261 patients admitted in 2015 to John Paul II hospital in Cracow due to ischemic heart disease were divided into two groups: under and over 75 yo.

Risk factors of: hypertension, smoking, hyperlipidemia, chronic kidney disease (CKD) and diabetes were determined and then compared in both groups. Prevalence of stable angina (SA) and acute coronary syndrome (ACS) (divided into UCAD, NSTEMI, STEMI) were compared. The data was collected retrospectively.

**Results:** The first group (<75yo) had n = 927 patients and the second (≥75yo): n = 334. In both groups the most common diagnosis was SA (<75 yo: 53,89%, ≥ 75 yo: 54,52%; p = 0,84325). There was a significant difference in NSTEMI prevalence - in the older group it was more common (n = 139, 15% vs n = 73; 21,99%; p = 0,00357). The research did not show differences in presentation of other types of coronary artery disease (SA, ACS and separately UCAD, STEMI).

In risk factors the study indicates significant difference in smoking (<75yo: 18,18% vs ≥75yo: 4,20%, p < 0,001), CKD (GFR < 60) (17,60% vs ≥ 47,74%, p < 0,001) and diabetes (30,87% vs 42,51%, p < 0,0012). The differences in hypertension and hyperlipidemia prevalence did not reach the level of significance.

**Conclusion:** The research shows that in the older age range (≥75yo) NSTEMI occurred more frequently than in the first group (<75yo). However, smoking as a risk factor was more common among younger patients. In the older group, diabetes and CKD were more prevalent, which can be attributed to the fact that CKD is usually a complication of diabetes.

**PS092 Analysis of consumption of drugs affecting bone structure and mineralisation in The Republic of Serbia in the period 2013 – 2016**

Djurdja Cvjetkovic^1^, Boris Milijasevic^2^

^1^*Faculty of Medicine, University of Novi Sad,*^2^*Department of Pharmacology, Toxicology and Clinical Pharmacology, Faculty of Medicine, University of Novi Sad E-mail address: dj.cvjetkovic@gmail.com (Djurdja Cvjetkovic)*

**Introduction:** According to the definition, osteoporosis is a progressive, skeletal and metabolic bone disease, characterized by loss of bone mass, microarchitectural deterioration of bone tissue, enhanced bone fragility and a consequent increase in risk of fracture.

**Aim:** Measuring the consumption of drugs affecting bone structure and mineralisation over the period 2013 to 2016 in Serbia and comparing results with consumption of forementioned drugs in three Scandinavian countries: Norway, Finland and Denmark.

**Methods:** The consumption of drugs is estimated using the Anatomical Therapeutic Chemical/Defined Daily Dose (DDD) methodology. The amount of total drugs consumed has been expressed in the number of Defined Daily Dose on 1000 inhabitants per day (DDD/1000 inhabitants/day).

**Results:** In Serbia the consumption of bisphosphonates predominates and their share in the total consumption of M05B drugs represents around 75%. Bisphosphonates make around 40% in Finland and around 80% in Norway and Denmark. In the group of bisphosphonates the most frequently used drug in Serbia in 2013 is alendronic acid. However, in 2016 the consumption of ibandronic acid takes the lead. In country comparisons, the alendronic acid has the highest rate of consumption amongst bisphosphonates.

The consumption level of other bisphosphonates and strontium ranelate in Serbia is constantly low compared to other drugs. The same applies to Norway and Denmark, with the exception of Finland where except alendronic acid, zolendronic and risedronic acid are also consumed. In comparison to the Scandinavian countries, the consumption of denosumab in Serbia is multiple times lower. There is an upward trend of denosumab's consumption registered in all four countries in the examined period.

**Conclusion:** The consumption of drugs affecting bone structure and mineralisation in the examined time period in Serbia compared to countries with well developed pharmacotherapeutical practice is three to six times lower. Furthermore, there is a difference concerning the structure of proscribed and consumed drugs.

**PS100 Evaluation of the antimicrobial efficiency of disinfectants in impression materials**

Maria João Azevedo^1^, Inês Correia^1^, Ana Portela^1^, Benedita Sampaio Mata^1,2,3^

^1^*Faculdade Medicina Dentária Universidade do Porto (FMDUP),*^2^*INEB – Instituto Nacional de Engenharia Biomédica;*^*3*^*I3S - Instituto de Investigação e Inovação em Saúde, University of Porto, Portugal E-mail address: mariajoaomazevedo@gmail.com (Maria João Maia Azevedo)*

**Introduction:** Cross-infection control in dental practice is a subject of great importance. Microorganisms can survive even within the impression material. It is essential to use a disinfectant that is effective in reducing the microbial load and, at the same time, allows the maintenance of the impression materials’ dimensional stability.

**Aim:** To evaluate the antimicrobial efficacy of the most commonly used disinfectants in alginate’ and addition silicone’ dental impressions.

**Methods:** To each participant, it was taken an impression in alginate and another in addition silicone, and the antimicrobial efficacy of the disinfectants MD520^®^ (Durr™), 1% sodium hypochlorite, 5.25% sodium hypochlorite and 3% hydrogen peroxide was evaluated by pour plate method and compared with water washing or no washing or disinfection (control). The results were converted to percentage of control and compared with a Friedman's test.

**Results:** Impressions in alginate had a higher microbial load than impression in addition silicone (p < 0.05). Washing the impressions with water reduced the microbial load by 11.72% on silicone and by 4.76% on alginate. There were significant differences between the antimicrobial efficiency of water washing and the different disinfectants evaluated (p < 0.001). In silicone impressions, the reduction of the microbial load was 99.960% with 3% hydrogen peroxide, 99,996% with 1% sodium hypochlorite, 99.998% with 5.25% sodium hypochlorite, and 99.996% with MD520^®^. In alginate impressions, the reduction of the microbial load was 99.997% with 3% hydrogen peroxide, 99.999% with MD520^®^, 99.998% with 1% sodium hypochlorite, and 99.999% with 5.25% sodium hypochlorite.

**Conclusion:** Our study showed that dental materials’ disinfectants present a much higher efficiency in reducing dental impression's microbial load than water washing, and consequently, should be used. It is therefore imperative to increase the disinfection literacy between dentists and dental prosthetics, demystifying that water washing is sufficient to significantly reduce the microbial load in dental impressions.

**PS104 Intestinal colonization with multidrug-resistant, ESBL or carbapenemase producing Enterobacteriaceae, in continuing-care facilities in the Northern region of Portugal**

Rita Sofia Conde Lopes^1^, Daniela Gonçalves^1,2,3,4^, Helena Ferreira^1,2,3^

^1^*Microbiologia - Departamento de Ciências Biológicas,*^2^*Faculdade de Farmácia da Universidade do Porto,*^3^*UCIBIO, Universidade do Porto,*^4^*ISAVE – Instituto Superior de Saúde, Amares E-mail address: rsclopes@hotmail.com (Rita Sofia Conde Lopes)*

**Introduction:** Population aging, namely in Portugal [1], creates a greater need for medium and long-term-care facilities, as those of the national Integrated Continuing Care Units (ICCU). Thus, a significant problem emerges about the appearance of multidrug-resistant microorganisms (MDR), especially Gram-negative, such as *Escherichia coli* (*E. coli*) and *Klebsiella pneumoniae* (*K. pneumoniae*) [2].

**Aim:** The objective of this work is to detect and characterize multi-resistant Gram-negative bacilli, as intestinal colonizers, in ICCU patients, with particular interest in extended-spectrum-b-lactamase (ESBL) and carbapenemase producing Enterobacteriaceae.

**Methods:** Forty fecal samples of 3 different ICCU of the Northern region, from the districts of Bragança (23), Porto (11) and Braga (6), were analyzed. b-lactam-antibiotic susceptibility test was performed according to the European Committee on Antimicrobial Susceptibility Testing (EUCAST) specifications. ESBL and carbapenemases were characterized by polymerase chain reaction (PCR).

**Results:** Fourty-four relevant intestinal isolates (19 *E. coli* e 19 *K. pneumoniae*) were identified in patients from the three ICCUs: 31 ESBL producers (15 *E. coli*; 12 *K. pneumoniae*) and 5 carbapenemase producers (all *K. pneumoniae*). Molecular characterization showed KPC-type and CTX-M group 1, particularly CTX-M-15 in intestinal isolates.

**Conclusion:** Thus, it can be concluded that intestinal colonization by ESBL and carbapenemases producers, in patients in ICCU, it is already a reality in Portuguese old and dependent people healthcare-facilities. We can deduce that these health-units function as effective vehicles in the spread of MDR bacteria and genes associated with resistance to antibiotics. As this study suggests, intestinal-colonization and spread of bacteria with acquired multidrug-resistance is a reality, and it is a major priority that population and health-units take serious infection control measures and that antibiotics are correctly used.

**References**

1. INE, O envelhecimento em Portugal - Situação demográfica e socio-económica recente das pessoas idosas. 2002.

2. Majumdar, S.S. and A.A. Padiglione, Nosocomial infections in the intensive care unit. Anaesthesia & Intensive Care Medicine, 2012. 13(5): p. 204-208.

**PS129 The use of anatomy computer-assisted learning towards a personalized study experience: The impact on medical students’ spatial abilities**

Stanislav Tsisar^1,2^, João Firmino-Machado^3,4^, Benedita Viana^1^, Marco Pinto-Sousa^1^, Raquel Sofia Santos^1^, Ricardo Cruz-Correia^2^, Maria Amélia Ferreira^1^, Bruno Guimarães^1,5,6^

^1^*Department of Public Health, Forensic Sciences and Medical Education, Faculty of Medicine, University of Porto, Porto, Portugal,*^2^*Center for Research in Health Technologies and Information Systems (CINTESIS). Faculty of Medicine, University of Porto, Porto, Portugal,*^3^*Unidade de Saúde Pública, ACeS Porto Ocidental, Porto, Portugal,*^4^*EPIUnit - Instituto de Saúde Pública, University of Porto (ISPUP), Porto, Portugal,*^5^*Department of Biomedicine, Faculty of Medicine, University of Porto, Porto, Portugal,*^6^*Physical and Rehabilitation Medicine Department, Centro Hospitalar de Entre o Douro e Vouga, Santa Maria da Feira, Portugal E-mail address: stastv94@gmail.com (Stanislav Tsisar)*

**Introduction:** The current medical curriculum reforms challenge anatomy's pedagogical approach, favouring the implementation of new strategies, such as Computer-assisted Learning (CAL). CAL provides an insight into students’ learning skills and concomitantly analyses their cognitive profiles, steering a personalized learning experience. Spatial abilities, a core skill in anatomy learning, have the potential to be addressed by CAL platforms.

**Aim:** Thus, a study was conducted to assess the influence of anatomy CAL training on students’ spatial abilities.

**Methods:** Medical students attending Musculoskeletal (MA) and/or Cardiovascular Anatomy (CA) courses were allocated in three groups (MA group, CA group, MA + CA group). Students’ baseline and post-training spatial abilities were assessed through Mental Rotations Test (MRT). The MRT score difference between the two moments (Delta MRT) was correlated with students’ characterization variables, while multiple linear regression models assessed the association between these variables and Delta MRT.

**Results:** After CAL training sessions, students’ spatial abilities improved (9,72 ± 4,785 vs. 17,05 ± 4,567, P < 0.001). Delta MRT score showed a correlation with the Musculoskeletal Anatomy training sessions in MA Group (r = 0.333, P < 0.001) and MA + CA Group (r = 0.342, P < 0.001), as well as the Delta MRT score correlated with the Cardiovascular Anatomy training sessions in CA Group (r = 0.461, P = 0.001) and MA + CA Group (r = 0.324, P = 0.001). Multiple linear regression models showed to be good predictors of the studied reality in all groups, showing an association between the amount of training and the improvement in spatial abilities.

**Conclusion:** CAL training in anatomy has an incremental dose-dependent effect on spatial abilities. The inclusion of CAL into the anatomy pedagogical context might improve students’ core skills, as well as help to understand the learning process itself (according to Learning Analytics principles [1,2]).

**References**

1. Siemens G. 2013. Learning analytics: The emergence of a discipline. Am Behav Sci 57:1380–1400.

2. Ferguson R. 2012. Learning analytics: Drivers, developments and challenges. Int J Tech Enhanc Learn 4:304–317.

**PS135 Variation in the severity of depression symptoms during different phases of the menstrual cycle: A pilot study**

Tejal Gapchup^1^, Aneesh Bhat^1^, Vaishali Korde^1^

^1^*Maharashtra University of Health Sciences, Nashik E-mail address: tejal.gapchup@gmail.com (Tejal Gapchup)*

**Introduction:** PMS (Pre-menstrual Syndrome) and PMDD (Pre-menstrual Dysphoric Disorder) are characterised by symptoms such as depression, mood lability, headache, breast tenderness, etc. [1]

The assessment of depression symptoms during different phases of the menstrual cycle could provide a glimpse of the underlying PMS or PMDD that may be silently affecting the quality of life of women.

**Aim:** The aim of the study was to assess the severity of symptoms of depression in the different phases of the menstrual cycle.

**Methods:** One hundred and twenty-three female students in the age group of 18–22 years with a regular menstrual cycle were part of this study.

Every participant responded to the Beck Depression Inventory (BDI) questionnaire [2] once in each of the three phases of the menstrual cycle- Follicular phase, Ovulatory phase and Pre-menstrual/Luteal phase.

Further, the severity of depression symptoms in each phase was determined by assessing the BDI scores. Friedman test with post hoc analysis was used to check if the severity of depression in the three menstrual phases was significantly different.

**Results:** The severity of depression varied significantly in the three phases of the menstrual cycle. (p < 0.001)

The severity of depression in the pre-menstrual phase was significantly higher than that in follicular (z = -6.002, p < 0.003) and ovulatory phase (z = -5.766, p < 0.003).

44% participants reported mild-severe depression symptoms in the pre-menstrual phase, compared to just 16% in the ovulatory phase and 10% in the follicular phase.

**Conclusion:** The results suggest that women experience more depression symptoms during the pre-menstrual phase, as compared to other phases. This could be a part of underlying conditions like PMS or PMDD, high suspicion of which could lead to early diagnosis and intervention, if required.

Being aware of these symptoms gives women the motivation to handle them better. This would lead to a more productive way of life.

**References**

1. Dickerson LM, Mazyck PJ, Hunter MH. Premenstrual syndrome. American family physician. 2003 Apr;67(8):1743-52

2. Beck AT, Steer RA, Carbin MG. Psychometric properties of the Beck Depression Inventory: Twenty-five years of evaluation. Clinical psychology review. 1988 Jan 1;8(1):77-100.

**Acknowledgements**

I would sincerely like to thank Mrs. Swati Raje for her tremendous help.

**PS145 Epidemiological investigation of tension-type headache among youth in Ukraine**

Ivanna Yaremchuk^1^, Oksana Yaremchuk^1^

^1^*Bukovinian State Medical University E-mail address: yaremchuk.cv@gmail.com (Ivanna Yaremchuk)*

**Introduction:** Epidemiological studies show a higher prevalence of headache among youth and especially - among medical students. Tension-type headache (TTH) is one of the most common types of headache by young people.

**Aim:** To study the prevalence, clinical characteristics and risk factors of TTH among Bukovinian medical students.

**Methods:** 146 students of Bukovinian State Medical University aged 18 to 26 years were interviewed by using specially designed questionnaires. The type of headache was determined according to the diagnostic criteria of the classification of the International Headache Society, 2013. All the students with TTH were controlled by complex clinical investigations. The intensity of headache was investigated by visual-analog scale. Results are expressed in numbers and percentage.

**Results:** The headache was founded by 82,2% (121) students. Among women the prevalence of headache was significantly higher than among men - 62.1% and 38.9% respectively. Among the students the most frequent headache (58.7%) was tension headache. According to the students’ responses the most prevalent causes of TTH were stress – 36%, sleep disturbance – 20%, and weather changes – 14%. The other causes included skipping meals – 11%, tea or coffee overuse – 8%, alcohol consumption – 6%, and menstrual cycle disturbances in 2% students. Level of physical activity assessment showed that only 6.3% of students did daily exercise, 26.0% did regular exercise twice-three times a week, 35.4% did not have any form of exercise; and the rest did mild to moderate form of exercise infrequently. Low physical activity was associated with higher prevalence of tension headache.

**Conclusion:** By means of the research we found high prevalence of tension-type headache among medical students. Students with headache might benefit from regular physical activity and low consumption of alcoholic drinks, while for migraine patients a low consumption of coffee should additionally be recommended. This problem needs further inquiry and active correction for improvement in quality of life, professional and personal succeeding of medical students.

**PS148 Is the assessment of smoking habitats with exhaled carbon monoxide level reliable in citizens of air-polluted regions? The multi-centered cross-sectional study**

Agnieszka Trynkiewicz^1^, Agnieszka Wachsmann^1^, Mikołaj Maga^1^, Maciej Janik^2^, Olga Chrza.stek^2^, Martyna Schönborn^1^, Małgorzata Ceben’ko^1^, Iwona Gregorczyk-Maga^1^

^1^*Jagiellonian University Medical College,*^2^*Medical University of Warsaw E-mail address: ag.trynk@gmail.com (Agnieszka Aneta Trynkiewicz)*

**Introduction:** The poor quality of air is one of the most important reasons for increased carbon monoxide level in exhaled air (eCO), just after smoking cigarettes. Even though the knowledge about high pollution in big towns, its’ influence on measurement of smoking habits by eCO has not been well studied yet.

**Aim:** The aim of this study was to asses influence of air pollution on measurement of smoking habits by eCO.

**Methods:** In this study participants were recruited in two biggest Polish air-polluted cities and one small unpolluted town. After filling the survey concerning smoking habits got examined eCO with Pico + Smokerlyzertool.

**Results:** 939 participants were recruited, 742 from big cities (171 smokers, 571 non-smokers) and 197 from small town (58 smokers, 139 non-smokers). Air pollution examination revealed the difference of average CO level in atmosphere in big cities (1212,3 μg/m3 and 1059,5 μg/m3) and small town (388,9 μg/m3).

In big cities smokers differences of eCO were observed only between joined occasional smokers (couple cigarettes per month) with light smokers (couple cigarettes per week) and joined regular smokers (1 pack per day) with heavy smokers (2 packs per day or more) (p < 0,001), when in small town amount of cigarettes was identifiable by eCO in every category: occasional vs light (p = 0,045), light vs regular (p < 0,001) regular vs heavy (p = 0,012).

In big cities passive smoker had significantly lower eCO than smokers (p < 0,001) but weren’t differentiable by eCO from rest of non-smokers (p > 0,05). In small town passive smoker were easily differentiable by level of eCO from smokers (p < 0,001) as well as form rest of non-smokers (p = 0,002).

**Conclusion:** Smoking habitats are easily measurable by eCO level in non-polluted areas. However in heavily air-polluted areas this method does not give reliable detailed results in assessing smoking habitats, it still can be used to differentiate smokers from non-smokers.

**PS153 Resistance to beta-lactam antibiotics in***Escherichia coli***and***Klebsiella pneumoniae***hospital isolates in the North of Portugal**

Diana Coelho Marques^1^, Daniela Gonçalves^1,2,3^, Helena Ferreira^1,2^

^1^*Microbiologia - Departamento de Ciências Biológicas, Faculdade de Farmácia da Universidade do Porto,*^2^*UCIBIO, Universidade do Porto,*^3^*ISAVE – Instituto Superior de Saúde, Amares E-mail address: cmarques.diana@gmail.com (Diana Coelho Marques)*

**Introduction:** Antibiotic resistance is a public health concern, especially in Portugal once infection rate is higher than the European average. Patients admitted to hospitals with bacteria resistant to antibiotics, are an added risk for dissemination, outbreaks and other complications in susceptible patients.

**Aim:** This retrospective work includes the study of *Escherichia coli* (*E. coli*) and *Klebsiella pneumoniae (K. pneumoniae*) isolates. It was intended to evaluate the presence of extended-spectrum-b-lactamases (ESBL), AmpC cephalosporinases and carbapenemases clinical-isolates, from a Portugal northern-region hospital.

**Methods:** Between 2010/2011, 76 isolates of *E. coli* and *K. pneumoniae* were collected from different biological products, from a hospital in Minho region. Clinical isolates were selected by the clinical pathology laboratory and frozen till characterization. Isolates with reduced susceptibility to one or more antibiotics tested were selected (n = 42). b-lactam-antibiotic susceptibility test was performed, according to the European Committee on Antimicrobial Susceptibility Testing (EUCAST) specifications. ESBL, AmpC and carbapenemases were studied by polymerase chain reaction (PCR).

**Results:** Of 42 clinical-isolates, 11 produced simultaneously ESBL and AmpC (n = 5 *E. coli* and n = 6 *K. pneumoniae*) and 31 were ESBL-producers (n = 16 *E. coli* and n = 15 *K. pneumoniae*). Results showed the prevalence of CTX-M-group1 (n = 32) and CTX-M-15 (n = 22) enzymes, followed by CTX-M-group9 (n = 4) and CTX-M-group25 (n = 3). The blaTEM (n = 28), blaOXA (n = 30) and blaSHV (n = 21) genes were detected. The blaampC (n = 1), blaCMY (n = 4), blaDHA (n = 5) and blaCIT (n = 1) genes for AmpC were detected.

**Conclusion:** CTX-M-group1 gene was found in all *K. pneumoniae*, followed by *E. coli*. Production of AmpC was less common than ESBL production. Our results showed similar phenotypes which can be explained by hospital clonal propagation. Hospitals represent a rapid and easy antibiotic-resistance dissemination system. This is an effort to demonstrate the risk of dissemination of b-lactamase-producing isolates, leading to eventual clinical complications and intestinal colonization with antibiotic resistant-bacteria able of spread even after patient discharge.

**Acknowledgements**

Laboratory of Microbiology of the Clinical Pathology Service of the Hospital de Braga

**PS157 Monitoring of bariatric patients using the growth effects method following the treatment of obesity**

Duda Patrycja^1^, Alicja Dudek^1^, Klaudia Proniewska^2^, Michał Wysocki^3^, Michał Pe¸dziwiatr^3^, Piotr Major^3^

^1^*Students’ Scientific Group of Telemedicine and Medical Informatics, Jagiellonian University Medical College, Krakow, Poland,*^2^*Department of Bioinformatics and Telemedicine, Jagiellonian University Medical College, Krakow, Poland,*^3^*2nd Department of General Surgery, Jagiellonian University Medical College, Krakow, Poland E-mail address: p.b.duda@gmail.com (Patrycja Beata Duda)*

**Introduction:** The medical application MyftinessPal has been implemented for monitoring nutritional habits of patients after bariatric surgery in the United States. Psychological studies prove the validity of self- efficiency in behavioural modifications. Therefore, constant monitoring, subsequent motivational alerts may improve treatment effects.

**Aim:** Relying on collected data and patients expectations obtained from questionnaires, to establish a telemedical tool to investigate the influence of constant monitoring and subsequent motivational alerts for post-operative effects in the bariatric patients.

**Methods:** The prepared application comprises patient communication centre, data transfer module, electronic patient records, central data management and data repository with a comprehensible interface. The method consisted of patients continuously monitored during a 6-month period along with typical follow-up visits, and a control group of retrospective patients, who have not been included in the follow-up the program, though they participated in scheduled follow-up visits at 1 and 6 months postoperatively.

**Results:** The study continues - several regular users are still exploiting the application. The second follow-up is being scheduled in June 2018, during the meeting questionnaires for will be dispensed to obtain feedback from participants of the study.

**Conclusion:** During approximately one year of preparations and observations, several conclusions and limitations have appeared e.g. technical incompatibility, financial for server utilization, RODO actualization. After second follow-up we would like to compare patients’ expectations collected from questionnaires to the actual impression of participants of the study. Nevertheless, we believe that our clinical study should contribute to obtaining better treatment effects in patients after bariatric surgery and help in the development of an appropriable platform for monitoring of bariatric patients.

**PS183 Meibomian gland dysfunction and its association with digital display use in young adults**

Ieva Alisauskaite^1^, Egle Danieliene^1,2^, Saulius Galgauskas^1,3^

^1^*Vilnius University, Faculty of Medicine, Vilnius, Lithuania,*^2^*Ltd. “Akiu Gydytoju Praktika”, Vilnius, Lithuania,*^3^*Vilnius University Hospital Santaros Klinikos, Vilnius, Lithuania E-mail address: ieva.alisauskaite95@gmail.com (Ieva Ališauskaitë)*

**Introduction:** The use of digital displays is one of the risk factors of dry eye disease (DED). The leading cause of DED is Meibomian gland dysfunction (MGD), which causes rapid tear film break up. Meibomian gland (MG) loss, the key component of MGD, is strongly associated with aging. However, MG loss may occur in children, but there are only few studies conducted in adolescent populations. Besides, data about the influence of digital displays in MGD is controversial.

**Aim:** To examine morphological and functional features of MG in young adults, and determine the association with duration of digital display use.

**Methods:** Prospective study carried out in Lithuania. 54 eyes of 27 volunteers (aged 18–25) were examined. Volunteers responded to SPEED questionnaire about dry eye symptoms and questionnaire about the use of digital displays. Examinations included the non-invasive tear break-up time (NITBUT), MG area of loss measurement using meibography, meibum quality, MG expressibility, fluorescein tear break-up time (FTBUT), and corneal fluorescein staining.

**Results:** 42 eyes were included in the final analysis. 76,2% had dry eye symptoms. The average usage time of digital displays was 11,9 ± 3,5 hours/day. NITBUT and FTBUT were 12,4 ± 4,8 s and 6,4 ± 2,4 s accordingly, with FTBUT statistically shorter (P < 0,01). 59,5% of examined eyes had statistically significant MG loss in upper eyelids and 77,8% in lower eyelids. Mean area of loss in upper and lower eyelids were 12% and 18,2% respectively. Correlation was not observed between NITBUT, MG area of loss in upper and lower lids and hours spent on digital displays (r = 0.12, P > 0,05; r = 0.11, P > 0,05; r = -0.07, P > 0,05).

**Conclusion:** It is still unclear whether asymptomatic young people with MG loss, will have more pronounced symptoms with age. In this study MG loss and functional changes were found in many subjects, but there was no significant correlation with digital display usage time.

**Acknowledgements**

Scientific supervisors: Egle Danieliene, M.D., Ph.D., Saulius Galgauskas, M.D., Ph.D.

**PS189 Increasing the number of tests increases student's learning**

Catarina Dias^1^, Milton Severo^2^, João-Tiago Guimarães^3,4^

^1^*Faculty of Medicine of the University of Porto,*^2^*Departamento Ciências da Saúde Pública e Forenses, e Educação Médica, Faculdade de Medicina da Universidade do Porto,*^3^*Departamento de Biomedicina da Faculdade de Medicina da Universidade do Porto,*^4^*Departmento de Patologia Clínica, São João Hospital, Porto Portugal E-mail address: catarina.dias510@gmail.com (Catarina Alexandra Pinto Amarante Dias)*

**Introduction:** In Medical Education traditional multiple-choice written examinations are still used in a great scale to access student's knowledge.

**Aim:** This study aims to compare student's performance on same content when evaluated in a single test, two tests or four tests.

**Methods:** We analyzed our data using to the Classic Test Theory [1].

**Results:** Our results showed that the the Standard Error of Measurement (SEM) decreased with the number of tests (T1: 1,236; T2: 1,003; T4: 0,894), meaning that the error of the student's score attributed to the error of the test decreased. The Cronbach Coefficient Alpha seemed to increase with the number of tests (T1: 0,803; T2: 0,828; T4: 0,871), but when normalized for the number of questions (a∗), this variation was attenuated (T1: 0,867; T2: 0,828; T4: 0,871). The proportion of correct answers of the tests raised as the number of tests increased (b-trend = 0,026; P = 0,022), meaning that the students answered more items correctly when the number of evaluations increased. On the other hand, the discrimination index did not vary significantly with the raise in the number of evaluations (b-trend; P = 0.162), which means that the effectiveness in discriminating students of higher and lower ability remained the same.

**Conclusion:** These results might indicate that the increase in the number of evaluation tests enables students to obtain higher grades, whilst maintaining the same discrimination between lower and higher ability students.

**References**

1. De Champlain, Andre F. “A primer on classical test theory and item response theory for assessments in medical education.” Medical education 44.1 (2010): 109-117.

**PS206 The weather conditions and frequency of acute coronary syndromes. Retrospective analysis**

Karol Nowak^1^, Patrycja Mołek^1^, Konrad Ste¸pien’^1^, Aleksandra Włodarczyk^1^, Paweł Pasieka^1^, Magdalena Złowocka^1^

^1^*Student's Scientific Group, Department of Coronary Artery Disease and Heart Failure, Jagiellonian University Medical College E-mail address: karol.nowak@student.uj.edu.pl (Karol Witold Nowak)*

**Introduction:** The correlation between weather conditions and the amount of patients with acute coronary syndrome (ACS) is still unclear and even the newest studies do not sufficiently answer this question. A lot of physicians claimed that weather conditions could have an influence on the frequency of ACS.

**Aim:** The aim of the study was to assess an influence of weather conditions on the incidence of ACS.

**Methods:** 1171 patients (average age of 60.5 ± 10.8;327, 27.9% were females), who were hospitalized in 2002 and 2003 because of ACS were analysed. The data of patients were compared with weather conditions data (atmospheric pressure, temperature and rainfall), as well as daily changes of parameters obtained from the Institute of Meteorology and Water Management

**Results:** Most patients - 796 (68.3%), were admitted with the ST segment elevation myocardial infarction (STEMI). Patients were categorized into four groups due to the amount of ACS per day: I: 0–1, II: 2–3, III: 4–6, IV: 7–8 ACS/day. The results showed that the higher minimum (P = 0.02) and maximum (P = 0.03) atmospheric pressure, the higher maximum fluctuations of the atmospheric pressure (P = 0.04), the longer time of maximum difference of the atmospheric pressure (P = 0.02) and higher level of precipitation (P = 0.08) during the day before ACS occurrence were associated with higher number of ACS next day. Whereas, on the day of ACS, higher maximum (P = 0.03) atmospheric pressure correlated with the higher rate of ACS. There were no influence of the values of temperature on ACS either the day before or in the day of ACS.

**Conclusion:** The number of ACS on the particular day is significantly correlated with registered weather conditions that day as well as the day before. This analysis could be useful to create an application that could inform about higher probability of ACS each day.

**PS215 The influence of item modification on multiple choice questions statistics. Evaluation using “Peer assessment”**

Andrzej Nowak^1^, Sebastian Janiec^1^, Rafał Król^1^, Konrad Ste¸pien’^1^

^1^*Students’ Scientific Group of Teaching and Examining Methodology JUMC E-mail address: andrzej.jerzynowak@gmail.com (Andrzej Jerzy Nowak)*

**Introduction:** Test items are the most objective method of assessment of medical students’ knowledge. Many item modifications and technical errors have been reported in the literature. Their appearance can significantly affect student's results. “Peer assessment” is a novel strategy in medical education. It is connected with a greater contribution of students in the assessment of knowledge at universities.

**Aim:** To evaluate the impact of item modifications on test results using “Peer assessment” strategy.

**Methods:** The exam, consisting of 100 questions, was created by students of the 4th year of medicine. It matched the 3rd year clinical subjects exam blueprint. Then equal number of questions was assigned to each modification: adding clinical case vs simple question(1), “none of the above” statement(2), convergence of distractors(3), “often”, “always” statements(4), shift in Bloom's taxonomy(5), grammatical hints(6), one answer longer than others(7). Each exam version consisted of the same proportions of modified and non-modified items. No modification introduced knowledge based flaws to the items.

**Results:** 142 students of the 4th year of medicine (of 252) took part in the test. The average score was 50 points. Comparing the percentage of correct responses, the highest impact was observed for 4- 7.76% and 7- 6.02%, and the lowest in 3- 0.18%. Analyzing Discrimination Index(DI) the positive effects of modification 3(0,17 vs 0,22), 4(0,15 vs 0,20), and negative 2(0,8 vs 0,13), 6(0,17 vs 0,12) were found. Some modifications (1,3,5,6,7) increased variability of DI depicted by increased SD.

**Conclusion:** Modifications of test items have a big impact on scores. The impact of modifications on test statistics is counterintuitive and in our case did not match outcomes predicted by item writing guidelines.

**PS239 The predictive value of BOAH scale among patients of sleep disorders clinic at the Department of Sleep Medicine in Edinburgh**

Agata Gabryelska^1^, Łukasz Mokros^2^, Renata Riha^3^, Grzegorz Kardas^4^, Mateusz Niedzielski^1^, Michał Panek^4^, Piotr Białasiewicz^1^

^1^*Department of Sleep Medicine and Metabolic disorders, Medical University of Łódz*’, ^2^*Clinical Pharmacology Department, Medical University of Łódz*’, ^3^*Sleep Medicine Clinic, Royal Hospital, Scotland,*^4^*Department of Internal Medicine, Asthma and Allergy, Medical University of Lodz, Lodz, Poland E-mail address: m.niedzielski95@gmail.com (Mateusz Niedzielski)*

**Introduction:** Obstructive sleep apnea (OSA) is an increasingly frequent problem of developed countries. The polysomnography (PSG) is the golden standard of OSA diagnosis. Unfortunately, access to that examination is significantly limited. It is thus crucial to develop a simple and effective questionnaire to assess the severity of OSA.

**Aim:** To analyse the predictive value of an original questionnaire, BOAH, used for prioritising the PSG among patients with suspected OSA.

**Methods:** The study involved 275 patients referred to the Department of Sleep Medicine of the Royal Infirmary (Edinburgh, Scotland) between June 2015 and July 2016. The variables of a 5-point BOAH scale are: patient's age (≥50 – 1 point), BMI (≥ 35 kg/m2 - 2 points, ≥ 30 kg/m2 – 1 point), history of hypertension (1 point) and presence of apnea during sleep observed by a third person (1 point). The analysed sample was divided into three study groups depending on the OSA severity measured with the apnea-hypopnea index (AHI): mild (AHI ≥ 5), moderate (AHI ≥ 15), severe (AHI ≥ 30).

**Results:** In the group of patients with severe OSA (AHI≥30), basing on the Youden index, the best cut-off point was chosen, which was 4. The area under the ROC curve for he BOAH questionnaire was 0.776 (95% CI, 0,718–0,833). With the initial risk of 37%, the positive predictive value of the studied questionnaire was 75%, the negative predictive value – 78%, specifity – 89%, sensivity – 57%.

**Conclusion:** The BOAH questionnaire analysis on a group of Scottish patients showed high specifity. It implies that it may be used as a highly valuable tool to assess the risk of severe OSA and prioritise the PSG among these patients.

Surgery

**PS002 The effect of surgical treatment of morbid obesity on functional capacity and risk factors for cardiovascular diseases**

Andrea Manojlovic^1^, Andrea Malesevic^1^, Ivana Nedeljkovic^2^

^1^*Faculty of Medicine, University of Belgrade,*^2^*Department of Ergospirometry, Clinic of Cardiology, Clinical Center of Serbia E-mail address: andrea.m93@gmail.com (Andrea Manojlovic)*

**Introduction:** Morbid obesity is associated with elevated risk of cardiovascular disease (CVD). Bariatric surgery is the most effective weight loss method for morbidly obese patients.

**Aim:** To examine the effect of surgical treatment of morbid obesity on functional capacity and risk factors for CVD.

**Methods:** In a group of 56 morbidly obese patients BMI, functional capacity and risk factors for CVD were compared before and after laparoscopic gastric bypass surgery. BMI was derived from the patients’ weight and height. Functional capacity parameters (peak VO2 and VE/VCO2 slope) and systolic (SBP) and diastolic (DBP) blood pressure were obtained during ergospirometry testing. Data on risk factors was acquired from medical histories and laboratory analyses

**Results:** A statistically significant difference was found in peak VO2 (p < 0.0001) and VE/VCO_2_ slope (p = 0.003) before and 6 months after the surgery. Resting SBP significantly decreased (p = 0.017), as did SBP in maximal effort (p < 0.0001). Similar results were observed when comparing resting and maximal effort DBP (p < 0.0001 and p = 0.002, respectively) before and after the surgery. Risk factors for CVD improved – hypercholesterolemia (12,5% vs 9%), diabetes (37% vs 19,6%) and hypertension (48,2% vs 33%).

**Conclusion:** Morbidly obese patients showed an improvement of functional capacity, as well as in the risk factors for CVD 6 months following laparoscopic gastric bypass surgery.

**PS010 Time of anterior cruciate ligament injury**

Maja Stankov^1^

^1^*Department of Surgery, Faculty of Medicine Novi Sad, University of Novi Sad, Serbia E-mail address: majastankov93@gmail.com (Maja Stankov)*

**Introduction:** Anterior cruciate ligament (ACL) is the main stabilizer and most commonly injured knee ligament. ACL injuries typically occur during sports activities. Due to the incidence of ACL injuries and the fact that they most often involve young, active population, ACL injuries represent a significant epidemiological problem.

**Aim:** The aim of this study is to determine in which part of the training, the season, in what month, the day of week and part of the day ACL injuries usually occur in recreational and professional athletes.

**Methods:** The study included 1152 patients surgically treated at Orthopedic Surgery and Traumatology Clinic in Novi Sad from 2012 to 2017. Among these patients 671 are recreational athletes and 481 are professional athletes. After the analysis, and grouping the data collected, the data were statistically processed and then compared to the literature data.

**Results:** Significantly more athletes injured ACL in the middle of the training (p < 0.001). Recreational athletes most often get injured during the middle of the season, while professional athletes often get injured at the beginning of the season. There is a statistically significant difference in favor of injuries which happened during the middle of the season (p < 0.001). The highest number of injuries in recreational athletes was recorded in May, while in professional athletes this was the case in October. Sunday is the day with the highest number of injuries in recreational athletes while the critical day for professional athletes is Saturday. There is significantly high difference in favor of injuries which happened on Saturdays (p < 0.001). Both groups of athletes most often get injured in the afternoon.

**Conclusion:** The results obtained in this study show that most ACL injuries occur in the middle of the training, during the middle of the season, in October, on Saturdays and in the afternoon.

**PS020 Weight-loss prior to surgical treatment of obesity is associated with superior bariatric effect**

Tomasz Stefura^1^, Jakub Droś^1^, Artur Kacprzyk^1^

^1^*Students’ Scientific Group at 2nd Department of Surgery, Jagiellonian University Medical College, Cracow, Poland*

E-mail address: tomasz.stefura@gmail.com (Tomasz Stefura)

**Introduction:** Preoperative weight loss (PWL) is commonly recommended prior to bariatric surgery. Current knowledge on its influence on specific postoperative outcomes remains inconsistent.

**Aim:** Our aim was to assess the correlation between PWL and outcomes of bariatric surgery.

**Methods:** This retrospective analysis was conducted among patients who underwent primary Laparoscopic Sleeve Gastrectomy (LSG) or Laparoscopic Roux-and-Y Gastric Bypass (LRYGB) in four referral bariatric centers in Poland. PWL of 5% or above was considered a cut-off point. We investigated influence of PWL on primary endpoints: operative time, intra- and postoperative adverse events, length of hospital stay, readmissions and secondary endpoints measured six months after surgery: percentages of weight loss (%WL), excess weight loss (%EWL) and excess BMI loss (%EBMIL).

**Results:** 909 subjects were analyzed with median PWL of 3.33%. 349 (38.4%) patients achieved a PWL of ≥5%. Median LSG and LRYGB operative times were shorter in this group (75 vs. 90 min, p < 0.001; 100 vs. 120 min, p = 0.010). PWL did not influence the incidence of intraoperative or postoperative adverse events, readmissions and length of hospital stay. Greater follow-up rate was associated with PWL of ≥5% (p = 0.023). Patients from this group obtained higher median %WL (32.41% vs. 29.96%, p = 0.009), but there was no difference in %EWL and %EBMIL. An increase of 1% in PWL correlated with an increase of 0.35% in %WL (p < 0.05).

**Conclusion:** Greater PWL is associated with shorter operative time, but does not affect clinically relevant perioperative outcomes. Patients who obtain better PWL are more likely to achieve superior weight-loss after bariatric surgery.

**PS021 Trunk of Henle – a systematic review and meta-analysis of the surgical anatomy**

Oksana Skomarovska^1^, Tomasz Stefura^1^, Artur Kacprzyk^1^, Jakub Dros’^1^, Kaja Trzeciak^1^

^1^*Students’ Scientific Group at 2nd Department of Surgery, Jagiellonian University Medical College, Cracow, Poland E-mail address: skomarovska.o@gmail.com (Oksana Skomarovska)*

**Introduction:** Venous trunk of Henle is a blood vessel, which may be comprised of different tributaries. Surgeons recognized clinical significance of the trunk in various pancreatic, colorectal and hepatobilliary procedures. As a frequently varying anatomical structure, trunk is particularly prone to being injured. Currently there is no available study following Evidence Based Anatomy principles regarding the trunk of Henle.

**Aim:** Our aim was to find, gather and systematize available anatomical data concerning this structure, including its prevalence, diameter, length and tributaries.

**Methods:** A search using MEDLINE/PubMed, ScienceDirect, EMBASE, BIOSIS, SciELO and Web of Science databases was performed. Following data was extracted: method of anatomy assessment (cadaver dissection, radiological imaging or intraoperative assessment), geographical origin, study sample, known health status, prevalence of the trunk of Henle, its mean diameter and length, as well as the information on trunk tributaries.

**Results:** Our search strategy resulted in finding 38 records, including 2686 subjects. Pooled prevalence of the trunk of Henle was 86.9% with mean diameter of 4.2 mm. Only one study reported length of the trunk (10.7 mm). The most common type of venous trunk (56.1%) was the vessel comprised of three tributaries: gastric (right gastro-epiploic vein), pancreatic (most commonly anterior superior pancreaticoduodenal vein) and colic (most commonly superior right colic vein).

**Conclusion:** The trunk of Henle is a common variant of the portal circulation. It is a highly varying vessel, but most common type is gastro-pancreato-colic trunk. In surgical practice, this venous trunk constitutes a risk of bleeding and may be useful landmark during various abdominal procedures.

**PS026 The comparison of short-term postoperative complications including MINS (Myocardial Injury after Noncardiac Surgery) in patients who underwent endovascular or open aneurysm repair because of abdominal aortic aneurysm**

Radosław Kacorzyk^1^, Anna Iwan'ska^1^, Anna Gajdosz^1^, Aleksandra Kaszuba^1^

^1^*Students’ Scientific Society of Jagiellonian University Medical College, Poland E-mail address: rkacorzyk@interia.pl (Radosław Kacorzyk)*

**Introduction:** MINS is prognostically relevant myocardial injury due to ischemia that occurs during or within 30 days after noncardiac surgery and is an independent predictor of 30-day mortality. Endovascular aneurysm repair (EVAR), as a less invasive technique, has been accepted to be an effective alternative to open aneurysm repair (OAR) in patients with abdominal aortic aneurysm (AAA), especially in individuals with multiple comorbidities.

**Aim:** The aim of our study was to compare the frequency of MINS and other short-term postoperative complications between patients undergoing elective EVAR or OAR for AAA in St. Grande Hospital in Kraków.

**Methods:** The study group consisted of 423 patients (84% men), aged 72 ± 8,15 who underwent elective endovascular (142 patients-EVAR group) or open aortic repair (281 patients-OAR group) due to AAA. Patients with ruptured/symptomatic aneurysms were excluded from our study. MINS was defined as a postoperative troponin elevation (high-sensitivity TnT ≥30ng/l, high-sensitivity TnI Vidas ≥19 ng/l).

**Results:** The study groups were varied regarding the coexisting diseases. Patients in EVAR group were older than patients in OAR group (76,5 ± 7,8vs69,7 ± 7,3;p < 0,01), had higher prevalence of coronary artery disease (64,8%vs31%;p < 0,01), history of myocardial infarction or acute coronary syndrome (45,8%vs20,3%;p < 0,01) and congestive heart failure (40,9%vs8,9%;p < 0,01).

We observed statistically significant lower prevalence of short-term postoperative complications in EVAR group: pneumonia (0%vs5,7%;p < 0,01), sepsis (0,7%vs4,3%;p = 0,04), acute kidney injury (AKI) (4,9%vs12,1%;p = 0,02), multiple organ failure (MOF) (0,7%vs5,7%;p = 0,01) and intrahospital mortality (1,4%vs7,1%;p = 0,01). Nevertheless, MINS were definitely more common in EVAR group (28,4%vs19,6%;p = 0,03).

**Conclusion:** The frequency of the majority of short-term postoperative complications was higher in patients undergoing OAR and our findings are consistent with the previous studies on this topic. However, we observed higher frequency of MINS in EVAR patients. Further studies are needed to determine the benefit of doing troponin tests after EVAR and prognostic meaning of MINS in EVAR patients.

**PS044 Anatomical variants of carotid and main cerebral arteries – are they associated with intracranial aneurysms occurrence?**

Maciej Polak^1^, Iwona Kucybała^1^, Martyna Klis’^1^, Jakub Giliavas^1^, Kamil Krupa^1^, Jakub Polak^1^

^1^*Department of Radiology, Jagiellonian University Medical College, Kraków, Poland E-mail address: maciejpolak8@gmail.com (Maciej Polak)*

**Introduction:** Vascular anatomical variations are very prevalent in population, but their coexistence with life-threatening vascular malformations remains unclear.

**Aim:** The objective of this study was to assess the correlation between the presence of carotid and main cerebral arteries anatomic variants and the occurrence of cerebral aneurysms.

**Methods:** We analysed examinations of 194 patients who had undergone computed tomography (CT) of head with subsequent CT angiography of head and neck between September 2016 and November 2017 in the University Hospital in Cracow. The mean age of patients was 52.6 ± 19.6 years. 57.2% of them were females. Assessed parametres: the variation of the common carotid arteries (CCAs) origin, hypoplasia or duplication of any main cerebral artery, carotid artery kinking and the presence of cerebral aneurysms. Statistical significance was set at p < 0.05.

**Results:** 5.6% of patients had a cerebral aneurysm. Hypoplasia of at least one main cerebral arteries occurred in 34.5% of patients, the most frequently – vertebral artery (21.6%), P1 segment of posterior cerebral artery (8.2%) and A1 segment of anterior cerebral artery (5.2%). Duplication of at least one main cerebral artery was detected in 2.6%. Cerebral aneurysms occurred more often along with hypoplasia (p = 0.041; OR = 3.175) or duplication (p < 0.001; OR = 18.500) of at least one main cerebral artery. In 7.2% of cases, origin of CCAs created a true bovine arch and in 4.1% a so-called bovine arch. Patients with non-standard origins of CCAs were more likely to have cerebral aneurysms (p = 0.002; OR = 5.903). 27.3% of patients had ICAs affected by kinking. Kinking was more prevalent in patients ≥65 years (p = 0.001; OR = 2.926) and females (p = 0.027; OR = 2.083). Carotid kinking did not predispose to cerebral aneurysm formation (p = 0.378).

**Conclusion:** There is an evident correspondence between the presence of some vascular anatomic variants (abnormal origin of the carotid arteries, hypoplasia or duplication of main cerebral artery) and the occurrence of cerebral aneurysms.

**PS052 Effect of perioperative blood transfusion in gastric cancer prognosis**

Catarina Henriques^1^, Hugo Santos Sousa^1^, Jorge Nogueiro^1^, José Barbosa^1^, José Costa Maia^1^

^1^*Sao Joao Medical Center - Faculty of Medicine University of Porto, Department of Surgery E-mail address: catarinadhenriques@hotmail.com (Catarina Souza-Soares Dantas Henriques)*

**Introduction:** Surgical resection combined with extended lymphadenectomy is the recommended main treatment for gastric cancer and is often associated with significant perioperative blood loss requiring blood transfusion. However, the effect of these transfusions in prognosis remains controversial.

**Aim:** The aim of this study was to analyze the impact of perioperative blood transfusion (PBT) in recurrence and survival of gastric cancer patients, as well as identify risk factors for PBT.

**Methods:** Retrospective analysis of a prospective database (n = 637) with gastric cancer cases submitted to gastrectomy between January 2010 and December 2017, in an Upper GI Surgery Unit. We analyzed 398 patients that met the inclusion criteria for this study and 49 (12,3%) of those required PBT.

**Results:** PBT had a negative effect in OS (31,3 vs 64 months; p < 0,001), DSS (55,8 vs 78 months; p = 0,005) and DFS (50,1 vs 75,6 months; p < 0,001). When adjusted to pStage, perioperative blood transfusion was significantly associated to OS (HR 2,274; CI95% 1,503–3,440; p < 0,001) and DFS (HR 1,951; CI95% 1,088–3,499; p = 0,025). In univariate analysis, age (p < 0,001), BMI (p = 0,049), ASA (p = 0,001), presence of comorbidities (p = 0,012), tumor size (p < 0,001) and location (p = 0,001), pT (p < 0,001), LN ratio (p = 0,026), lymphatic invasion (p = 0,032), venous invasion (p = 0,012), pStage (p = 0,003), type of surgical approach (p < 0,001), lymphadenectomy type (p = 0,035), neoadjuvant treatment (p = 0,018) and resection margins (R) [p = 0,048] were significantly correlated with PBT. In multivariate logistic regression analysis, age (OR 1,06; CI95% 1,023–1,090; p = 0,001), type of surgical approach (OR 3,997; CI95% 1,827–8,747; p = 0,001), tumor location (proximal third: OR 4,096; CI95% 1,004–16,703; p = 0,049; middle third: OR 2,323 CI95% 1,157–4,662; p = 0,018) and pT (OR 3,015; CI95% 1,482–6,132; p = 0,002) were independent risk factors for PBT.

**Conclusion:** This study has shown a worse prognosis in gastric cancer patients that required perioperative blood transfusion. Strategies to reduce blood losses and to avoid blood transfusion should be implemented, especially in patients with the risk factors identified.

**PS058 Prognostic factors in node-negative advanced gastric cancer**

Eduardo Martins^1^, Hugo Santos-Sousa^1^, Jorge Nogueiro^1^, José Barbosa^1^, José Costa-Maia^1^

^1^*Sao Joao Medical Center - Faculty of Medicine University of Porto, Department of Surgery, Porto, Portugal E-mail address: ehdmartins@gmail.com (Eduardo Henrique Dias Martins)*

**Introduction:** It is well established that the existence of lymph node (LN) metastasis is the most important prognostic factor in advanced gastric cancer after curative gastrectomy. However, some patients have node-negative advanced gastric cancer. The identification of others useful prognostic factors may be important for the selection of patients who may benefit from more aggressive postoperative treatments.

**Aim:** So, our purpose is to identify the clinicopathological factors that influence the prognosis in node-negative advanced gastric cancer.

**Methods:** Retrospective analysis of a prospective database (n = 637) with gastric cancer cases submitted to curative intent surgery between January 2010 and December 2017, in an Upper GI Surgery Unit. In this study, were included 81 patients with node-negative stage T2-4 gastric cancer that met the inclusion criteria.

**Results:** Of the 81 patients, 33 (40,3%), 31 (38,3%) and 17 (20,9%) had T2, T3 and T4 tumors, respectively. Our recurrence rate was of 8,6% (7). The recurrence rate was 0%, 9,7% (all distant metastasis) and 23,5% (50% loco-regional and 50% distant metastasis) in T2, T3 and T4, respectively. In univariate analysis, macroscopic type (p = 0,007), pT (p = 0,001), peri-operative blood transfusion (p < 0,001) and lymphadenectomy type (p = 0,036) were significantly correlated with tumor recurrence. While tumor location (p < 0,001), pT (p = 0,028), peri-operative blood transfusion (p = 0,014) and age (p = 0,044) were significantly correlated with overall survival. In multivariate logistic regression analysis (forward stepwise conditional) macroscopic type [HR 3,25; CI 95% (1,227 – 8,606), p = 0,018] and peri-operative blood transfusions [HR 21,775; CI 95% (3,870 – 122,538), p < 0,001] were significantly and independently correlated with recurrence. Whereas peri-operative blood transfusion [HR 2,749; CI 95% (1,174 – 6,440), p = 0,02] was significantly and independently correlated with overall survival.

**Conclusion:** Macroscopic type and peri-operative blood transfusion reliably predict recurrence, whilst peri-operative blood transfusion reliably predict overall survival. In patients with these characteristics more aggressive postoperative treatments and timely follow-up should be considered.

**PS061 The clinical rationale for using polypropylene mesh for preventing dislocation of hip endoprostheses**

Mithilesh Bharadwaj Burra^1^, Sergei Maslennikov^2^,Maxim Golovaha^2^, Maxim Kozhemyaka^2^

^1^*Zaporozhye State Medical University,*^2^*Department of Traumatology and Orthopedics, Zaporozhye State Medical University E-mail address: mithil.bharadwaj@gmail.com (Burra Mithilesh Bharadwaj)*

**Introduction:** Today, Total Hip Replacement is one of the most successful surgical procedures in the field of orthopedic and trauma surgery. One of the complications of total hip arthroplasty is the dislocation of the femoral component. According to various authors, the frequency of dislocations ranges from 0.2 to 2% in which 16–59% of patients experience relapses.

**Aim:** The aim of our study is to prove the effectiveness of using polypropylene mesh to prevent dislocation of hip endoprostheses.

**Methods:** From 2015 to 2018, 21 patients 8 (38%) are female and 13 (62%) are male, with an average age of 58 years are included. In these patients, the instability of the acetabulum component was diagnosed near 35%, and closed dislocation after revision THA surgery – 33%. In these cases, patients underwent surgery for reinstallation of the acetabulum component with closure of the capsular defect with polypropylene mesh.

**Results:** All patients in the postoperative period were monitored for laboratory blood tests, C-reactive protein and a score on the scale of M. D’Aubigne and M. Postel. In the laboratory analysis, blood cells are normal but C-reactive protein is a bit decreased which indicate an attenuation of the acute phase of the inflammatory process, before 72 ± 6 and after 20 ± 2. None of the patients showed complications in the form of dislocations after using the polypropylene mesh. As for the M. D’Aubigne and M. Postel scale, an average score from 8–11 was observed in all patients after the operation.

**Conclusion:** Polypropylene mesh is absolutely bioinert in closure of the defect of the joint capsule and does not have an allergenic, toxicogenic and carcinogenic effect. The use of polypropylene mesh in clinical practice has shown good results of treatment, made it possible to prevent dislocations and further complications of the hip endoprostheses.

**PS085 Acute cholecystitis treatment – retrospective Study**

Sofia Moreira^1^, Fabiana Sousa^2^, Rui Costa^1,2^, Daniela Linhares^1,2^, José Costa Maia^2^, João Paulo Araújo Teixeira^1,2^, Luís Graça^1,2^, Renato Bessa Melo^1,2^

^1^*Faculdade de Medicina da Universidade do Porto,*^2^*Centro Hospitalar de São João E-mail address: sofiasousamoreira@gmail.com (Sofia Alexandra Sousa Moreira)*

**Introduction:** Acute cholecystitis (AC) is the second source of complicated intra-abdominal infections and represents one third of all surgical emergency hospital admissions. The Tokyo Guidelines aim to provide guidelines of diagnosis, severity stratification and treatment. Urgent cholecystectomy is the first-line treatment; in selected patients, antibiotics and cholecystostomy may be useful.

**Aim:** The aim of this study was to understand how treatment performed affects the outcomes of these patients, particularly readmission and mortality rates. We also intended to understand how co-morbidities and clinical presentation will affect the treatment performed and correlated outcomes. Another goal of our study is to evaluate if our service abides by international guidelines, particularly the Tokyo Guidelines, which recommend early cholecystectomy as treatment of choice for AC patients and preconizes conservative treatment and drainage as alternatives for surgically high-risk patients.

**Methods:** Retrospective study of patients with AC treated between January 1st 2012 and December 31st 2015 at a tertiary hospital. Data were collected by consulting patient's clinical file. Statistical analysis was performed using SPSS^®^ 25.0 and was based on Tokyo Guidelines 2018.

**Results:** 532 patients were admitted with AC: 339 were treated exclusively with antibiotics (group A); 67 with urgent cholecystostomy (group B); 126 with urgent cholecystectomy (group C). Group C patients increased by 20,9% between 2012 and 2015. Statistically significant differences were found concerning age, co-morbidity and clinical severity amongst the groups: group B patients were older, had more co-morbidities (diabetes mellitus, chronic kidney disease and cardiovascular disease) and greatest clinical severity. Statistically significant differences (p < 0,001) were found concerning length of hospital stay, which was shorter for group C.

**Conclusion:** Urgent cholecystectomy is the treatment of choice for AC. It is associated with shorter hospital stay and lowest readmission rates. Antibiotics and cholecystostomy may be considered for high-risk patients (Tokyo Guidelines grade III).

**PS095 Carotid artery stenosis: What about screening?**

Marta Cerqueira Silva^1^, Andreia Pires Coelho^2^

^1^*Faculdade de Medicina da Universidade do Porto,*^2^*Angiologia e Cirurgia Vascular, Centro Hospitalar de Vila Nova de Gaia/Espinho E-mail address: marta.cerqueirasilva95@gmail.com (Marta Alexandra Cerqueira Silva)*

**Introduction:** In Europe, cerebrovascular stroke causes 1.1 million deaths annually. It is the commonest cause of acquired disability in adults, with more than half of stroke survivors being dependent on others for everyday activities. [1] What if it could be preventable?

Screening is possible based on the rationale that the condition being prevented is importante and has a latent phase; there is a reliable screening test that is acceptable to the population in question; there is an accepted treatment for screen-positive patients and the intervention for screen-positive patients is cost-effective.

**Aim:** Assuming that patients aged > 80 years with asymptomatic stenosis do not benefit from carotid endarterectomy (CEA), the yield for finding patients with > 70% stenosis through unselected screening of patients aged < 80 years would be < 2%, which is not enough to be cost-effective or clinically effective. [1] Selective screening for CAS should be considered in patients with multiple cardiovascular risk factors such as increasing age (>65 years), tobacco smoking, peripheral arterial disease, diabetes, early onset atherosclerosis, hypertension, history of coronary heart disease and hyperlipidemia, based in the knowledge of the systemic nature of atherosclerotic disease. These were identified as independent predictors of a 50% CAS. [1]

**Methods:** All 60 patients submitted to CEA for asymptomatic CAS in the past two years in Hospital Santos Silva were retrospectively reviewed.

**Results:** Patients had a mean of 4 risk factors and 70% had other affected territories besides the cerebrovascular. 87% had at least 3 risk factors. This finding is in accordance with findings from literature and could lead to selecting groups in which screening is clinically relevant to prevent negative outcomes.

**Conclusion:** There may be room for a selective carotid artery stenosis since it's possible to identify subjects in high risk for stenosis which could favorably change the epidemiology of cerebrovascular stroke.

**References**

1. AR Naylor et al, Management of Atherosclerotic Carotid and Vertebral Artery Disease: 2017 Clinical Practice Guidelines of the European Society for Vascular Surgery (ESVS), Eur J Vasc Endovasc Surg (2017): 1-79

**PS103 Prevalence and anatomical characteristics of artery of Adamkiewicz: a meta-analysis**

Karolina Brzegowy^1^, Dominik Taterra^1,2^, Bendik Skinningsrud^1,2^, Przemysław Pekala^1,2^, Wan Chin Hsieh^2,3^, Roberto Cirocchi^4^, Jerzy Walocha^1,2^, Shane Tubbs^5^, Krzysztof Tomaszewski^1,2^, Brandon Michael Henry^2^

^1^*Department of Anatomy, Jagiellonian University Medical College, Kraków, Poland,*^2^*International Evidence-Based Anatomy Working Group, Kraków, Poland,*^3^*First Faculty of Medicine, Charles University, Prague, Czech Republic,*^4^*Department of Surgical Sciences, Radiology and Dentistry, University of Perugia, Italy,*^5^*Seattle Science Foundation, Seattle, Washington, USA E-mail address: karolina.brzegowy@gmail.com (Karolina Maria Brzegowy)*

**Introduction:** The artery of Adamkiewicz (AKA), also known as the great anterior radiculomedullary artery, is a major artery that originates from lumbar or intercostal arteries and joins the anterior spinal artery in the lower one-third of the spinal cord. AKA makes a major contribution to the blood supply to the anterior thoracolumbar spinal cord and iatrogenic injury or inadequate reconstruction of this vessel during surgery can result in postoperative neurological deficit due to spinal cord ischemia.

**Aim:** The aim of this study was to provide comprehensive data on the prevalence and anatomical features of the AKA.

**Methods:** Major electronic databases were thoroughly searched for studies eligible for inclusion. Additionally, reference search of included articles was performed. Data regarding study type, prevalence of the AKA, gender, number of AKA per patient, laterality, origin based on vertebral level, side of origin, morphometric data, and ethnicity subgroups was extracted and pooled into a meta-analysis.

**Results:** A total of 60 studies (n = 5,437 subjects) were included in this meta-analysis. Our main findings revealed that the AKA was present in 84.6% of the population. Patients most frequently had a single AKA (87.4%) on the left side (76.6%) originating between T8 and L1 (89%). AKA continued from the aorta to the anterior spinal artery in 76% of patients. The analysis showed a pooled mean diameter of AKA of 1.09 mm.

**Conclusion:** As the AKA is present in the majority of the population, caution should be taken during vascular and endovascular surgical procedures to avoid injury or ensure proper reconstruction. Iatrogenic injuries are partly due to a high degree of variability in the anatomical location and characteristics of the AKA. Therefore, all surgeons operating in the thoracolumbar spinal cord should have a thorough understanding of the anatomical features and surgical implications of an AKA.

**PS116 Decreased risk of acute pancreatitis after endoscopic trans-papillary intervention with guidewire techniques in patients with different types of major duodenal papilla**

Raja Raghupathy Rao Cherukuri^1^, Andrii Alexandrovich Steshenko^2^, Andrii Vladimirovich Klymenko^2^

^1^*Zaporozhye State Medical University,*^2^*Department of Faculty Surgery; Zaporozhye State Medical University E-mail address: rao.raja.95@gmail.com (Raja Raghupathy Rao Cherukuri)*

**Introduction:** Endoscopic trans-papillary interventions (ETI) serve as a cornerstone in the treatment of biliary tract diseases although leading to life-threatening complications such as acute pancreatitis (AP).

**Aim:** To determine the risk of AP in patients who had undergone ETI in correlation with different types of major duodenal papilla (MDP) as classified by R.H. Hawes, 2017[1].

**Methods:** We studied 746 patients who had undergone successful ETI. They were divided into two groups. Group A – 432 patients (57.9%; age– 51.96 ± 17.6; males– 45.83% and females– 51.16%) who had undergone ETI without the use of a guidewire and prophylactic stenting of the pancreatic duct. Group B – 314 patients (42.1%; age– 52.41 ± 17.6; males– 45.54% and females– 54.45%) who underwent ETI with guidewire and prophylactic stenting of pancreatic duct.

**Results:** 34 patients (7.87%) of group A and 11 patients (3.5%) of group B suffered from AP after surgery. 17 (50%) patients of group A with AP have ‘S-type’ MDP, 12 (35.29%) patients have ‘Shar-pei type’ and 5 (14.71) patients have ‘Direct type’. 5 (45.45%) patients of group B with AP have ‘S-type’ MDP, 5 (45.45%) patients have ‘Shar-pei’ type and 1 (9.09%) patient has ‘direct type’. Average levels of amylase in blood of patients, without structural changes in the pancreas in group A with ‘direct type’– 65.56 ± 19.09, ‘S-type– 86.73 ± 18.26 and ‘Shar-pei’ type– 97.86 ± 22.62 and that in group B with ‘direct type’– 62.74 ± 18.39, ‘S-type’– 68.27 ± 17.06 and ‘Shar-pei’ type– 79.86 ± 15.70

**Conclusion:** Occurrence of severe form of AP was higher in group A, especially in patients with ‘S-type’ papilla followed by those with ‘shar-pei’ type papilla. Serum amylase levels in group B were lower than in that of group A which is also reflected by the reduced number of post-procedural AP cases. The study also showed that the use of prophylactic stenting and guidewire usage has reduced the incidences of post-procedural AP.

**References**

1. How I cannulate the bile duct. Hawes R.H., Deviere J. (2018) Gastrointestinal Endoscopy, 87 (1), pp. 1–3. DOI - https://doi.org/10.1016/j.gie.2017.09.008

**PS122 Comparative effect of Ringer's solution and distilled water pharyngeal packing on postoperative sore throat after head and neck surgery: a randomized controlled trial**

Farshid Gholami^1^, Armin Shahlaee^1^

^1^*Azad University of Tehran E-mail address: shahlaeearmin@yahoo.com (Armin Shahlaee)*

**Introduction:** For reduction of sore throat after operation we must other do an operation or apply tracheal intubation or consider applying pharyngeal packing or use more pain killers after operation. The first two options are out of question. For the last option the application of nonsteroids pain killers has the side effect of reduction of regenerative tissue after operation. Opiums has also the side effect nausea, vomiting and hemodynamic disorders.

**Aim:** This study was performed to determine the comparative effects of ringer's solution versus water pharyngeal packing on postoperative sore throat after head and neck surgery.

**Methods:** In this interventional study that was performed as a randomized controlled trial, 220 patients undergoing head and neck surgery in Boali Hospital, Tehran, Iran were enrolled and randomly assigned to receive either ringer's solution or distilled water added to the pharyngeal packing before operation. The percentage of patients with sore throat was then determined and compared across the groups in three timeframes; during recovery room stay, 6 hours after surgery, and one day after surgery. Logistic regression was used to control for covariance among predictor variables.

**Results:** The two treatment arms were balanced with respect to age and gender distribution. We found that the rate of sore throat was higher for patients undergoing longer operative time. The percentage of patients with sore throat in the recovery room was 75.8% in the ringer's pharyngeal packing group versus 25.4% for the distilled water pharyngeal packing group (P < 0.001). This significant difference persisted even after correcting for the nuisance variable.; namely, length of the operation time (P = 0.05). For the observations after 6 hours, there were no significant difference between the two treatment groups.

**Conclusion:** Distille water pharyngeal packing following head and neck surgery decreases the rate of immediate postoperative pain compared to ringer's packing

**PS124 Impact of intrastromal corneal ring segments implantation on the quality of life of patients with keratoconus**

Anita Antunes Sousa^1^, Luís Miguel Gonçalves Torrão^2^

^1^*Faculdade de Medicina da Universidade do Porto,*^2^*Centro Hospitalar de São João E-mail address: anita.antunes.sousa@gmail.com (Anita Antunes Sousa)*

**Introduction:** Keratoconus is an ectatic disease, characterized by a progressive decrease in corneal thickness, leading to its cone-shaped protrusion. Thereby, it results in myopia and irregular astigmatism, affecting visual function and conditioning a significant loss of quality of life. Intrastromal corneal ring segments (ICRS) have been associated with improvement of visual acuity and corneal topography parameters in keratoconus, however little is known about the repercussion of ICRS on patient's perceived visual function.

**Aim:** To evaluate the influence of the implantation of ICRS on the quality of life of patients with keratoconus, through the National Eye Institute Visual Function Questionnaire-25 (NEI VFQ-25).

**Methods:** Descriptive retrospective study of 18 patients diagnosed with keratoconus, who underwent ICRS implantation between 2011 and 2017. The NEI VFQ-25 was administered, adjusted to the retrospective design of the study and to the portuguese language. The results were evaluated together with pre and postoperative data of best corrected visual acuity (BCVA), maximum curvature of the anterior surface of the cornea (Kmax), keratometric astigmatism (KA) and root mean square of coma aberration (RMS Coma).

**Results:** Statistically significant improvements were observed in BCVA (p = 0.001), from a preoperative median of 0.40 to 0.65, and in the composite score of the NEI VFQ-25 (p = 0.001), from 70.21 to 80.51 postoperatively. Additionally, there were statistically significant decreases in Kmax (p = 0.012), from a median of 59.20 to 53.90 diopters, and in RMS Coma (p = 0.001), from 2.63 to 1.81 mm. The median decrease in KA, from -3.25 to -1.50 diopters, was not statistically significant (p = 0.896).

**Conclusion:** The implantation of ICRS in keratoconus significantly improves clinical and topographic measures of vision, as well as patients’ quality of life.

**PS133 Vascular 3D printing: from models to simulation devices**

Mickael Bartikian^1^, Angélica Ferreira^1^, Pedro Garrido^2,3^, Luís Mendes Pedro^2,3^, António Gonçalves Ferreira^1,4^, Lia Lucas Neto^1,5^

^1^*Anatomy Department, Faculdade de Medicina, Universidade de Lisboa; Lisbon Academic Medical Centre (CAML),*^2^*Vascular Surgery Department, University Hospital of Santa Maria (HSM-CHLN),*^3^*Faculdade de Medicina, Universidade de Lisboa; Lisbon Academic Medical Centre (CAML),*^4^*Neurosurgery Department, University Hospital of Santa Maria (HSM-CHLN),*^5^*Neuroradiology Department, University Hospital of Santa Maria (HSM-CHLN) E-mail address: mickael.bartikian@gmail.com (Mickael Varoujan Bartikian)*

**Introduction:** The teaching of anatomy is a fundamental part of medical education, especially for radiologists and surgeons. Currently, the vascular system is mainly presented through text and atlas images, lacking the three-dimensionality of cadaver dissections or resin models. Therefore, the reduction in dissection-based teaching in medical training demands alternative and modern approaches to anatomy.

**Aim:** We sought to produce 3D-printed vascular models of the arterial system of the brain, to be used in anatomy classes and made available to students. Furthermore, our goal was to print 3D models of cerebral and renal arteries with malformations, such as aneurysms, and integrate them into an endovascular simulation device to provide interventional radiologists and vascular surgeons better tools to assess, plan, and practice procedures.

**Methods:** We selected Angio-CT scans of healthy subjects and patients with vascular disease. The images were processed with segmentation software to reconstruct cerebral or renal arteries. Segmented volumes were verified and 3D-printed in plastic. For the simulation device, the arterial system was printed with soluble material and coated with transparent silicone. After dissolving the model, we obtained hollow silicone arteries which were connected to a perfusion system to simulate the blood flow and contrast injection.

**Results:** Normal cerebral arterial models were successfully printed with anatomical detail and dimensional accuracy. We also printed a middle cerebral artery aneurysm and six renal artery aneurysms. These models allowed an easy visualisation of the anatomy and real 3D manipulation. The simulation device was built and we tested a mechanical thrombectomy of the internal carotid artery.

**Conclusion:** Image post-processing and 3D-printing technology show great value to modern Medicine. We can reliably create cost-effective vascular models for anatomy classes and students. Interventional radiologists and surgeons can benefit from the integration of 3D-printing in the preoperative evaluation and planning and as a way to communicate more easily with the patient.

**PS137 Total gastrectomy with lymphadenectomy D2 in gastric cancer, totally laparoscopic versus open approach – systematic review and meta-analysis of short-term outcomes**

Grzegorz Torbicz^1^, Natalia Gajewska^1^, Nadia Sajuk^1^, Kamil Rozmus^1^

^1^*Students’ Scientific Group at 2nd Department of General Surgery, Jagiellonian University Medical College E-mail address: grzegorz.torbicz@gmail.com (Grzegorz Torbicz)*

**Introduction:** Worldwide stomach cancer remains the fourth most common cancer and the second cause of cancer deaths. The standard of surgical treatment is subtotal or total gastrectomy with lymphadenectomy D2. The literature comparing laparoscopic vs. open approach in D2 total gastrectomy is sparse.

**Aim:** This study aimed to evaluate short-term outcomes of laparoscopic approach in comparison to open surgery through systematic review with meta-analysis.

**Methods:** The Medline, Embase and Cochrane databases were searched to identify eligible studies. The inclusion criteria are: gastric cancer, total gastrectomy D2, total laparoscopic vs open surgery, paper in English. The outcomes of interest involved morbidity, harvested lymph nodes, operative time, R0 rate and length of hospital stay. Articles lacking comparative data on overall morbidity outcomes, focusing on procedures other than total gastrectomy D2 or if extraction of data was not possible, were excluded.

**Results:** 8 eligible studies were included, with a total of 1,582 patients. The higher postoperative complications were in open group (RR 0.67, 95%CI [0.51, 0.86]). The length of hospital stay was shorter in lap group (MD -2.45, 95%CI [-3.66, -1.24]). There were no differences in operative time (p = 0.68), harvested lymph nodes (p = 0.91) and R0 rate (p = 0.88).

**Conclusion:** Our meta-analysis confirms that laparoscopic total gastrectomy D2 in gastric cancer had better results than open approach in the case of postoperative complications and length of hospital stay.

**PS140 Aortocoronary graft failure according to the degree of native coronary stenosis**

Celina Luca^1^, Ioana Irina Rezus¸^1^, Albert George Nechifor^1^, Andreea Ciolacu^1^, Grigore Tinică^1,2^, Cristina Luca^1,2^

^1^*University of Medicine and Pharmacy ”Gr. T. Popa” Ias¸i,*^2^*Institute for Cardiovascular Diseases “Prof. Dr. George I. M. Georgescu” Ias¸i, Romania E-mail address: celina_luca@yahoo.com (Celina Luca)*

**Introduction:** Using saphenous venous graft (SVG) for coronary arteries revascularization offers many advantages such as availability, accessibility, ease of harvest and resistance to spasm. SVG patency rate has increased due to continuous improvements in surgical techniques combined with antiplatelet or anticoagulant agents and lipid lowering drug therapy.

**Aim:** We evaluated the late patency of SVG and left internal mammary artery (LIMA) grafts according to the native coronary stenotic status using angiographic data.

**Methods:** A total number of 65 patients with clasic myocardial revascularization (mean age of 67.35 ± 7.51 years, range 48–82 years) were enrolled in the present study, and underwent a coronary computed tomography angiography (CCTA) at a mean time of 10.6 ± 1.46 years after surgery. The grafts were classified as patent, occluded, stenotic.

**Results:** A total number of 212 distal anastomoses were performed (mean 3.2 anastomoses per patient). The left internal thoracic artery (LIMA) showed excellent patency, significantly higher than the SVG (p < 0.05). The overall SVG patency was 71.52%. The univariate analysis revealed that the patency was worse for SVG anastomosed on right coronary system than on the left coronary system. Multivariate analysis revealed the target vessel stenotic degree was closely related to LIMA graft failure (p = 0.007, r = 0.371) and was not related to SVG failure (p = 0.435, r = 0.103).

**Conclusion:** Saphenous veins, unlike arterial grafts, are less susceptible to spasm and are less affected by competitive flow and autoregulation. Our data revealed that the SVG showed a good long-term patency that is independent to the target vessel stenosis, and should be preferred for revascularization when the angiography reveals a lower degree of coronary stenosis.

**PS141 Radial artery graft failure according to the degree of native coronary stenosis**

Andreea Ciolacu^1^, Celina Luca^1^, Ioana-Irina Rezus^1^, Albert George Nechifor^1^, Grigore Tinica^1,2^, Cristina Luca^1,2^

^1^*University of Medicine and Pharmacy ”Grigore T. Popa” Iasi, Romania, Faculty of Medicine,*^2^*Institute for Cardiovascular Diseases “Prof. Dr. George I. M. Georgescu” Ias¸i, Romania E-mail address: deea.ciolacu@yahoo.com (Andreea Ciolacu)*

**Introduction:** The radial artery is used as a second or tertiary arterial conduit for surgical myocardial revascularization, together with the internal mammary arteries. Many authors noted that the patency of the radial artery graft (RAG) decreases when grafted to coronary arteries that have mild to moderate obstruction.

**Aim:** We evaluated the patency of the RAG according to the native coronary stenotic status by using coronary computed tomography angiography (CCTA).

**Methods:** A total number of 55 patients with total arterial myocardial revascularization (mean age of 63.76 ± 8.29 years, range 46–77 years) were enrolled in the present study and underwent a postoperative CCTA at a mean period of 7.86 ± 2.66 years after surgery. The arterial grafts were classified as patent, occluded and string arterial grafts.

**Results:** A total number of 175 distal anastomoses were performed (mean 3.18 anastomoses per patient). The left internal thoracic artery (LIMA) showed excellent patency, significantly higher than the RAG (p < 0.05). The RAG overall patency was 80.55%. The univariate analysis revealed that the patency was worse for RAG used for the right coronary system vs. left coronary system. The RAG showed a higher patency rate in the case of a preoperatively critical stenotic lesion, no matter the grafted territory, than in the case of a less severe lesion. Multivariate analysis revealed that the target vessel stenotic degree was closely related to RAG failure (p = 0.008, r = 0.638).

**Conclusion:** Our data revealed that the RAG showed a good long-term patency when grafted to a critical stenotic lesion (over 90%). When the preoperative coronary angiogram reveals less than a 90% coronary stenosis, especially in the right coronary system, a saphenous venous graft should be preferred.

**PS164 Perioperative fluid therapy- does it have an impact on short-term and long-term outcomes in colorectal cancer surgery?**

Natalia Gajewska^1^, Grzegorz Torbicz^1^

^1^*Students’ Scientific Group at 2nd Department of General Surgery, Jagiellonian University Medical College E-mail address: natgajewska92@gmail.com (Natalia Gajewska)*

**Introduction:** Enhanced recovery after surgery (ERAS) protocol have proven to enhance postoperative recovery and reduce postoperative morbidity and length of hospital stay (LOS) after colorectal cancer surgery. One of its several interventions is balanced perioperative fluid therapy. The impact of this single intervention on short-term and long-term outcomes have not been yet establish.

**Aim:** This study aimed to analyse the impact of balanced perioperative fluid therapy on short-term outcomes and long-term survival.

**Methods:** Between November 2011 and May 2017, 402 patients underwent laparoscopic colorectal cancer resection in 2nd Department of General Surgery Jagiellonian University Medical College. Patients were divided into two groups: group 1 with balanced (≤2500 ml) and group 2 with liberal (>2500 ml) perioperative fluid therapy. All patients were treated according to ERAS protocol. Study endpoints were: recovery parameters, morbidity rate, LOS, 30-day readmission rate and 3-year survival.

**Results:** Group 1 consisted of 323 and group 2 of 79 patients. There were no statistically significant differences between the groups in terms of demographic and operative parameters. Our analysis revealed, that balanced perioperative fluid therapy was associated with significantly shorter LOS (4 vs 5 days, p < 0.001), lower rate of postoperative complications (26.9% vs 39.2%, p = 0.031) and improvement in functional recovery parameters: tolerance of oral diet (76.8% vs. 58.2%, p < 0.001) and mobilization (93.5% vs. 78.5%, p < 0.001) on the first postoperative day. 30-day readmission rate was similar in both groups (8.1% vs. 15.2%, p = 0.052). 3-year overall survival was 83% in group 1 and 72% in group 2. Difference in long-term survival between groups was not statistically significant (p = 0.054).

**Conclusion:** A balanced perioperative fluid therapy on the day of surgery may be associated with improvement in recovery parameters, lower morbidity rate and shorter LOS, however does not have an impact on 30-day readmission rate and long-term survival after surgery.

**PS176 Morphometric analysis of radiographic images in patients with Perthes disease**

Olivera Lijeskic’^1^, Nebojša Miloševic’^2^

^1^*Faculty of Medicine, University of Belgrade,*^2^*Institute of Medical Biophysics, Faculty of Medicine, University of Belgrade E-mail address: oliveralijeskic@gmail.com (Olivera Lijeskic*’*)*

**Introduction:** Perthes disease is an avascular necrosis of the proximal femoral epiphysis. It occurs most commonly in childhood, between the ages of 4 and 8 years. There are different radiographic classifications of femoral head damage in patients with this disease. Due to the gradual development of the diseases through four different stages, it is desirable to diagnose and start treatment as soon as possible.

**Aim:** The aim of this study was to verify the possibility of applying modern techniques to the analysis of radiographic images (binary and grayscale) of the lateral pillar of the femoral head, in order to differentiate the structure of the femoral head of patients with a normal finding and patients with diagnosed Perthes disease.

**Methods:** This study analyzed the radiographic images of the femoral head of 63 children, aged 7 to 13 years. The images were divided into two groups, a control group and a group of patients with diagnosed Perthes disease, and were further analyzed in the “Image J” program. For the texture analysis of grayscale images, the GLCM (Gray Level Co-occurrence Matrix) technique was used, and the box-counting method was used for fractal analysis of binary images.

**Results:** For four parameters of fractal analysis (Dbin, Dskel, Dnorm, L) a statistically significant difference was observed between the group with Perthes disease and control group. For textural parameters, there was no statistically significant difference except for two parameters SCON and SASM.

**Conclusion:** The results showed that fractal analysis is more suitable for application than texture analysis in this sample. Further research should explore the possibility of using fractal and texture analysis in the quantification of the pathological process and then application of the obtained data to existing radiographic categories in order to expand them and improve diagnostics.

**PS231 The risk of injury of the sural nerve during posterolateral approach to the distal Tibia. A USG study**

Mateusz Paziewski^1^, Ewa Mizia^1^, Przemysław Pe¸kala^1^, Jerzy Walocha^1^, Piotr Chomicki-Bindas^1^, Krzysztof Tomaszewski^1^

^1^*Department of Anatomy, Jagiellonian University Medical College, 12 Kopernika St, 31– 034 Kraków, Poland E-mail address: mateusz19paziewski@gmail.com (Mateusz Paweł Paziewski)*

**Introduction:** The posterolateral approach to the distal tibia is the surgical technique of choice for the treatment of injuries of the ankle. During this procedure, one of the most common complications is damage to the sural nerve [SN].

**Aim:** The aim of this study was to estimate how variation in length and location of the surgical incision, in the posterolateral approach, may affect the risk of SN damage.

**Methods:** Forty people were recruited for this study (n = 80 lower limbs). An ultrasound simulation was utilized to locate the course of the SN relative to the fibular bone and Achilles tendon and its deviation in relation to the midline marked between these structures. The risk of injury to the SN, during posterolateral approach, has been estimated and presented on a quasi-three-dimensional figure illustrating the anatomical structures of this area.

**Results:** At the proximal level from the tip of the lateral malleolus the SN runs closer to the Achilles tendon. It is proven that there is an increased risk of iatrogenic injuries of the SN in incisions made closer to the Achilles tendon. At the level of the tip of the lateral malleolus the risk of SN damage increased from 66.3% 2.0 cm from the midline towards the fibula to 100% 2.0 cm from the midline towards the Achilles tendon (in relation to the midline between these structures at the level of the tip of the lateral malleolus) (p < 0.001).

**Conclusion:** The Sural Nerve carries an increased risk of iatrogenic injuries. Surgeons should take this under consideration during posterolateral approach to the distal tibia. The model created in this study will help raise clinical awareness and help better plan the incision sites to avoid SN damage.

**PS240 The relevance of Short-Term Variation (STV) value measured within 1 hour before delivery in predicting adverse neonatal outcomes**

Gabriela Wilczynska^1^, Agata Staron^1^, Mirella Brzozowska^1^

^1^*Department of Obstetrics and Perinatology, Jagiellonian University Medical College in Cracow E-mail address: gabriela-wilczynska@wp.p (Gabriela Wilczynska)*

**Introduction:** Cardiotocography (CTG) is one of the basic examinations in obstetrics, which enables the assessment of foetal heart rate and uterine contraction. CTG computer analysis is one of the methods of monitoring foetal condition during delivery. Short-term variation (STV) is the variation in length of consecutive foetal heart cycles. Low STV values are associated with increased risk of metabolic acidosis and intrauterine death.

**Aim:** The aim of our study was to define appropriability of STV measured within 1 hour before delivery in prediction of neonatal outcomes.

**Methods:** The retrospective study included 1014 pregnant women of gestational age from 24 to 41 weeks, who gave birth in the Department of Obstetrics and Perinatology, Jagiellonian University Medical College in Cracow, Poland between March and December 2017. Inclusion criteria were: singleton pregnancy and continuous monitoring of CTG in the last hour before delivery. Exclusion criteria consisted of multiple pregnancies and planned caesarean section. Participants were divided into two groups: group 1 (<37 weeks of pregnancy) and group 2 (37–41 weeks). In each of them, two subgroups have been separated: control (STV ≥ 3 ms) and study group (STV < 3 ms). Differences in socio-demographic factors between subgroups were not statistically significant. Data was analysed using Student's t-test; p < 0,05 was statistically significant.

**Results:** In on-term pregnancies (37–41 weeks) both control and study groups presented no statistically significant differences (p > 0,05) in Apgar score in 1st, 3rd and 5th minute after delivery. Moreover, for 37–41 weeks the sensitivity, specificity, positive predictive value and negative predictive value were: 22,7%, 83,9%, 3,3% and 97,8% and for lower than 37: 45,7%, 65,4%, 47,1%, 64,2% respectively.

**Conclusion:** In case of normal STV value, there is a high probability of good neonatal outcomes. High specificity and negative predictive value in case of pregnancy at term indicates good condition of newborn child. Low STV value does not correlate with bad neonatal outcomes.

STV is not a good predictor in pregnancies lower than 37 weeks.

**Surgery**

**PS242 Evaluation of clinical factors independently associated with new language deficite after awake craniotomy for intrisic brain tumor**

Aneta Myszka^1^, Roger Krzyz.ewski^2^, Borys Kwinta^2^

^1^*Students’ Scientific Group at Department of Neurosurgery and Neurotraumatology Jagiellonian University Medical College,*^2^*Department of Neurosurgery and Neurotraumatology Jagiellonian University, Medical College E-mail address: anetamyszka95@gmail.com (Aneta Myszka)*

**Introduction:** Awake craniotomy for brain leasions in or near eloquent brain regions enables neurosurgeons to asses neurologic function of patients intraoperatively, reducing the risk of permanent neurologic defficits and increasing the extent of resection. Nevertheless this kind of the surgical treatment is associated with higher risk of language impairment. There are many preoperative risk factors which pose significant impact to postoperative eloquent functions.

**Aim:** The aim of this study was to corelate preoperative risk factors with appearance of language deficite in patients underwent awake craniotomy.

**Methods:** In this study we retrospectively and prospectively enrolled 23 patients underwent awake craniotomy in our hospital in case of supratentorial intraaxial brain tumors between 2014 to 2018. We analyzed the treatment results, as well as pre-and post operative risk factors.

**Results:** Stud group consisted of 23 patients. Ten (43%) patients developed new language deficite, of which 7 were transient and 3 were permament. Patients with new language deficite had higher frequency of hypertension (60.00% vs. 15.38%; p = 0.026) and Mallapati score > 2 (100.00% vs. 45.45%; p = 0.057). Those patients also had higher values of preoperative Caprini score (3.70 ± 1.57 vs. 2.15 ± 2.08; p = 0.063) and were older (56.70 ± 11.57 years vs. 43.31 ± 16.81 years; p = 0.043). In multivariant logistic regression analysis after adjustment for possible confounders pre-existing hypertension (OR: 8.25, 95%CI: 1.02–66.54; p = 0.036) remained independently associated with development of new language deficite after awake craniotomy for intrisic brain tumor.

**Conclusion:** Pre-existing hypertension is independently associated with new language deficit after awake brain tumor surgery. Higher age, Mallampati score and Caprini score might associated with developemnt of new language deficit after awake brain tumor surgery.

